# GRAFENE: Graphlet-based alignment-free network approach integrates 3D structural and sequence (residue order) data to improve protein structural comparison

**DOI:** 10.1038/s41598-017-14411-y

**Published:** 2017-11-02

**Authors:** Fazle E. Faisal, Khalique Newaz, Julie L. Chaney, Jun Li, Scott J. Emrich, Patricia L. Clark, Tijana Milenković

**Affiliations:** 1 Department of Computer Science and Engineering, University of Notre Dame, Notre Dame, IN 46556 USA; 2 Department of Chemistry and Biochemistry, University of Notre Dame, Notre Dame, IN 46556 USA; 3 Department of Applied and Computational Mathematics and Statistics, University of Notre Dame, Notre Dame, IN 46556 USA; 4 Department of Chemical and Biomolecular Engineering, University of Notre Dame, Notre Dame, IN 46556 USA; 5 Interdisciplinary Center for Network Science and Applications, University of Notre Dame, Notre Dame, IN 46556 USA; 6 Eck Institute for Global Health, University of Notre Dame, Notre Dame, IN 46556 USA

**Keywords:** Network topology, Protein structure predictions

## Abstract

Initial protein structural comparisons were sequence-based. Since amino acids that are distant in the sequence can be close in the 3-dimensional (3D) structure, 3D contact approaches can complement sequence approaches. Traditional 3D contact approaches study 3D structures directly and are alignment-based. Instead, 3D structures can be modeled as protein structure networks (PSNs). Then, network approaches can compare proteins by comparing their PSNs. These can be alignment-based or alignment-free. We focus on the latter. Existing network alignment-free approaches have drawbacks: 1) They rely on naive measures of network topology. 2) They are not robust to PSN size. They cannot integrate 3) multiple PSN measures or 4) PSN data with sequence data, although this could improve comparison because the different data types capture complementary aspects of the protein structure. We address this by: 1) exploiting well-established graphlet measures via a new network alignment-free approach, 2) introducing normalized graphlet measures to remove the bias of PSN size, 3) allowing for integrating multiple PSN measures, and 4) using ordered graphlets to combine the complementary PSN data and sequence (specifically, residue order) data. We compare synthetic networks and real-world PSNs more accurately and faster than existing network (alignment-free and alignment-based), 3D contact, or sequence approaches.

## Introduction

### Motivation and related work

Proteins perform important cellular functions. While understanding protein function is clearly important, doing so experimentally is expensive and time-consuming^1,2^. Because of this, the functions of many proteins remain unknown^2,3^. Consequently, computational prediction of protein function has received attention. In this context, protein structural comparison (PC) aims to quantify similarity between proteins with respect to their sequence or 3-dimensional (3D) structural patterns. Then, functions of unannotated proteins can be predicted based on functions of similar, annotated proteins. By “function”, we mean traditional notions of protein function, such as its biological process, molecular function, or cellular localization^4^, or any protein characteristic (e.g., length, hydrophobicity/hydrophilicity, or folding rate), as long as the given characteristic is expected to correlate well with the protein structure. In this study, we propose a new PC approach, which we evaluate in an established way: by measuring how accurately it captures expected (dis)similarities between known groups of structurally (dis)similar proteins^5^, such as protein structural classes from Class, Architecture, Topology, Homology (CATH)^6,7^, or Structural Classification of Proteins (SCOP)^8^. Application of our proposed PC approach to protein function prediction is out of the scope of the current study and is the subject of future work.

Early PC has relied on sequence analyses^9–11^. Due to advancements of high-throughput sequencing technologies, rich sequence data are available for many species, and thus, comprehensive sequence pattern searches are possible. However, amino acids that are distant in the linear sequence can be close in 3D structure. Thus, 3D structural analyses can reveal patterns that might not be apparent from the sequence alone^12^. For example, while high sequence similarity between proteins typically indicates their high structural and functional similarity^3^, proteins with low sequence similarity can still be structurally similar and perform similar function^13,14^. In this case, 3D structural approaches, unlike sequence approaches, can correctly identify structurally and thus functionally similar proteins. On the other extreme, proteins with high sequence similarity can be structurally dissimilar and perform different functions^15–19^. In this case, 3D structural approaches, unlike sequence approaches, can correctly identify structurally and thus functionally different proteins.

3D structural approaches can be categorized into traditional 3D contact approaches, which are alignment-based, and network approaches, which can be alignment-based or alignment-free. By alignment-based (3D contact or network) approaches, we mean approaches whose main goal is to map amino acid residues between the compared proteins in a way that conserves the maximum amount of common substructure. In the process, alignment-based approaches can and typically do quantify similarity between the compared protein structures, and they do so *under their resulting residue mappings*. Given this, alignment-based approaches can and have been used in the task of PC as we define it^5,20^, even though they are not necessarily directly designed for this task. On the other hand, by alignment-free (network) approaches, we mean approaches whose main goal is to quantify similarity between the compared protein structures *independent of any residue mapping*, typically by extracting from each structure some network patterns (also called network properties, network features, network fingerprints, or measures of network topology) and comparing the patterns between the structures. Alignment-free approaches are directly designed for the task of PC as we define it. We note that there exist approaches that are alignment-free but not network-based^21^, which are out of the scope of our study. Below, we discuss 3D contact alignment-based PC approaches, followed by network alignment-based PC approaches, followed by network alignment-free PC approaches.

*3D contact alignment-based PC approaches* are typically rigid-body approaches^22,23^, meaning that they treat proteins as rigid objects. Such approaches aim to identify alignments that satisfy two objectives: 1) they maximize the number of mapped residues and 2) they minimize deviations between the mapped structures (with respect to e.g., Root Mean Square Deviation). Different rigid-body approaches mainly differ in how they combine these two objectives. There exist many approaches of this type^5,24–26^. Prominent ones are DaliLite^27^ and TM-align^28^. These two approaches have explicitly been used and evaluated in the task of PC as we define it^5,20^, and are thus directly relevant for our study.

*Network alignment-based PC approaches* are typically flexible alignment methods, meaning that they treat proteins as flexible (rather than rigid) objects, because proteins can undergo large conformational changes. These approaches align local protein regions in a rigid-body manner but account for flexibility by allowing for twists between the locally aligned regions^5,29^. Also, these approaches are typically of the Contact Map Overlap (CMO) type. That is, first, they represent a 3D protein structure consisting of *n* residues as a contact map, i.e., *n* × *n* matrix *C*, where position *C*_*ij*_ has a value of l if residues *i* and *j* are close enough and are thus in contact, and it has a value of 0 otherwise. Note that contact maps are equivalent to protein structure networks (PSNs), in which nodes are residues and edges link spatially close amino acids^30^. Second, CMO approaches aim to compare contact maps of two proteins, in order to align a subset of residues in one protein to a subset of residues in another protein in a way that maximizes the number of common contacts and also conserves the order of the aligned residues^31^. Prominent CMO approaches are Apurva, MSVNS, AlEigen7, and GR-Align^5^. When evaluated in the task of PC as we define it, i.e., when used to compare proteins labeled with structural classes of CATH or SCOP, GR-Align outperformed Apurva, MSVNS, and AlEigen7 in terms of both accuracy and running time^5^. So, we consider GR-Align to be the state-of-the-art CMO (i.e., network alignment-based) approach. In addition to these network alignment-based approaches, GR-Align was evaluated in the same manner against the existing 3D contact alignment-based approaches (DaliLite and TM-Align mentioned above, as well as three additional approaches, MATT, Yakusa, and FAST)^5^. In terms of running time, GR-Align was the fastest. In terms of accuracy, GR-Align was superior to MATT, Yakusa, and FAST, but it was inferior or comparable to DaliLite and TM-Align. So, while GR-Align remains the state-of-the-art network alignment-based PC approach, DaliLite and TM-Align remain state-of-the-art 3D contact alignment-based PC approaches, and we continue to consider all three in our study.

*Network alignment-free approaches* also deal with PSN representations of compared proteins, but they aim to quantify protein similarity without accounting for any residue mapping. We propose a novel network alignment-free PC approach (see below). We first compare our approach to its most direct competitors, i.e., existing alignment-free approaches. Then, we compare our approach to existing alignment-based approaches. We recognize that evaluation of alignment-free against alignment-based approaches should be taken with caution^32,33^, (because the two comparison types quantify protein similarity differently – see above). Yet, as we show later in our evaluation, existing alignment-based PC approaches are superior to existing alignment-free PC approaches and are thus our strongest (though not necessarily fairest) competitors.

Next, we discuss existing network alignment-free PC approaches. Such approaches have already been developed^14,34^, to compare network topological patterns within a protein or across proteins, for example to study differences in network properties between transmembrane and globular proteins, analyze the packing topology of structurally important residues in membrane proteins, or refine homology models of transmembrane proteins^35–37^. Existing network alignment-free PC approaches, however, have the following limitations:

1. They rely on naive measures of network topology, such as average degree, average or maximum distance (diameter), or average clustering coefficient of a network, which capture the global view of a network but ignore complex local interconnectivities that exist in real-world networks, including PSNs^38–40^.
2. They can bias PC by PSN size: networks of similar topology but different sizes can be mistakenly identified as dissimilar by the existing approaches simply because of their size differences alone.
3. Because different network measures quantify the same PSN topology from different perspectives^41^, and because each existing approach uses a single measure, PC could be biased towards the perspective captured by the given measure.
4. They ignore valuable sequence information (also, the existing sequence approaches ignore valuable PSN information).

### Our contributions

We present a new network alignment-free PC framework that overcomes the above limitations. Specifically:

1. We extend graphlets^42,43^, a sensitive measure of *local* network topology, to improve PC. Graphlets have already been used for alignment-free comparisons of protein-protein interaction networks^44–46^, via graphlet degree distribution agreement (GDDA)^43^, relative graphlet frequency distance (RGFD)^42^, and graphlet correlation distance (GCD)^47^ approaches. While these three graphlet approaches can trivially be used in the task of PC (which we do in our study), we use graphlets differently. Namely, we summarize the topology of a PSN (i.e., the corresponding protein) into a graphlet vector. Then, we use graphlet vectors of all considered PSNs as input to principal component analysis (PCA) to reduce the dimensionality of the vectors in a way that keeps only the most important (discriminative) information from the vectors. Finally, we quantify the similarity between the PSNs by comparing their PCA dimensionality-reduced graphlet vectors. The combination of graphlets and PCA is a novel PC approach.
2. We perform graphlet normalization to address the bias of PSN size.
3. We allow for integrating different and complementary network topological measures within our PCA framework.
4. We extend the idea of *ordered graphlets*^5^ to integrate the PSN amino acid interconnectivity information with sequence (i.e., residue order) information. Ordered graphlets were already used by GR-Align^5^. However, even though GR-Align is also a network PC approach and even though GR-Align also uses ordered graphlets, one key difference between GR-Align and our work is that GR-Align is alignment-based, while our graphlet PCA framework is alignment-free. This is expected to provide drastic speed-up compared to GR-Align (and it does, as we verify in our evaluation), because our approach does not need to actually map residues between compared proteins. Yet, if one wished to actually map residues, using an alignment-free approach such as ours would not suffice. Another key difference between GR-Align and our work is that GR-Align uses only up to 3-node ordered graphlets. Using larger graphlets can be beneficial in many real-world contexts^41,44–46^. Hence, we extend the existing notion of 3-node ordered graphlets both theoretically and implementation-wise to be able to deal with larger graphlets. Additionally, we introduce a novel concept of *“long-range(K)” ordered graphlets* to give higher importance to amino acids that are close enough in the protein 3D structure but are at least *K* amino acids apart in the protein sequence, because such longer-range interactions could help distinguish protein structures better^12,48^. We include the extended idea of (larger size, normalized, and “long-range(*K*)”) ordered graphlets into our software (available at: http://nd.edu/~cone/PSN/).

**Figure 1:**
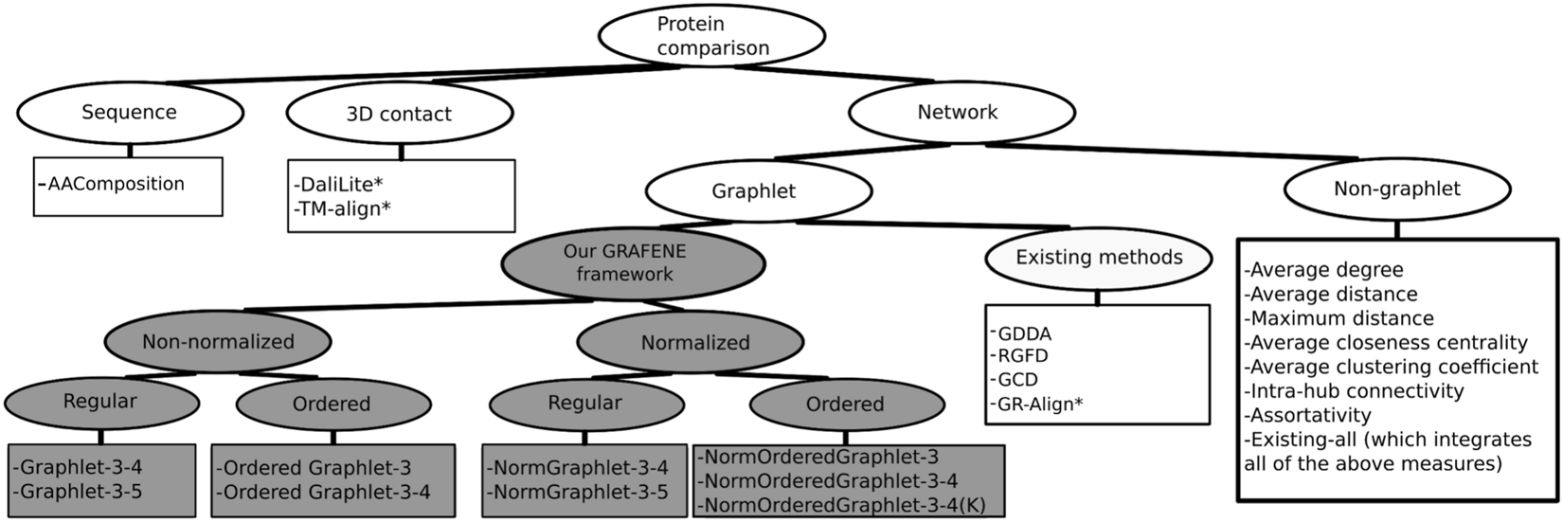
Categorization of the 24 approaches (listed in squares) that we evaluate. Different versions of our graphlet PCA approach are colored in grey. Alignment-based approaches are marked with “*”; all other approaches are alignment-free.

We refer to our entire PC framework as GRAFENE (**gr**aphlet-based **a**lignment-**f**r**e**e **ne**twork comparison), which has nine different approach versions, depending on graphlet size, whether graphlets are normalized, whether graphlets are ordered, and whether the “long-range(*K*)” constraint is considered (see Fig. 1 and Methods).

We study two network types: synthetic networks (in order to illustrate wide applicability of our approach across many domains) and real-world PSNs (in order to illustrate a specific application of our approach in the task of PC). For each network type, we analyze multiple data sets. In each data set, each network has a known label, meaning that we know that networks having the same label should be identified as similar, while networks having different labels should be identified as dissimilar. For synthetic networks, labels correspond to different random graph models. For real-world PSNs, labels correspond to structural classes from CATH or SCOP, where we study all four levels of CATH and SCOP hierarchies (see Methods). We study 21 network approaches (Fig. 1): the nine versions of our proposed GRAFENE approach and 12 existing approaches. Of the 12 existing network approaches, four use graphlets, namely GDDA, RGFD, GCD, and GR-Align, and eight do not use graphlets. Also, of the 12 existing network approaches, only GR-Align is alignment-based, and the others are alignment-free. In addition to the 21 network approaches, we also study the two prominent 3D contact alignment-based approaches (DaliLite and TM-align) and a sequence-based approach (namely, amino acid composition, AAComposition, which has been used in several tasks, including PC^9^, i.e., discriminating between different structural classes and folding types^49^, as well as protein function prediction^50^). Note that not all of the 21 + 2 + 1 = 24 approaches are applicable to synthetic networks. This is because nine of the 24 approaches require sequence residue order or 3D structural information, which synthetic networks do not contain. All 24 approaches are applicable to real-world PSNs.

Given a data set and an approach, we compute similarity/distance between each pair of networks/proteins. We evaluate each approach by measuring how accurately it can identify networks/proteins of the same label as similar and networks/proteins of different labels as dissimilar. We measure this by computing the area under precision-recall curve (AUPR) and area under receiver operator characteristic curve (AUROC), which is an established PC evaluation framework that was used by e.g., GR-Align^5^. Also, we evaluate each method’s running time. For details, see Methods.

Our key findings are as follows. *For synthetic networks*, our GRAFENE approach (the larger size, normalized, and regular, i.e., non-ordered, graphlet version – NormGraphlet-3-5) is superior to all eight evaluated existing alignment-free network approaches that have been used in the task of PC, none of which use graphlets. This already justifies the introduction of our approach. Also, GRAFENE (the same version – NormGraphlet-3-5) is superior to all three evaluated existing graphlet alignment-free approaches that are general-purpose network comparison tools (GDDA, RGFD, and GCD), indicating that our approach is advantageous for general-purpose network comparison tasks. Similarly, *for real-world PSNs*, GRAFENE (the larger size, normalized, ordered graphlet, and “long-range(*K*)” version – NormOrderedGraphlet-3-4(K)) is superior to all evaluated approaches. For details, see Results.

## Methods

Because of space constraints, we provide only the most relevant methodological information here. All additional information, in enough detail to ensure the reproducibility of our results, is presented in the Supplementary Information.

### Data

We collect from the Protein Data Bank (PDB)^51^ 3D atomic structures of all 17,036 non-redundant proteins and denote this data set as *ProteinPDB* (Supplementary Section S1). A protein is typically composed of one or more domains (a domain refers to a part of a protein structure that can fold and often function independently). Class, Architecture, Topology, Homology (CATH) and Structural Classification of Proteins (SCOP) are independent databases of categorized (annotated) protein domains^6–8^.

### Forming networks

We evaluate the considered approaches in the task of PC on: 1) synthetic networks, i.e., artificially generated networks for which we know the topology-based ground truth categorization, and 2) real-world PSNs, for which we know CATH- or SCOP-based categorizations that we hypothesize correlate well with the PSNs’ topology-based characteristics.

#### Synthetic networks

We generate synthetic networks by using different network models. A good general approach should identify networks from the same model (i.e., with the same label) as similar, and it should identify networks from different models (i.e., with different labels) as dissimilar. We use three well-established models: *Erdös-Rényi random graphs (ER)*, *geometric random graphs (GEO)*, and *scale-free random graphs (SF)*^38,40^.

First, we aim to analyze a synthetic network set in which all networks are of the same size but have different labels. We study individually three network sets of this type, which differ from each other in terms of the sizes of their networks. The three sets are: *Synthetic-100*, *Synthetic-500*, and *Synthetic-1000*, reflecting the number of nodes in the network (Supplementary Section S2 and Supplementary Table S1).

Second, we aim to analyze networks of different sizes and different labels, to check whether an approach can correctly identify: 1) as similar networks from the same model despite the networks being of different sizes, and 2) as dissimilar networks from different models despite the networks being of the same size. To generate a synthetic network set of different sizes, we combine Synthetic-100, Synthetic-500, and Synthetic-1000, resulting in *Synthetic-all* (Supplementary Table S1).

The synthetic network sets allow us to evaluate our proposed GRAFENE approach against existing network comparison approaches without having available any sequence or 3D structural information.

#### Forming real-world PSNs

Each protein in ProteinPDB (defined above) is composed of 3D coordinates of the heavy atoms of its amino acids. Given a protein, we use its 3D coordinate information to construct its PSN in which nodes represent amino acid residues and edges connect pairs of amino acid residues that are sufficiently close (i.e., within a given Euclidean distance cut-off) in the protein’s 3D structure. Clearly, PSN construction depends on two parameters: 1) the atom type that is considered to represent an amino acid residue as a node in the PSN, and 2) the distance cut-off threshold that determines whether two amino acids (i.e., their atoms defined in step 1 above) are close enough and thus form an edge in the PSN. Different choices of these parameters can result in different PSNs and consequently affect the performance of a network-based PC approach. To evaluate this effect, we consider four PSN construction strategies (i.e., combinations of the above two parameters), as follows.

In three out of the four PSN construction strategies that we use, regarding the choice of atom type, we consider for the given amino acid *any* of its heavy atoms, as is often done^30^. Regarding the choice of distance cut-off threshold, while effective definitions of contact between amino acids may differ from fold to fold^52^, we use suggested distance cut-offs in the 4 Å–6 Å range (because when considering any atom type, cut-offs below 4 Å result in highly disconnected PSNs, while cut-offs beyond 6.5 Å result in random-like PSN structures^30^). Specifically, in this range, we consider cut-offs of 4 Å, 5 Å, and 6 Å.

The remaining (fourth) PSN construction strategy that we use is the default strategy of GR-Align^5^, which (as we show later) is the best existing network PC approach in our evaluation. Specifically, we use the *α*-carbon atom type and the 7.5 Å distance cut-off. For additional discussion about these parameter choices, see Supplementary Section S3.

#### Real-world PSNs with CATH categorization

**Figure 2:**
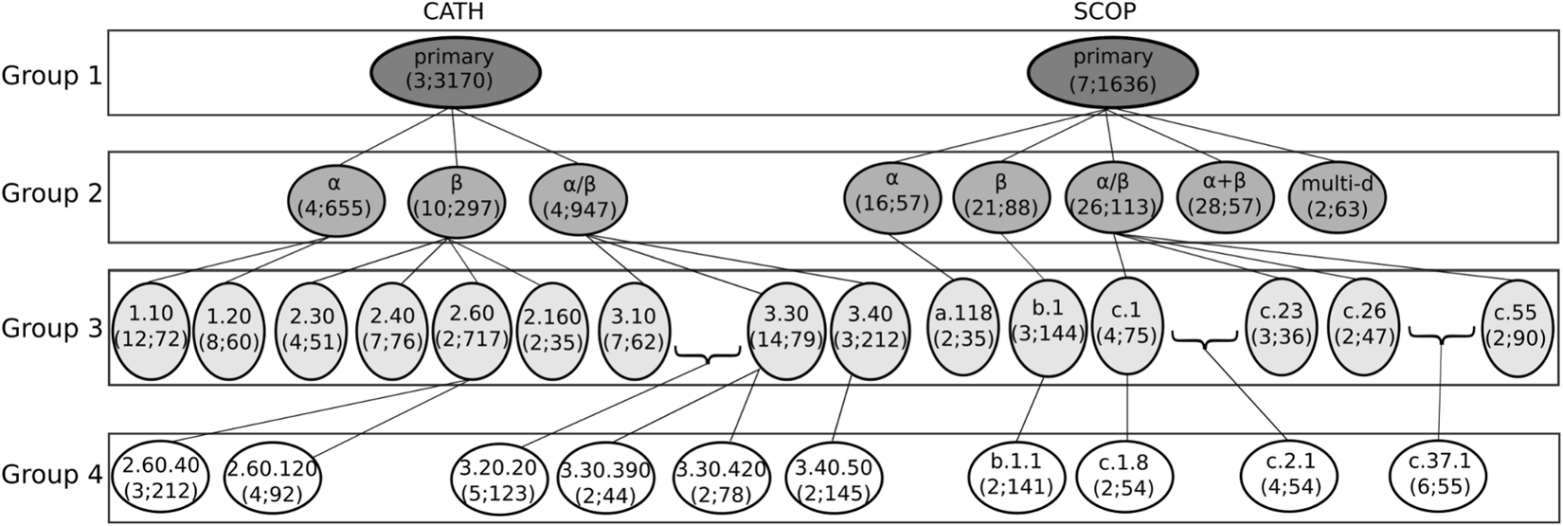
Hierarchical representation of the 35 real-world PSN sets that we use. Each oval shape represents a PSN set. The top line in the given oval indicates the name of the PSN set. The bottom line in the given oval contains two numbers represented as “*x*; *y*”, where *x* is the number of categories (labels) that are present in the PSN set and *y* is the number of PSNs averaged over all categories in the PSN set. For example, for the CATH database, PSN set CATH-primary has three categories, which on average have 3,170 PSNs. All of the PSN sets at a given level form a PSN set group (see Methods). For example, PSN sets CATH-primary and SCOP-primary form PSN set group 1. A given category of a PSN set in group $i$ may be present as a PSN set in group $i+1$. For example, each of the categories of PSN set CATH-primary (in group 1), i.e., $\alpha$, $\beta$, and $\alpha /\beta$, is present as a PSN set in group 2 as CATH-$\alpha$, CATH-$\beta$, and CATH-$\alpha /\beta$. Note that since we select a PSN set if and only if it has at least two categories each with at least 30 PSNs (see Methods), not all of the categories of a PSN set in group $i$ are necessarily present as PSN sets in group $i+1$. For example, PSN set CATH-$\alpha$ has four categories in group 2, but only two of its categories exist as PSN sets in group 3, namely CATH-1.10 and CATH-1.20. Also note that because of our PSN set selection criterion, it is not necessary that a PSN set in group $i+1$ has to be present as a category of a PSN set in group $i$. For example, PSN set CATH-3.20.20, which is present in group 4, is not present as a category of any PSN set in group 3. This is because CATH-3.20 contains only one category that has at least 30 PSNs (i.e., 3.20.20) and hence, CATH-3.20 is not considered as a PSN set in our analysis.

We analyze all 9,509 protein domains (i.e., their corresponding PSNs) from ProteinPDB that have a CATH categorization (i.e., label) (Supplementary Section S4 and Supplementary Table S2). At each of the four CATH hierarchy levels, for each CATH category (which we refer to as a *PSN set*), we test how well each considered PC approach can compare PSNs between the different lower-level subcategories. We analyze all $1+3+9+\mathrm{6=19}$ CATH PSN sets across the four levels (Fig. 2, Supplementary Section S4, and Supplementary Tables S3–S5).

#### Real-world PSNs with SCOP categorization

Also, we analyze all 11,451 protein domains (i.e., their corresponding PSNs) from ProteinPDB that have a SCOP categorization (Supplementary Section S5 and Supplementary Table S2). Just as with CATH, we analyze all four hierarchy levels of SCOP. This results in $1+5+6+4\,=\,16$ SCOP PSN sets across the four levels (Fig. 2, Supplementary Section S5, and Supplementary Tables S3–S5).

#### Real-world PSN set groups

Over both CATH and SCOP, we analyze $19+16\,=\,35$ PSN sets. We partition the 35 PSN sets into four PSN set groups (Fig. 2): group 1 (all $1+1\,=\,2$ PSN sets in which we compare PSNs between the top-level categories of CATH or SCOP), group 2 (all $3+5=8$ PSN sets in which we compare PSNs between the second-level categories of CATH or SCOP), group 3 (all $9+6=15$ PSN sets in which we compare PSNs between the third-level categories of CATH or SCOP), and group 4 (all $6+4\,=\,10$ PSN sets in which we compare PSNs between the fourth-level categories of CATH or SCOP). Also, we denote by “all groups” the PSN set group that contains all 35 PSN sets of CATH and SCOP.

#### Real-world PSNs of the same size

To study the bias of PSN size, we need a data set with PSNs of the same (or similar) network size. Hence, focusing on PSNs of *α* and *β* categories from the CATH-primary PSN set (Fig. 2), we infer three such same-size PSN sets, denoted as *CATH-95*, *CATH-99*, and *CATH-251-265* (Supplementary Section S6). We denote by “equal size” the group consisting of these three PSN sets.

### Our GRAFENE framework

#### The PCA component of GRAFENE

The novelty of GRAFENE comes from combining graphlet-based measures of network topology with principal component analysis (PCA) (Fig. 1). Yet, GRAFENE is generalizable, as it can use any measure(s). For a given measure, given a network set, GRAFENE first computes one vector per network. Then, it performs PCA (a standard dimension reduction technique) on the resulting vectors to compute principal components for each network. We pick the first *r* principal components, where the value of *r* is at least two and as low as possible so that the *r* components account for at least 90% of variation in the data. For every pair of networks ${N}_{i}$ and ${N}_{j}$, we compute their cosine similarity, ${s}^{cos}({N}_{i},{N}_{j})$, based on the networks’ first *r* principal components. We convert the similarity into distance as ${d}^{cos}({N}_{i},{N}_{j})=1-{s}^{cos}({N}_{i},{N}_{j})$. We use the PCA-based distances to hypothesize that same-label networks will be close in the PCA space while networks of different labels will be distant. Like most of the network approaches from Fig. 1, GRAFENE performs alignment-free network comparison.

#### Our graphlet measures

**Figure 3:**
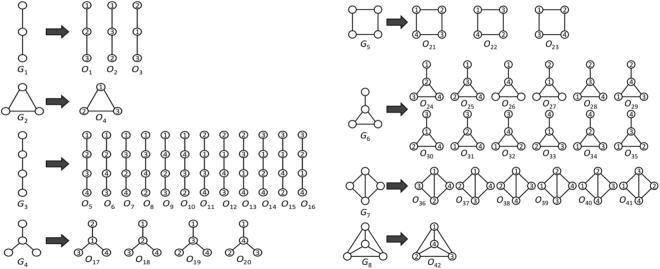
All possible eight regular (non-ordered) 3-4-node graphlets (${G}_{1},{G}_{2}\mathrm{,...,}{G}_{8}$; on the left of the given arrow) and their corresponding 42 ordered graphlets (${O}_{1},{O}_{2}\mathrm{,...,}{O}_{42}$; on the right of the given arrow).

Graphlets are small connected *induced* subgraphs (Fig. 3). They are proven to be sensitive and superior measures of topology when studying protein-protein interaction networks^5,41–43,53,54^. We use graphlets as PSN measures for PC, as follows.

#### *Graphlet counts*

**Figure 4:**
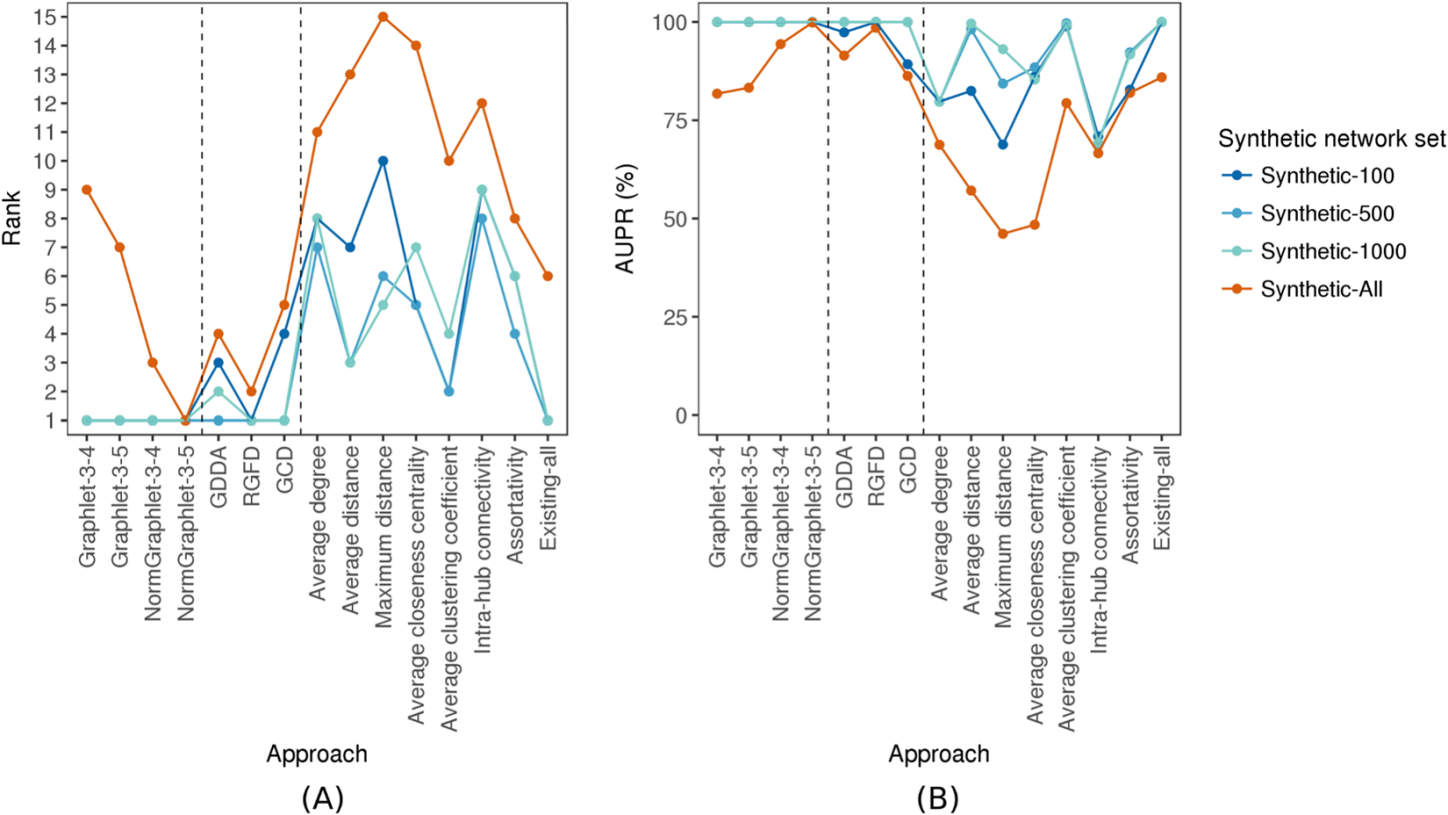
The performance comparison of the 15 considered approaches on each of the four considered synthetic network sets, with respect to AUPR, in terms of: (**A**) the approaches’ ranks compared to one another, and (**B**) the approaches’ raw AUPR values. In panel (A), for a given synthetic network set, the 15 approaches are ranked from the best (rank 1) to the worst (rank 15). So, the lower the rank, the better the approach. In panel (B), for each approach, its raw AUPR value is shown for each of the four synthetic network sets. So, the higher the AUPR value, the better the approach. For equivalent results with respect to AUROC values, see Supplementary Fig. S2.

**Figure 5:**
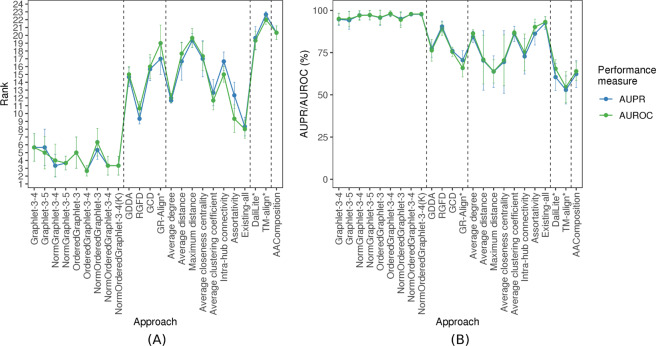
The performance comparison of the 24 considered approaches, averaged over all three considered real-world PSN sets of same network sizes (that form the “equal size” PSN set group), with respect to AUPR/AUROC, in terms of: (**A**) the approaches’ ranks compared to one another, and (**B**) the approaches’ raw AUPR/AUROC values. In panel (A), for a given PSN set, the 24 approaches are ranked from the best (rank 1) to the worst (rank 24). Then, for a given approach, its three ranks (corresponding to the three PSN sets) are averaged (the average ranks are denoted by circles, and bars denote the corresponding standard deviations). So, the lower the average rank, the better the approach. In panel (B), for each approach, its three raw AUPR/AUROC values (corresponding to the three PSN sets) are averaged (the average values are denoted by circles, and bars denote the corresponding standard deviations). So, the higher the average AUPR/AUROC value, the better the approach.

We count occurrences of each graphlet on up to *n* nodes in the given network. To investigate the best choice for *n*, we use counts for 3-4-node (Fig. 3) and 3–5–node graphlets, resulting in *Graphlet-3-4* and *Graphlet-3-5* measures (i.e., GRAFENE versions), respectively. Graphlet counts typically vary by orders of magnitude in real-world networks^42^. Hence, we normalize graphlet counts by taking their logarithms. Here, we do not consider 3-node-only graphlets, because there are only two 3-node graphlets, which may not be suitable for our PCA-based GRAFENE framework, and also because using up to 4- or 5-node graphlets improves accuracy upon using only 3-node graphlets^44–46^.

#### *Normalization of graphlet counts*

Networks with similar topology can have dissimilar graphlet counts simply because of their dissimilar network sizes (see Results). To remove the bias of PSN size, we normalize graphlet counts by scaling them between 0 and 1. Formally, given a network, let ${g}_{1},{g}_{2}\mathrm{,...,}{g}_{n}$ be counts of *n* graphlets ${G}_{1},{G}_{2}\mathrm{,...,}{G}_{n}$, respectively ($n=8$ for 3-4-node graphlets and $n=29$ for 3-5-node graphlets). We normalize count ${g}_{i}$ of graphlet *G*_*i*_ as ${g}_{i}/{\sum }_{j=1}^{n}{g}_{j}$. We denote the normalized Graphlet-3-4 and Graphlet-3-5 measures as *NormGraphlet-3-4* and *NormGraphlet-3-5*, respectively.

#### **Integration of graphlets with residue order in the protein sequence: ordered graphlet counts**

While amino acids appear in a particular order in the sequence, graphlets were not originally designed to capture this node order information. For example, nodes in graphlet *G*_1_ can appear in three different orders (Fig. 3), but *G*_1_ cannot differentiate between them. To take advantage of both network and sequence data, *ordered graphlets* were recently proposed^5^, which embed the *relative* order of nodes onto graphlets. For example, the three different orders of graphlet *G*_1_ were formulated as three different ordered graphlets: *O*_1_, *O*_2_, and *O*_3_ (Fig. 3). This way, Malod-Dognin and Pržulj^5^ defined all four possible 3-node ordered graphlets for all two possible 3-node “regular” (i.e., original non-ordered) graphlets. We denote the measure consisting of the existing four counts for 3-node ordered graphlets as *OrderedGraphlet-3*, and we denote our normalized counterpart of *OrderedGraphlet-3* as *NormOrderedGraphlet-3* (normalization is done in the same way as explained above). Unlike for regular (non-ordered) graphlets, we *do* consider 3-node-only ordered graphlets within GRAFENE. We do this to compare as fairly as possible our GRAFENE approach with the existing GR-Align approach that can support only 3-node ordered graphlets^5^. To benefit from larger graphlets, we *extend* this idea to include within GRAFENE all 38 possible 4-node ordered graphlets for all six possible 4-node regular graphlets on top of the existing four 3-node ordered graphlets. We denote the resulting measure consisting of 42 ordered graphlet counts for 3-4-node graphlets (Fig. 3) as *OrderedGraphlet-3-4* and its normalized counterpart as *NormOrderedGraphlet-3-4*. Inclusion of ordered graphlets on five nodes would cause the number of graphlets to grow significantly (e.g., graphlet *G*_9_ can be formulated as 60 different ordered graphlets). Since using too many measures often causes overfitting, which can eventually lead to increased error rate^55^, we do not consider 5-node ordered graphlets. Note that ordered graphlet counts do not vary by orders of magnitude in our data as regular graphlet counts do, so we do not take their logarithms.

Even though ordered graphlets capture relative sequence positions of interacting amino acids, they do not capture how far those amino acids are in the sequence. While there have been conflicting findings regarding the effect of long-range interactions on secondary structure prediction accuracy^12^, we hypothesize that amino acids that are close enough in the protein 3D structure but are far away in the protein sequence are more important than amino acids that are close enough in the 3D structure simply because they are also close in the sequence. To evaluate this hypothesis, we propose a novel concept of *“long-range(K)” ordered graphlets*, where the “long-range(*K*)” constraint is introduced so that a given ordered graphlet is identified in the given PSN if and only if: 1) the same ordered graphlet would also be identified in the above described analysis, and 2) every pair of amino acids that are linked by an edge in the graphlet are at least *K* distance apart in the sequence (that is, *K* is the absolute difference between sequence positions of two amino acids of interest). Supplementary Fig. S1 illustrates this concept. Clearly, all graphlets identified under the “long-range(*K*)” ordered graphlet approach will also be identified under the traditional ordered graphlet approach, but the opposite is not necessarily true. As a proof of concept, we apply the concept of “long-range(*K*)” ordered graphlets on the *NormOrderedGraphlet-3-4* (which as we show in Results is the best of all graphlet features) and we denote the new measure as *NormOrderedGraphlet-3-4(K)*. To evaluate the performance of *NormOrderedGraphlet-3-4(K)*, we vary *K* from one to 10 in increments of one and from 10 to 35 in increments of five. Then, for each considered data set, we report results for the value of *K* that results in the best PC accuracy (for details, see Results).

### Existing approaches

We use 15 existing *network*, *3D contact*, and *sequence* approaches in the task of PC (Fig. 1). Due to space constraints, we describe these approaches in Supplementary Section S7.

### Evaluation of PC accuracy

Given a set of objects (proteins or networks) with known labels, the distance between objects of the same label should ideally be small, while the distance between objects of different labels should ideally be large. To evaluate this, we rely on an established unsupervised strategy^32^. By “unsupervised”, we mean that we rely on object labels only in the phase of evaluating a method’s output. That is, we do not use any label information to train the given method or produce its output, as a supervised (classification) approach would do. Specifically, for each considered approach, we first compute the distance between each pair of objects. Then, we sort all object pairs in terms of their increasing distance and consider *k* closest object pairs, where we vary *k* from 0% to 100% in increments of 0.1%. Next, we compute the accuracy in terms of *precision* and *recall*, where precision is the fraction of label-matching object pairs out of the considered object pairs, and recall is the fraction of the considered label-matching object pairs out of all label-matching object pairs. To summarize the precision and recall results over the whole [0–100%] range of *k*, we measure overall accuracy of the given approach by computing AUPR. Alternatively, we compute the accuracy in terms of *sensitivity* and *specificity*, where sensitivity is the fraction of the considered label-matching object pairs out of all label-matching object pairs, and specificity is the fraction of the considered non-label-matching object pairs out of all non-label-matching object pairs. To summarize the sensitivity and specificity results over the whole [0–100%] range of *k*, we measure overall accuracy of the given approach by computing AUROC. Given a data set, we compare different approaches by comparing their AUPR or AUROC values.

## Results

### Comparison of synthetic networks

We first analyze synthetic networks to demonstrate the general applicability of our PCA-driven GRAFENE approach, compared to the 15 existing network-only approaches (i.e., no 3D structural, sequence, or node order information available), some of which have been used in tasks different than our considered task of PC. Because we show that these approaches cannot successfully cope with synthetic networks of different sizes, we develop a normalized version of GRAFENE, as follows.

#### Evaluation of non-normalized network measures

We evaluate non-normalized versions of our GRAFENE approach (Graphlet-3-4 and Graphlet-3-5), existing graphlet approaches (GDDA, RGFD, and GCD), and existing non-graphlet approaches (average degree, average distance, maximum distance, average closeness centrality, average clustering coefficient, intra-hub connectivity, assortativity, and Existing-all), on synthetic network data of the same size (Synthetic-100, Synthetic-500, and Synthetic-1000); see Methods.

For each data set, both non-normalized GRAFENE versions outperform the existing graphlet and non-graphlet approaches, as the former two always achieve 100% accuracy (Fig. 4 and Supplementary Tables S6,S7). Some existing methods also achieve 100% accuracy on some of the data sets, but only one (RGFD) does so on all three data sets. However, RGFD loses its comparable performance in other tests (see below). Note that the graphlet (PCA and non-PCA) approaches outperform all seven existing non-graphlet approaches that have been used for PC (Fig. 4 and Supplementary Tables S6,S7). Even so, combining the seven measures into Existing-all and using Existing-all in our PCA framework improves the accuracy of each individual non-graphlet measure. Although Existing-all is comparable to our GRAFENE approach (Fig. 4 and Supplementary Tables S6,S7), it also loses its comparable performance in other tests (see below).

#### Network size affects comparison via non-normalized measures

To test whether the considered approaches are robust to the sizes of compared networks, we evaluate them on the Synthetic-all set that contains networks of different topologies *and* different sizes. For these evaluations, we observe a decline in accuracy for each approach (Fig. 4 and Supplementary Tables S6,S7).

#### Normalization of graphlet measures improves comparison

Given that the accuracy appears biased by network size, we use the normalized GRAFENE versions (NormGraphlet-3-4 and NormGraphlet-3-5), which we hope will preserve maximum (100%) accuracy for the three equal-size network sets (Synthetic-100, Synthetic-500, and Synthetic-1000) while improving accuracy for Synthetic-all that contains networks of different sizes. Indeed, this is exactly what we observe (Fig. 4 and Supplementary Tables S6,S7). Now our NormGraphlet-3-5 GRAFENE version outperforms each of the three existing general-purpose graphlet (non-PCA) approaches, including RGFD, suggesting that henceforth GRAFENE should be used for general-purpose network comparison. Also, now our NormGraphlet-3-5 GRAFENE version outperforms the non-graphlet Existing-all approach under the same PCA framework.

### Comparison of PSNs

Unlike the previous synthetic data, protein structure networks (PSNs) have 3D structural and sequence-based information. Recall that we use four PSN construction strategies (Section Methods), to evaluate whether the performance of the network-based PC approaches depends on the choice of PSN construction strategy. Below, we show that this is *not* the case, i.e., the method comparison results are robust across the different choices. Yet, to give the best-case advantage to each PC approach, unless otherwise noted, for each PC approach and each PSN set, we report results for the best PSN construction strategy.

With this in mind, first, we evaluate all approaches (i.e., their existing versions that are non-normalized in terms of network size) on the three PSN sets that form the “equal size” PSN set group, each of which contains PSNs of the same size (Section Methods). Second, we test the approaches on all 35 PSN sets that form the “all groups” PSN set group, each of which contains PSNs of different sizes (Section Methods). Third, we test whether graphlet normalization improves PC on the different-size PSN sets. Fourth, to investigate whether the integration of network topology with protein sequence (i.e., residue order) data can improve PC, we test the ordered graphlet version of our GRAFENE approach, including the effect of the “long-range(*K*)” constraint. Here, we compare in terms of accuracy the best of our GRAFENE versions (with normalized ordered graphlets and the “long-range(K)” constraint – NormOrderedGraphlet-3-4(K)) against the existing network, 3D contact, and sequence approaches. Fifth, in order to see whether the results vary across the four different levels of the CATH or SCOP hierarchies, we break down the above analyses (that are for the “all groups” PSN set group) into per-level analyses, i.e., into analyses for each of PSN set groups 1–4 (Fig. 2 and Section Methods). All of the analyses up to this point are for the best of the four PSN construction strategies. So, sixth, we evaluate whether the results vary across the four different PSN construction strategies. Seventh, we compare the considered PC approaches in terms of their running times (rather than accuracy, as up to this point). Eighth, we summarize our key findings. The eight items are discussed in the following eight subsections.

#### Evaluation of non-normalized measures

Here, we benchmark the non-normalized versions of our PCA-driven GRAFENE approach, existing graphlet (non-PCA) network approaches, existing non-graphlet network approaches, existing 3D contact approaches, and existing sequence approach on all PSN sets for which the networks within the given set are of same size, i.e., CATH-95, CATH-99, and CATH-251-265. For each PSN set, just as for the synthetic networks, the non-normalized versions of GRAFENE (Graphlet-3-4 and Graphlet-3-5) are superior to all existing approaches (Fig. 5 and Supplementary Tables S8,S9). Again, combining the seven existing non-graphlet measures into Existing-all typically improves the accuracy of each individual measure (Fig. 5 and Supplementary Tables S8,S9). Existing-all is superior to the non-normalized GRAFENE versions only on one of the three “equal size” PSN sets.

#### Network size affects comparison via non-normalized measures

**Figure 6:**
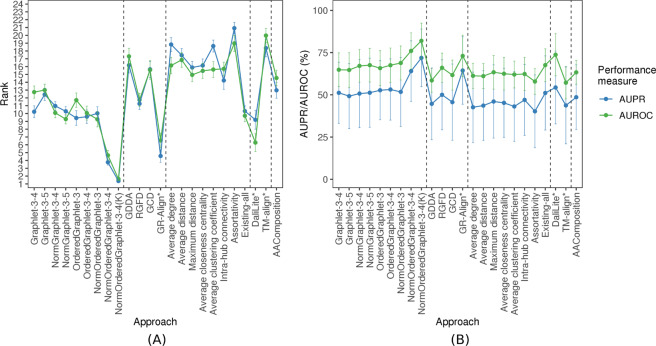
The performance comparison of the 24 considered approaches, averaged over all 35 considered real-world PSN sets of different PSN sizes (that form the “all groups” PSN set group). The figure can be interpreted in the same way as Fig. 5.

Next, we evaluate the same non-normalized PC approaches on all 35 real-world PSN sets of different network sizes (Section Methods). As with the synthetic data, we observe a decline in accuracy for most of the PC approaches (Fig. 6) compared to their performance on the “equal size” PSN sets (Fig. 5 and Supplementary Tables S11 and S12). Even so, the non-normalized GRAFENE versions remain superior or comparable to all existing methods except GR-Align, Existing-all, and DaliLite (Fig. 6). However, as we show below, these three existing approaches lose their superiority in other tests.

#### Normalization of graphlet measures improves comparison

Motivated by the above results, we next evaluate the normalized versions of GRAFENE. As with the synthetic network comparisons, we hope to ideally improve or at least preserve the accuracy for the “equal size” PSN sets (CATH-95, CATH-99, and CATH-251-265) while improving the accuracy for the 35 PSN sets of different sizes, compared to the accuracy of the non-normalized counterparts. Indeed, this is exactly what we observe (Figs 5, 6(B), and Supplementary Tables S11 and S12).

#### Integration of network and sequence (i.e., residue order) data via ordered graphlets

Thus far, we considered GRAFENE versions that are based on regular (non-ordered, as considered thus far) graphlets. These versions already perform better than the considered existing AAComposition sequence approach (Fig. 6 and Supplementary Tables S10–S12). Integration of network data with sequence data may further improve the performance. To test this, we rely on ordered graphlets. GR-Align already used 3-node-only ordered graphlets to impose the sequence-based order of amino acid residues onto nodes in regular graphlets. We adopt this existing idea but: 1) we do so within our alignment-free GRAFENE framework as opposed to the alignment-based GR-Align approach; 2) we extend this idea into larger, 3-4-node ordered graphlets (Fig. 3); 3) we normalize ordered graphlets; and 4) we add the “long-range(*K*)” constraint. We find that each of these four extensions improves PC performance, as follows.

First, when we consider 3-node-only ordered graphlets, which makes the comparison with GR-Align as fair as possible, GRAFENE (version OrderedGraphlet-3) is superior to GR-Align for the three “equal size” PSN sets (Fig. 5). At the same time, GR-Align is ~25 times slower than OrderedGraphlet-3 (Supplementary Table S10).

Second, when we also consider larger ordered graphlets, this further improves the performance of GRAFENE, i.e., OrderedGraphlet-3-4 is superior to OrderedGraphlet-3 (Figs 5 and 6, and Supplementary Table S10).

Third, considering only non-normalized graphlet measures, the ordered graphlet version of GRAFENE (OrderedGraphlet-3-4) on average improves upon its regular graphlet counterpart (Graphlet-3-4) for the three “equal size” PSN sets as well as for the 35 PSNs of different sizes (Figs 5 and 6). Considering normalized graphlet measures, the normalized ordered graphlet version of GRAFENE (NormOrderedGraphlet-3-4) also improves upon its non-ordered counterpart (NormGraphlet-3-4) (Figs 5 and 6). Hence, using ordered graphlets to integrate network and residue order data is always beneficial in our evaluations.

Fourth, adding the “long-range(*K*)” constraint, i.e., considering the *NormOrderedGraphlet-3-4(K)* version of GRAFENE, further improves accuracy (Figs 5, 6, and Supplementary Tables S11, S12). Recall that in these tests, we vary *K* (see Methods). The best value of *K* is data set-dependent. Of the three same-size PSN sets and 35 different-size PSN sets, increasing *K* to at least two (i.e., considering the “long-range(*K*)” ordered graphlet approach) improves accuracy compared to *K* = 1 (i.e., the traditional ordered graphlet approach) for the majority (30) of the PSN sets (Supplementary Tables S13–S36). In particular, accuracy improves for most of the PSN sets at the lower hierarchy levels of CATH or SCOP (i.e., PSN sets from groups 2–4). For the 30 PSN sets, the best value of *K* ranges from two to 35. Since even as high value of *K* as 35 can yield better accuracy than smaller values of *K*, these results exemplify the importance of long-range interactions in the task of PC. Note that for the $35-30=5$ PSN sets where increasing *K* to at least two does not improve accuracy, i.e., where *K* = 1 is superior, NormOrderedGraphlet-3-4(K) results in the same performance as NormOrderedGraphlet-3-4.

In terms of the comparison of our GRAFENE approach against the existing ones, the best GRAFENE version, i.e., NormOrderedGraphlet-3-4(K), is statistically significantly superior to all considered existing network, 3D contact, and sequence approaches (with paired *t*-test *p*-values between $1.1\times {10}^{-5}$ and $7.98\times {10}^{-14}$ for AUPR and between $2.22\times {10}^{-4}$ and $1.43\times {10}^{-16}$ for AUROC; Supplementary Table S10). The fact that within our PCA-driven framework ordered graphlets beat regular graphlets alone and the considered sequence approach alone confirms that PSN data and sequence (residue order) data are complementary and should thus be integrated. We consider this to be one of our key contributions. Another of our key contributions is that our GRAFENE approach (and especially its larger-size normalized ordered graphlet “long-range(*K*)” version) is superior to traditional 3D contact approaches, even though both approach types (network vs. 3D contact) use 3D structural information. This highlights the usefulness of network analyses of protein structures.

#### Performance comparison of PC approaches is similar across different PSN set groups

**Figure 7:**
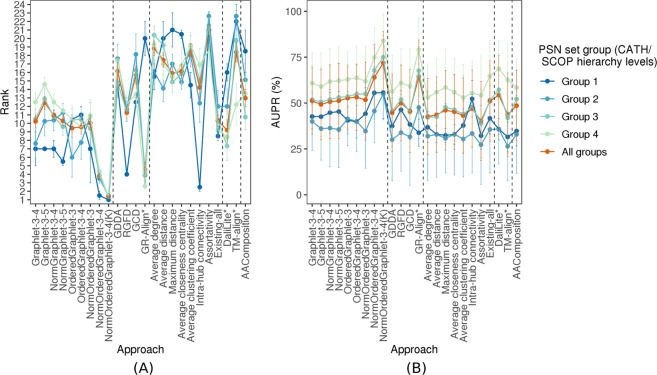
The *PSN set group-specific* performance comparison of the 24 considered approaches, averaged over all PSN sets in the given PSN set group. The figure can be interpreted in the same way as Fig. 5, except that here results are shown only with respect to AUPR but not AUROC. The trends are very similar with respect to AUROC as well (Supplementary Fig. S3). These results are for the best PSN construction strategy. Equivalent results for each of the PSN construction strategies (which are qualitatively similar) are shown in Supplementary Figs S4–S7.

The above analyses have encompassed all 35 PSN sets that form the “all groups” PSN set group. Recall that we divide the 35 PSN sets into groups 1–4, which correspond to the four hierarchy levels of CATH and SCOP (Fig. 2 and Section Methods). Here, we evaluate whether the above key results (e.g., the statistically significant superiority of our best performing GRAFENE version, NormOrderedGraphlet-3-4(K)) vary across the different PSN set groups. We find that this is not the case. That is, NormOrderedGraphlet-3-4(K) still significantly outperforms (with *p*-values <0.05 according to the paired *t*-test) all other approaches for each of the PSN set groups (Fig. 7 and Supplementary Fig. S3).

#### Performance comparison of PC approaches is similar across different PSN construction strategies

Thus far, for each approach and each PSN set, we have selected the best of the four considered PSN construction strategies.

Instead, here, first, for each PC approach, we evaluate whether any one of the four PSN construction strategies is consistently better than the other three over all (or at least the majority) of the PSN sets. We find that this is not the case, i.e., the choice of the best PSN construction strategy is heavily PSN set-dependent (Supplementary Figs S8–S13). The only exceptions are the GDDA, GR-Align, and average closeness centrality approaches, which favor the fourth strategy (*α*-carbon atom type, 7.5 Å distance cut-off). Importantly, for the given PC approach, the performances of the different PSN construction strategies are typically within 5% of each other (Supplementary Figs S8–S13) and can thus be considered as somewhat similar.

**Figure 8:**
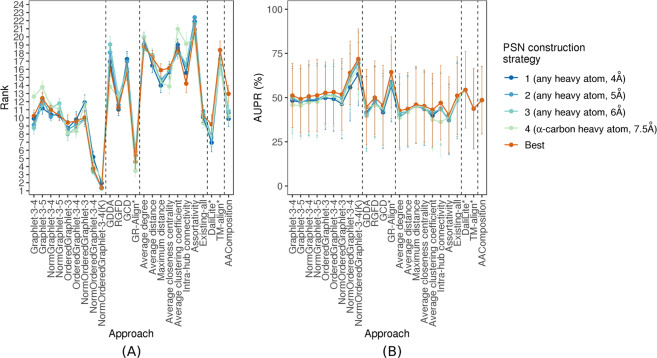
The *PSN construction strategy-specific* performance comparison of the 24 considered PC approaches, with respect to AUPR. The figure can be interpreted in the same way as Fig. 5, except that here results are shown only with respect to AUPR but not AUROC. The trends are very similar with respect to AUROC as well (Supplementary Fig. S14). These results are for the “all groups” PSN set group that spans the 35 PSN sets of different sizes. Equivalent results for each of groups 1–4 (which are qualitatively similar) are shown in Supplementary Figs S15–S18.

Second, we evaluate whether the above results (statistically significant superiority of our best GRAFENE version, namely NormOrderedGraphlet-3-4(K)) vary across the different PSN construction strategies. We find that the choice of PSN construction strategy has no effect on the results. That is, our NormOrderedGraphlet-3-4(K) approach still significantly outperforms (with *p*-values <0.05 according to the paired *t*-test) all other approaches for each of the PSN construction strategies, with only two exceptions, as follows. 1) For the fourth PSN construction strategy, NormOrderedGraphlet-3-4(K)’s performance is comparable to GR-Align’s performance with respect to AUPR (Fig. 8). However, with respect to AUROC, NormOrderedGraphlet-3-4(K) keeps its significantly superior performance over GR-Align (Supplementary Fig. S14). 2) For the first PSN construction strategy, NormOrderedGraphlet-3-4(K)’s performance is only marginally better than DaliLite’s performance with respect to AUROC (Supplementary Fig. S14). However, with respect to AUPR, NormOrderedGraphlet-3-4(K) keeps its significantly superior performance over DaliLite (Fig. 8).

#### Running time comparison

In terms of running time, all alignment-free network approaches (including ours) are comparable to each other (running times between 0.38 and 2.41 hours) as well as to the sequence approach (running time of 0.24 hours), they are followed by the only alignment-based network approach, GR-Align (running time of 9.49 hours), and all of them are significantly faster than the two 3D contact approaches, DaliLite and TM-Align (running times of 2,021 and 168 hours, respectively). See Supplementary Table S10 for more details.

Regarding the running times of our regular (un-ordered) graphlet approach Graphlet-3-4 and its ordered counterpart OrderedGraphlet-3-4, one might intuitively expect the running time should be lower for counting ordered graphlets than for counting regular graphlets, because the order constraint decreases the number of possible graphlets that can be found in a network. Our results reveal that ordered graphlet counting (OrderedGraphlet-3-4) is actually 5.5 times slower than regular graphlet counting (Graphlet-3-4). While this result might be counterintuitive, it is actually expected. This is because each regular graphlet that is found in a network corresponds to some ordered graphlet, and thus, counting ordered graphlets entails: 1) counting regular graphlets and 2) on top of that, determining for each identified (regular) graphlet, the order of its nodes.

#### Summary of results for PSNs

**Figure 9:**
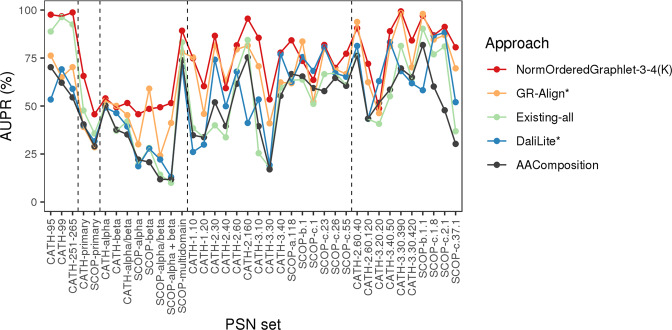
The performance comparison of only the best PC approach in each category (for aesthetics purposes) on all three “equal size” PSN sets and all 35 PSN sets of different size, with respect to raw AUPR values. Namely, results are shown for: the best of our proposed PCA graphlet-based network approaches (GRAFENE version NormOrderedGraphlet-3-4(K)), the best of the existing non-PCA graphlet-based network approaches (GR-Align), the best of the existing non-graphlet network approaches (Existing-all), the best of the existing non-network 3D structural approaches (DaliLite), and the sequence-based approach (AAComposition). The vertical dotted lines separate the PSN sets into the five PSN set groups, namely (from left to right): “equal size”, group 1, group 2, group 3, and group 4. For the equivalent results in terms of raw AUROC values, see Supplementary Fig. S21.

We use graphlet measures within the PCA framework in the task of PC. By normalizing the graphlet measures, we improve upon our non-normalized graphlet measures (Fig. 6 and Supplementary Tables S11 and S12). By imposing sequence order onto nodes via ordered graphlets, we further improve the accuracy. By distinguishing between shorter- and longer-range amino acid interactions via “long-range(*K*)” ordered graphlets, we additionally improve the performance. The best version of our GRAFENE approach, NormOrderedGraphlet-3-4(K), is significantly superior to all other considered approaches in terms of its accuracy, and it is comparable to or faster than the considered approaches in terms of running time (Fig. 9, Supplementary Table S10, and Supplementary Figs S19–S24).

Note that for all network-based PC approaches, we observe better performance (higher AUPR/AUROC values) on the synthetic networks than on the real-world PSNs. One potential explanation of this behavior could be that the network categories are more precisely defined (in the sense that they correlate better with network topology) for the synthetic data than for the real-world PSN data. That is, in the case of the synthetic networks, network categories are defined by well-understood, artificial models, where networks of different categories are defined by rules of clearly distinct network models, while networks of the same category follow the same rules. Consequently, for the synthetic networks, it is highly expected that networks that belong to different categories will be topologically dissimilar, and that networks that belong to the same category will be topologically similar, and that a good network approach will capture well the network (dis)similarities. On the other hand, the PSNs are categorized based on the protein structural domain categories to which they belong, which is a non-artificial and consequently likely (theoretically) imperfect network categorization process, which possibly results in lower AUPR/AUROC values compared to the synthetic networks.

Also, note that we provide in Supplementary Section S8 additional results regarding an occasional difference between the performance of: 1) different PC approaches on same PSN sets (e.g., some approaches showing higher accuracy on SCOP-*α* than on SCOP-*β*, but other approaches showing higher accuracy on SCOP-*β* than on SCOP-*α*), and 2) same PC approaches on different PSN sets (e.g., the given approach’s accuracy being higher for CATH-*α* than for CATH-*β*, but the same approach’s accuracy being lower for SCOP-*α* than for SCOP-*β*). We provide these results and their discussion in Supplementary Section S8 rather than in the main paper due to space constraints.

### Validation of graphlet PCA measures in revealing biochemically interesting PSN patterns

We aim to identify graphlet patterns that lead to successful distinction of different CATH or SCOP label categories from the PSN data, focusing as an illustration on the PSN sets containing networks of the same size (CATH-95, CATH-99, and CATH-251-265) from *α* or *β* protein domain labels. Such graphlets that are significantly (Mann-Whitney *U* test; *p*-value <0.05) represented in *α* but not in *β*, or vice versa, could be linked to the functionality of the given domain label.

**Figure 10:**
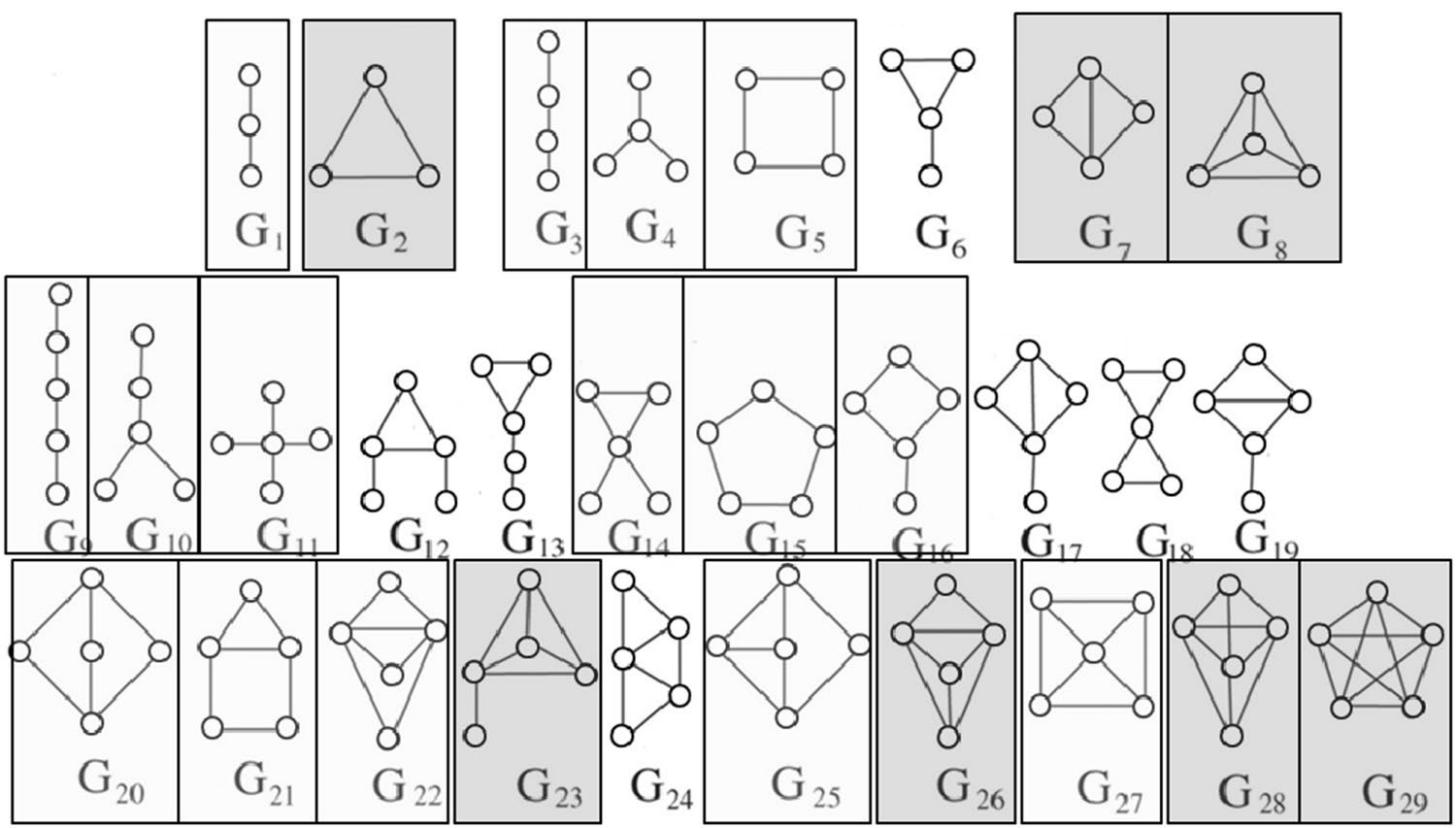
Regular (non-ordered) graphlets that are significantly represented in *α* (dark gray) or *β* (light gray) PSNs. For equivalent results for ordered graphlets, see Supplementary Fig. S25.

For the 3-5-node regular graphlet measure (Graphlet-3-5), graphlets represented in *α* tend to be denser than those represented in *β* (Fig. 10). For example, all of the complete graphlets (i.e., *G*_2_, *G*_8_, *G*_29_, which are the densest graphlets) are represented in *α*, while all of the path-like graphlets (i.e., *G*_1_, *G*_3_, *G*_9_, which are the sparsest graphlets) are represented in *β*.

For the 3-4-node ordered graphlet measure (OrderedGraphlet-3-4), in ordered graphlets represented in *α* (e.g., *O*_1_), there is typically a node order-respecting path through the graphlet, unlike in most of ordered graphlets represented in *β* (e.g., *O*_2_ and *O*_3_) (Supplementary Fig. S25). Note that for the PSN sets from this section (CATH-95, CATH-99, and CATH-251-265), the “long-range(*K*)” constraint does not improve accuracy, and so we do not consider NormOrderedGraphlet-3-4(*K*) here.

The above trends, especially the *α* category being enriched in denser graphlets and the *β* category being enriched in sparser graphlets, are somewhat expected, given the formulations of *α*-helix^56^ and *β*-sheet^57^ 3D configurations. The fact that our graphlet PCA framework uncovers these biochemically relevant PSN patterns in the different protein structural categories further validates the approach. Given this, we hypothesize that our approach can be used to identify biochemically interesting PSN patterns in other applications, such as studying functionally (rather than structurally) different protein categories. Testing this hypothesis is the subject of our future work.

## Conclusions

We present GRAFENE, a general PCA-based computational framework for alignment-free network comparison, which can use any measure(s) of network topology. In the task of general-purpose network comparison, when using graphlets as state-of-the-art measures, GRAFENE outperforms the existing state-of-the-art general-purpose alignment-free network comparison approaches, which are also graphlet-based but not PCA-based. This validates GRAFENE as a new state-of-the-art general-purpose alignment-free network comparison approach. At the same time, in the more specific task of PC, we use ordered graphlets, along with several additional methodological improvements (e.g., normalization or the “long-range(*K*)” constraint), to integrate complementary protein 3D structural data with sequence (i.e., residue order) data. The resulting GRAFENE version significantly outperforms all existing state-of-the-art PC approaches in our evaluations.

## Electronic supplementary material

Supplementary Information
