# Author Correction: GRAFENE: Graphlet-based alignment-free network approach integrates 3D structural and sequence (residue order) data to improve protein structural comparison

**DOI:** 10.1038/s41598-020-69025-8

**Published:** 2020-08-10

**Authors:** Fazle E. Faisal, Khalique Newaz, Julie L. Chaney, Jun Li, Scott J. Emrich, Patricia L. Clark, Tijana Milenković

**Affiliations:** 1 Department of Computer Science and Engineering, University of Notre Dame, Notre Dame, IN 46556 USA; 2 Department of Chemistry and Biochemistry, University of Notre Dame, Notre Dame, IN 46556 USA; 3 Department of Applied and Computational Mathematics and Statistics, University of Notre Dame, Notre Dame, IN 46556 USA; 4 Department of Chemical and Biomolecular Engineering, University of Notre Dame, Notre Dame, IN 46556 USA; 5 Interdisciplinary Center for Network Science and Applications, University of Notre Dame, Notre Dame, IN 46556 USA; 6 Eck Institute for Global Health, University of Notre Dame, Notre Dame, IN 46556 USA

Correction to: *Scientific Reports*10.1038/s41598-017-14411-y, published online 02 November 2017

The original version of this Article contained errors. The authors found a minor bug in the software implementation corresponding to a part of the analysis. After repeating all of the analyses that the software in question was used for, the authors found that the bug had no major effect on the results and it has no effect on the conclusions reported in the paper.

In the Results, under the subheading ‘Integration of network and sequence (i.e., residue order) data via ordered graphlets’,

“Of the three same-size PSN sets and 35 different-size PSN sets, increasing *K* to at least two (i.e., considering the “long-range(*K*)” ordered graphlet approach) improves accuracy compared to *K* = 1 (i.e., the traditional ordered graphlet approach) for the majority (25) of the PSN sets (Supplementary Tables S13–S36). In particular, accuracy improves for most of the PSN sets at the lower hierarchy levels of CATH or SCOP (i.e., PSN sets from groups 2–4). For the 25 PSN sets, the best value of *K* ranges from two to 35. Since even as high value of *K* as 35 can yield better accuracy than smaller values of *K*, these results exemplify the importance of long-range interactions in the task of PC. Note that for the 35 − 25 = 10 PSN sets where increasing *K* to at least two does not improve accuracy, i.e., where *K* = 1 is superior, NormOrderedGraphlet-3-4(K) results in the same performance as NormOrderedGraphlet-3-4.”

now reads:

“Of the three same-size PSN sets and 35 different-size PSN sets, increasing *K* to at least two (i.e., considering the “long-range(*K*)” ordered graphlet approach) improves accuracy compared to *K* = 1 (i.e., the traditional ordered graphlet approach) for the majority (30) of the PSN sets (Supplementary Tables S13–S36). In particular, accuracy improves for most of the PSN sets at the lower hierarchy levels of CATH or SCOP (i.e., PSN sets from groups 2–4). For the 30 PSN sets, the best value of *K* ranges from two to 35. Since even as high value of *K* as 35 can yield better accuracy than smaller values of *K*, these results exemplify the importance of long-range interactions in the task of PC. Note that for the 35 − 30 = 5 PSN sets where increasing *K* to at least two does not improve accuracy, i.e., where *K* = 1 is superior, NormOrderedGraphlet-3-4(K) results in the same performance as NormOrderedGraphlet-3-4.”

“In terms of the comparison of our GRAFENE approach against the existing ones, the best GRAFENE version, i.e., NormOrderedGraphlet-3-4(K), is statistically significantly superior to all considered existing network, 3D contact, and sequence approaches (with paired *t*-test *p*-values between **4.6 × 10**^**−4**^ and **2.46 × 10**^**−13**^ for AUPR and between **2.44 × 10**^**−3**^ and **7.08 × 10**^**−15**^ for AUROC; Supplementary Table S10).”

now reads:

“In terms of the comparison of our GRAFENE approach against the existing ones, the best GRAFENE version, i.e., NormOrderedGraphlet-3-4(K), is statistically significantly superior to all considered existing network, 3D contact, and sequence approaches (with paired *t*-test *p*-values between **1.1 × 10**^**−5**^ and **7.98 × 10**^**−14**^ for AUPR and between **2.22 × 10**^**−4**^ and **1.43 × 10**^**−16**^ for AUROC; Supplementary Table S10).”

In the Results, under the subheading ‘Performance comparison of PC approaches is similar across different PSN set groups’,

“That is, NormOrderedGraphlet-3-4(K) still significantly outperforms (with *p*-values < 0.05 according to the paired *t*-test) all other approaches for each of the PSN set groups, except that for group 4, our approach is comparable to GR-Align (Fig. 7 and Supplementary Fig. S3).”

now reads:

“That is, NormOrderedGraphlet-3-4(K) still significantly outperforms (with *p*-values < 0.05 according to the paired *t*-test) all other approaches for each of the PSN set groups (Fig. 7 and Supplementary Fig. S3).”

Figures 5, 6, 7, 8 and 9 have been corrected in the original Article. In Figures 5, 6, 7 and 8 the data points for the following approaches are corrected: “OrderedGraphlet-3”, “OrderedGraphlet-3-4”, “NormOrderedGraphlet-3”, “NormOrderedGraphlet-3-4”, and “NormOrderedGraphlet-3-4(K)”. In Figure 9 the data points for the following approach are corrected: “NormOrderedGraphlet-3-4(K)”.

The original versions of Figures 5, 6, 7, 8 and 9 appear below as Figures 1, 2, 3, 4 and 5 respectively.

**Figure 1:**
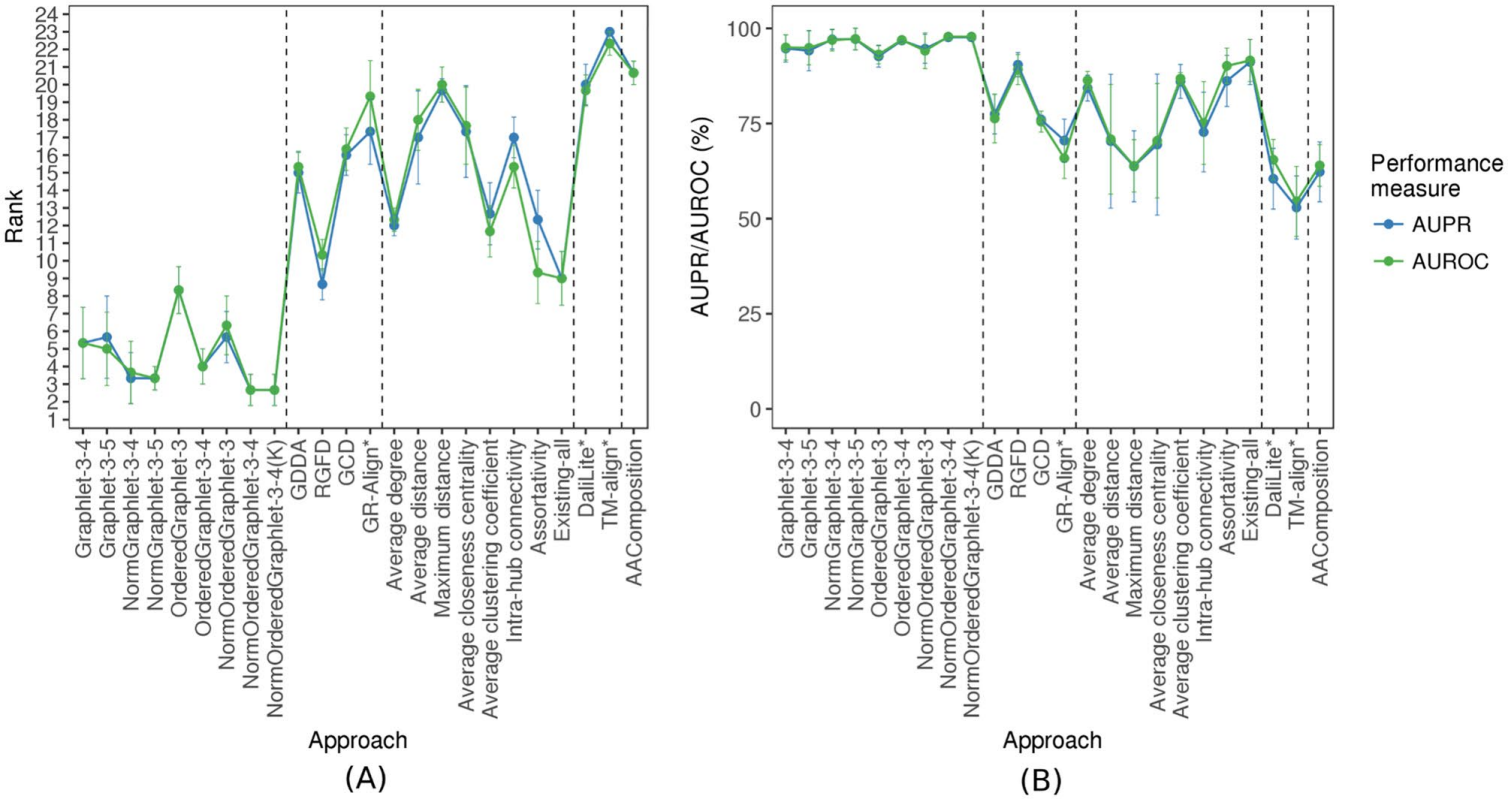
The performance comparison of the 24 considered approaches, averaged over all three considered real-world PSN sets of same network sizes (that form the “equal size” PSN set group), with respect to AUPR/AUROC, in terms of: (**A**) the approaches’ ranks compared to one another, and (**B**) the approaches’ raw AUPR/AUROC values. In panel (**A**), for a given PSN set, the 24 approaches are ranked from the best (rank 1) to the worst (rank 24). Then, for a given approach, its three ranks (corresponding to the three PSN sets) are averaged (the average ranks are denoted by circles, and bars denote the corresponding standard deviations). So, the lower the average rank, the better the approach. In panel (**B**), for each approach, its three raw AUPR/AUROC values (corresponding to the three PSN sets) are averaged (the average values are denoted by circles, and bars denote the corresponding standard deviations). So, the higher the average AUPR/AUROC value, the better the approach.

**Figure 2:**
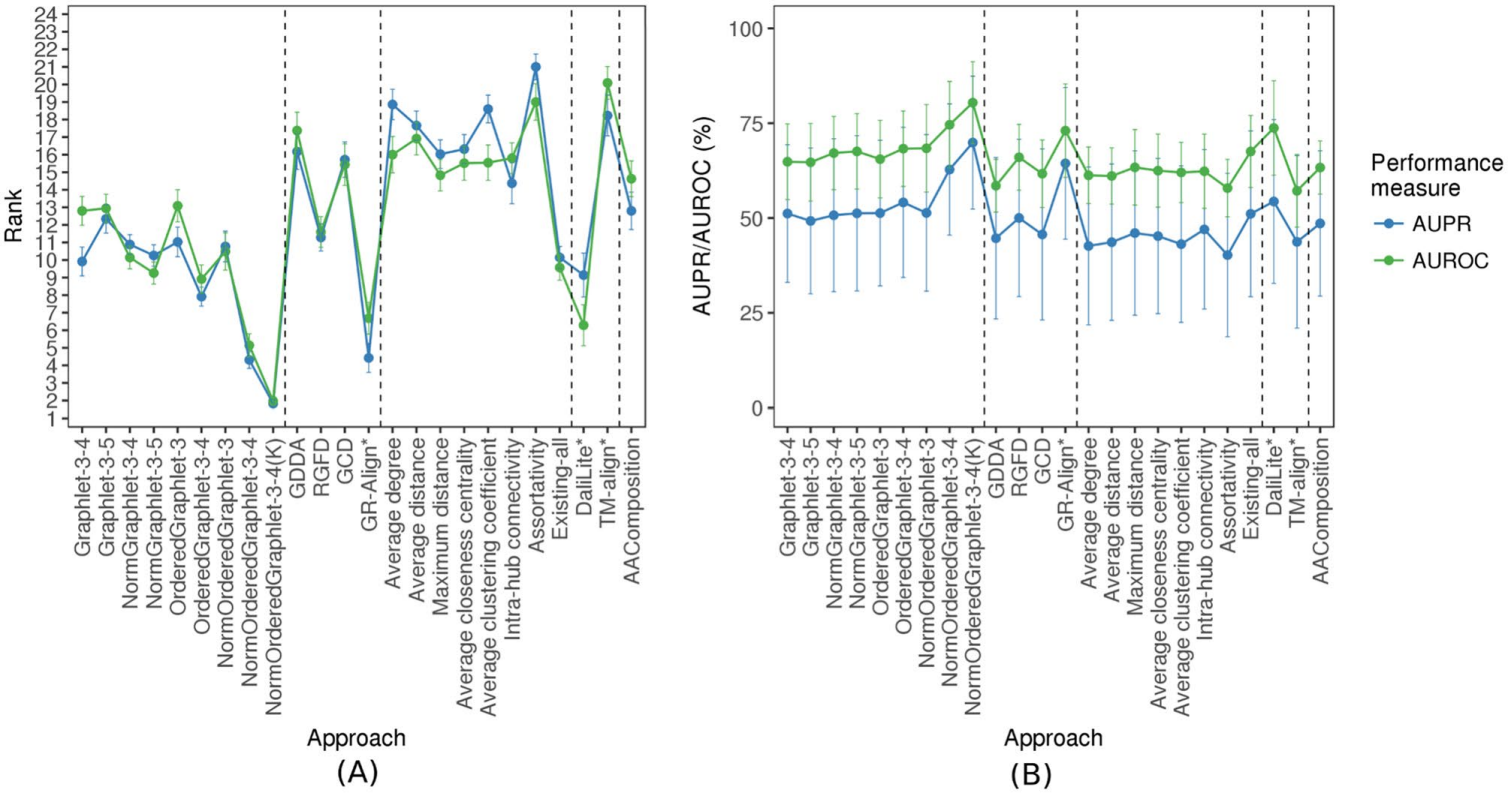
The performance comparison of the 24 considered approaches, averaged over all 35 considered real-world PSN sets of different PSN sizes (that form the “all groups” PSN set group). The figure can be interpreted in the same way as Fig. 5.

**Figure 3:**
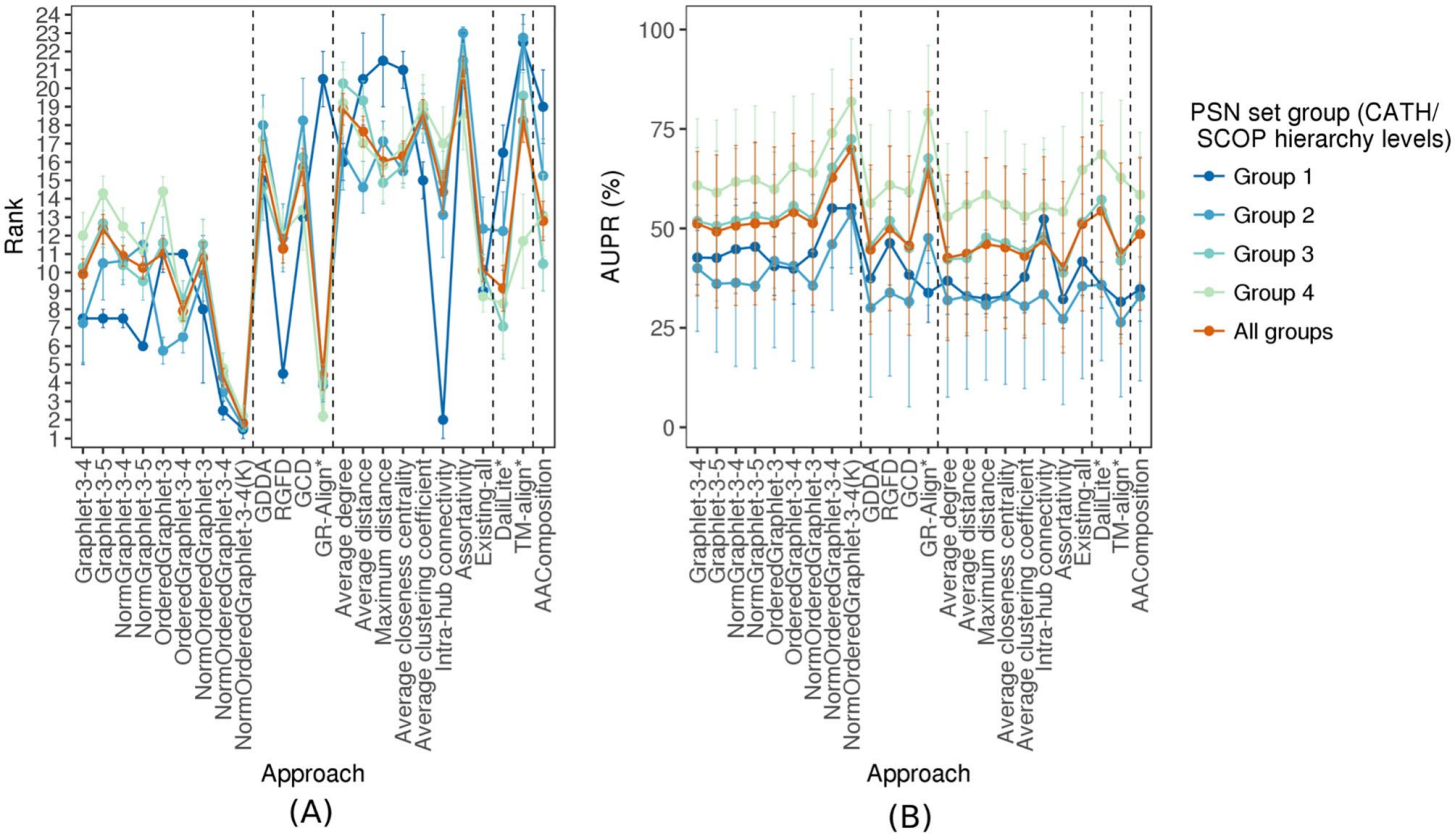
The *PSN set group-specific* performance comparison of the 24 considered approaches, averaged over all PSN sets in the given PSN set group. The figure can be interpreted in the same way as Fig. 5, except that here results are shown only with respect to AUPR but not AUROC. The trends are very similar with respect to AUROC as well (Supplementary Fig. S3). These results are for the best PSN construction strategy. Equivalent results for each of the PSN construction strategies (which are qualitatively similar) are shown in Supplementary Figs S4–S7.

**Figure 4:**
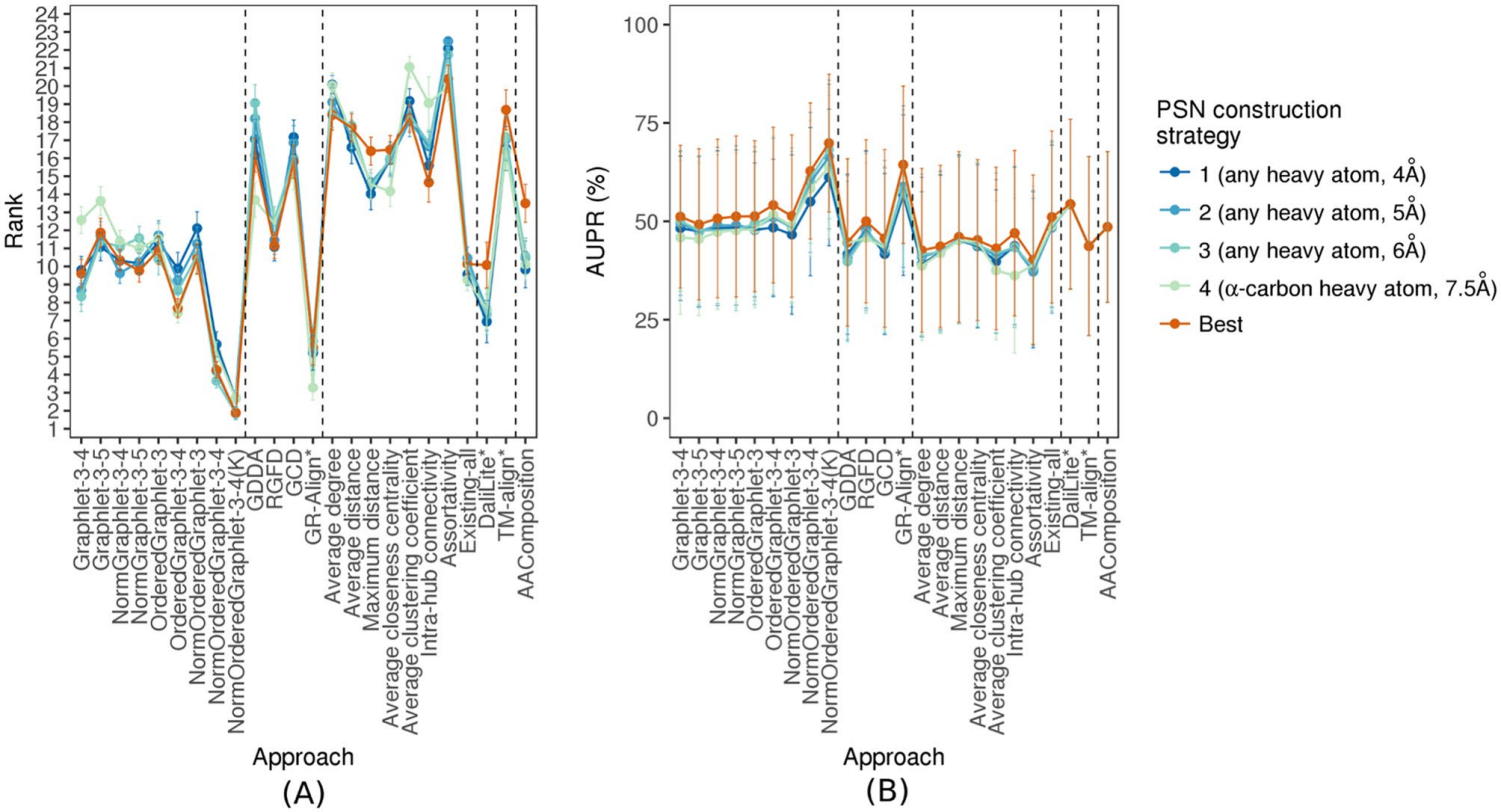
The *PSN construction strategy-specific* performance comparison of the 24 considered PC approaches, with respect to AUPR. The figure can be interpreted in the same way as Fig. 5, except that here results are shown only with respect to AUPR but not AUROC. The trends are very similar with respect to AUROC as well (Supplementary Fig. S14). These results are for the “all groups” PSN set group that spans the 35 PSN sets of different sizes. Equivalent results for each of groups 1–4 (which are qualitatively similar) are shown in Supplementary Figs S15–S18.

**Figure 5:**
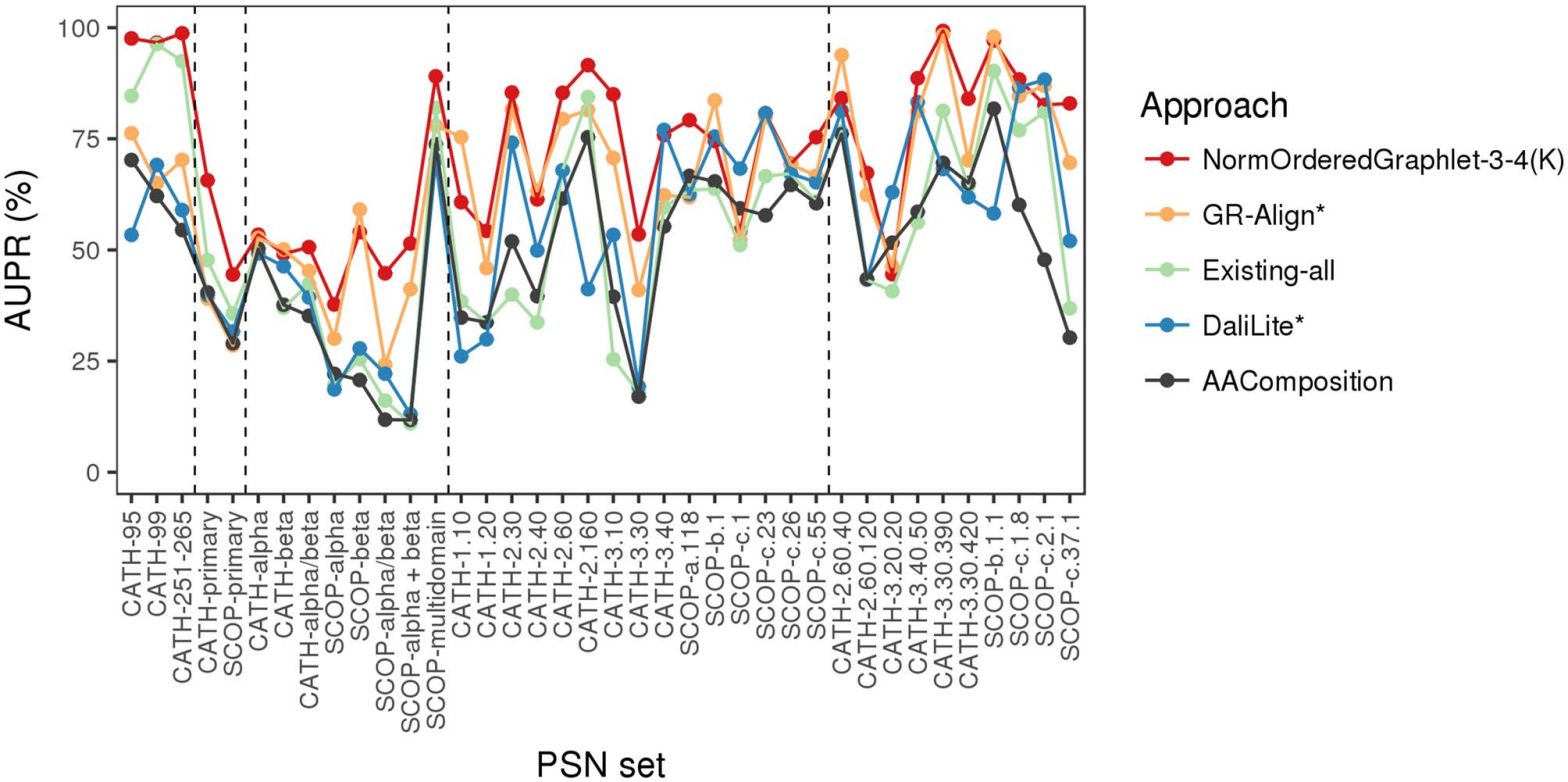
The performance comparison of only the best PC approach in each category (for aesthetics purposes) on all three “equal size” PSN sets and all 35 PSN sets of different size, with respect to raw AUPR values. Namely, results are shown for: the best of our proposed PCA graphlet-based network approaches (GRAFENE version NormOrderedGraphlet-3-4(K)), the best of the existing non-PCA graphlet-based network approaches (GR-Align), the best of the existing non-graphlet network approaches (Existing-all), the best of the existing non-network 3D structural approaches (DaliLite), and the sequence-based approach (AAComposition). The vertical dotted lines separate the PSN sets into the five PSN set groups, namely (from left to right): “equal size”, group 1, group 2, group 3, and group 4. For the equivalent results in terms of raw AUROC values, see Supplementary Fig. S21.

Supplementary Figures and Tables that contain one or more of the following approaches are affected by the changes in these approaches’ accuracy scores: “OrderedGraphlet-3”, “OrderedGraphlet-3-4”, “NormOrderedGraphlet-3”, “NormOrderedGraphlet-3-4”, and “NormOrderedGraphlet-3-4(K)”. As a result, Supplementary Figures S3–S24 and Supplementary Tables S8–S36 have been updated in the Supplementary Information file that now accompanies this Article.

The original Supplementary Information accompanies this correction.

These errors have now been corrected in the PDF and HTML versions of the Article, and in the Supplementary Information that now accompanies the Article.

## Supplementary information

Original Supplementary Information

