# Supplementary material for: GRAFENE: Graphlet-based alignment-free network approach integrates 3D structural and sequence (residue order) data to improve protein structural comparison
Source: Sci Rep. 2017 Nov 2;7:14890. doi: 10.1038/s41598-017-14411-y (PMC5668259; doi:10.1038/s41598-017-14411-y)
Supplement: Supplementary file 1 — Supplementary Information [file 41598_2017_14411_MOESM1_ESM.pdf]

Supplementary Information for: GRAFENE: Graphlet-based alignment-free network approach integrates 3D structural and sequence (residue order) data to improve protein structural comparison

Fazle E. Faisal[1,5,6,†], **Khalique Newaz**[1,5,6,†], **Julie L. Chaney**[2], **Jun Li**[3], **Scott J. Emrich**[1], **Patricia L. Clark**[2,4,6], **and Tijana Milenković**[1,5,6,*]

[1]Department of Computer Science and Engineering, University of Notre Dame, Notre Dame, IN 46556, USA
[2]Department of Chemistry and Biochemistry, University of Notre Dame, Notre Dame, IN 46556, USA
[3]Department of Applied and Computational Mathematics and Statistics, University of Notre Dame, Notre Dame, IN 46556, USA
[4]Department of Chemical and Biomolecular Engineering, University of Notre Dame, Notre Dame, IN 46556, USA
[5]Interdisciplinary Center for Network Science and Applications, University of Notre Dame, Notre Dame, IN 46556, USA
[6]Eck Institute for Global Health, University of Notre Dame, Notre Dame, IN 46556, USA
[†]These authors equally contributed to this work
[*]To whom correspondence should be addressed

I Supplementary Sections

S1 Data

We collect 3D atomic structures of proteins from the Protein Data Bank (PDB)[1]. Since PDB contains multiple copies of the same or nearly identical proteins, we aim to reduce the redundancy by selecting a set of proteins from PDB such that each protein in the set is not more than 90% sequence identical to any other protein in the set. If a protein is not more than 90% sequence identical to any other protein from PDB, we immediately select the protein. If a protein is more than 90% sequence identical to one or more proteins from PDB, we select a "representative" protein from such a protein group so that the representative protein is of the highest quality (in terms of resolution) among all proteins in the group. This strategy results in the selection of 17,036 proteins. We denote this data set as *ProteinPDB*. Each protein in the data is comprised of the X, Y, and Z orthogonal Angstrom (Å) coordinates of heavy atoms (i.e., *carbon*, *nitrogen*, *oxygen*, and *sulfur*) of each amino acid within the protein. The data is available at http://www.rcsb.org/pdb/home/home.do for free download.

Both Class, Architecture, Topology, Homology (CATH) and Structural Classification of Proteins (SCOP) are protein domain categorization databases[2–4]. A protein is typically composed of one or more domains (a domain refers to a part of a protein structure that can fold and often function independently). The purpose of CATH and SCOP is to annotate these domains. We use the protein domain categorization schemes of CATH and SCOP to assign labels to the protein domains from ProteinPDB.

S2 Synthetic networks

We generate synthetic networks by using different network models. A good approach should identify networks from the same network model (i.e., with the same label) as similar, and it should identify networks from different models (i.e., with different labels) as dissimilar. Specifically, we use three well-established network models: *Erdős-Rényi random graphs (ER)*, *geometric random graphs (GEO)*, and *scale-free random graphs (SF)*[5,6]. We note that these models are not necessarily representative of PSNs. Instead, they are general-purpose models. This is intentional, because the models that we use are intended to illustrate wide applicability of our GRAFENE approach to any domain where data can be modeled as networks. It is our analyses of real-world PSNs that focus specifically on the task of PC.

First, we evaluate the considered approaches on synthetic networks of the same size but of different labels (originating from the three network models). To evaluate the robustness of GRAFENE to the choice of network size, we repeat this analysis three times, by increasing the size of the considered networks. That is, we perform three separate analyses of three different network data sets, where in a given data set, all networks are of the same size, and one third of the networks in the set comes from each of the three network models. We denote these network sets as *Synthetic-100*, *Synthetic-500*, and *Synthetic-1000* (Supplementary Table S1), where each set consists of 50 networks per model (totaling to $50 \times 3 = 150$ networks). The numbers of nodes and edges in these networks are set to mimic sizes of real-world PSNs.

Second, we evaluate the considered approaches on networks of different sizes as well as different labels, to check whether the approaches can correctly identify as similar networks from the same model despite the networks being of different sizes, as well as that they can correctly identify as dissimilar networks from different models despite the networks being of the same size. To generate a synthetic network set of different sizes, we combine networks from Synthetic-100, Synthetic-500, and Synthetic-1000 together. We denote the combined network set as *Synthetic-all* (Supplementary Table S1).

S3 Forming real-world PSNs

Here, we continue our discussion regarding the fourth PSN construction strategy that uses the α-carbon atom type and the 7.5 Å distance cut-off. Note that the original GR-Align study used a distance cut-off of 12 Å because this study argued that when considering the α-carbon atom type, this cut-off showed better performance compared to all other tested cut-offs (in the 5 Å-20 Å range)[7]. However, we use the 7.5 Å cut-off for the following reasons. First, even at this cut-off, GR-Align is already much slower than our proposed GRAFENE approach (as we show in our evaluation), and increasing the distance cut-off would only result in more edges and thus further slow down GR-Align. And it was the original GR-Align study that recommended using the 7.5 Å cut-off when aiming to achieve speed-up (as reflected by linear time complexity at this cut-off). Second, as demonstrated in the GR-Align study, for two out of three evaluated performance measures, the improvement when using the 12 Å cut-off compared to when using the 7.5 Å cut-off is negligible and thus not worth the extra increase in computational time that would result from using the 12 Å cut-off compared to using the 7.5 Å cut-off.

S4 Real-world PSNs with CATH categorization

ProteinPDB contains 17,884 protein domains that have CATH categorization, which for a given PSN construction strategy results in 17,884 PSNs. Of these PSNs, to ensure that PSNs are of reasonable "confidence", we focus for further analyses on those PSNs that meet all of the following criteria: 1) the given network has more than 100 nodes, 2) the maximum diameter of the network is more than five, and 3) the network is composed of a single connected component. For different PSN construction strategies, the above criteria can result in different numbers of PSNs. For the first PSN construction strategy (any heavy atom type, 4 Å distance cut-off), this results in 9,509 such PSNs. In the main paper (also, see Supplementary Table S2), we report the number of PSNs with respect to this PSN construction strategy. The number of PSNs resulting from using one of the other three PSN construction strategies is of the similar order.

First, we test how well the considered PC approaches can compare PSNs between the top hierarchical categories (i.e., labels) of CATH: *alpha* (α), *beta* (β), *alpha/beta* (α/β), and *few secondary structures*. Only for few secondary structures, none of the domains in ProteinPDB belong to this category, and so we remove the few secondary structures category from further consideration. Of the 9,509 PSNs, 2,628, 3,085, and 3,796 PSNs belong to (i.e., are labeled with) α, β, and α/β categories, respectively. We denote this PSN set as *CATH-primary* (Fig. 2 in the main paper). The set contains a large enough number of PSNs in each category, which ensures enough statistical power for further analyses.

Second, we test how well the PC approaches can compare PSNs between the second-level hierarchical categories of CATH. That is, within each of the top-level categories of CATH, we compare PSNs belonging to their sub-categories, i.e., second-level categories of CATH. To ensure enough statistical power for further analyses, we focus only on those top-level categories that have at least two sub-categories with at least 30 PSNs each. Each of the three top-level CATH categories satisfies this, and hence, for each of them, we analyze all of their sub-categories that each contain at least 30 PSNs. This results in three PSN sets, denoted as α, β, and α/β (Fig. 2 in the main paper).

Third, we test how well the PC approaches can compare PSNs between the third-level hierarchical categories of CATH. That is, within each of the second-level categories of CATH, we compare the PSNs belonging to their sub-categories, i.e., third-level categories of CATH. To ensure enough statistical power for further analyses, we focus only on those second-level categories that have at least two sub-categories with at least 30 PSNs each. This results in nine PSN sets, denoted as 1.10, 1.20, 2.30, 2.40, 2.60, 2.160, 3.10, 3.30, and 3.40 (Fig. 2 in the main paper).

Fourth, we test how well the PC approaches can compare PSNs between the fourth-level hierarchical categories of CATH. That is, within each of the third-level categories of CATH, we compare PSNs belonging to their sub-categories, i.e., fourth-level categories of CATH. To ensure enough statistical power for further analyses, we focus only on those third-level categories that

have at least two sub-categories with at least 30 PSNs each. This results in six PSN sets, denoted as 2.60.40, 2.60.120, 3.20.20, 3.30.390, 3.30.420, and 3.40.50 (Fig. 2 in the main paper).

Thus, in total, we analyze $1+3+9+6=19$ CATH PSN sets (Fig. 2 in the main paper and Supplementary Tables S3-S5).

S5 Real-world PSNs with SCOP categorization

ProteinPDB has 15,762 protein domains with SCOP categorization, which results in 15,762 PSNs. Of these PSNs, to ensure that PSNs are of reasonable "confidence", we focus on those PSNs that meet the same three criteria that PSNs with CATH categorization are also required to meet, resulting in 11,451 PSNs with SCOP categorization (again, for the first of the four PSN construction strategies). For details, see Supplementary Table S2.

Again, first, we evaluate how well the considered PC approaches can compare PSNs between the top hierarchical categories of SCOP: α, β, α/β, *alpha plus beta* ($\alpha+\beta$), *coiled coil*, *membrane*, *multi-domain*, *small*, *low resolution*, *peptide*, and *designed*. For small, low resolution, peptide, or designed, none of the domains in ProteinPDB belong to these categories, and so we remove these four categories from further consideration. Of the 11,451 PSNs, 1,678, 2,541, 3,835, 2,879, 44, 156, and 318 PSNs belong to α, β, α/β, $\alpha+\beta$, coiled coil, membrane, and multi-domain categories, respectively. This PSN set, denoted as *SCOP-primary* (Fig. 2 in the main paper), contains enough PSNs in each category to ensure enough statistical power for further analyses. Second, we test how well the PC approaches can compare PSNs between the second-level hierarchical categories of SCOP. This results in five PSN sets, denoted as α, β, α/β, $\alpha+\beta$, and multi-domain (Fig. 2 in the main paper). Third, we test how well the PC approaches can compare PSNs between the third-level hierarchical categories of SCOP. This results in six PSN sets, denoted as *a*.118, *b*.1, *c*.1, *c*.23, *c*.26, and *c*.55 (Fig. 2 in the main paper). Fourth, we test how well the PC approaches can compare PSNs between the fourth-level hierarchical categories of SCOP. This results in four PSN sets, denoted as *b*.1.1, *c*.1.8, *c*.2.1, and *c*.37.1 (Fig. 2 in the main paper).

Thus, in total, we analyze $1+5+6+4=16$ SCOP PSN sets (Fig. 2 in the main paper and Supplementary Tables S3-S5).

S6 Real-world PSNs of the same size

To benchmark PSN-based approaches for protein comparison in a way that the comparison cannot be biased by PSN size, we need PSN data of the same (or at least similar) network size (analogous to the synthetic network data sets). For this analysis, we focus on PSNs of α and β labels from the CATH-primary data set. First, within this data set, we aim to identify PSNs that are of reasonable size, i.e., that have $\sim$100 nodes. We further filter the resulting PSN set according to the following rules: 1) the number of nodes in all α and β PSNs is the same, 2) the number of edges in all α and β PSNs is statistically significantly similar (Mann-Whitney U test; p-value < 0.05), and 3) there are at least six PSNs in each of the two label categories. We end up with two such PSN sets. The first set is comprised of 24 PSNs having 95 nodes and 343-362 edges, where 12 PSNs are from α and 12 PSNs are from β. We denote this PSN set as *CATH-95*. The second set is comprised of 28 PSNs having 99 nodes and 347-374 edges, where 12 PSNs are from α and 16 PSNs are from β. We denote this PSN set as *CATH-99*. Second, within the CATH-primary data set, we aim to identify even larger PSNs, i.e., PSNs that have $\sim$250 nodes. We again further filter the resulting PSN set according to the same three rules as above, except that in rule 1, we do not force the number of nodes of all PSNs to match (as we could not identify multiple PSNs that satisfy this constraint) but instead it is sufficient that the PSNs are of statistically significantly similar size in terms of the number of nodes (Mann-Whitney U test; p-value < 0.05). This results in another PSN set, which is comprised of 16 PSNs having 251-265 nodes and 1,003-1,076 edges, where nine PSNs are from α and seven PSNs are from β. We denote this PSN set as *CATH-251-265*. Note that the reported numbers of PSNs in these three "equal size" PSN sets are with respect to the first PSN construction strategy (any heavy atom type, 4 Å distance cut-off). Yet, the numbers remain the same for the other three PSN construction strategies.

S7 Existing approaches

S7.1 Existing network approaches

Existing approaches of this type that we use for PC (not all of which were proposed for PC but can be adapted to it) can be categorized into graphlet and non-graphlet approaches. None of them use PCA as we do.

Existing graphlet approaches. These include graphlet degree distribution agreement (GDDA)[8], relative graphlet frequency distance (RGFD)[9], graphlet correlation distance (GCD)[10], and GR-Align[7]. Among them, GDDA, RGFD, and GCD can compare any type of networks, while GR-Align has been specifically designed to compare PSNs. GDDA, RGFD, and GCD are alignment-free, while GR-Align is alignment-based. For each network pair, each of the four existing graphlet-based network approaches outputs a similarity (or equivalently, a distance) score. Then, for each approach, we sort all network pairs in terms of their increasing distance and evaluate the given approach the given approach as discussed in Section "Evaluation of PC accuracy" of the main manuscript.

Two alternative graphlet approaches were used in the context of PSNs[11,12], but they were used to predict (classify in a supervised manner) functional residues in PSNs (where residues are nodes in PSNs) and not for PSN comparison. Since these approaches compare nodes rather than networks, and since they are supervised (while our study is unsupervised, per our discussion in Section "Evaluation of PC accuracy" of the main manuscript), the approaches do not fit the context of our study. As such, we do not consider them further.

Existing non-graphlet approaches. Several PSN measures have already been used for PC: *average degree*, *average distance*, *maximum distance*, *average closeness centrality*, *average clustering coefficient*, *intra-hub connectivity*, and *assortativity*[13–17]. For each measure, for each pair of networks, we compute Euclidean distance between the networks' vectors (because all vectors are 1-dimensional, here we cannot use cosine similarity as for our GRAFENE approach). We describe these measures below.

Average degree. The average degree of a network can be interpreted as a measure of the overall connectivity of the network. The degree of a node is the number of its network neighbors. The average degree of a network is the average of degrees of all nodes in the network. This measure has been used for analyzing protein structures by[13–17].

Average distance. The distance between two nodes in a network is the length of the shortest path between the nodes. The average distance of a network is the average of distances over all pairs of nodes in the network. This measure has been used for analyzing protein structures by[16,17].

Maximum distance. The maximum distance of a network is the largest of all distances in the network. This measure has been used for analyzing protein structures by[16].

Average closeness centrality. The *closeness centrality* of a node in a network can be interpreted to be the *nearness* of the node to all other nodes in the network. The closeness centrality $cl(v)$ of a node $v \in V$ is computed as $cl(v) = \frac{1}{\sum\limits_{u \in V} d(u,v)}$, where $d(v,u)$ is the distance between nodes v and u. The average closeness centrality of a network is the average of the closeness centrality values of all nodes in the network. This measure has been used for analyzing protein structures by[16,17].

Average clustering coefficient. The *clustering coefficient* of a node in a network can be interpreted as a measure of the connectivity between the neighbors of the node. Given a node v with m neighbors, the clustering coefficient $cc(v)$ of the node v is computed as $cc(v) = \frac{b}{\frac{m(m-1)}{2}}$, where b is the number of edges in the network connecting the m neighbors of v. The average clustering coefficient of a network is the average of clustering coefficient values of all nodes in the network. This measure has been used for analyzing protein structures by[16,17].

Intra-hub connectivity. The intra-hub connectivity of a network can be interpreted as the overall connectivity of the hub nodes within the network.[14] defined a node to be a hub in a PSN if the degree of the node is at least three. We adopt the same strategy to define a hub node in this study. Given k such hub nodes in a network, the intra-hub connectivity of the network is computed as $\frac{m}{\frac{k(k-1)}{2}}$, where m is the number of connections between the hub nodes and $\frac{k(k-1)}{2}$ is the maximum possible number of connections between the hub nodes. This measure has been used for analyzing protein structures by[14].

Assortativity. The assortativity of a network can be interpreted as the tendency of the high degree nodes to be connected with other high degree nodes (see[18] for details). This measure has been used for analyzing protein structures by[16].

We combine the seven measures into an eighth measure, *Existing-all*, to investigate whether the integration of different and complementary topological measures helps PC. We use Existing-all within our PCA framework. This way, we can fairly compare our graphlet measures (i.e., different versions of our GRAFENE approach) and the existing non-graphlet measures within the same framework.

S7.2 Existing 3D contact approaches

These include DaliLite[19] and TM-align[20]. Given two proteins (i.e., 3D co-ordinates of their residues), each of DaliLite and TM-align outputs the proteins' structural similarity score: z-score in the case of DaliLite and TM-score in the case of TM-align. In our evaluation framework, we sort all protein pairs in terms of their increasing distance, i.e., decreasing z-scores for DaliLite and decreasing TM-scores for TM-Align, and then we evaluate DaliLite and TM-Align as discussed in Section "Evaluation of PC accuracy" of the main manuscript.

S7.3 Existing sequence approach

The sequence-based approach that we use, which we call AAComposition, works as follows. For a given protein, for each amino acid type i (out of 20 possible types), we divide the number of amino acids of type i by the total number of amino acids in the protein sequence. We use the resulting 20 values, along with the length of the protein sequence, as the protein's sequence-based measure (i.e., feature vector). Then, we use this measure within our PCA framework. This way, we can fairly compare network- and sequence-based measures within the same framework.

S8 Performance trends of different PC approaches on same PSN sets and of same PC approaches on different PSN sets

Performance trends of different PC approaches on same PSN sets. We sometimes observe a difference in trends between different PC approaches for same PSN sets. Specifically, in the case of the CATH database, all approaches result in a consistent trend that their accuracy for CATH-α is higher than their accuracy for CATH-β. Similarly, in the case of the SCOP database, the majority of the approaches show a consistent trend that their accuracy for SCOP-β is higher than their accuracy for SCOP-α, except the GDDA, GCD, and AAComposition PC approaches, whose accuracy for SCOP-α is higher than their accuracy for SCOP-β. This difference in the trends between the different approaches (GDDA, GCD, and AAComposition versus all others) for SCOP is an approach-specific issue, meaning that some approaches might simply work better for (i.e., better capture patterns in) data of type 1 (e.g., α) than for data of type 2 (e.g., β), while other approaches might show the opposite trend (i.e., work better for data of type 2 than for data of type 1). It is hard to explain why this is, especially for the network-based approaches, because these approaches are heuristics (due to the computational intractability, i.e., NP-hardness, of the network comparison problem) without a theoretic guarantee on their accuracy (and especially on their accuracy on certain data types as opposed to other data types).

Performance trends of same PC approaches on different PSN sets. Additionally, we observe a difference in the performance of same PC approaches on different PSN sets. Specifically, a given approach might have higher accuracy for CATH-α than for CATH-β, but the same approach might have lower accuracy for SCOP-α than for SCOP-β. This trend inconsistency holds for all considered PC approaches except GDDA, GCD, and AAComposition; for both CATH and SCOP, the accuracy of these three approaches is higher for α than for β. This trend inconsistency is likely a data-specific issue: 1) CATH and SCOP do not necessarily contain the exact same PSNs (meaning that some PSNs that are in CATH might be missing from SCOP, and vice versa), and 2) for those PSNs that are in both CATH and SCOP, the PSNs might be categorized into some protein domain group (e.g., α) in CATH but to a different protein domain group (e.g., α/β) in SCOP, because the methodologies that CATH and SCOP use to categorize proteins into domain groups are not identical. If any of these two conditions is met, this could explain the observed trend inconsistency. Indeed, we find that:

1. Of all (α, β, or α/β) PSNs that are in CATH, only 27% are in SCOP. Similarly, of all (α, β, α/β, or α+β) PSNs that are in SCOP, only 24% are in CATH. That is, most of the PSNs are unique to CATH and SCOP.
2. For all PSNs that are present in both CATH and SCOP:
    - 8% of the PSNs that are labeled as α in CATH are labeled as β, α/β, or α+β in SCOP.
    - 0.3% of the PSNs that are labeled as α in SCOP are labeled as β or α/β in CATH.
    - 37% of the PSNs that are labeled as β in CATH are labeled as α, α/β, or α+β in SCOP.
    - 38% of the PSNs that are labeled as β in SCOP are labeled as α or α/β in CATH.
    - 40% of the PSNs that are labeled as α/β in CATH are labeled as α or β in SCOP.
    - 43% of the PSNs that are labeled as α/β or α+β in SCOP are labeled as α or β in CATH.

Clearly, both of the above conditions are met, and hence, the observed trend inconsistency is not surprising.

Note that the above results are with respect to the first PSN construction strategy (any heavy atom, 4 Å) and the performance evaluation using AUPR.

II Supplementary Figures

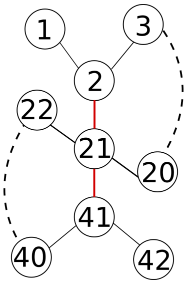

Supplementary Figure S1. Illustration of the importance of "long-range(K)" ordered graphlets. A PSN is shown for a toy protein that consists of 42 amino acids in the sequence, i.e., nodes in the PSN (amino acids 4-19 and 23-39 are not shown for simplicity, as indicated by dashed lines). The nodes are denoted by their amino acid positions (i.e., residue order) in the sequence. Black solid lines are network edges that indicate sequence closeness of the corresponding amino acids (meaning that the amino acids are adjacent in the sequence), which in turn yields sufficient 3D spatial proximity of the amino acids. On the other hand, red solid lines are network edges that indicate only spatial proximity, without sequence adjacentness. On the one hand, both the three-node path 1–2–3 as well as the three-node path 2–21–41 correspond to the same ordered graphlet, namely O_1 from Fig. 3 in the main manuscript, under the traditional ordered graphlet approach. However, we argue that the latter is more interesting than the former, as the former is O_1 simply because of sequence adjacentness of amino acids 1 and 2 as well as 2 and 3, while the latter is O_1 because of spatial proximity of amino acids 2 and 21 as well as 21 and 41. On the other hand, even for K value as low as two, the path 1–2–3 will not be detected as O_1 under the "long-range(K)" ordered graphlet approach, while the path 2–21–41 will, because all of its linked node pairs are at least two amino acids apart in the sequence. Note that the path 2–21–41 will be identified as O_1 up to K value of $min(21-2, 41-21) = 19$).

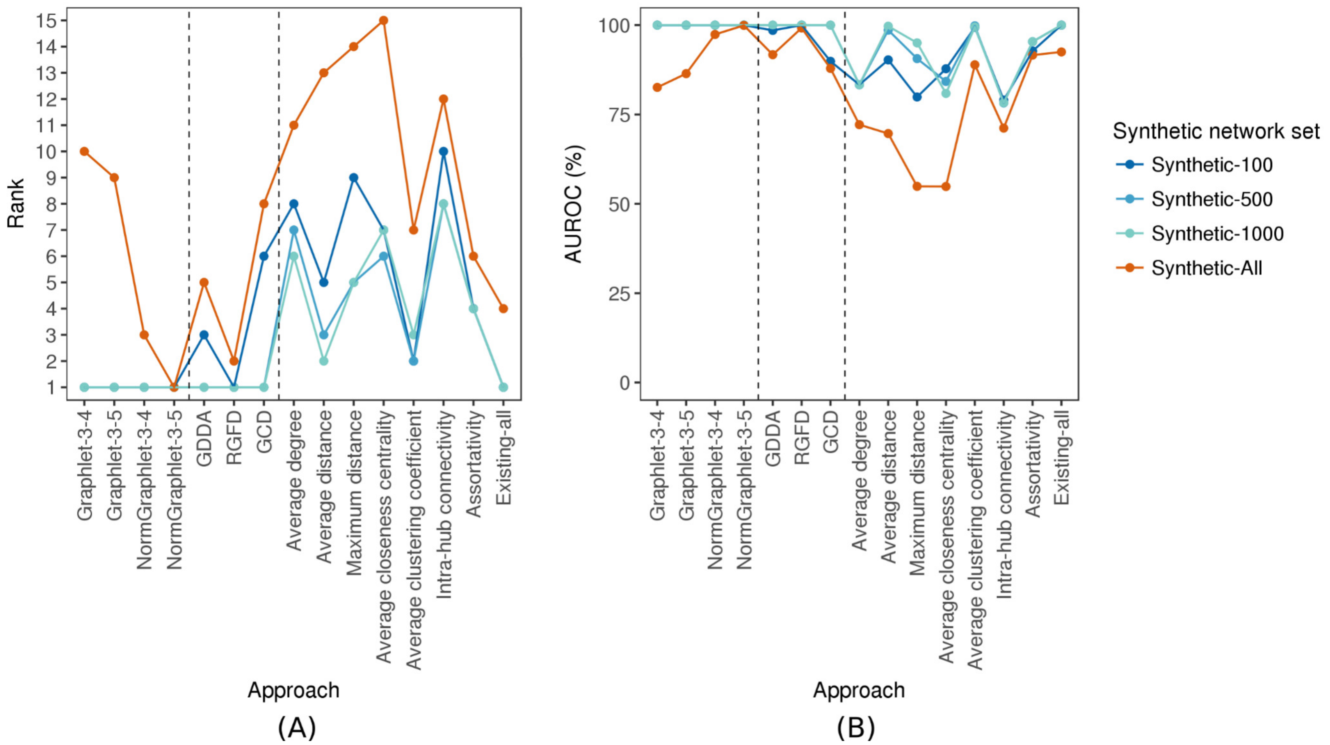

Supplementary Figure S2. The performance comparison of the 15 considered approaches on each of the four considered synthetic network sets, with respect to AUROC, in terms of: (A) the approaches' ranks compared to one another, and (B) the approaches' raw AUROC values. In panel (A), for a given synthetic network set, the 15 approaches are ranked from the best (rank 1) to the worst (rank 15). So, the lower the rank, the better the approach. In panel (B), for each approach, its raw AUPR value is shown for each of the four synthetic network sets. So, the higher the AUROC value, the better the approach. For equivalent results with respect to AUPR values, see Fig. 4 in the main manuscript.

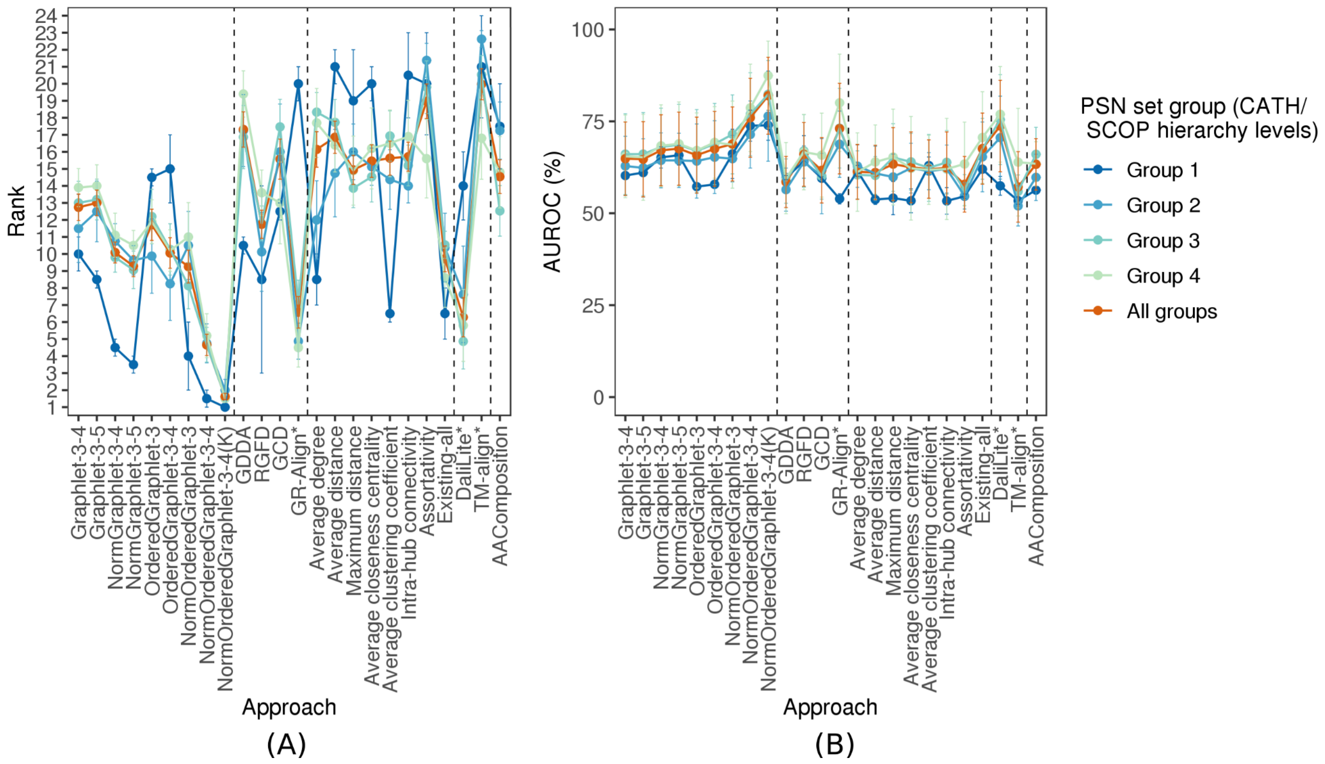

Supplementary Figure S3. The PSN set group-specific performance comparison of the 24 considered approaches, averaged over all PSN sets in the given PSN set group, with respect to AUROC, in terms of: (A) the approaches' ranks compared to one another, and (B) the approaches' raw AUROC values. In panel (A), for a given PSN set, the 24 approaches are ranked from the best (rank 1) to the worst (rank 24). Then, for a given approach, its ranks over all group-specific PSN sets are averaged (the average ranks are denoted by circles, and bars denote the corresponding standard deviations). So, the lower the average rank, the better the approach. In panel (B), for each approach, its group-specific raw AUROC scores are averaged (the average values are denoted by circles, and bars denote the corresponding standard deviations). So, the higher the average AUROC value, the better the approach. The trends are very similar with respect to AUPR as well (Fig. 7 in the main manuscript). These results are for the best PSN construction strategy. Equivalent results for each of the PSN construction strategies are shown in Supplementary Fig. S4-S7.

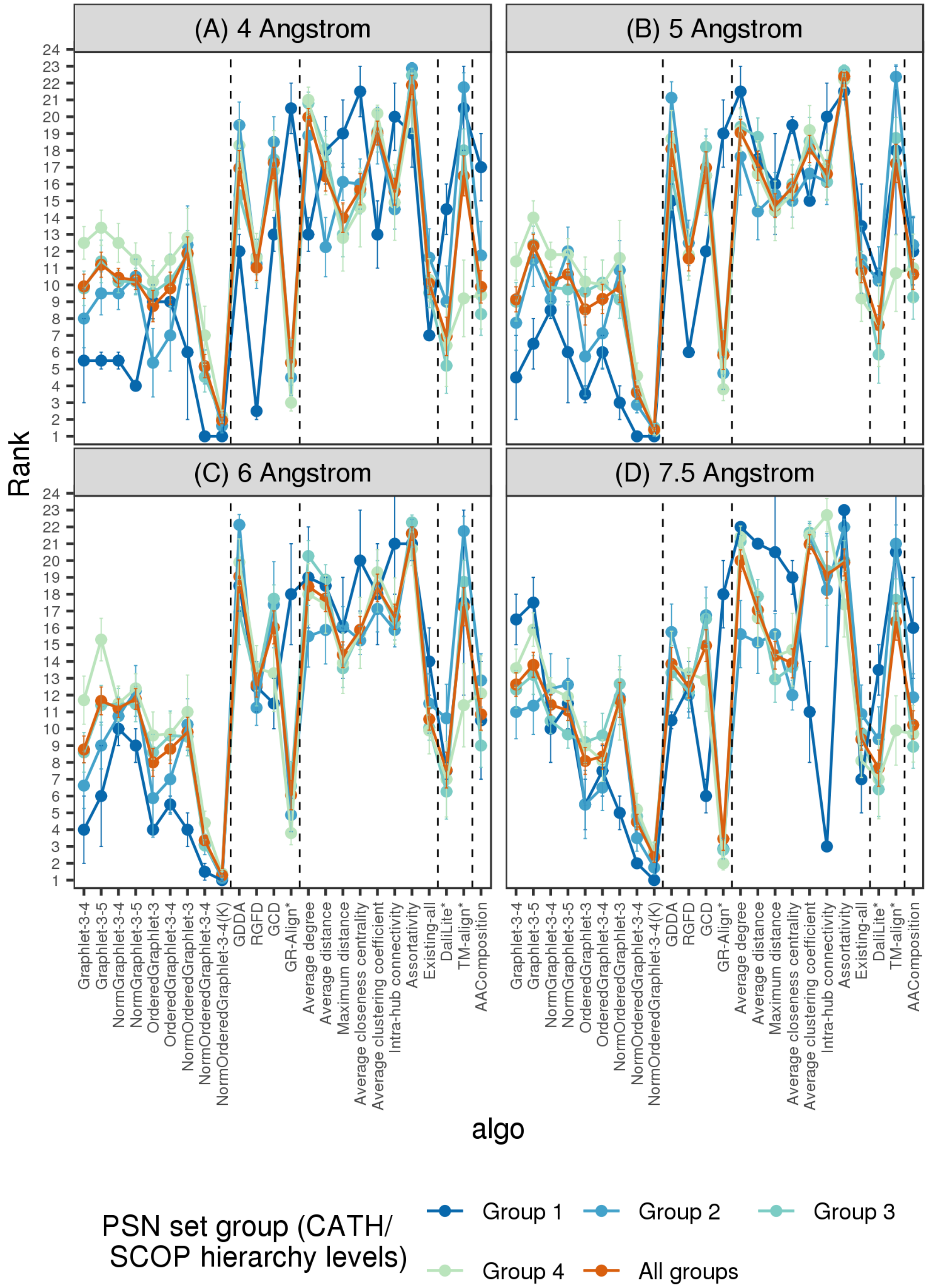

Supplementary Figure S4. The PSN set group-specific rank performance comparison of the 24 considered approaches, averaged over all PSN sets in the given PSN set group, with respect to AUPR, corresponding to the (A) first (any heavy atom, 4 Å), (B) second (any heavy atom, 5 Å), (C) third (any heavy atom, 6 Å), and (D) fourth (α-carbon heavy atom, 7.5 Å) PSN construction strategy. For a given PSN set, the 24 approaches are ranked from the best (rank 1) to the worst (rank 24). Then, for a given approach, its ranks over all group-specific PSN sets are averaged (the average ranks are denoted by circles, and bars denote the corresponding standard deviations). So, the lower the average rank, the better the approach. The trends are very similar with respect to AUROC as well (Supplementary Fig. S5).

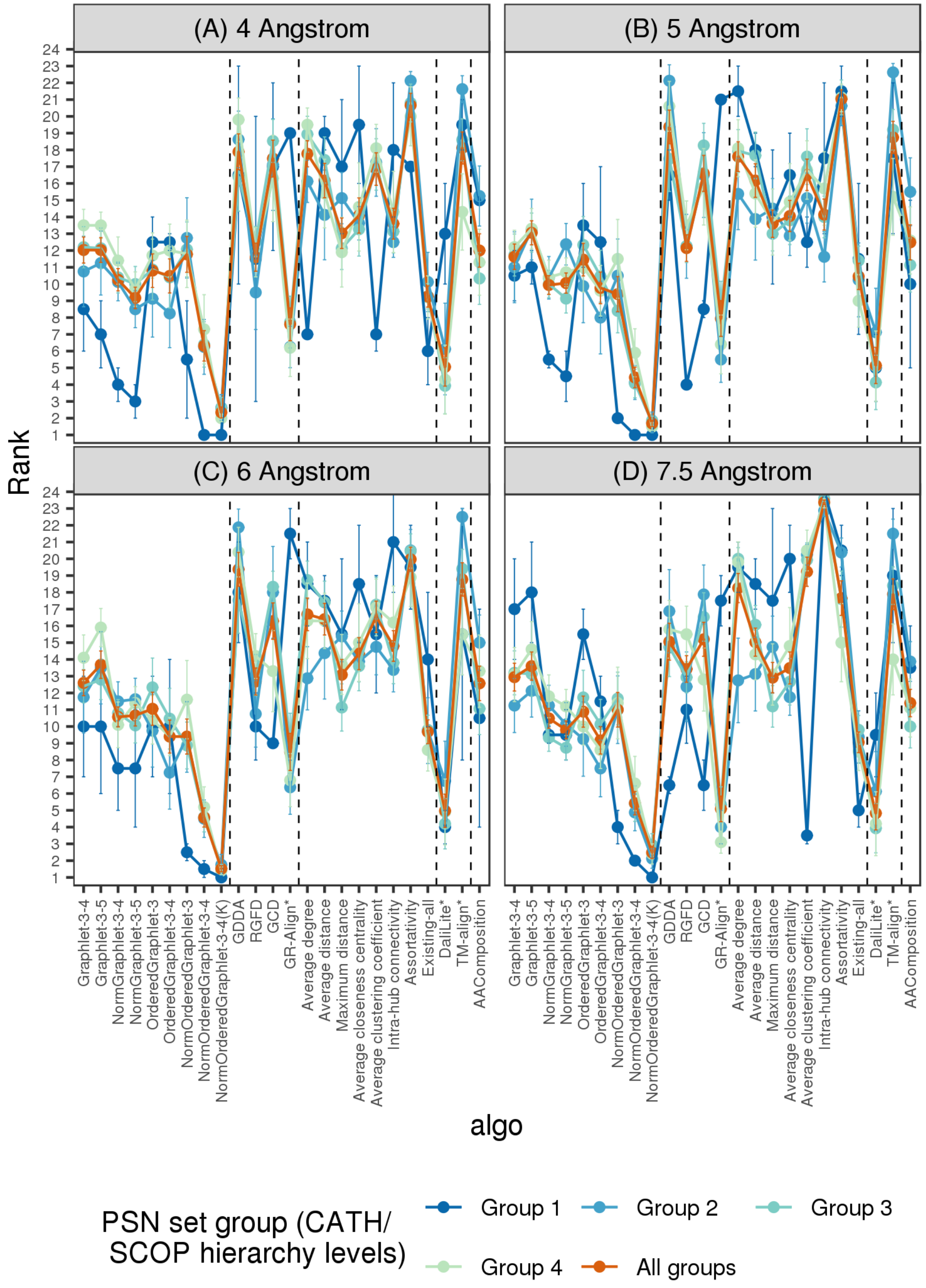

Supplementary Figure S5. The PSN set group-specific rank performance comparison of the 24 considered approaches, averaged over all PSN sets in the given PSN set group, with respect to AUROC, corresponding to the (A) first (any heavy atom, 4 Å), (B) second (any heavy atom, 5 Å), (C) third (any heavy atom, 6 Å), and (D) fourth (α-carbon heavy atom, 7.5 Å) PSN construction strategy. For a given PSN set, the 24 approaches are ranked from the best (rank 1) to the worst (rank 24). Then, for a given approach, its ranks over all group-specific PSN sets are averaged (the average ranks are denoted by circles, and bars denote the corresponding standard deviations). So, the lower the average rank, the better the approach. The trends are very similar with respect to AUPR as well (Supplementary Fig. S4).

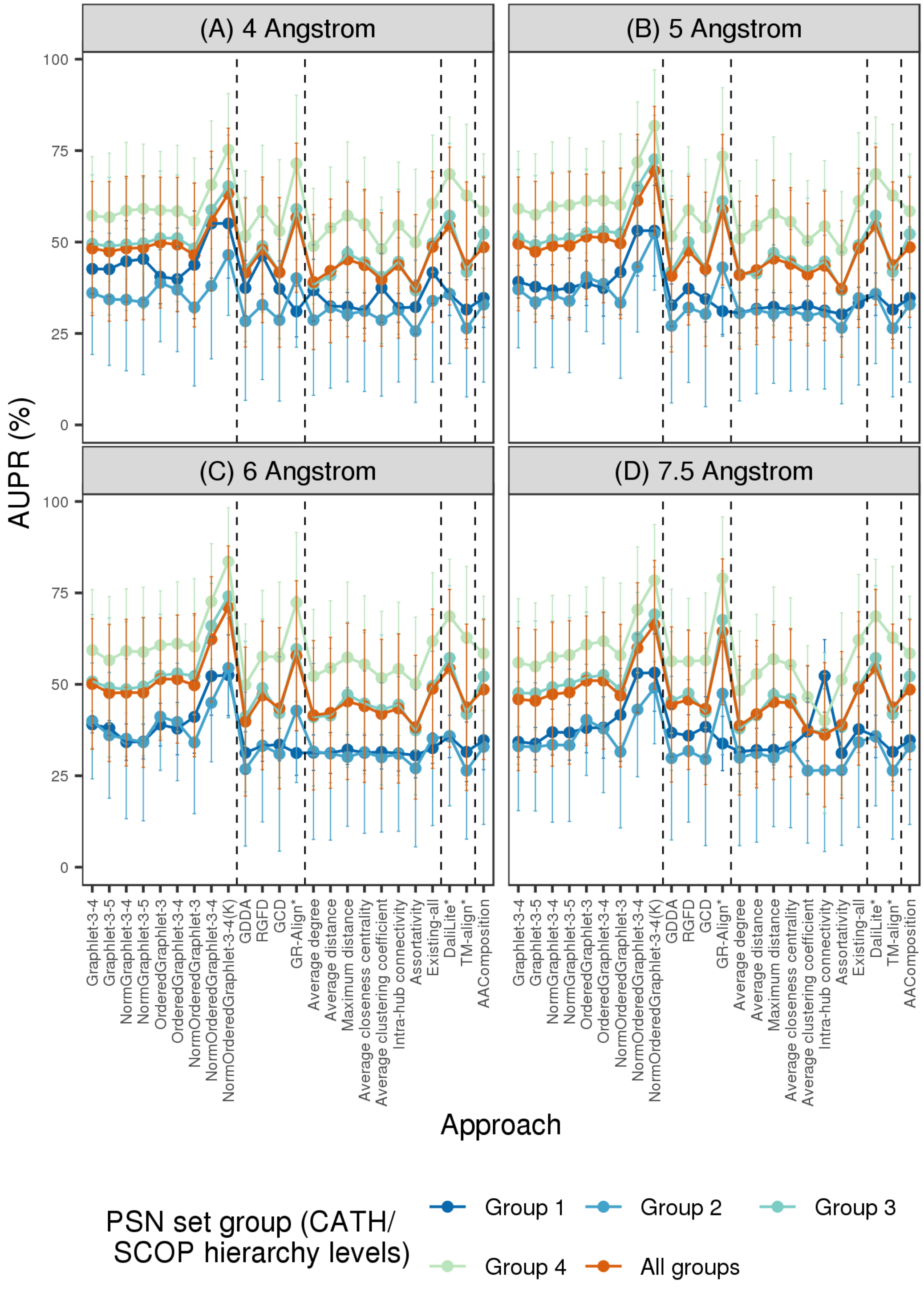

Supplementary Figure S6. The PSN set group-specific performance comparison of the 24 considered approaches, averaged over all PSN sets in the given PSN set group, with respect to AUPR values (expressed as percentages), corresponding to the (A) first (any heavy atom, 4 Å), (B) second (any heavy atom, 5 Å), (C) third (any heavy atom, 6 Å), and (D) fourth (α-carbon heavy atom, 7.5 Å) PSN construction strategy. For each approach, its group-specific raw AUPR values are averaged (the average values are denoted by circles, and bars denote the corresponding standard deviations). So, the higher the average AUPR value, the better the approach. The trends are very similar with respect to AUROC as well (Supplementary Fig. S7).

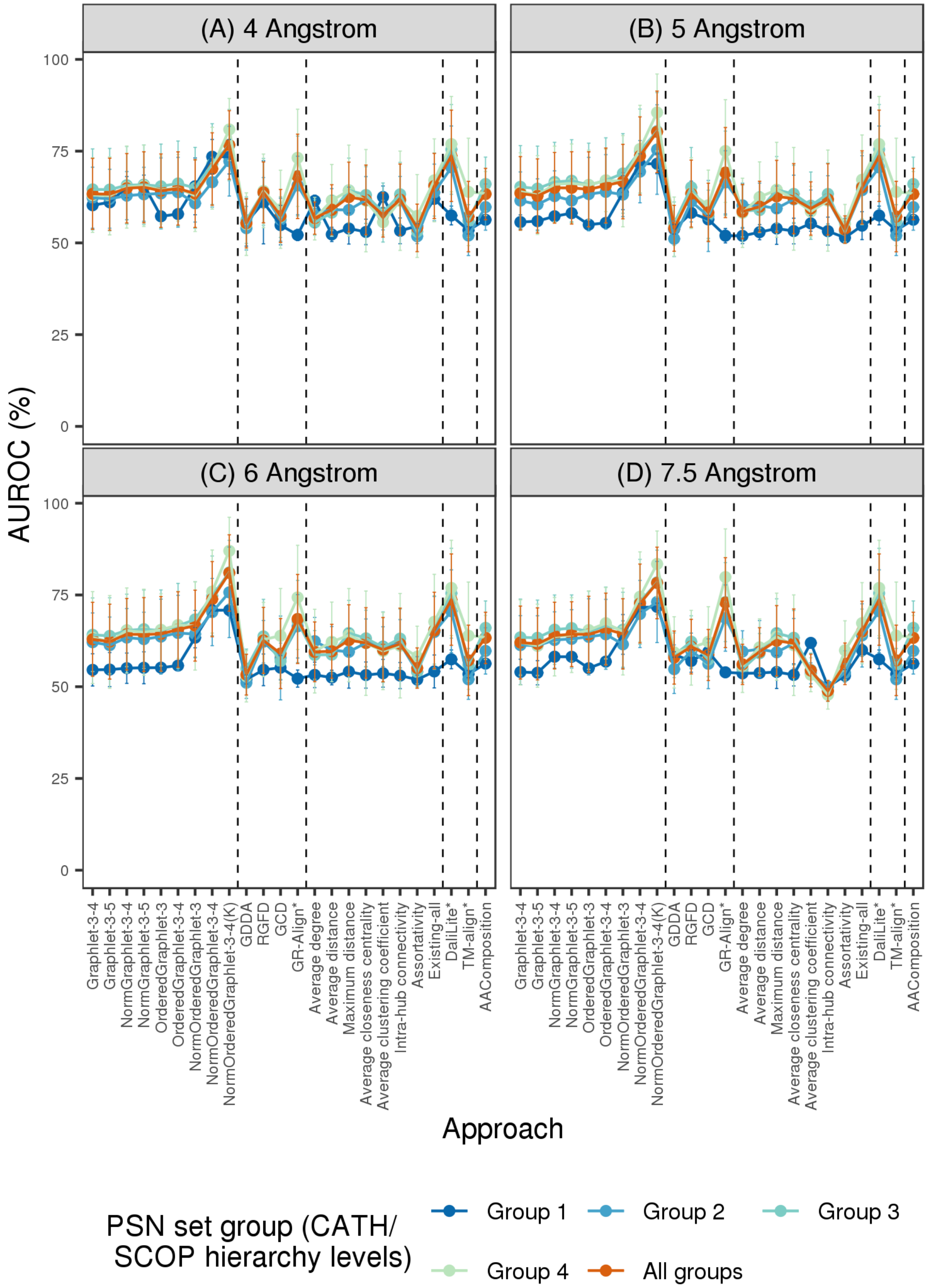

Supplementary Figure S7. The PSN set group-specific performance comparison of the 24 considered approaches, averaged over all PSN sets in the given PSN set group, with respect to AUROC values (expressed as percentages), corresponding to the (A) first (any heavy atom, 4 Å), (B) second (any heavy atom, 5 Å), (C) third (any heavy atom, 6 Å), and (D) fourth (α-carbon heavy atom, 7.5 Å) PSN construction strategy. For each approach, its group-specific raw AUROC values are averaged (the average values are denoted by circles, and bars denote the corresponding standard deviations). So, the higher the average AUROC value, the better the approach. The trends are very similar with respect to AUPR as well (Supplementary Fig. S6).

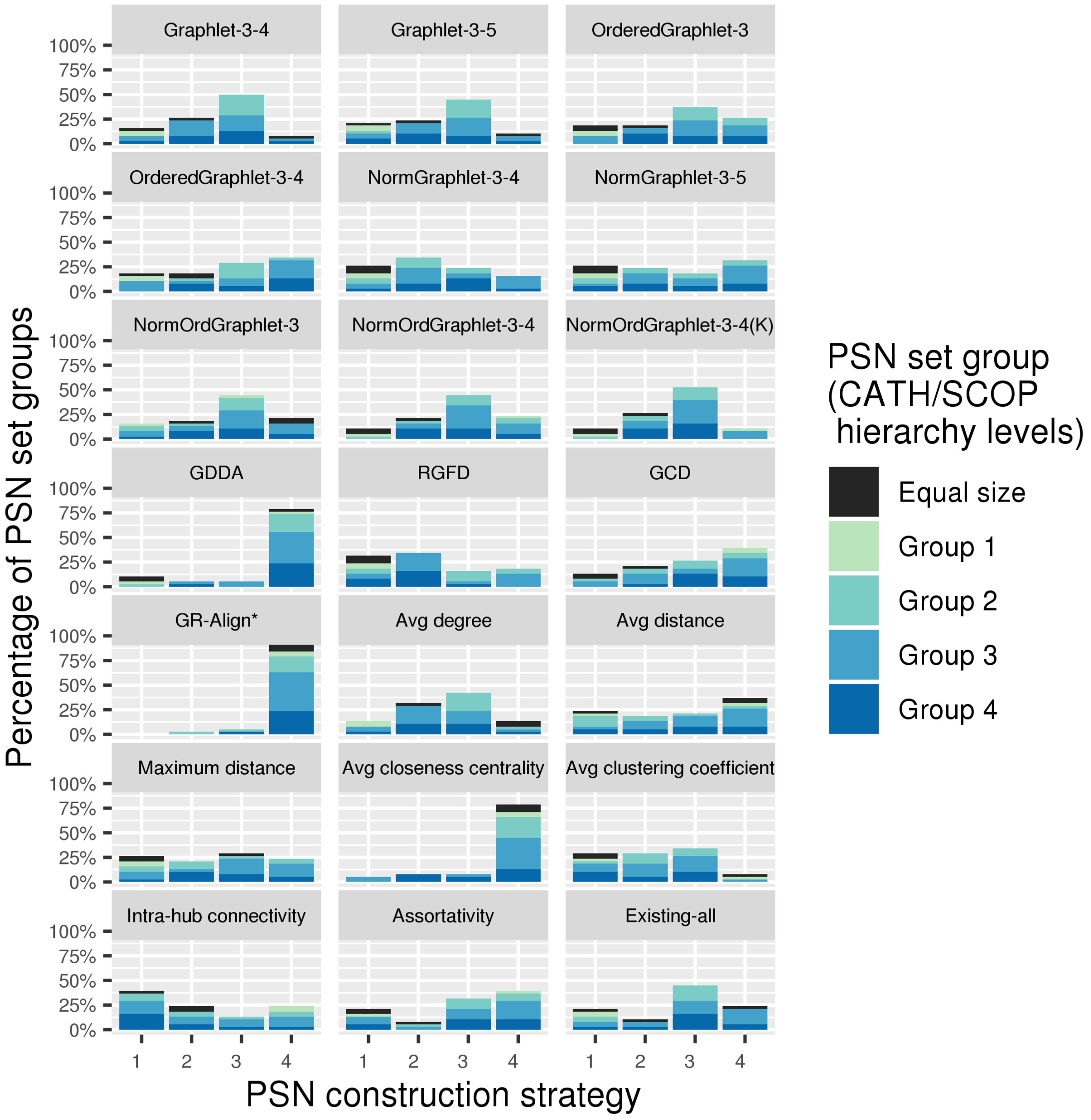

Supplementary Figure S8. Distribution of PSN sets across four PSN construction strategies: 1, 2, 3, and 4. The results are with respect to AUPR. Each panel (one panel per PC approach) shows the following: for each PSN construction strategy, we calculate the percentage of all $3+35=38$ real-world PSN sets for which the given PSN construction strategy performs the best; this is what the height of the given bar shows. Then, within each bar, we label the PSN sets according to the PSN set groups to which they belong.

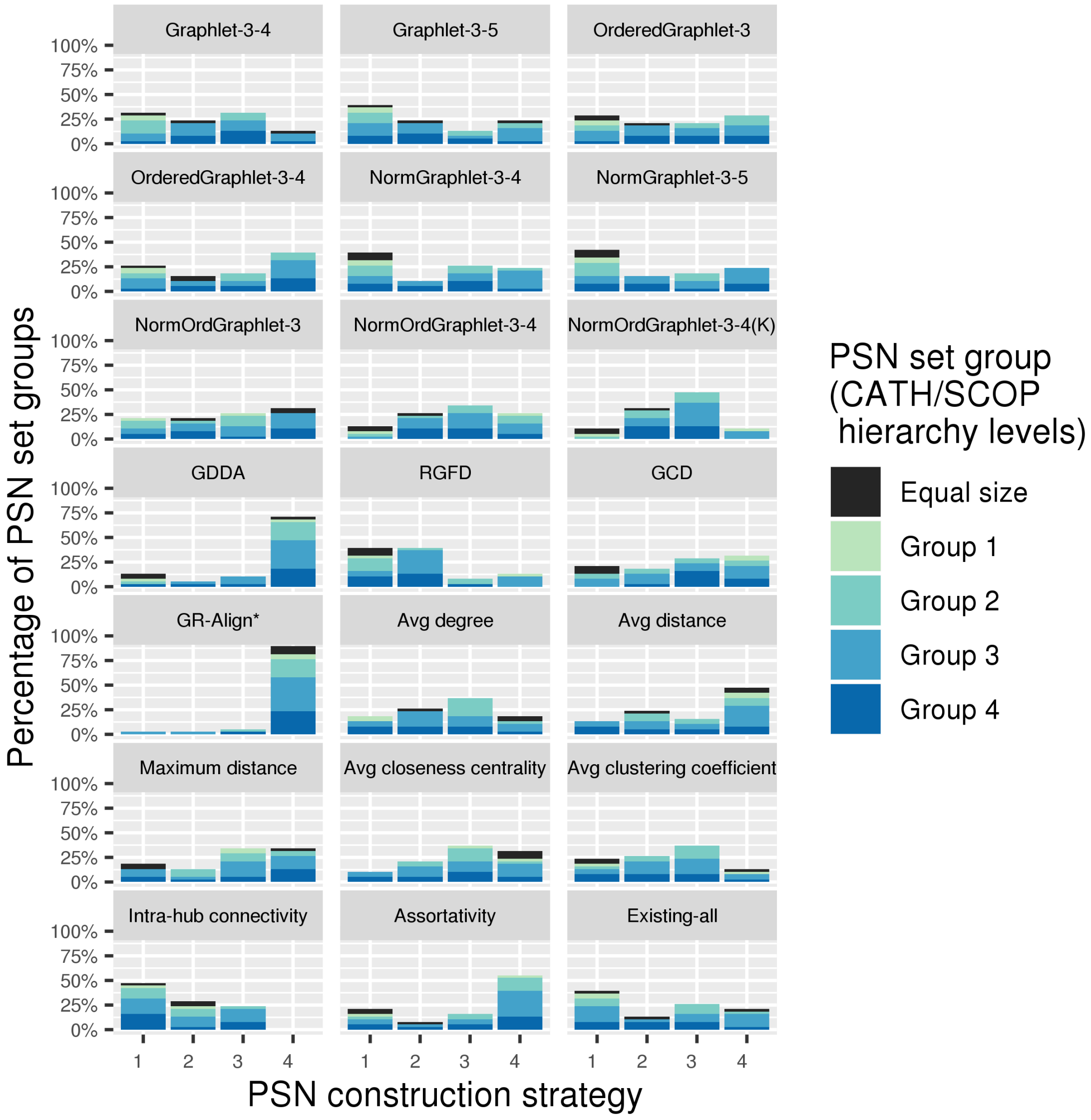

Supplementary Figure S9. Distribution of PSN sets across four PSN construction strategies: 1, 2, 3, and 4. The results are with respect to AUROC. Each panel (one panel per PC approach) shows the following: for each PSN construction strategy, we calculate the percentage of all $3+35=38$ real-world PSN sets for which the given PSN construction strategy performs the best; this is what the height of the given bar shows. Then, within each bar, we label the PSN sets according to the PSN set groups to which they belong.

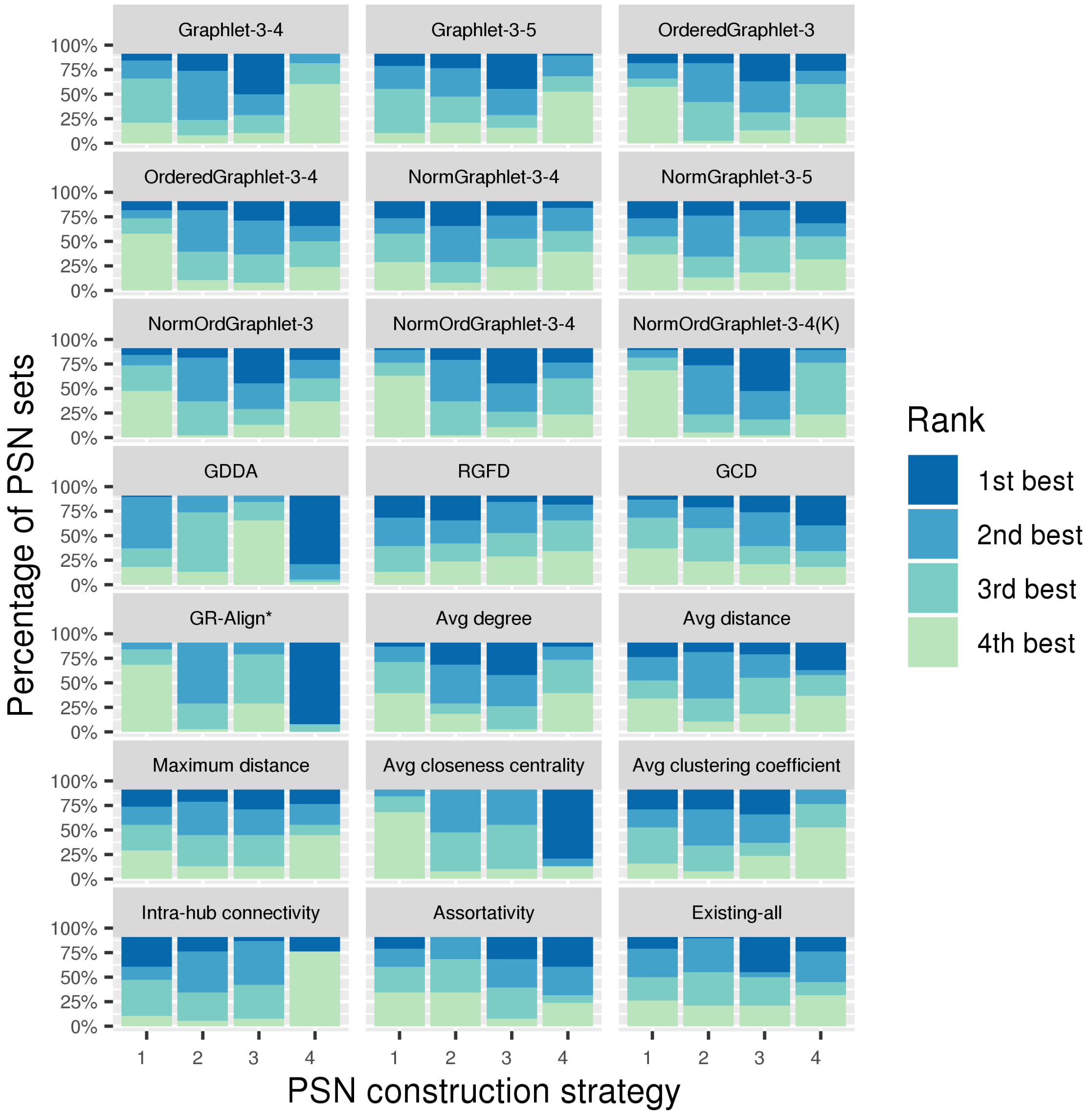

Supplementary Figure S10. The ranking of the four PSN construction strategies: 1, 2, 3, and 4. The ranking is shown with respect to AUPR. Each panel (one panel per PC approach) shows the following: for each PSN construction strategy, we calculate the percentage of all $3+35=38$ real-world PSN sets in which the given PSN construction strategy performs the best, the second best, the third best, and the fourth best.

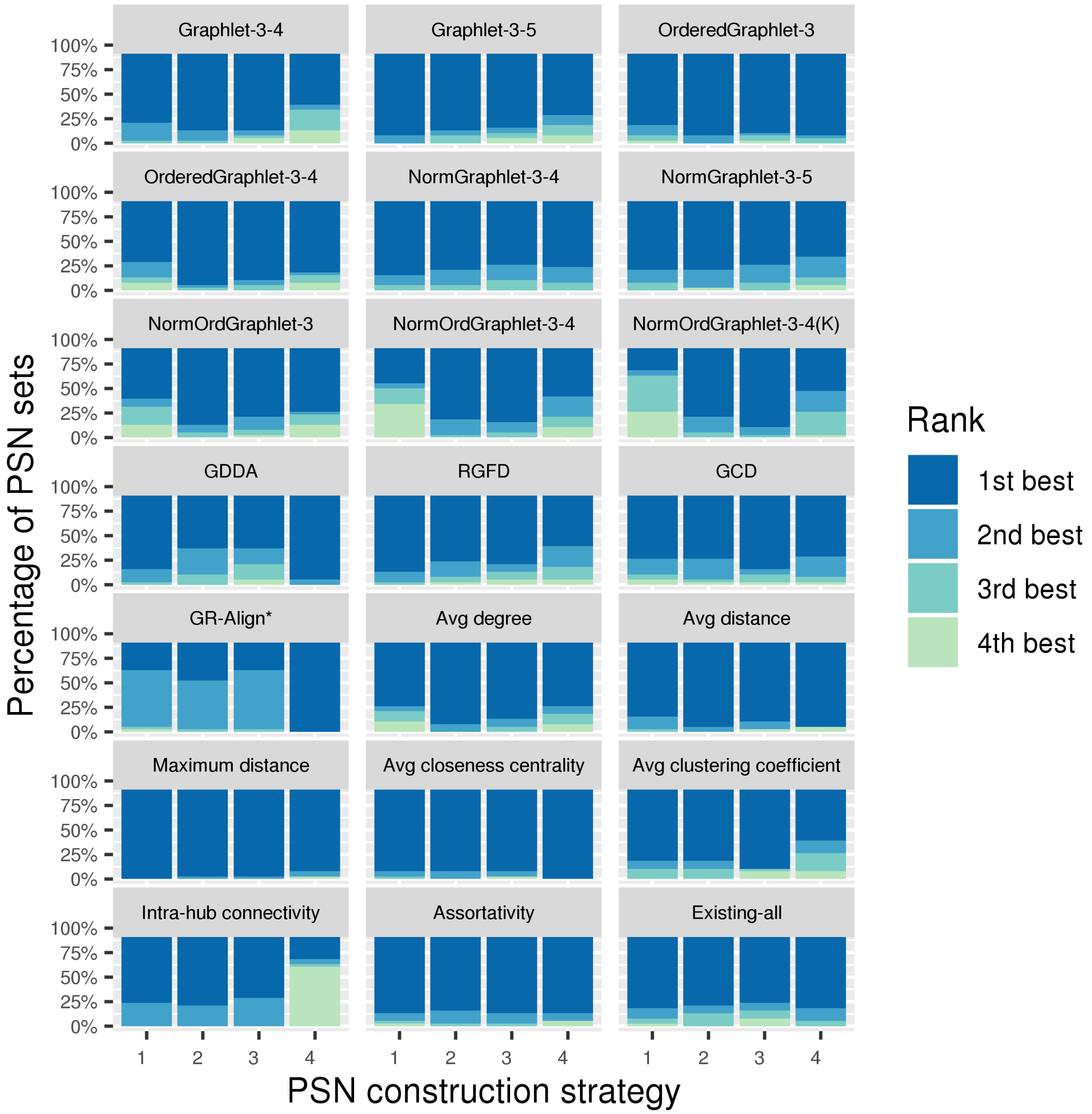

Supplementary Figure S11. The ranking of the four PSN construction strategies: 1, 2, 3, and 4. The ranking is shown with respect to AUPR. Each panel (one panel per PC approach) shows the following: for each PSN construction strategy, we calculate the percentage of all $3+35=38$ real-world PSN sets in which the given PSN construction strategy performs the best, the second best, the third best, and the fourth best. Note that unlike in Supplementary Fig. S10, here we consider two AUPR values to be tied if the absolute difference between them is $\leq 5\%$ of the maximum achievable AUPR value.

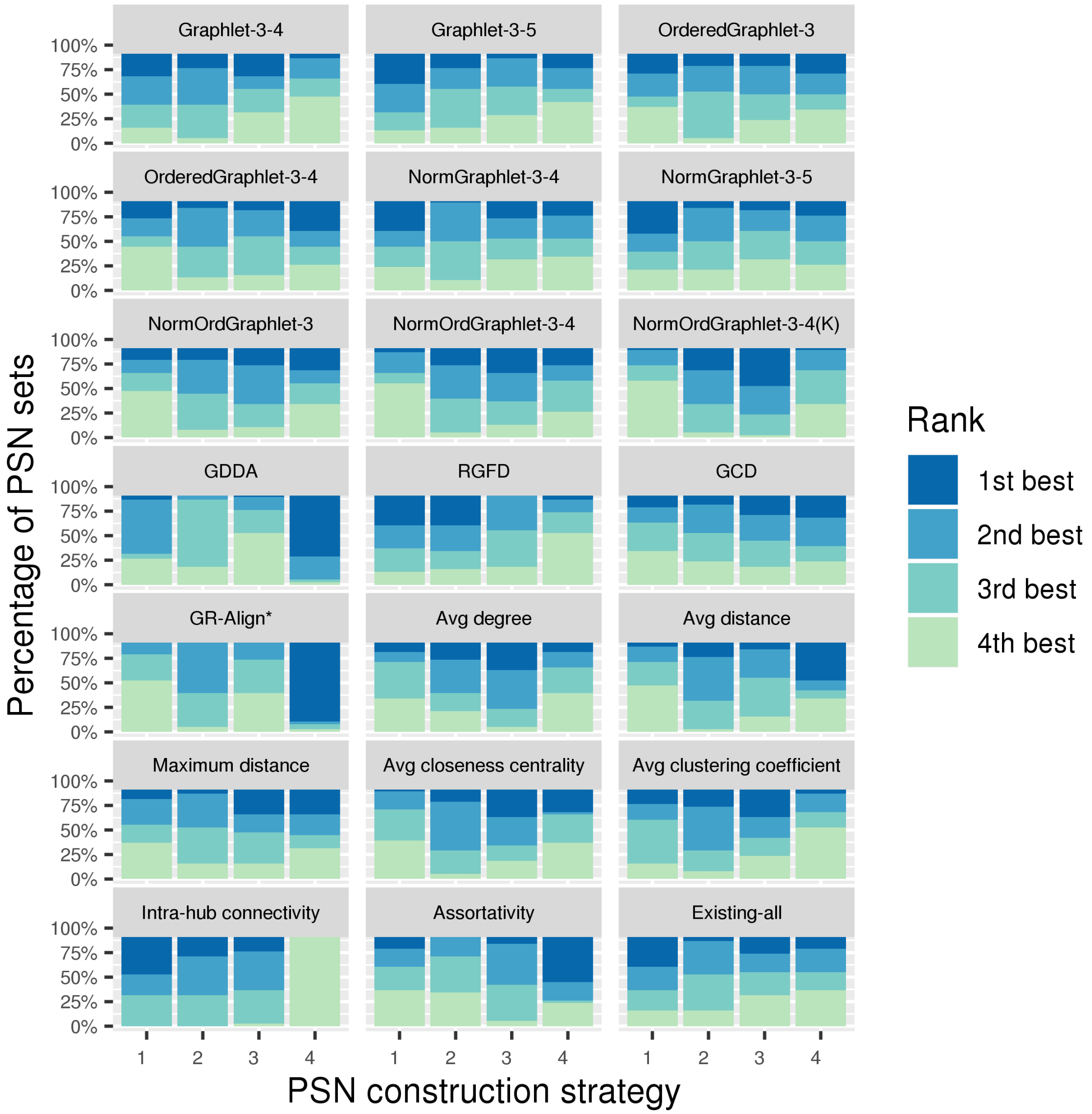

Supplementary Figure S12. The ranking of the four PSN construction strategies: 1, 2, 3, and 4. The ranking is shown with respect to AUROC. Each panel (one panel per PC approach) shows the following: for each PSN construction strategy, we calculate the percentage of all $3+35=38$ real-world PSN sets in which the given PSN construction strategy performs the best, the second best, the third best, and the fourth best.

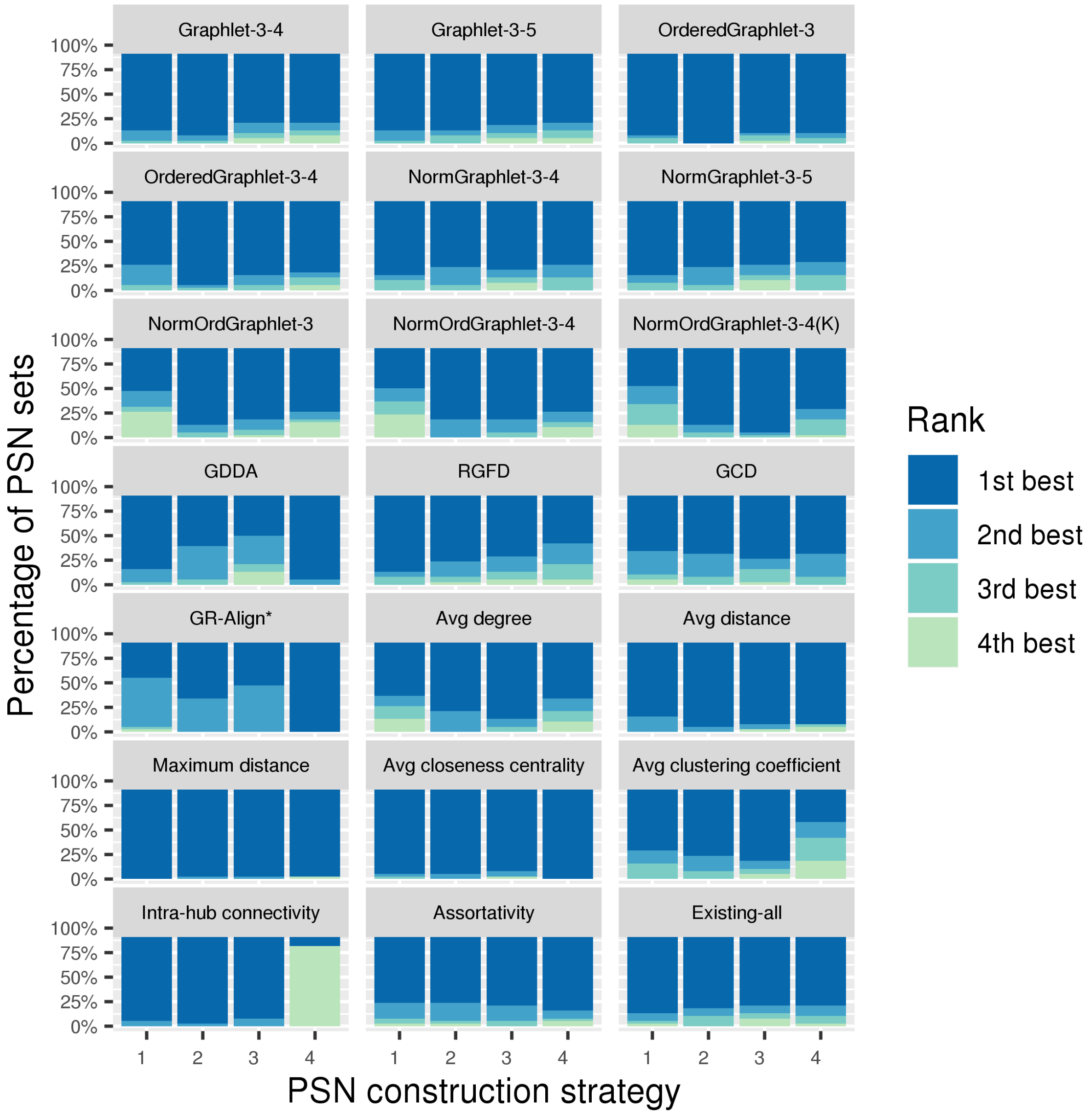

Supplementary Figure S13. The ranking of the four PSN construction strategies: 1, 2, 3, and 4. The ranking is shown with respect to AUROC. Each panel (one panel per PC approach) shows the following: for each PSN construction strategy, we calculate the percentage of all $3+35=38$ real-world PSN sets in which the given PSN construction strategy performs the best, the second best, the third best, and the fourth best. Note that unlike in Supplementary Fig. S12, here we consider two AUROC values to be tied if the absolute difference between them is $\leq 5\%$ of the maximum achievable AUROC value.

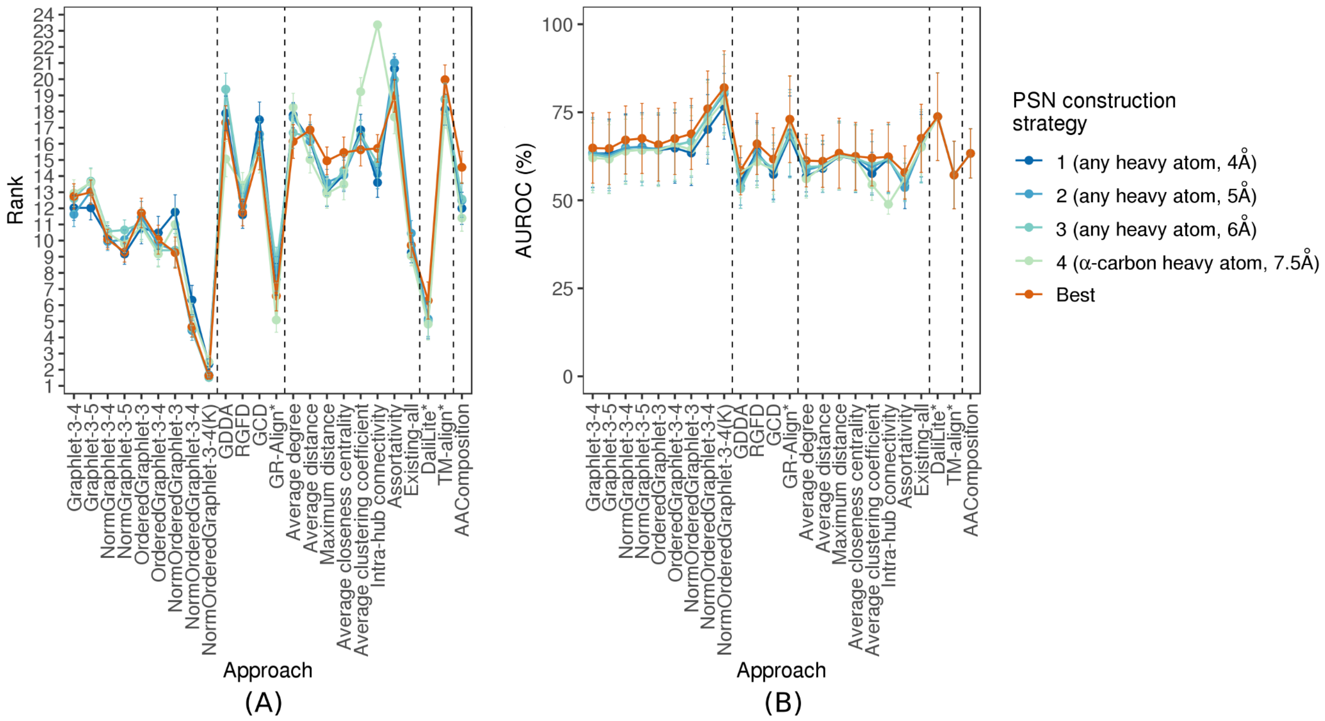

Supplementary Figure S14. The PSN construction strategy-specific performance comparison of the 24 considered PC approaches, with respect to AUROC, in terms of: (A) the approaches' ranks compared to one another, and (B) the approaches' raw AUROC values. In panel (A), for each PSN construction strategy, for a given PSN set, the 24 approaches are ranked from the best (rank 1) to the worst (rank 24). Then, for a given approach, its 35 ranks (corresponding to the 35 PSN sets) are averaged (the average ranks are denoted by circles, and bars denote the corresponding standard deviations). So, the lower the average rank, the better the approach. In panel (B), for each PSN construction strategy, for each approach, its 35 raw AUROC values (corresponding to the 35 PSN sets) are averaged (the average values are denoted by circles, and bars denote the corresponding standard deviations). So, the higher the average AUROC value, the better the approach. The trends are very similar with respect to AUPR as well (Fig. 8 in the main manuscript). These results are for the "all group" PSN set group that spans the 35 PSN sets of different sizes. Equivalent results for the individual groups 1-4 are shown in Supplementary Fig. S15-S18.

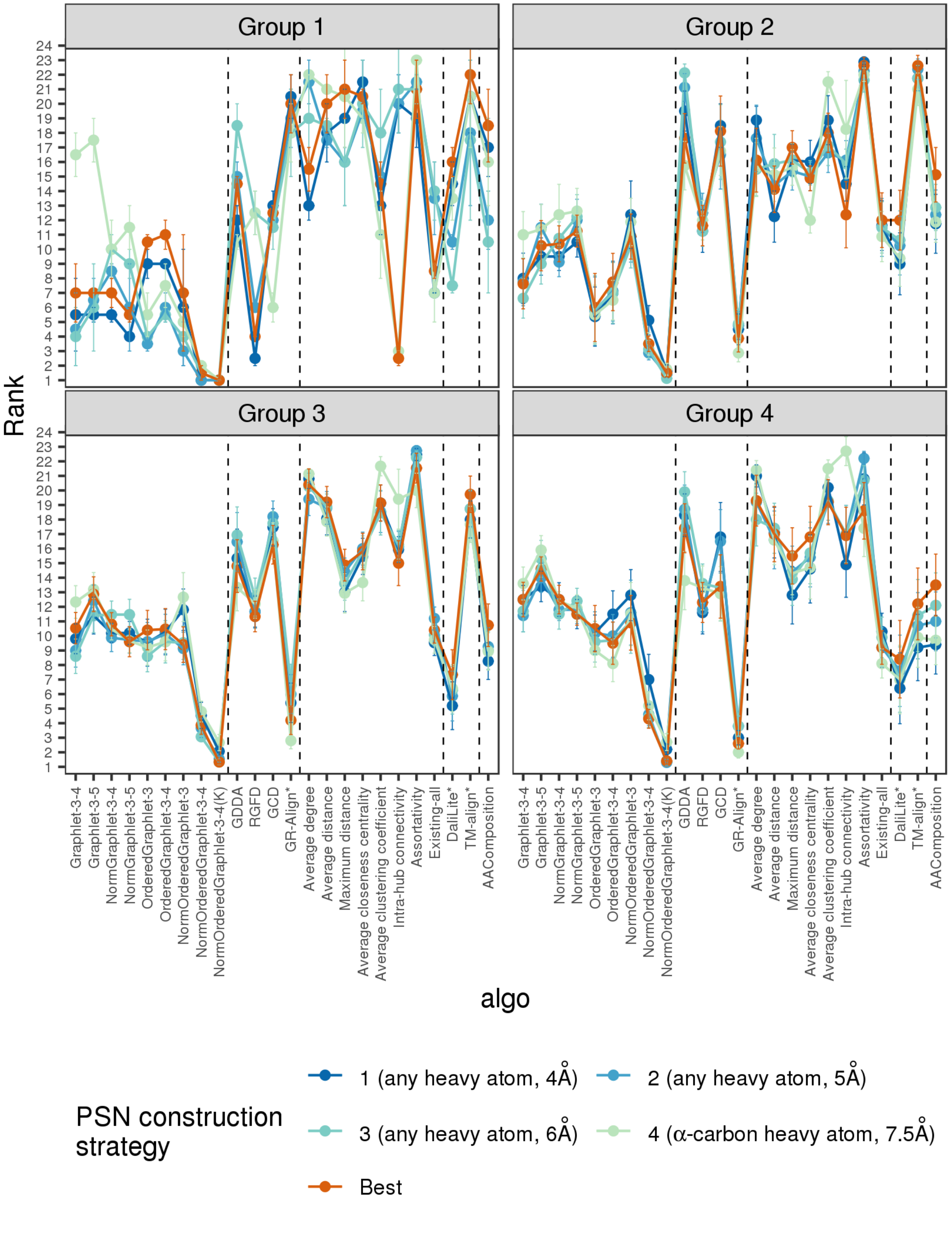

Supplementary Figure S15. The PSN construction strategy-specific rank performance comparison of the 24 considered PC approaches, with respect to AUPR, corresponding to PSN set group : (A) 1, (B) 2, (C) 3, and (D) 4. For each PSN construction strategy, for a given PSN set, the 24 approaches are ranked from the best (rank 1) to the worst (rank 24). Then, for a given approach, its 35 ranks (corresponding to the 35 PSN sets) are averaged (the average ranks are denoted by circles, and bars denote the corresponding standard deviations). So, the lower the average rank, the better the approach. The trends are very similar with respect to AUROC as well (Supplementary Fig. S16).

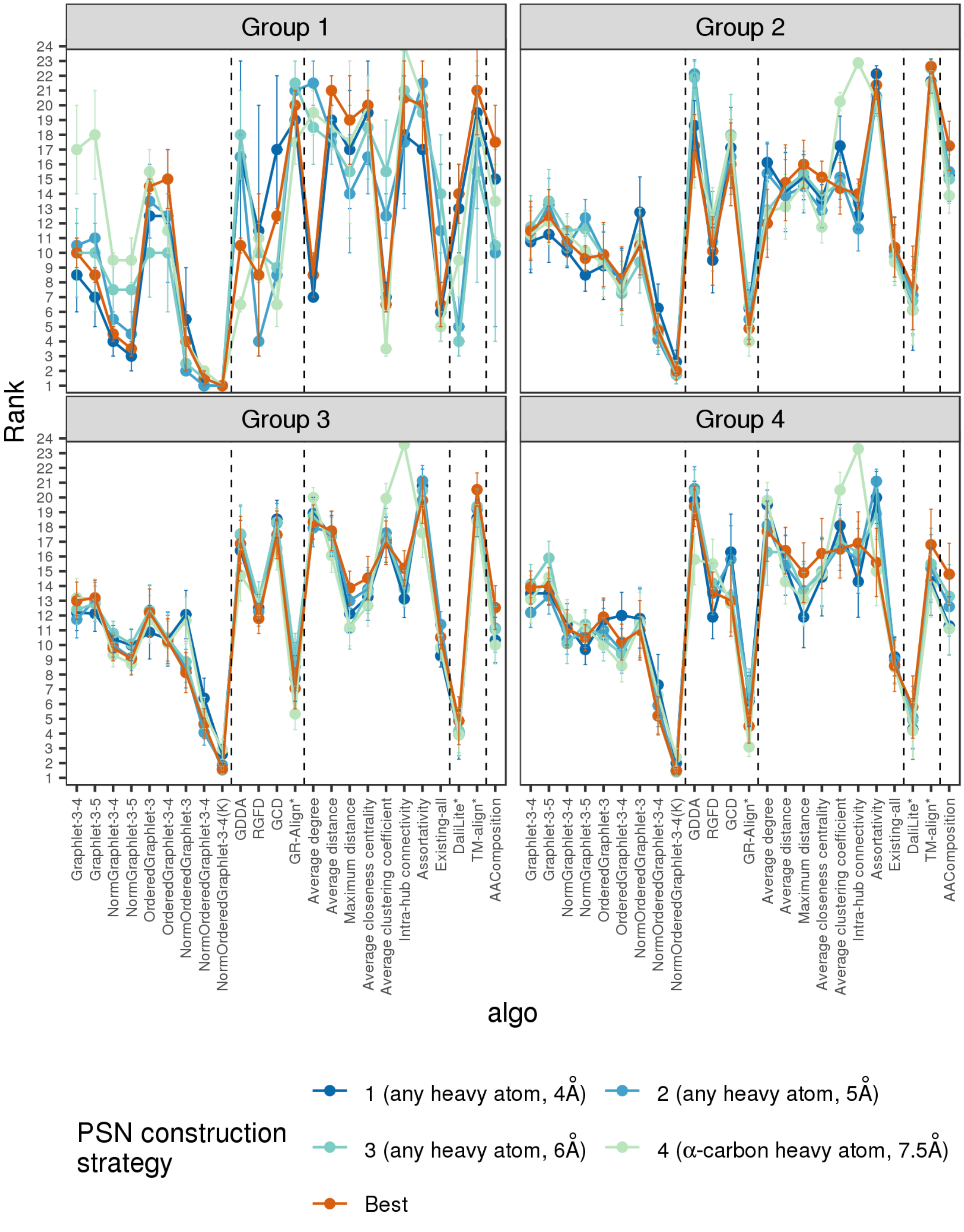

Supplementary Figure S16. The PSN construction strategy-specific rank performance comparison of the 24 considered PC approaches, with respect to AUROC, corresponding to PSN set group : (A) 1, (B) 2, (C) 3, and (D) 4. For each PSN construction strategy, for a given PSN set, the 24 approaches are ranked from the best (rank 1) to the worst (rank 24). Then, for a given approach, its 35 ranks (corresponding to the 35 PSN sets) are averaged (the average ranks are denoted by circles, and bars denote the corresponding standard deviations). So, the lower the average rank, the better the approach. The trends are very similar with respect to AUPR as well (Supplementary Fig. S15).

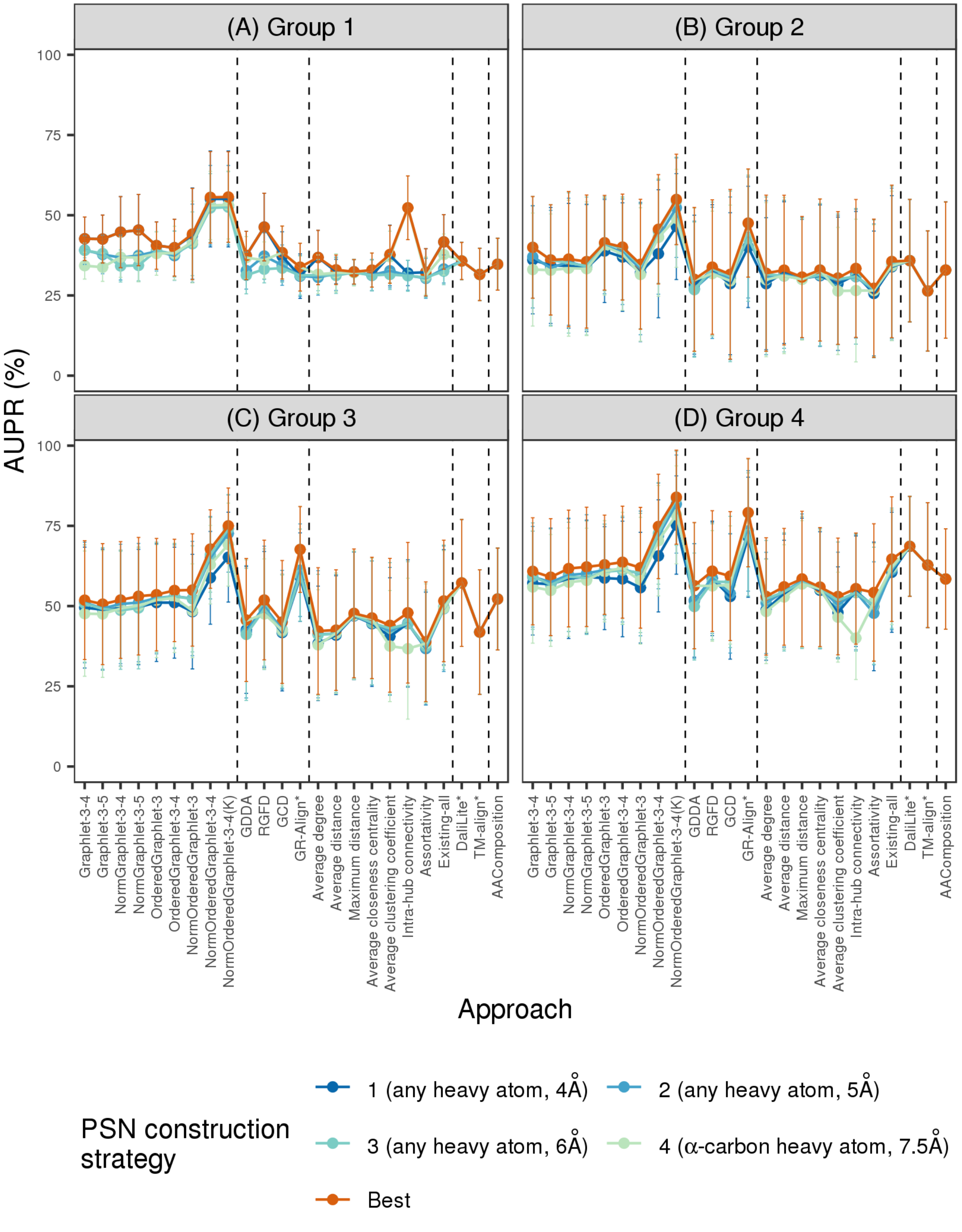

Supplementary Figure S17. The PSN construction strategy-specific performance comparison of the 24 considered PC approaches, with respect to AUPR values (expressed as percentages), corresponding to PSN set group : (A) 1, (B) 2, (C) 3, and (D) 4. For each PSN construction strategy, for each approach, its 35 raw AUPR values (corresponding to the 35 PSN sets) are averaged (the average values are denoted by circles, and bars denote the corresponding standard deviations). So, the higher the average AUPR value, the better the approach. The trends are very similar with respect to AUROC values as well (Supplementary Fig. S18).

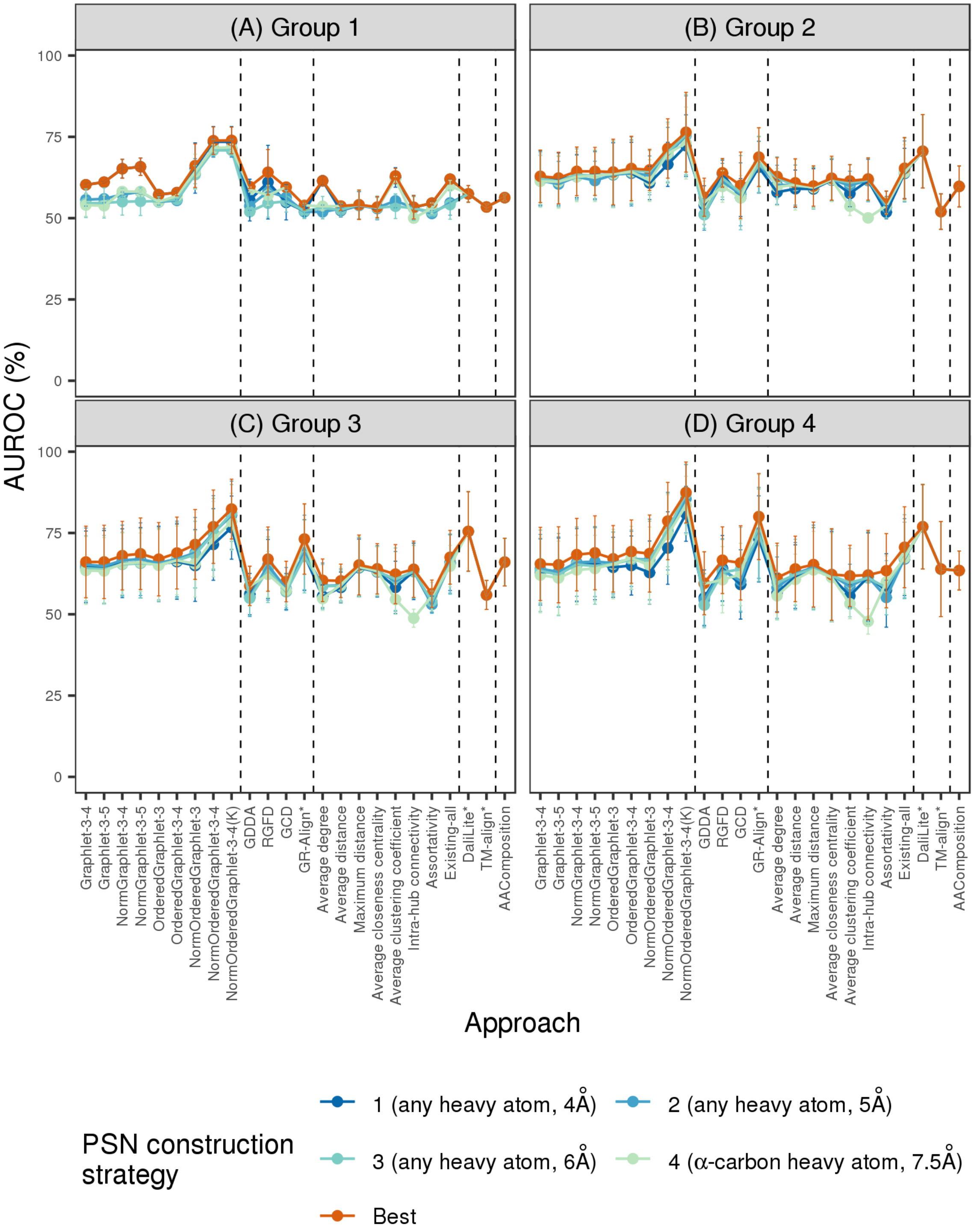

Supplementary Figure S18. The PSN construction strategy-specific performance comparison of the 24 considered PC approaches, with respect to AUROC values (expressed as percentages), corresponding to PSN set group : (A) 1, (B) 2, (C) 3, and (D) 4. For each PSN construction strategy, for each approach, its 35 raw AUROC scores (corresponding to the 35 PSN sets) are averaged (the average values are denoted by circles, and bars denote the corresponding standard deviations). So, the higher the average AUROC value, the better the approach. The trends are very similar with respect to AUPR values as well (Supplementary Fig. S17).

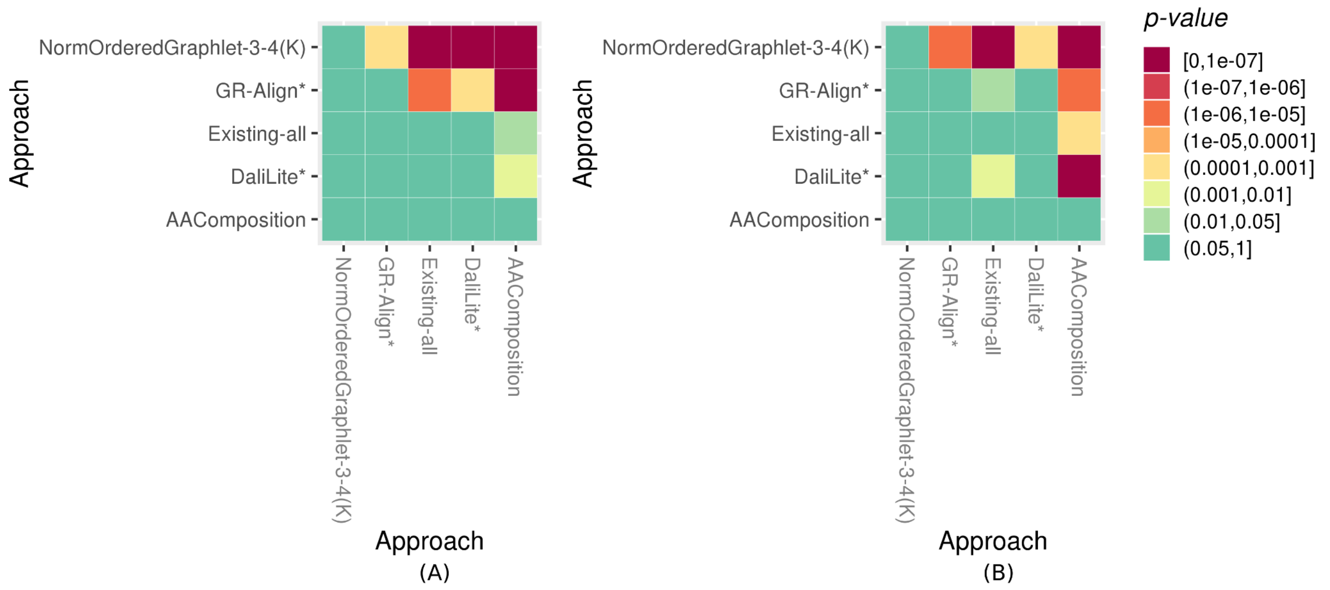

Supplementary Figure S19. Statistical significance of the difference between average ranks of the PC approaches, with respect to: (A) AUPR and (B) AUROC. For aesthetics, these results are only for the best approach in each category, namely: the best of our proposed PCA graphlet-based network approaches (GRAFENE version NormOrderedGraphlet-3-4(K)), the best of the existing non-PCA graphlet-based network approaches (GR-Align), the best of the existing non-graphlet network approaches (Existing-all), the best of the existing non-network 3D structural approaches (DaliLite), and the sequence-based approach (AAComposition). For each of the 35 PSN sets, the five approaches are ranked from the best (rank 1) to the worst (rank 5). Hence, for each approach, there are 35 ranks (corresponding to the 35 PSN sets). For each pair of approaches, we compare the two given approaches' 35 ranks using paired t-test. In the figure, every cell (i, j) indicates the statistical significance (in terms of p-value) of approach i being superior to approach j. The results are similar when we use raw AUPR/AUROC values instead of ranks (Supplementary Fig. S20).

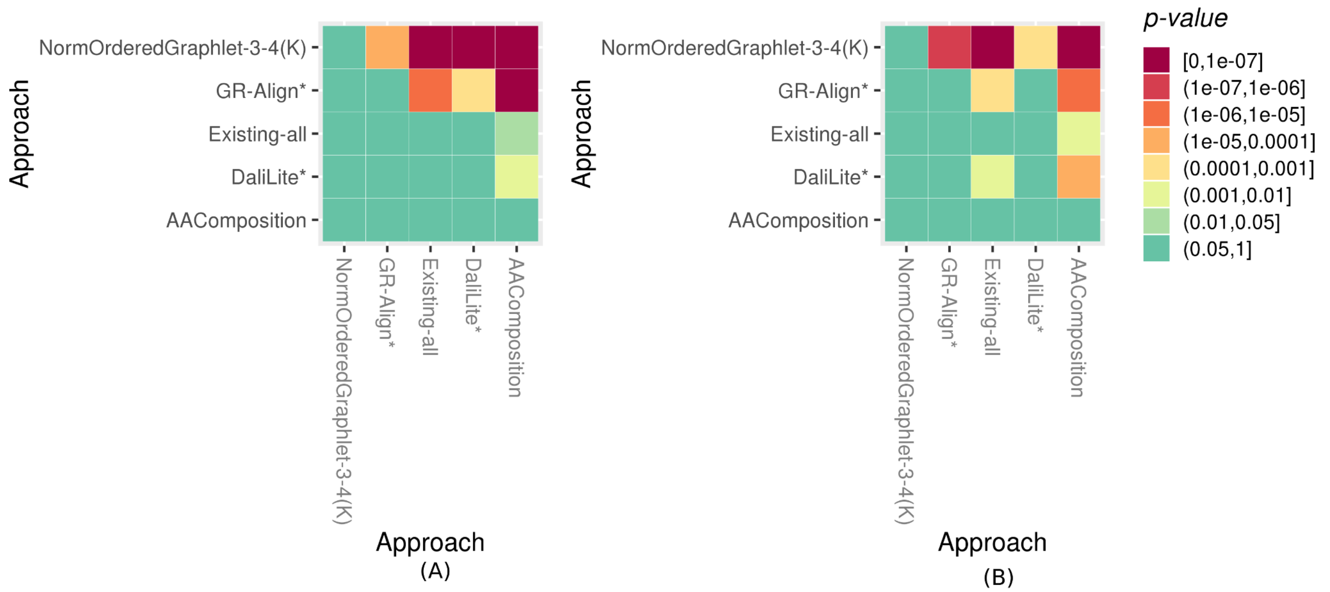

Supplementary Figure S20. Statistical significance of the difference between average raw values of the PC approaches, with respect to: (A) AUPR and (B) AUROC. For aesthetics, these results are only for the best approach in each category, namely: the best of our proposed PCA graphlet-based network approaches (GRAFENE version NormOrderedGraphlet-3-4(K)), the best of the existing non-PCA graphlet-based network approaches (GR-Align), the best of the existing non-graphlet network approaches (Existing-all), the best of the existing non-network 3D structural approaches (DaliLite), and the sequence-based approach (AAComposition). For each of the 35 PSN sets, raw AUPR/AUROC values for all five approaches are measured. Hence, for each approach, there are 35 raw AUPR/AUROC values (corresponding to the 35 PSN sets). For each pair of approaches, we compare the two given approaches' 35 raw AUPR/AUROC values using paired t-test. In the figure, every cell (i, j) indicates the statistical significance (in terms of p-value) of approach i being superior to approach j. The results are similar when we use ranks instead of raw AUPR/AUROC values (Supplementary Fig. S19).

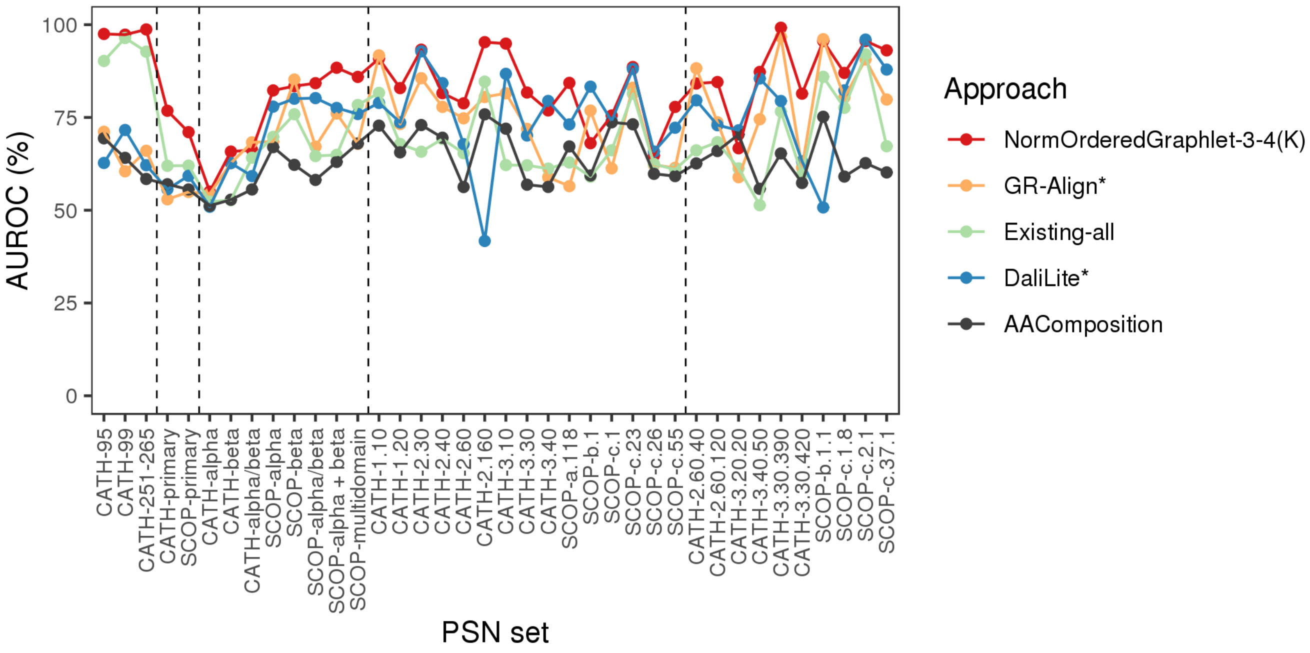

Supplementary Figure S21. The performance comparison of only the best PC approach in each category (for aesthetics purposes) on all three “equal size” PSN sets and all 35 PSN sets of different size, with respect to raw AUROC values. Namely, results are shown for: the best of our proposed PCA graphlet-based network approaches (GRAFENE version NormOrderedGraphlet-3-4(K)), the best of the existing non-PCA graphlet-based network approaches (GR-Align), the best of the existing non-graphlet network approaches (Existing-all), the best of the existing non-network 3D structural approaches (DaliLite), and the sequence-based approach (AAComposition). The vertical dotted lines separate the PSN sets into the five PSN set groups, namely (from left to right): “equal size”, group 1, group 2, group 3, and group 4. For the equivalent results in terms of raw AUPR values, see Fig. 9 in the main manuscript.

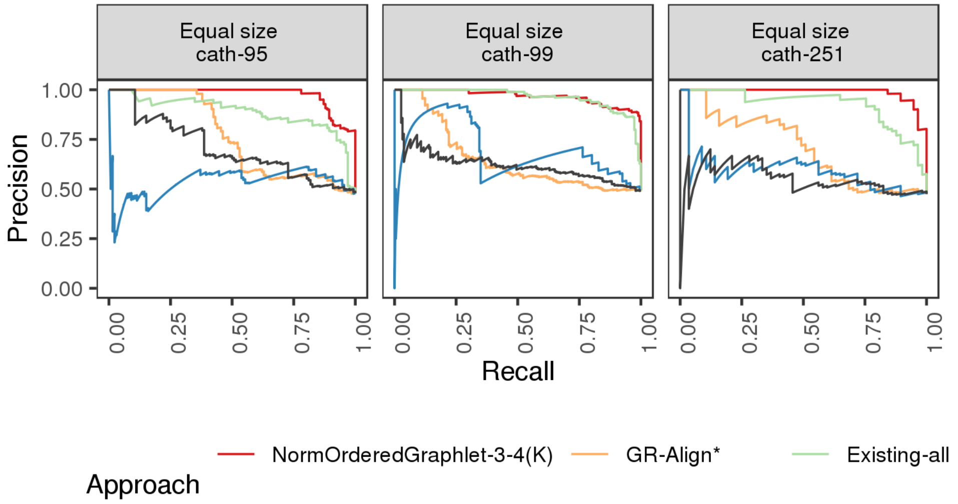

(A)

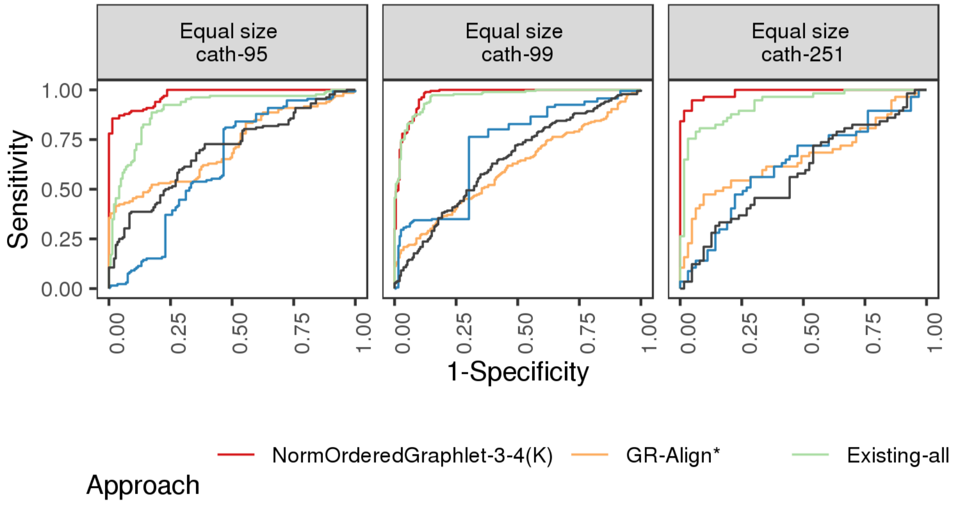

(B)

Supplementary Figure S22. (A) Precision-recall (PR) and (B) receiver operating characteristic (ROC) curves for the best approach in each category, namely: the best of our proposed PCA graphlet-based network approaches (GRAFENE version NormOrderedGraphlet-3-4(K)), the best of the existing non-PCA graphlet-based network approaches (GR-Align), the best of the existing non-graphlet network approaches (Existing-all), the best of the existing non-network 3D structural approaches (DaliLite), and the sequence-based approach (AAComposition). The results are for the three "equal-size" PSN sets. Also, these results are for the best PSN construction strategy.

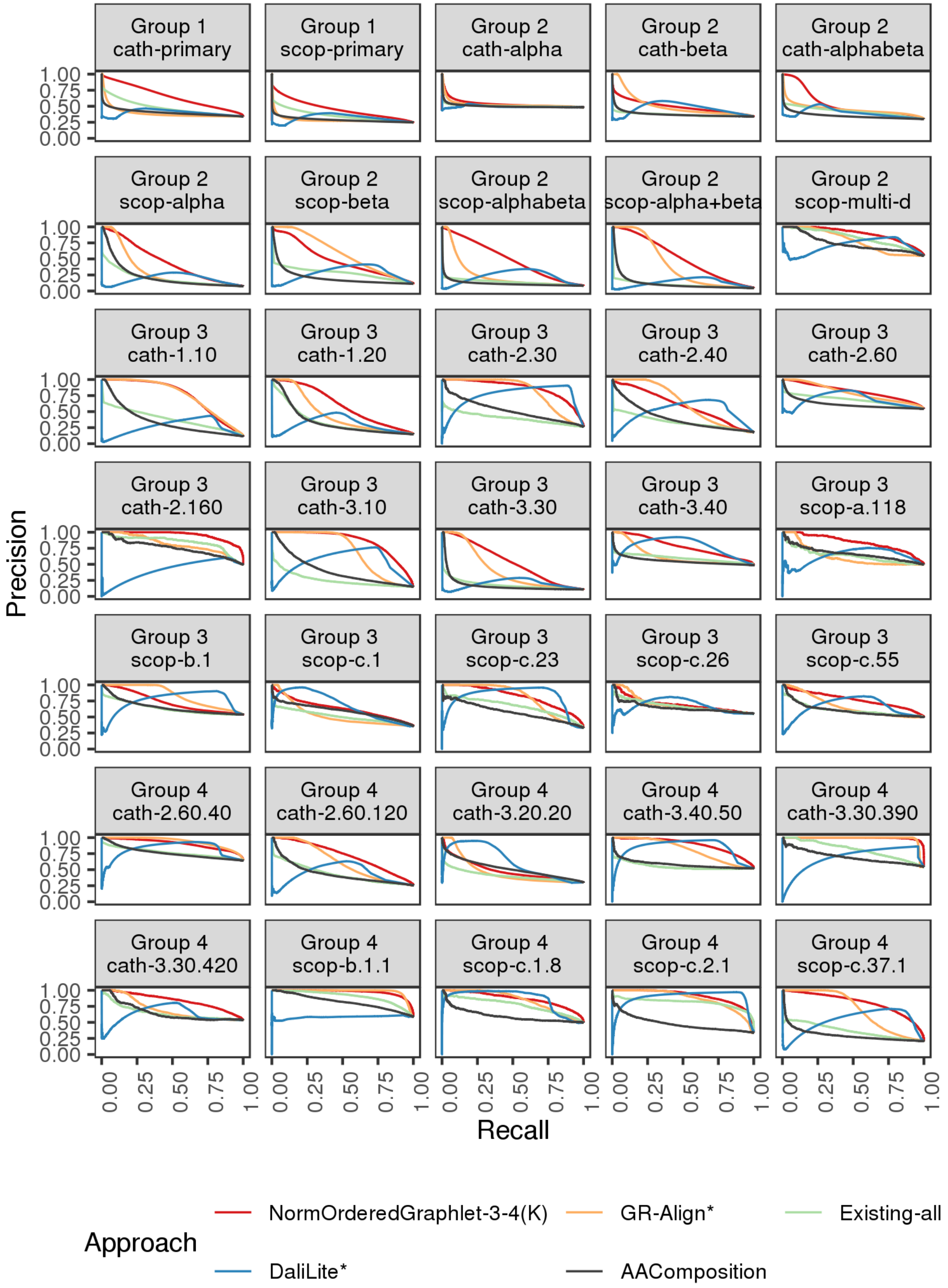

Supplementary Figure S23. Precision-recall (PR) curves for the best approach in each category, namely: the best of our proposed PCA graphlet-based network approaches (GRAFENE version NormOrderedGraphlet-3-4(K)), the best of the existing non-PCA graphlet-based network approaches (GR-Align), the best of the existing non-graphlet network approaches (Existing-all), the best of the existing non-network 3D structural approaches (DaliLite), and the sequence-based approach (AAComposition). These results are for the 35 PSN sets of different size. Also, these results are for the best PSN construction strategy.

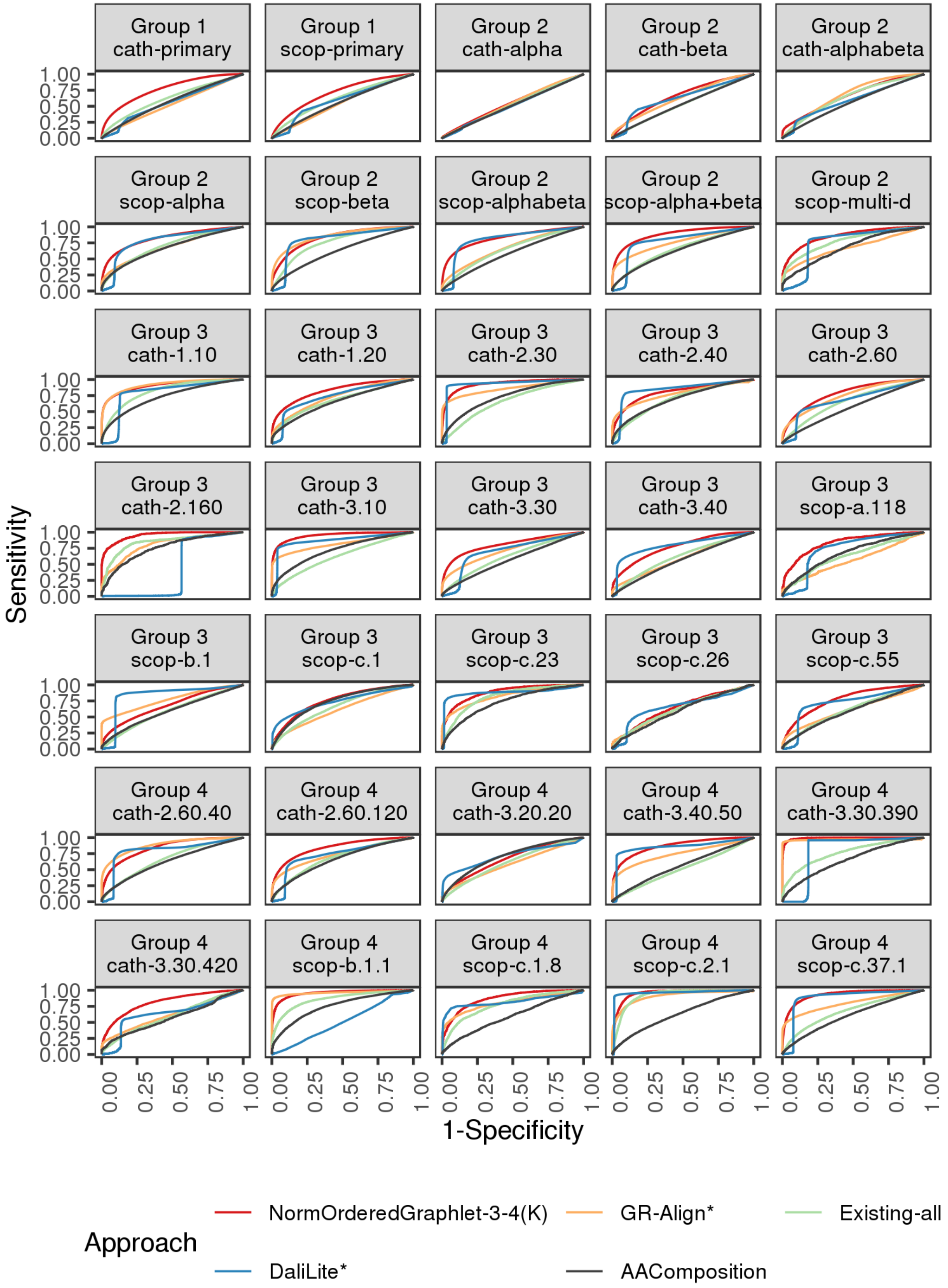

Supplementary Figure S24. Receiver operating characteristic (ROC) curves for the best approach in each category, namely: the best of our proposed PCA graphlet-based network approaches (GRAFENE version NormOrderedGraphlet-3-4(K)), the best of the existing non-PCA graphlet-based network approaches (GR-Align), the best of the existing non-graphlet network approaches (Existing-all), the best of the existing non-network 3D structural approaches (DaliLite), and the sequence-based approach (AAComposition). These results are for the 35 PSN sets of different size. Also, these results are for the best PSN construction strategy.

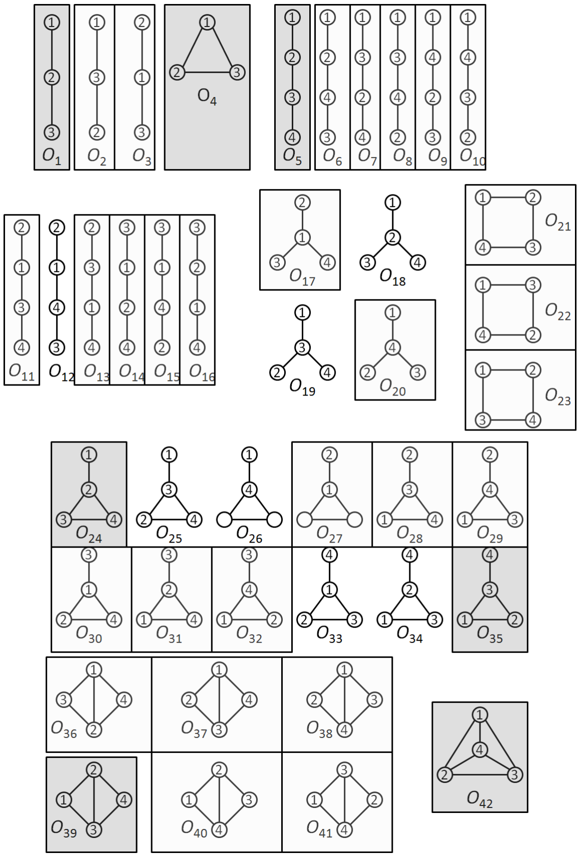

Supplementary Figure S25. Ordered graphlets that are significantly represented in α (dark gray) or β (light gray) PSNs.

III Supplementary Tables

Supplementary Table S1. Synthetic network sets that we use. For the given data set, the second column indicates whether its networks are of the same size or different sizes, and the last three columns indicate the number of its networks as well as their size(s) in terms of the number of nodes and edges.

Data set			Number of		
Type	Size	Name	Networks	Nodes	Edges
Synthetic networks	Same	Synthetic-100	150	100	400
		Synthetic-500	150	500	2,000
		Synthetic-1000	150	1,000	4,000
	Different	Synthetic-all	450	100-1,000	400-4,000

Supplementary Table S2. The number of categories and the number of PSNs averaged over all categories for each of the 35 real-world PSN sets, with respect to four different PSN construction strategies: first (any heavy atom, 4 Å), second (any heavy atom, 5 Å), third (any heavy atom, 6 Å), and fourth (α-carbon heavy atom, 7.5 Å).

	PSN construction strategy 1		PSN construction strategy 2		PSN construction strategy 3		PSN construction strategy 4	
	# of categories	Avg # of PSNs/category	# of categories	Avg # of PSNs/category	# of categories	Avg # of PSNs/category	# of categories	Avg # of PSNs/category
CATH-primary	3	3170	3	3167	3	3133	3	3153
CATH-α	4	655	4	656	4	650	4	541
CATH-β	10	297	10	297	10	295	10	295
CATH-α/β	4	947	4	947	4	935	4	944
CATH-1.10	12	72	12	72	12	71	12	72
CATH-1.20	8	60	8	59	8	59	8	59
CATH-2.30	4	51	4	51	4	51	4	51
CATH-2.40	7	76	7	76	7	75	7	74
CATH-2.60	2	717	2	718	2	716	2	716
CATH-2.160	2	35	2	35	2	35	2	35
CATH-3.10	7	62	7	62	7	61	7	61
CATH-3.30	14	79	14	79	14	79	14	78
CATH-3.40	3	212	3	212	3	203	3	212
CATH-2.60.40	3	212	3	212	3	210	3	212
CATH-2.60.120	4	92	4	93	4	93	4	92
CATH-3.20.20	5	123	5	123	5	123	5	123
CATH-3.30.390	2	44	2	44	2	44	2	44
CATH-3.30.420	2	78	2	78	2	78	2	78
CATH-3.40.50	2	145	2	145	2	145	2	145
SCOP-primary	7	1636	7	1638	7	1628	7	1624
SCOP-α	16	57	16	58	16	58	16	57
SCOP-β	21	88	21	88	21	88	21	88
SCOP-α/β	26	113	26	114	26	112	26	113
SCOP-$\alpha+\beta$	28	57	28	57	28	57	28	57
SCOP-multidomain	2	63	2	63	2	63	2	63
SCOP-a.118	2	35	2	35	2	35	2	35
SCOP-b.1	3	144	3	144	3	144	3	144
SCOP-c.1	4	75	4	75	4	75	4	75
SCOP-c.23	3	36	3	36	3	34	3	35
SCOP-c.26	2	47	2	47	2	47	2	47
SCOP-c.55	2	90	2	90	2	90	2	90
SCOP-b.1.1	2	141	2	141	2	141	2	141
SCOP-c.1.8	2	54	2	54	2	54	2	54
SCOP-c.2.1	4	54	4	54	4	54	4	54
SCOP-c.37.1	6	55	6	54	6	54	6	54

Supplementary Table S3. Details about our PSN sets belonging to the second-level hierarchical categories of CATH and SCOP. At the top-level of the CATH hierarchy, there are three categories: α, β, and α/β. At the top-level of the SCOP hierarchy, there are five categories: α, β, α/β, α+β, and Multi domain. Each top-level category has multiple second-level categories, as shown in the table. For example, the α top-level hierarchical category of CATH has four second-level categories: Orthogonal Bundle, Up-down Bundle, Alpha Horseshoe, and Alpha/Alpha Barrel. For each top-level hierarchical category, we specify its name and label (separated by semicolon), where the labels are as given by CATH/SCOP. For each second-level hierarchical category, we specify its name and the number of PSNs (shown in parentheses).

	Top-level hierarchical categories	Second-level hierarchical categories
CATH	α; 1	1. Orthogonal Bundle (1632)<br>2. Up-down Bundle (807)<br>3. Alpha Horseshoe (133)<br>4. Alpha/Alpha Barrel (53)
	β; 2	1. Ribbon (44)<br>2. Roll (242)<br>3. Beta Barrel (699)<br>4. Sandwich (1562)<br>5. Distorted Sandwich (102)<br>6. Trefoil (79)<br>7. 6 Propellor (45)<br>8. 7 Propellor (42)<br>9. 3 Solenoid (70)<br>10. Beta Complex (87)
	α/β; 3	1. Roll (611)<br>2. Alpha-Beta Barrel (839)<br>3. 2-Layer Sandwich (1668)<br>4. 3-Layer(aba) Sandwich (675)
SCOP	α; a	1. Globin-like (95)<br>2. Cytochrome c (35)<br>3. DNA/RNA-binding 3-helical bundle (113)<br>4. Spectrin repeat-like (41)<br>5. Four-helical up-and-down bundle (76)<br>6. Ferritin-like (66)<br>7. 4-helical cytokines (38)<br>8. Bromodomain-like (41)<br>9. EF Hand-like (64)<br>10. GST C-terminal domain-like (49)<br>11. SAM domain-like (33)<br>12. Alpha/alpha toroid (53)<br>13. Alpha-alpha superhelix (113)<br>14. Tetracyclin repressor-like, C-terminal domain (35)<br>15. Nuclear receptor ligand-binding domain (30)<br>16. Phospholipase A2, PLA2 (37)
	β; b	1. Immunoglobulin-like beta-sandwich (528)<br>2. Common fold of diphtheria toxin/transcription factors/cytochrome f (85)<br>3. Cupredoxin-like (77)<br>4. C2 domain-like (33)<br>5. Galactose-binding domain-like (68)<br>6. Concanavalin A-like lectins/glucanases (119)<br>7. SH3-like barrel (60)<br>8. PDZ domain-like (39)<br>9. OB-fold (122)<br>10. Beta-Trefoil (61)<br>11. Reductase/isomerase/ elongation factor common domain (39)<br>12. Split barrel-like (33)<br>13. Trypsin-like serine proteases (96)<br>14. Acid proteases (33)<br>15. PH domain-like barrel (83)<br>16. Lipocalins (65)<br>17. 6-bladed beta-propeller (33)<br>18. 7-bladed beta-propeller (35)<br>19. Single-stranded right-handed beta-helix (37)<br>20. Nucleoplasmin-like/VP (viral coat and capsid proteins) (95)<br>21. Double-stranded beta-helix (114)
	α/β; c	1. TIM beta/alpha-barrel (519)<br>2. NAD(P)-binding Rossmann-fold domains (291)<br>3. FAD/NAD(P)-binding domain (102)<br>4. The "swivelling" beta/beta/alpha domain (35)<br>5. Leucine-rich repeat, LRR (right-handed beta-alpha superhelix) (35)<br>6. ClpP/crotonase (38)<br>7. Flavodoxin-like (173)<br>8. Adenine nucleotide alpha hydrolase-like (95)<br>9. Thiamin diphosphate-binding fold (THDP-binding) (45)<br>10. P-loop containing nucleoside triphosphate hydrolases (422)<br>11. Thioredoxin fold (108)<br>12. Anticodon-binding domain-like (31)<br>13. Restriction endonuclease-like (61)

Supplementary Table S2 – continued on next page

	Top-level hierarchical categories	Second-level hierarchical categories
SCOP	α/β; c	14. Ribonuclease H-like motif (211)<br>15. Phosphorylase/hydrolase-like (76)<br>16. PRTase-like (39)<br>17. S-adenosyl-L-methionine-dependent methyltransferases (128)<br>18. PLP-dependent transferase-like (87)<br>19. Nucleotide-diphospho-sugar transferases (42)<br>20. Alpha/beta-Hydrolases (117)<br>21. Ribokinase-like (33)<br>22. Periplasmic binding protein-like I (32)<br>23. Periplasmic binding protein-like II (95)<br>24. Thiolase-like (43)<br>25. HAD-like (61)<br>26. NagB/RpiA/CoA transferase-like (31)
	α+β; d	1. Lysozyme-like (33)<br>2. Cysteine proteinases (73)<br>3. Ribosomal protein S5 domain 2-like (53)<br>4. Beta-Grasp (ubiquitin-like) (56)<br>5. Cystatin-like (79)<br>6. UBC-like (40)<br>7. Glyoxalase/Bleomycin resistance protein/Dihydroxybiphenyl dioxygenase (45)<br>8. Thioesterase/thiol ester dehydrase-isomerase (56)<br>9. Alpha/beta-Hammerhead (32)<br>10. Ferredoxin-like (213)<br>11. Bacillus chorismate mutase-like (63)<br>12. FwdE/GAPDH domain-like (50)<br>13. Zincin-like (70)<br>14. SH2-like (38)<br>15. Acyl-CoA N-acyltransferases (Nat) (79)<br>16. Profilin-like (55)<br>17. Nudix (31)<br>18. TBP-like (71)<br>19. ATP-grasp (41)<br>20. Protein kinase-like (PK-like) (84)<br>21. Ntn hydrolase-like (63)<br>22. Metallo-hydrolase/oxidoreductase (34)<br>23. Metallo-dependent phosphatases (31)<br>24. LDH C-terminal domain-like (30)<br>25. DNA breaking-rejoining enzymes (34)<br>26. C-type lectin-like (67)<br>27. Nucleotidyltransferase (30)<br>28. Class II aaRS and biotin synthetases (44)
	multidomain; e	1. Beta-lactamase/transpeptidase-like (42)<br>2. DNA/RNA polymerases (84)

Supplementary Table S4. Details about our PSN sets belonging to the third-level hierarchical categories of CATH and SCOP. At the second-level of the CATH hierarchy, there are nine categories: 1.10, 1.20, 2.160, 2.30, 2.40, 2.60, 3.10, 3.30, and 3.40. At the second-level of the SCOP hierarchy, there are six categories: *a*.118, *b*.1, *c*.1, *c*.23, *c*.26 and *c*.55. Each second-level category has multiple third-level categories, as shown in the table. For example, the 2.60 second-level hierarchical category of CATH has two third-level categories: Jelly-rolls and Immunoglobulin-like. For each second-level hierarchical category, we specify its name and label (separated by semicolon), where the labels are as given by CATH/SCOP. For each third-level hierarchical category, we specify its name and the number of PSNs (shown in parentheses).

	**Second-level hierarchical categories**	**Third-level hierarchical categories**
**CATH**	Orthogonal Bundle; 1.10	1. Endonuclease III; domain 1 (38)<br>2. Tetracycline Repressor; domain 2 (69)<br>3. Actin-binding protein, T-fimbrin; domain 1 (46)<br>4. Recoverin; domain 1 (58)<br>5. Cytochrome Bc1 Complex; Chain D, domain 2 (47)<br>6. DNA polymerase; domain 1 (65)<br>7. Tetracycline Repressor; domain 2 (69)<br>8. Retenoid X Receptor (51)<br>9. Arc Repressor Mutant, subunit A (97)<br>10. Globin-like (123)<br>11. Cytochrome p450 (42)<br>12. Lysozyme (33)
	Up-down Bundle; 1.20	1. Glutathoine S-transferase Yfyf (Class Pi); chain A, domain 2 (76)<br>2. Butyryl-CoA Dehydrogenase, subunit A; domain 3 (45)<br>3. Fumarase C; chain A, domain 2 (30)<br>4. Methane Monooxygenase Hydroxylase; chain G, domain 1 (56)<br>5. Ferritin (61)<br>6. Four Helix Bundle (120)<br>7. Phospholipase A2 (46)<br>8. Growth hormone; chain A (42)
	3 Solenoid; 2.160	1. UDP N-Acetylglucosamine Acyltransferase; domain 1 (34)<br>2. Pectate Lyase C-like (36)
	Roll; 2.30	1. SH3 type barrels (33)<br>2. Pdz3 Domain (54)<br>3. PH-domain like (70)<br>4. Pnp Oxidase; chain A (46)
	Beta Barrel; 2.40	1. Thrombin, subunit H (123)<br>2. Porin (31)<br>3. Elongation factor Tu; domain 3 (36)<br>4. Lipocalin (102)<br>5.Cyclophilin (32)<br>6. Cathepsin D; subunit A, domain 1 (81)<br>7. OB fold (125)
	Sandwich; 2.60	1. Jelly rolls (507)<br>2. Immunoglobulin-like (932)
	Roll; 3.10	1. Mannose-binding protein A; chain A (75)<br>2. Ubiquitin Conjugating enzyme (39)<br>3. Thiol ester dehydrase; chain A (55)<br>4. Ubiquitin-like (69)<br>5. Endonuclease I-crel (42)<br>6. Nuclear transport factor 2; chain A (85)<br>7. 2-3 Dihydroxybiphenyl 1,2-Dioxygenase; domain 1 (68)
	2-Layer sandwich; 3.30	1. 60s Ribosomal protein L30; chain A (90)<br>2. Ribosomal protein S5; domain 2 (48)<br>3. GMP synthetase; chain A, domain 3 (31)<br>4. Dihydrodipicolinate Reductase; domain 2 (69)<br>5. Enolase-like; domain 1 (93)<br>6. Nucleotidytransferase; domain 5 (177)<br>7. Beta-Lactamase (76)<br>8. Beta polymerase; domain 2 (45)<br>9. D-amino acid aminotransferase; chain A, domain 1 (62)<br>10. SHC adaptor protein (52)<br>11. Alpha-D-glucose-1,6-bisphosphate; chain A, domain 1 (30)<br>12. Heat shock protein 90 (45)<br>13. Alpha-Beta plaits (239)<br>14. Enolase-like; domain 1 (53)
	2-Layer(aba) Sandwich; 3.40	1. Glutaredoxin (154)<br>2. Peroxisomal Thiolase; chain A, domain 1 (71)<br>3. Rossmann fold (412)
**SCOP**	Alph-alpha superhelix; a.118	1. ARM repeat (37)<br>2. TPR-like (32)
	Immunoglobulin-like beta-sandwich; b.1	1. Fibronectin like III (55)<br>2. E-set domains (73)<br>3. Immunoglobulin (304)
	TIM beta/alpha-barrel; c.1	1. (Trans)glycosidases (160)<br>2. Adolase (54)<br>3. Ribulose-phosphate binding barrel (36)<br>4. Metallo-dependent hydrolases (49)

Supplementary Table S3 – continued on next page

Supplementary Table S3 – continued from previous page

	Second-level hierarchical categories	Third-level hierarchical categories
**SCOP**	Flavodoxin-like; c.23	1. CheY-like (41)<br>2. Class-1 glutamine amidotransferase-like (35)<br>3. Flavoproteins (32)
	Adenine nucleotide alpha hydrolase-like; c.26	1. Nucleotidyl transferase (62)<br>2. Adenine nucleotide alpha hydrolase-like (31)
	Ribonuclease H-like motif; c.55	1. Actin-like ATPase domain (88)<br>2. Ribonuclease H-like (92)

Supplementary Table S5. Details about our PSN sets belonging to the fourth-level hierarchical categories of CATH and SCOP. At the third-level CATH hierarchy, there are six categories: 2.60.120, 2.60.40, 3.20.20, 3.30.390, 3.30.420, and 3.40.50. At the third-level SCOP hierarchy, there are four categories: *b*.1.1, *c*.1.8, *c*.2.1, and *c*.37.1. Each third-level category has multiple fourth-level categories, as shown in the table. For example, the 3.40.50 third-level hierarchical category of CATH has two fourth-level categories: Vaccinia virus protein VP39 and P-loop containing nucleotide triphosphate hydrolase. For each third-level hierarchical category, we specify its name and label (separated by semicolon), where the labels are as given by CATH/SCOP. For each fourth-level hierarchical category, we specify its name and the number of PSNs (shown in parentheses).

	**Third-level hierarchical categories**	**Fourth-level hierarchical categories**
**CATH**	Jelly rolls; 2.60.120	1. Not yet named (71)<br>2. Jelly rolls (112)<br>3. Not yet named (106)<br>4. Galactose-binding domain-like (82)
	Immunoglobulin-like; 2.60.40	1. C2-domain Calcium/lipid binding domain (36)<br>2. Cupredoxins-blue copper proteins (102)<br>3. Immunoglobulins (501)
	TIM barrel; 3.20.20	1. NADP-dependent oxidoreduxtase (39)<br>2. Aldolase class I (267)<br>3. Glycosidases (184)<br>4. Enolase superfamily (67)<br>5. Metal-dependent hydrolases (58)
	Enolase-like, domain 1; 3.30.390	1. Not yet named (30)<br>2. Enolase-like; N-terminal domain (58)
	Nucleotidyltransferase, domain 5; 3.30.420	1. Not yet named (93)<br>2. Not yet named (53)
	Rossmann fold; 3.40.50	1. Vaccinia virus protein VP39 (175)<br>2. P-loop containing nucleotide triphosphate hydrolase (115)
**SCOP**	Immunoglobulin; b.1.1	1. C1 set domains (antibody variable domain-like) (81)<br>2. V set domains (antibody variable domain-like) (200)
	(Trans)glycosidases; c.1.8	1. Beta-glycanases (53)<br>2. Amylase, catalytic domain (55)
	NAD(P)-binding Rossmann-fol domain; c.2.1	1. LDH-N-terminal domain-like (30)<br>2. Glyceraldehyde-3-phosphate dehydrogenase-like, N-terminal domain (45)<br>3. Alcohol dehydrogenase-like, C-terminal domain (30)<br>4. Tyrosine-dependent oxidoreduxtases (110)
	P-loop containing nucleoside triphosphate hydrolase; c.37.1	1. Nucleotide and nucleoside kinases (48)<br>2. Nitrogenase iron protein-like (30)<br>3. Extended AAA-ATPase domain (40)<br>4. G proteins (111)<br>5. ABC transporter ATPase domain-like (33)<br>6. Tandem AAA-ATPase domain (63)

Supplementary Table S6. Accuracy with respect to AUPR values (expressed as percentages) on synthetic networks. Results for non-normalized approaches are highlighted in 1) light gray for network data of the same size and 2) dark gray for network data of different sizes. Results for normalized approaches are not highlighted. Given a network data set (within a column), the AUPR of the best approach is shown in bold. For equivalent results with respect to AUROC values, see Supplementary Table S7.

Approach	Synthetic			
	Synthetic-100	Synthetic-500	Synthetic-1000	Synthetic-All
Graphlet-3-4	**100.00**	**100.00**	**100.00**	81.76
Graphlet-3-5	**100.00**	**100.00**	**100.00**	83.28
NormGraphlet-3-4	**100.00**	**100.00**	**100.00**	94.37
NormGraphlet-3-5	**100.00**	**100.00**	**100.00**	**99.86**
GDDA	97.36	**100.00**	99.99	91.46
RGFD	**100.00**	**100.00**	**100.00**	98.55
GCD	89.26	**100.00**	**100.00**	86.27
Average degree	79.76	79.76	79.76	68.77
Average distance	82.47	98.12	99.60	57.10
Maximum distance	68.82	84.32	93.08	46.11
Average closeness centrality	86.10	88.46	85.33	48.41
Average clustering coefficient	98.93	99.68	99.25	79.37
Intra-hub connectivity	70.88	69.11	69.31	66.61
Assortativity	82.79	92.27	91.73	81.98
Existing-all	**100.00**	**100.00**	**100.00**	85.92

Supplementary Table S7. Accuracy with respect to AUROC values (expressed as percentages) on synthetic networks. Results for non-normalized approaches are highlighted in 1) light gray for network data of the same size and 2) dark gray for network data of different sizes. Results for normalized approaches are not highlighted. Given a network data set (within a column), the AUROC of the best approach is shown in bold. For equivalent results with respect to AUPR values, see Supplementary Table S6.

Approach	Synthetic			
	Synthetic-100	Synthetic-500	Synthetic-1000	Synthetic-All
Graphlet-3-4	**100.00**	**100.00**	**100.00**	82.58
Graphlet-3-5	**100.00**	**100.00**	**100.00**	86.43
NormGraphlet-3-4	**100.00**	**100.00**	**100.00**	97.39
NormGraphlet-3-5	**100.00**	**100.00**	**100.00**	**99.93**
GDDA	98.53	**100.00**	**100.00**	91.73
RGFD	**100.00**	**100.00**	**100.00**	99.21
GCD	89.88	**100.00**	**100.00**	87.89
Average degree	83.33	83.33	83.33	72.14
Average distance	90.28	98.61	99.70	69.66
Maximum distance	79.89	90.63	95.04	54.88
Average closeness centrality	87.80	84.24	80.89	54.84
Average clustering coefficient	99.39	99.81	99.48	88.91
Intra-hub connectivity	79.02	78.22	78.31	71.19
Assortativity	92.75	95.36	95.37	91.61
Existing-all	**100.00**	**100.00**	**100.00**	92.50

Supplementary Table S8. Accuracy with respect to AUPR values (expressed as percentages) on the three real-world PSN sets that form the "equal size" group, each of which contains networks of the same size. Also, average accuracy over all three PSN sets is shown ("Average"), along with the corresponding standard deviation ("SD"). Results for non-normalized approaches are highlighted in light gray. Results for normalized approaches are not highlighted. Given a PSN set (within a given column), the AUPR of the best approach is shown in bold. For equivalent results with respect to AUROC values, see Supplementary Table S9.

Approach	"Equal size" PSN sets			
	CATH-95	CATH-99	CATH-251-265	Average (SD)
Graphlet-3-4	93.31	92.05	98.77	94.71 (3.57)
Graphlet-3-5	89.67	92.78	**100.00**	94.15 (5.29)
NormGraphlet-3-4	96.03	**100.00**	95.28	97.1 (2.54)
NormGraphlet-3-5	94.11	99.73	97.67	97.17 (2.84)
OrderedGraphlet-3	90.99	95.93	**100.00**	95.64 (4.51)
OrderedGraphlet-3-4	96.69	97.13	99.88	**97.90 (1.73)**
NormOrderedGraphlet-3	91.53	98.9	94.49	94.97 (3.71)
NormOrderedGraphlet-3-4	**97.59**	96.73	98.74	97.68 (1.00)
NormOrderedGraphlet-3-4(K)	**97.59**	96.73	98.74	97.68 (1.00)
GDDA	80.21	80.78	71.46	77.48 (5.22)
RGFD	87.87	89.49	94.00	90.45 (3.18)
GCD	75.89	74.92	77.23	76.01 (1.16)
GR-Align	76.25	65.03	70.25	70.51 (5.61)
Average degree	80.47	86.91	85.57	84.32 (3.40)
Average distance	72.90	86.54	51.60	70.35 (17.60)
Maximum distance	62.86	73.49	54.89	63.75 (9.33)
Average closeness centrality	73.12	85.88	49.37	69.46 (18.53)
Average clustering coefficient	87.01	81.21	89.96	86.06 (4.45)
Intra-hub connectivity	70.24	84.24	63.76	72.75 (10.47)
Assortativity	79.94	85.34	93.31	86.20 (6.73)
Existing-all	88.80	96.32	92.48	92.53 (3.76)
DaliLite	53.38	69.12	58.96	60.49 (7.98)
TM-align	50.93	62.02	45.79	52.91 (8.29)
AAComposition	70.23	62.14	54.48	62.28 (7.88)

Supplementary Table S9. Accuracy with respect to AUROC values (expressed as percentages) on the three real-world PSN sets that form the "equal size" group, each of which contains networks of the same size. Also, average accuracy over all three PSN sets is shown ("Average"), along with the corresponding standard deviation ("SD"). Results for non-normalized approaches are highlighted in light gray. Results for normalized approaches are not highlighted. Given a PSN set (within a given column), the AUROC of the best approach is shown in bold. For equivalent results with respect to AUPR values, see Supplementary Table S8.

Approach	CATH of the same size			
	CATH-95	CATH-99	CATH-251-265	Average (SD)
Graphlet-3-4	93.629	92.55	98.80	94.99 (3.34)
Graphlet-3-5	91.97	92.65	**100.00**	94.87 (4.45)
NormGraphlet-3-4	96.48	**100.00**	94.35	96.94 (2.85)
NormGraphlet-3-5	94.114	99.73	97.83	97.22 (2.86)
OrderedGraphlet-3	91.49	96	**100.00**	95.83 (4.26)
OrderedGraphlet-3-4	96.69	97.50	99.89	**98.03 (1.66)**
NormOrderedGraphlet-3	89.69	99.04	94.76	94.49 (4.68)
NormOrderedGraphlet-3-4	**97.51**	97.31	98.72	97.84 (0.76)
NormOrderedGraphlet-3-4(K)	**97.51**	97.31	98.72	97.84 (0.76)
GDDA	80.62	79.33	68.98	76.31 (6.38)
RGFD	85.65	88.45	93.43	89.18 (3.94)
GCD	73.9	73.88	78.67	75.48 (2.76)
GR-Align	71.14	60.49	66.03	65.89 (5.33)
Average degree	85.36	88.99	84.71	86.35 (2.31)
Average distance	73.45	83.79	55.33	70.86 (14.41)
Maximum distance	60.39	71.80	59.45	63.88 (6.88)
Average closeness centrality	74.93	82.73	53.69	70.45 (15.03)
Average clustering coefficient	86.98	85.15	88.30	86.81 (1.58)
Intra-hub connectivity	73.98	86.52	64.88	75.13 (10.87)
Assortativity	85.48	90.19	94.79	90.15 (4.66)
Existing-all	90.22	96.41	92.73	93.12 (3.11)
DaliLite	62.74	71.62	62.13	65.16 (5.65)
TM-align	50.73	65.03	47.84	54.53 (9.20)
AAComposition	69.38	64.12	58.42	63.97 (5.48)

Supplementary Table S10. Summary of method accuracy and running times. Accuracy of the given approach is shown with respect to its average ranking as well as its average raw score compared to all considered approaches across all 35 different-size PSN sets, and the results are shown based on AUPR as well as AUROC. We rank the approaches as follows. For the given PSN set, we determine which approach results in the highest accuracy (rank 1), the second highest accuracy (rank 2), etc. Then, we average the rankings of the given method over all PSN sets. So, the lower the average rank, the better the method. Since NormOrderedGraphlet-3-4(K) has the best average rank with respect to both AUPR and AUROC (shown in bold), we compute the statistical significance of the improvement of NormOrderedGraphlet-3-4(K) over each of the other approaches in terms of their ranks using paired t-test. We also do the same in terms of raw AUPR/AUROC values. Note that in the case of raw values, the higher the average AUPR/AUROC value, the better the approach. Running times of the approaches are shown when comparing proteins from the CATH-α set. Running times for the other data sets are qualitatively the same.

	Rank-based				Raw score-based				
Approach	AUPR		AUROC		AUPR		AUROC		Running
	Avg rank	*p*-value	Avg rank	*p*-value	Avg score	*p*-value	Avg score	*p*-value	time (hrs)
Graphlet-3-4	10.23	2.91e-14	12.74	9.50e-16	51.18	2.84e-11	64.84	5.42e-13	0.43
Graphlet-3-5	12.43	6.64e-17	13.00	1.29e-16	49.23	3.13e-12	64.69	5.56e-13	0.49
NormGraphlet-3-4	10.97	8.55e-20	10.09	1.29e-16	50.73	3.16e-12	67.12	1.47e-13	0.44
NormGraphlet-3-5	10.29	1.57e-17	9.28	4.60e-15	51.24	4.34e-12	67.56	3.87e-13	0.51
OrderedGraphlet-3	9.43	3.17e-10	11.71	1.10e-12	52.71	6.21e-11	65.59	1.96e-12	0.38
OrderedGraphlet-3-4	9.6	2.12e-11	10.06	5.32e-11	53.15	3.16e-12	67.52	6.29e-13	2.39
NormOrderedGraphlet-3	10.03	2.16e-12	9.26	2.15e-10	51.73	3.49e-12	68.86	3.64e-12	0.39
NormOrderedGraphlet-3-4	3.77	1.72e-08	4.66	1.32e-06	64.03	1.34e-09	75.99	7.65e-09	2.41
NormOrderedGraphlet-3-4(K)	**1.37**	-	**1.63**	-	**71.87**	-	**81.97**	-	2.41
GDDA	16.17	1.15e-16	17.31	1.79e-16	44.68	9.62e-13	58.51	2.19e-14	0.54
RGFD	11.26	1.99e-15	11.74	1.34e-13	50.02	7.93e-12	66.01	2.83e-12	0.49
GCD	15.66	6.04e-16	15.57	1.10e-13	45.67	2.06e-12	61.68	4.92e-14	1.32
GR-Align	4.57	2.64e-04	6.57	8.86e-06	64.40	1.10e-05	73.02	1.56e-07	9.49
Average degree	18.83	3.24e-20	16.14	1.38e-15	42.64	2.78e-13	61.26	5.44e-14	0.39
Average distance	17.46	3.97e-19	16.86	5.50e-17	43.63	3.05e-13	61.08	2.04e-14	0.48
Maximum distance	15.89	5.02e-19	14.94	1.18e-16	46.04	1.24e-12	63.36	8.21e-13	0.49
Average closeness centrality	16.14	9.44e-19	15.46	1.82e-15	45.24	1.13e-12	62.49	1.33e-11	0.48
Average clustering coefficient	18.63	1.35e-22	15.63	6.21e-16	43.11	2.64e-13	62.02	1.08e-13	0.56
Intra-hub connectivity	14.23	4.09e-13	15.71	6.94e-17	47.01	2.17e-10	62.32	8.94e-12	0.64
Assortativity	20.91	1.65e-24	18.97	4.51e-18	40.22	7.98e-14	57.88	1.06e-15	0.46
Existing-all	10.31	7.64e-16	9.71	1.62e-12	51.10	8.50e-11	67.59	4.04e-12	1.01
DaliLite	9.20	9.99e-08	6.29	1.02e-04	54.36	8.73e-08	73.73	2.22e-04	2021.41
TM-align	18.37	1.16e-16	19.97	3.06e-20	43.72	5.32e-13	57.18	1.43e-16	168.32
AAComposition	12.97	3.80e-13	14.54	4.55e-15	48.58	5.99e-13	63.31	9.92e-15	0.24

Supplementary Table S11. Detailed accuracy results for each PC approach, each PSN set, and each PSN construction strategy, with respect to AUPR values.

http://nd.edu/~cone/PSN/ST11.xlsx

Supplementary Table S12. Detailed accuracy results for each PC approach, each PSN set, and each PSN construction strategy, with respect to AUROC values.

http://nd.edu/~cone/PSN/ST12.xlsx

Supplementary Table S13. Accuracy of the NormOrderedGraphlet-3-4(K) approach when varying the value of K, with respect to **AUPR values** (expressed as percentages), corresponding to the PSN sets of **"equal size", group 1, and group 2**. Given a PSN set (within a given column), the AUPR for the "best" K is shown in bold. These results are with respect to the first PSN construction strategy, **(any heavy atom type, 4 Å distance cut-off)**.

K value	"Equal size" PSN sets			CATH group 1 and group 2 PSN sets				SCOP group 1 and group 2 PSN sets					
	CATH-95	CATH-99	CATH-251-265	primary	α	β	α/β	primary	α	β	α/β	$\alpha+\beta$	Multi domain
1	**97.5907**	**96.5771**	**98.7353**	**65.6516**	53.0728	**49.3343**	44.3477	**44.5069**	21.7461	26.2314	16.6646	19.9182	72.695
2	95.2756	87.5503	95.4586	62.7671	53.0854	44.8639	42.8751	40.3947	23.3953	22.0232	16.4849	20.4017	71.813
3	88.8915	93.2875	95.4625	63.0474	**53.4148**	42.8472	40.944	40.1442	25.3495	24.3654	17.0831	23.8401	70.5777
4	82.6216	89.9151	87.9388	57.7995	53.387	41.8727	41.9876	38.2845	**31.6257**	26.0871	21.4571	27.443	72.0587
5	81.9265	80.9028	70.0715	46.0584	51.2881	41.9493	42.2158	34.8211	22.9261	28.5727	27.1045	32.5745	80.15
6	86.2847	84.4491	74.8161	45.958	50.8029	42.082	42.3182	34.6656	22.7179	28.6674	28.3451	33.8087	81.1718
7	86.0194	87.1074	75.9893	46.0629	50.4549	41.9585	42.4536	34.3967	22.0997	28.9025	29.3658	**33.8359**	80.7989
8	86.7051	87.0867	79.6482	46.2146	50.3169	41.8143	42.6578	34.2873	21.7503	28.7242	30.1709	33.5889	81.3369
9	85.8064	90.7948	77.2424	46.367	50.2707	41.5483	42.6963	34.2836	21.601	28.2805	30.8011	32.3956	**82.0152**
10	87.2977	91.0244	79.729	46.1782	50.1931	41.2059	43.2325	34.4044	21.1446	28.1126	31.5902	31.9091	80.5146
15	90.0798	88.9304	84.481	44.7598	50.0598	39.8044	46.1777	35.4319	17.9958	22.9517	**34.3508**	25.923	76.7302
20	85.0209	77.1504	84.173	44.0788	49.8056	40.6313	**48.3086**	36.343	16.0554	24.9723	34.1548	23.0386	70.9673
25	76.7759	68.7322	70.2256	42.1777	49.8105	40.1944	45.1245	33.9106	16.061	24.3791	26.2348	22.3209	69.4278
30	68.4945	72.9198	66.7278	40.1754	49.9376	39.9909	40.3299	30.1401	14.8808	27.3717	18.2031	19.2868	71.8487
35	72.8877	72.551	72.6056	39.1643	49.7902	42.4063	37.5206	28.5898	14.2925	**38.8313**	15.0003	16.8235	74.4817

Supplementary Table S14. Accuracy of the NormOrderedGraphlet-3-4(K) approach when varying the value of K, with respect to **AUROC values** (expressed as percentages), corresponding to the PSN sets of **"equal size", group 1, and group 2**. Given a network data set (within a given column), the AUROC for the "best" K is shown in bold. These results are with respect to the first PSN construction strategy, **(any heavy atom type, 4 Å distance cut-off)**.

K value	"Equal size" PSN sets			CATH group 1 and group 2 PSN sets				SCOP group 1 and group 2 PSN sets					
	CATH-95	CATH-99	CATH-251-265	primary	α	β	α/β	primary	α	β	α/β	$\alpha+\beta$	Multi domain
1	**97.5063**	**96.5474**	**98.719**	**76.7992**	53.6152	**65.8059**	62.2703	**70.1481**	70.1844	72.4759	66.1797	75.8884	65.8822
2	95.4861	88.5529	95.5723	74.1997	53.7436	62.6332	60.8591	66.5967	71.1121	68.0998	66.2109	75.8605	63.8069
3	90.5303	92.8343	95.1267	74.5916	**54.6141**	59.4459	59.8402	66.38	72.2224	68.1506	67.6919	78.4727	62.4508
4	83.4701	89.7681	88.1649	71.0703	54.5974	58.17	60.3451	64.7223	**76.5624**	69.6688	73.1774	81.2198	63.4953
5	83.0387	82.7901	71.2058	61.5761	53.1395	58.0683	60.4218	61.3279	68.8734	71.7692	76.8098	83.4135	74.4544
6	86.8161	85.3355	74.826	61.4999	52.536	58.0416	60.5189	61.1776	68.6371	71.2808	77.3252	**83.7493**	75.8451
7	86.9581	88.1664	77.0259	61.6935	52.1318	57.8011	60.5151	60.9443	68.9523	71.297	77.7241	83.3214	75.4427
8	88.3733	87.7408	80.1448	61.8951	51.8638	57.6244	60.628	60.855	69.289	71.0992	78.1114	83.1375	76.1182
9	87.2106	90.4766	78.279	62.0832	51.7987	57.1752	60.5787	60.8541	69.6458	70.6048	78.3253	82.5695	**77.1106**
10	87.7736	90.3646	80.0334	61.8661	51.6322	56.5845	60.9359	60.9248	69.2482	70.0239	78.4249	81.8158	75.4035
15	91.0511	89.4741	84.9624	60.1361	51.3839	55.6159	63.1589	61.6798	67.3932	65.9048	**78.7761**	78.5889	71.3872
20	86.7582	77.3269	84.7396	59.1382	51.6232	56.1634	**65.3814**	62.6331	65.3899	66.6936	77.7261	75.6472	65.1717
25	79.1193	71.5082	72.8209	57.8993	51.5256	55.7813	63.7101	60.598	65.6534	65.4956	70.7485	74.8885	62.0886
30	72.6904	77.4502	64.7173	56.4285	51.6013	55.3446	60.4836	56.8655	65.5548	67.2713	64.6058	73.3497	65.6116
35	73.1534	73.1043	71.8184	55.4317	51.5061	55.909	58.1067	55.1192	65.2114	**76.1271**	62.6461	72.2011	68.721

Supplementary Table S15. Accuracy of the NormOrderedGraphlet-3-4(K) approach when varying the value of K, with respect to **AUPR values** (expressed as percentages), corresponding to the PSN sets of **group 3**. Given a PSN data set (within a given column), the AUPR for the “best” K is shown in bold. These results are with respect to the first PSN construction strategy, **(any heavy atom type, 4 Å distance cut-off)**.

K	CATH (group 3 PSN sets)									SCOP (group 3 PSN sets)					
	1.10	1.20	2.30	2.40	2.60	2.160	3.10	3.30	3.40	a.118	b.1	c.1	c.23	c.26	c.55
1	**61.6497**	37.3066	55.8405	41.3778	66.3825	90.5255	53.6395	31.6033	59.5785	69.1122	61.6951	**55.4437**	65.9708	61.9618	**69.9541**
2	61.1347	38.7983	59.1252	39.3218	66.4699	92.3749	52.8605	29.8561	60.9406	72.2417	60.0198	49.4282	59.9673	61.7931	69.5016
3	57.7577	38.8244	65.4813	41.5462	70.5958	**93.5816**	60.1352	31.7617	62.9763	75.1294	64.6573	47.1585	56.8235	65.0498	69.3076
4	54.153	**44.2358**	66.3363	43.4598	72.1159	89.0275	61.8899	36.2384	64.8037	**77.9167**	65.4388	48.5055	61.3643	64.6248	66.5923
5	39.2762	31.717	**67.1861**	44.5438	73.2692	86.9324	67.8142	40.4848	64.5877	73.5331	65.5757	50.6558	71.4121	65.5377	65.8091
6	42.9408	31.8378	66.2358	**44.747**	73.1501	86.7412	69.9871	40.6044	65.4958	74.0479	65.9237	52.367	**73.4215**	**66.4273**	67.0529
7	44.6416	31.686	66.2895	44.53	73.1275	84.2336	**71.1725**	**41.429**	64.8618	72.0664	**66.0931**	52.6928	72.1031	64.8639	66.7736
8	47.2659	30.7835	65.1814	44.1375	73.2808	82.7926	70.7415	40.9149	65.2843	71.1136	65.6392	53.188	72.0753	64.3293	66.4107
9	46.1363	30.1821	66.1842	43.7361	**73.3936**	81.5877	69.4965	40.5886	65.8174	71.4964	65.549	52.9555	70.1742	63.8262	66.5227
10	46.2111	30.705	64.8469	42.3354	73.3931	81.9588	70.2329	39.6051	66.861	71.8999	65.6117	52.9819	68.4943	62.9939	67.3752
15	42.9211	33.594	58.608	42.4207	68.9495	77.2118	63.4317	35.5546	70.5138	65.2419	62.345	52.8172	63.4918	59.5013	68.6646
20	27.5774	34.1977	55.1215	41.1606	66.7431	71.3578	54.7203	33.3406	**71.9807**	62.15	61.598	51.5218	62.4108	57.7799	64.8636
25	25.1013	31.1811	47.6866	40.1425	66.1568	72.327	52.8544	29.0502	68.6304	59.7452	62.6812	47.5469	58.5513	57.8235	60.757
30	23.2269	28.6583	42.9651	36.4888	65.1921	71.4899	51.6955	26.3731	61.3078	58.8246	63.5856	50.0953	58.4472	61.4041	59.7995
35	24.1706	28.2333	44.559	34.3772	71.0748	63.3739	47.8531	24.5838	55.3463	55.3327	65.7722	53.3428	54.3796	56.3274	58.6811

Supplementary Table S16. Accuracy of the NormOrderedGraphlet-3-4(K) approach when varying the value of K, with respect to **AUROC values** (expressed as percentages), corresponding to the PSN sets of **group 3**. Given a third-level PSN data set (within a given column), the AUROC for the “best” K is shown in bold. These results are with respect to the first PSN construction strategy, **(any heavy atom type, 4 Å distance cut-off)**.

K	CATH (group 3 PSN sets)									SCOP (group 3 PSN sets)					
	1.10	1.20	2.30	2.40	2.60	2.160	3.10	3.30	3.40	a.118	b.1	c.1	c.23	c.26	c.55
1	87.9064	71.9687	76.0773	71.3318	62.3073	90.8253	83.3425	70.662	59.1063	69.2623	53.8498	**68.8026**	80.4364	55.7677	**70.3527**
2	**87.9479**	72.1152	78.7857	71.3139	62.2314	92.2095	82.4135	70.9781	60.8956	72.8724	52.579	63.4412	75.8963	55.642	69.4618
3	86.7571	71.8331	82.2259	73.9953	66.391	**93.3396**	85.8219	71.9767	63.1886	75.8975	57.443	61.138	71.8574	59.5382	69.1148
4	85.5275	**75.9211**	82.5299	75.7971	68.1558	88.6882	87.4806	73.8058	64.2929	**77.9562**	57.5852	61.2642	74.5441	59.2774	66.1889
5	77.216	67.456	**82.9012**	**76.264**	69.429	86.3614	89.177	76.8114	63.4984	72.3658	57.1149	61.9951	80.9876	60.4773	66.3474
6	78.285	67.3926	82.4013	76.019	69.2595	86.1986	90.4164	76.9983	64.5066	73.8982	57.1728	63.635	**82.0814**	**61.7124**	67.3958
7	78.9492	67.0871	82.1281	75.5805	69.2734	84.0521	90.9096	77.2419	63.8133	71.9434	57.513	63.6872	81.1722	59.3455	66.7769
8	79.5771	66.6671	81.6175	74.6915	69.4873	82.7248	**90.9961**	77.2767	64.3511	71.3426	57.2657	64.211	81.0899	58.5351	66.2163
9	79.2747	66.7389	82.1159	74.1164	69.6417	81.6298	90.5578	**77.383**	64.9591	71.5803	57.4373	63.9026	80.1876	58.2017	66.3382
10	79.4423	66.9476	81.2768	73.0254	**69.8404**	81.8274	90.568	76.5993	66.1675	72.0447	57.6969	63.8682	78.4138	57.2376	67.3639
15	78.2981	69.5398	76.8072	73.0018	65.4872	76.1443	86.4741	73.926	71.1178	63.9432	55.0879	63.7811	75.4877	53.6676	68.6794
20	72.2244	69.3484	73.826	71.9277	62.7087	70.9286	79.2302	71.7272	**73.6788**	61.5369	54.5132	62.6509	73.5873	51.3641	65.056
25	70.2178	67.5331	69.8604	70.88	61.5072	74.3408	78.4901	68.2751	70.5657	58.6903	56.4873	59.8231	71.1257	51.1501	59.4359
30	68.124	66.828	65.5758	68.8937	60.2929	75.6396	79.0114	69.4121	63.955	58.0699	56.1275	63.4792	73.8265	54.3649	58.5781
35	69.5849	66.9865	68.0921	67.0596	65.228	66.7312	79.2407	68.5353	58.5676	54.7865	**60.5836**	67.5628	70.124	50.6713	57.4946

Supplementary Table S17. Accuracy of the NormOrderedGraphlet-3-4(K) approach when varying the value of K, with respect to **AUPR values** (expressed as percentages), corresponding to the PSN sets of **group 4**. Given a fourth-level PSN data set (within a given column), the AUPR for the "best" K is shown in bold. These results are with respect to the first PSN construction strategy, **(any heavy atom type, 4 Å distance cut-off)**.

K	CATH (group 4 PSN sets)						SCOP (group 4 PSN sets)			
	2.60.40	2.60.120	3.20.20	3.40.50	3.30.390	3.30.420	b.1.1	c.1.8	c.2.1	c.37.1
1	74.0129	53.5481	42.0719	67.0583	89.8898	73.0981	89.9496	57.7783	69.1796	39.8701
2	72.3984	47.2372	39.5803	68.162	92.1488	**75.4497**	86.6299	58.8563	70.1417	40.6941
3	73.5762	51.9826	40.1298	73.1309	89.7978	73.7605	88.7342	63.1792	65.0664	42.2417
4	75.2457	56.5999	40.4983	76.9781	87.9975	70.1175	89.6381	66.7726	63.2845	47.0372
5	75.8121	59.0995	40.9274	78.8556	92.3089	67.0177	90.632	72.2999	58.9595	54.7122
6	76.0811	59.0608	42.3467	78.2438	92.1667	67.4768	**91.8197**	73.8813	63.1193	56.1089
7	76.2132	**59.9906**	42.6461	78.6772	92.6649	68.4334	91.3819	75.0938	66.739	57.0584
8	75.9835	58.6083	43.027	77.9667	91.6557	69.1337	90.5482	74.5461	67.908	57.2884
9	75.6654	56.6933	42.8402	77.9436	**92.9615**	69.154	90.0004	74.935	69.9117	56.9172
10	75.2327	54.7984	42.7549	78.5071	92.3534	70.7664	89.8094	76.0051	72.6259	57.0701
15	74.7265	46.9835	43.0315	**79.4679**	91.0645	71.7181	86.3153	78.1429	74.4646	57.1303
20	77.8387	44.1702	42.3096	78.9863	78.8769	70.0945	85.7307	**80.2374**	73.55	59.6178
25	78.6806	43.7084	40.8759	76.0138	77.2618	66.5414	88.5031	79.5795	**79.3343**	**60.1238**
30	78.9468	43.2403	43.2012	69.0823	78.1224	65.2842	81.383	73.591	74.4732	49.5931
35	**87.2965**	44.2277	**45.9117**	64.5867	73.5819	64.6016	84.1403	60.7921	74.3511	36.656

Supplementary Table S18. Accuracy of the NormOrderedGraphlet-3-4(K) approach when varying the value of K, with respect to **AUROC values** (expressed as percentages), corresponding to the PSN sets of **group 4**. Given a fourth-level PSN data set (within a given column), the AUROC for the "best" K is shown in bold. These results are with respect to the first PSN construction strategy, **(any heavy atom type, 4 Å distance cut-off)**.

K	CATH (group 4 PSN sets)						SCOP (group 4 PSN sets)			
	2.60.40	2.60.120	3.20.20	3.40.50	3.30.390	3.30.420	b.1.1	c.1.8	c.2.1	c.37.1
1	58.1132	74.5315	59.3962	65.0205	88.6014	69.1872	85.8934	56.9848	77.1939	68.7057
2	56.9371	69.772	57.8308	66.0915	**91.2418**	**71.0785**	81.0668	57.2255	78.2053	69.2715
3	57.81	73.1268	58.4725	71.981	88.2398	69.1043	83.5544	62.7311	75.3783	70.8371
4	59.6047	75.6386	58.8614	75.4526	86.1592	64.1868	85.2766	67.0123	74.6185	74.1633
5	60.1985	77.4704	59.4521	77.3086	90.3773	62.0909	86.6989	72.8931	69.9824	80.9865
6	60.2806	77.5629	60.8914	76.6154	90.0344	61.9436	**88.2641**	74.7568	73.1214	82.2719
7	60.4866	**78.4103**	61.014	76.9524	90.6204	62.9082	87.6796	76.0552	76.1955	82.7798
8	60.3251	77.1709	61.3108	76.1721	89.3736	63.126	86.5605	75.6122	77.0959	83.1138
9	60.0355	76.0164	61.1259	76.1666	91.0227	63.1855	85.8984	75.9648	80.3217	82.9186
10	59.6049	74.8005	60.9885	76.6529	90.2346	64.7431	85.5801	77.0159	82.9047	83.353
15	59.4108	69.0034	61.288	**77.439**	89.2454	66.9032	80.4538	78.4614	85.0739	83.8948
20	65.0575	66.9816	60.7998	76.7231	74.8711	63.6198	78.8447	**79.9889**	84.3669	84.9866
25	67.2629	66.9292	58.4684	73.7451	74.664	60.9307	83.9559	79.8852	89.3177	**85.756**
30	67.0853	67.3643	62.3348	66.4361	74.9636	59.1785	71.9261	74.1606	90.2976	79.5967
35	**80.8403**	67.885	**65.2304**	60.6393	70.9555	58.9356	78.4228	60.7747	**90.7156**	70.1137

Supplementary Table S19. Accuracy of the NormOrderedGraphlet-3-4(K) approach when varying the value of K, with respect to **AUPR values** (expressed as percentages), corresponding to the PSN sets of **"equal size", group 1, and group 2**. Given a PSN data set (within a given column), the AUPR for the "best" K is shown in bold. These results are with respect to the second PSN construction strategy, **(any heavy atom type, 5 Å distance cut-off)**.

	"Equal size" PSN sets			CATH group 1 and group 2 PSN sets				SCOP group 1 and group 2 PSN sets					
K	CATH-95	CATH-99	CATH-251-265	primary	α	β	α/β	primary	α	β	α/β	α+β	Multi domain
1	**90.1293**	**96.7329**	92.4337	**61.8708**	53.8463	**48.0196**	47.7063	**44.35**	29.2498	33.1333	22.8616	32.2998	78.1976
2	89.6195	95.2273	84.9005	59.3544	53.303	46.7265	47.179	43.0547	31.1435	33.3119	22.3318	33.2543	76.8828
3	85.8699	95.7829	**92.7763**	61.5902	53.1238	45.6588	45.2924	43.2427	32.9887	37.1399	21.9893	37.2948	75.2136
4	76.2696	87.5723	85.7654	55.6374	**53.9913**	45.3545	46.3995	40.4639	**39.3916**	40.4997	27.8103	41.0889	77.0128
5	72.2232	83.5286	73.5438	45.965	52.3566	45.7878	46.0736	36.1553	33.7444	43.4279	32.5696	45.9052	83.6805
6	74.4759	84.4109	67.193	48.2658	51.5463	45.7951	46.1279	37.0148	34.5151	44.5318	36.3742	**48.8451**	88.224
7	77.6668	87.7908	67.1035	48.4988	51.2998	45.6369	45.8423	36.5923	33.16	**45.3129**	37.601	48.691	88.7471
8	80.7965	87.522	67.0638	48.4973	51.0773	45.6949	45.7426	36.2597	32.6432	45.0688	38.4064	48.7131	**89.2435**
9	80.3752	89.2913	68.6071	48.1902	50.8201	45.4121	45.5353	35.9353	33.0695	44.4781	38.7857	47.918	88.6229
10	80.7622	88.6804	67.0252	47.9054	50.7087	45.2699	45.9115	35.7686	32.1983	43.6678	39.9818	46.8986	87.0965
15	82.8087	84.3218	70.3052	45.2898	50.4142	42.8407	47.9474	35.1799	29.5758	32.656	43.1693	40.5544	84.3173
20	80.5605	76.2962	68.5103	44.0739	50.299	43.5713	**49.8881**	36.4337	25.6157	35.5099	**44.1328**	35.5403	76.1873
25	77.3181	68.7135	61.5178	41.7811	50.2084	43.388	47.6185	34.327	25.2414	33.75	36.346	32.4704	70.923
30	71.3141	68.2464	60.9921	39.297	50.1873	42.3812	43.2253	30.1104	23.8993	32.2922	25.4569	28.3639	73.1053
35	65.7073	64.5557	60.2081	37.884	50.2172	43.5731	40.1525	28.1892	22.3197	45.0514	19.8854	24.8853	75.2268

Supplementary Table S20. Accuracy of the NormOrderedGraphlet-3-4(K) approach when varying the value of K, with respect to **AUROC values** (expressed as percentages), corresponding to the PSN sets of **"equal size", group 1, and group 2**. Given a PSN data set (within a given column), the AUROC for the "best" K is shown in bold. These results are with respect to the second PSN construction strategy, **(any heavy atom type, 5 Å distance cut-off)**.

	"Equal size" PSN sets			CATH group 1 and group 2 PSN sets				SCOP group 1 and group 2 PSN sets					
K	CATH-95	CATH-99	CATH-251-265	primary	α	β	α/β	primary	α	β	α/β	α+β	Multi domain
1	**92.1559**	**97.3062**	**93.4559**	**73.4624**	54.2521	**64.3227**	63.8175	**69.6482**	74.0648	75.3711	72.3441	81.7101	69.4508
2	90.4619	95.9313	89.8635	71.2761	53.7283	63.034	62.9988	68.4734	74.5817	76.9205	72.5458	82.5299	67.2366
3	86.0848	95.1865	92.8154	73.2331	53.9075	60.0108	62.3128	68.31	75.8531	77.5661	72.3877	84.6455	65.5069
4	76.8992	88.7881	84.8232	68.9091	**55.0325**	59.004	62.5683	65.8379	**80.7895**	79.7001	77.4867	86.0845	68.0466
5	70.4335	85.4475	73.9348	60.1658	54.0004	59.1786	62.0895	61.4664	76.1225	81.5755	80.0728	87.3183	78.3846
6	73.6637	85.0722	66.0262	63.0276	53.1223	59.0371	61.9005	62.3984	75.068	81.8906	81.2816	**88.394**	84.3454
7	78.5669	87.9648	66.2768	63.2071	52.8895	58.848	61.4987	61.983	74.4767	**81.9161**	81.7972	88.181	85.0481
8	83.528	87.6624	65.9705	63.2372	52.4994	58.8626	61.4004	61.7126	74.9483	81.5017	**82.0041**	88.1123	**85.9252**
9	83.5701	88.8721	66.973	63.038	52.1364	58.525	61.1564	61.4525	75.7291	81.0546	81.8133	88.0375	85.3287
10	84.1698	88.4017	67.7806	62.7383	51.8745	58.1755	61.2736	61.3159	75.6926	80.4262	81.89	87.5313	83.4395
15	85.0011	85.2039	66.3603	60.0823	51.4955	57.1557	62.7371	60.8863	74.9065	72.999	81.7203	84.5089	79.6962
20	83.428	77.0021	65.3857	58.4853	51.9202	57.4631	**64.9767**	61.8427	72.8786	73.4677	80.689	81.6836	70.6637
25	78.5354	71.0181	62.8794	56.6543	51.622	57.3838	64.3919	60.194	71.9344	71.6672	74.4912	80.2369	64.0938
30	75.3262	66.8375	61.7655	54.848	51.6327	56.8225	61.9054	56.2927	71.6975	71.3695	68.0335	77.7805	66.3761
35	67.3085	64.4237	63.6313	53.7939	51.2877	56.1179	60.2608	54.4337	71.3292	77.4008	66.0644	76.3772	69.7125

Supplementary Table S21. Accuracy of the NormOrderedGraphlet-3-4(K) approach when varying the value of K, with respect to **AUPR values** (expressed as percentages), corresponding to the PSN sets of **group 3**. Given a third-level PSN data set (within a given column), the AUPR for the “best” K is shown in bold. These results are with respect to the second PSN construction strategy, **(any heavy atom type, 5 Å distance cut-off)**.

K	CATH (group 3 PSN sets)									SCOP (group 3 PSN sets)					
	1.10	1.20	2.30	2.40	2.60	2.160	3.10	3.30	3.40	a.118	b.1	c.1	c.23	c.26	c.55
1	70.1888	47.8657	70.3681	47.828	73.3026	94.601	64.3766	42.4682	66.8807	72.0039	66.1478	55.8399	70.7324	62.7786	**70.6016**
2	**70.6977**	49.0484	76.4055	49.5749	75.4275	**95.5012**	68.1964	44.1884	66.3608	73.3984	66.5451	52.0822	66.5276	62.0854	68.5837
3	68.1273	52.8204	82.3922	52.8286	77.8616	95.1551	71.3621	43.5402	68.2534	77.3863	72.0065	50.6113	64.69	65.5102	68.6647
4	66.6286	**59.254**	84.6694	54.5257	79.6314	93.1276	77.2766	47.8715	68.4487	76.8486	73.1701	51.1257	65.7376	**65.7142**	65.6009
5	58.5658	49.1199	**85.489**	55.6096	80.6366	91.8032	81.8818	50.6905	67.8352	73.4327	72.5766	51.8513	71.7028	64.8037	65.0926
6	58.6434	50.5092	84.5194	56.4366	80.937	91.9434	83.5079	**52.2111**	68.6687	**80.8925**	73.2142	54.057	**76.8178**	64.2804	66.7878
7	60.5322	50.6914	83.2262	56.5682	81.02	89.7315	**83.9781**	52.0927	68.558	80.6302	73.2984	54.7762	76.1762	63.9446	67.5092
8	61.8906	49.3619	81.236	**57.1834**	81.2453	88.0825	83.2815	51.9634	69.0176	80.3644	73.4212	55.166	76.2531	63.0373	68.2376
9	61.3601	48.6017	81.6801	56.7066	81.4193	86.5099	82.8035	51.6281	69.4408	80.1994	73.3961	55.3225	76.19	63.6811	68.4679
10	62.3744	48.0166	80.9702	55.8827	**81.6573**	84.8987	82.8162	50.5859	70.3467	80.1583	**73.5128**	55.5556	76.4906	62.5436	69.672
15	60.6219	48.9625	75.7437	54.4968	76.7357	78.1878	76.7591	45.3084	74.2734	70.382	69.1947	56.6455	70.8174	61.3799	69.3588
20	46.729	48.7413	74.7478	53.4289	74.0422	75.0448	68.1382	43.6596	**75.2544**	64.0047	69.0965	56.1739	68.486	60.3308	64.7637
25	42.0577	44.6506	63.9787	51.8313	71.7495	74.5992	64.108	38.3799	72.5094	61.1756	70.4049	53.7586	68.3244	59.7777	60.8801
30	38.8251	41.3439	51.5053	48.949	69.7585	71.2794	62.8689	35.0744	66.9182	56.8104	67.2423	57.4644	68.4592	63.4404	60.6074
35	36.9035	40.9553	54.7528	45.4604	73.4811	62.1884	57.8925	31.7169	60.6862	52.1481	69.0252	**60.5649**	69.4463	61.072	61.276

Supplementary Table S22. Accuracy of the NormOrderedGraphlet-3-4(K) approach when varying the value of K, with respect to **AUROC values** (expressed as percentages), corresponding to the PSN sets of **group 3**. Given a third-level PSN data set (within a given column), the AUROC for the “best” K is shown in bold. These results are with respect to the second PSN construction strategy, **(any heavy atom type, 5 Å distance cut-off)**.

K	CATH (group 3 PSN sets)									SCOP (group 3 PSN sets)					
	1.10	1.20	2.30	2.40	2.60	2.160	3.10	3.30	3.40	a.118	b.1	c.1	c.23	c.26	c.55
1	89.9729	77.1599	84.6076	75.322	70.035	94.217	87.6714	75.9683	66.6643	70.3818	56.123	68.0257	83.056	55.3798	**70.5165**
2	**90.0127**	76.495	87.4928	76.4451	72.621	**95.2882**	88.6743	76.8722	65.9341	72.2064	57.9833	64.3704	80.0444	55.7951	67.7572
3	89.4629	77.6302	90.636	79.6179	74.7998	95.023	90.8405	76.8585	67.5907	76.1401	64.3072	62.9341	77.2639	58.4613	66.9407
4	88.7562	**82.9048**	91.8545	80.9303	76.7151	93.1104	92.5375	78.3884	67.1961	76.2392	65.332	62.8916	77.852	59.06	64.0275
5	85.2459	77.3024	**92.5461**	81.2686	77.6998	92.064	93.4237	79.7623	65.3028	74.1751	63.76	62.8722	82.2157	**59.553**	63.876
6	83.838	77.052	92.01	**81.3507**	78.0082	92.1077	94.3204	80.8325	65.9783	80.312	64.2612	63.9343	**86.3713**	58.3682	65.6053
7	84.3395	77.0565	91.4701	80.9709	78.0584	89.5526	**94.5846**	80.9295	65.9316	**80.4693**	64.3841	64.3037	85.7224	57.1569	66.366
8	84.9838	77.1446	90.429	81.028	78.3103	87.9869	94.3644	81.1298	66.2637	80.1642	64.8272	64.3707	85.307	56.4776	67.1123
9	84.9935	77.0521	90.4537	80.8046	78.5432	86.5946	94.136	**81.4463**	66.8804	80.1675	65.4751	64.1919	85.1176	57.2636	67.4526
10	85.3338	76.9249	90.0514	80.0571	**78.8021**	85.0836	94.053	81.0906	67.9563	80.0001	**66.1363**	64.2459	84.9293	55.9299	68.8651
15	85.0128	77.2307	87.0989	78.967	73.7657	77.435	90.2351	78.2846	72.6969	68.5613	61.7839	65.3766	81.2183	54.9503	69.062
20	81.7327	76.3778	85.1317	77.2364	69.5309	73.4921	85.4608	75.0744	**74.6562**	63.1703	61.2053	65.1353	78.4686	53.365	64.143
25	79.8247	73.1182	78.3547	76.6581	66.4971	75.7984	83.7475	72.0773	72.755	59.7832	63.2383	64.1264	79.8979	52.1927	58.7856
30	79.4104	72.7787	71.9534	75.0257	64.5791	74.8023	83.6246	71.8832	68.0134	57.2002	61.2363	69.8803	81.2289	56.6285	57.9063
35	79.9387	72.5298	73.847	72.4848	66.6064	65.3388	82.5317	70.9099	62.9533	53.2405	64.1483	**72.2598**	81.3092	55.4175	59.4918

Supplementary Table S23. Accuracy of the NormOrderedGraphlet-3-4(K) approach when varying the value of K, with respect to **AUPR values** (expressed as percentages), corresponding to the PSN sets of **group 4**. Given a fourth-level PSN data set (within a given column), the AUPR for the "best" K is shown in bold. These results are with respect to the second PSN construction strategy, **(any heavy atom type, 5 Å distance cut-off)**.

K	CATH (group 4 PSN sets)						SCOP (group 4 PSN sets)			
	2.60.40	2.60.120	3.20.20	3.40.50	3.30.390	3.30.420	b.1.1	c.1.8	c.2.1	c.37.1
1	74.9033	66.2649	42.1689	74.2736	93.2583	74.8143	93.6504	71.0191	78.9898	49.125
2	74.9948	64.9926	40.9472	77.34	94.6824	73.2939	92.1548	72.8109	78.6754	51.7017
3	77.9506	65.5812	42.0975	80.0803	95.3137	72.1778	94.7775	71.8968	76.6829	55.9321
4	79.7081	68.1882	42.8928	83.0066	96.4343	67.7385	96.1868	76.1084	72.6883	58.1231
5	79.8533	70.5004	42.7044	83.2421	97.9571	67.8518	96.5378	78.8051	71.3546	58.1717
6	80.4625	71.2236	43.8778	84.4857	99.0126	70.2086	96.8925	81.6035	74.5734	65.7896
7	80.7754	**71.9896**	44.3455	85.1513	99.2929	71.7876	**97.0257**	83.583	78.5694	67.5704
8	80.7397	70.4704	44.4256	85.3767	99.2766	74.3872	96.7908	84.8475	81.4623	67.8088
9	80.5879	68.5018	44.174	86.1002	**99.3276**	75.933	96.0822	**86.8751**	84.109	66.7331
10	80.3312	65.6508	44.2013	86.4784	99.0535	76.6184	95.083	86.3391	86.1268	68.1461
15	79.4145	54.8775	45.5161	**86.725**	97.7053	**76.8103**	90.0741	84.6791	88.5273	67.7888
20	82.8401	50.0895	46.3268	84.7328	95.582	72.7069	88.256	84.743	87.63	70.4865
25	84.1294	49.6145	43.8172	82.1014	88.2795	68.8854	91.0842	84.5114	**91.1862**	**71.3548**
30	81.1752	49.8611	46.1784	75.9178	88.2805	68.1941	81.3324	79.5306	85.0275	60.2713
35	**88.7565**	49.5381	**47.9033**	69.733	84.0834	68.046	83.3324	67.8933	83.6966	43.8514

Supplementary Table S24. Accuracy of the NormOrderedGraphlet-3-4(K) approach when varying the value of K, with respect to **AUROC values** (expressed as percentages), corresponding to the PSN sets of **group 4**. Given a fourth-level PSN data set (within a given column), the AUROC for the "best" K is shown in bold. These results are with respect to the second PSN construction strategy, **(any heavy atom type, 5 Å distance cut-off)**.

K	CATH (group 4 PSN sets)						SCOP (group 4 PSN sets)			
	2.60.40	2.60.120	3.20.20	3.40.50	3.30.390	3.30.420	b.1.1	c.1.8	c.2.1	c.37.1
1	58.8008	82.4139	59.5833	71.5016	92.0205	71.0827	91.185	70.1913	86.1179	72.7638
2	60.1264	81.3671	58.6709	75.2663	93.4667	68.3258	88.5631	72.3502	85.9045	74.7514
3	64.1267	79.8166	59.6279	78.7545	93.9172	66.1151	92.2954	71.7763	83.9021	78.436
4	66.8206	80.8921	60.4946	81.4952	95.2641	61.7746	94.3679	76.0131	81.314	79.7211
5	67.1765	82.9976	60.2252	81.4806	97.4081	61.8939	94.9122	79.2552	79.7896	81.1317
6	67.6505	83.628	61.2001	82.535	98.7691	64.2648	95.4347	81.7727	81.0896	86.1182
7	68.0732	**84.5437**	61.6668	82.936	99.1331	66.2552	**95.5938**	83.6685	84.9956	87.155
8	68.2873	83.6944	61.83	83.0858	99.1031	69.2327	95.2513	84.8491	87.7008	87.1924
9	68.1462	82.607	61.5542	83.9001	**99.1791**	70.6866	94.1204	**87.0056**	90.2834	87.114
10	67.9777	80.6415	61.703	84.2648	98.8114	71.2492	92.5962	86.6156	92.1025	87.8992
15	66.865	72.7715	63.0178	**84.4044**	97.0409	**71.6779**	85.2979	84.7492	94.3329	87.1983
20	71.8673	69.8949	63.6893	82.4023	94.2872	65.8528	82.5724	84.9073	94.2025	88.4179
25	73.583	69.5855	60.5995	79.1058	84.3048	61.7587	86.4893	85.0014	**95.6084**	**88.8489**
30	71.1125	71.0534	64.0941	72.0095	84.6123	60.4806	71.2567	79.8022	94.3146	83.1081
35	**82.4339**	71.2768	**65.9334**	66.2128	80.5164	59.5284	76.3821	67.1088	93.9284	74.0693

Supplementary Table S25. Accuracy of the NormOrderedGraphlet-3-4(K) approach when varying the value of K, with respect to **AUPR values** (expressed as percentages), corresponding to the PSN sets of **"equal size", group 1, and group 2**. Given a PSN data set (within a given column), the AUPR for the "best" K is shown in bold. These results are with respect to the third PSN construction strategy, **(any heavy atom type, 6 Å distance cut-off)**.

	"Equal size" PSN sets			CATH group 1 and group 2 PSN sets				SCOP group 1 and group 2 PSN sets					
K	CATH-95	CATH-99	CATH-251-265	primary	α	β	α/β	primary	α	β	α/β	α+β	Multi domain
1	**93.8243**	**92.8393**	87.0351	59.8257	53.4222	**47.8743**	48.9485	**44.6876**	33.353	36.5249	27.1641	34.2874	78.071
2	93.083	89.6121	90.2352	58.8208	53.0435	47.085	48.5169	43.9354	35.278	36.8596	26.9914	37.5493	77.4191
3	90.8128	92.436	**93.3112**	**60.3527**	52.9737	46.3418	47.5231	42.703	39.5471	40.0912	28.028	40.4788	77.4308
4	80.0816	81.1256	85.3033	54.3881	**53.5014**	46.1865	47.7531	40.2684	**45.7485**	43.3209	33.0808	43.9846	78.2156
5	74.6997	79.6314	81.5785	48.6689	52.5398	46.646	47.9072	38.354	44.6273	46.751	38.9037	48.6853	82.2865
6	72.7505	78.5	62.6273	46.999	51.0541	46.5803	48.0473	37.5606	39.5633	47.844	42.76	**51.54**	85.922
7	75.0194	79.2788	60.3636	47.4455	51.0282	46.615	47.9182	37.5255	39.4813	48.2551	43.9197	51.3459	86.9902
8	78.1553	79.8152	63.1837	47.7116	50.8978	46.657	47.9727	37.5344	39.219	**48.5201**	44.8585	51.1235	**87.5817**
9	80.531	80.8223	61.0374	47.6222	50.6649	46.4533	47.9137	37.3604	39.6423	47.7429	45.6195	50.3756	87.1385
10	83.7978	79.9477	60.9419	47.3671	50.5379	46.1252	48.1997	37.278	38.6598	47.1577	46.99	49.0089	86.3161
15	80.7855	70.8327	64.7825	44.9937	50.3908	43.8084	50.0358	37.3946	35.9479	34.5486	49.3008	41.8156	82.5436
20	73.88	65.8775	63.67	43.5881	50.1897	44.4702	**51.5864**	38.1564	31.999	37.0766	**49.3707**	37.0811	76.3455
25	75.8971	62.8909	60.1876	41.4456	50.4805	44.0248	48.6661	35.1114	30.0962	34.5814	40.2873	34.7357	73.9207
30	65.7328	61.9828	60.6293	39.0491	50.652	43.2755	44.5186	30.4832	27.3895	33.7027	29.2733	30.9804	74.5788
35	64.6727	60.3011	59.7696	37.8675	50.7119	44.5025	41.3703	28.4752	24.758	48.322	23.4522	28.2751	76.2512

Supplementary Table S26. Accuracy of the NormOrderedGraphlet-3-4(K) approach when varying the value of K, with respect to **AUROC values** (expressed as percentages), corresponding to the PSN sets of **"equal size", group 1, and group 2**. Given a PSN data set (within a given column), the AUROC for the "best" K is shown in bold. These results are with respect to the third PSN construction strategy, **(any heavy atom type, 6 Å distance cut-off)**.

	"Equal size" PSN sets			CATH group 1 and group 2 PSN sets				SCOP group 1 and group 2 PSN sets					
K	CATH-95	CATH-99	CATH-251-265	primary	α	β	α/β	primary	α	β	α/β	α+β	Multi domain
1	**94.3234**	**93.8788**	88.6383	71.9276	54.0966	**63.738**	64.8737	**69.7367**	75.7123	77.5732	75.8479	82.013	68.8426
2	94.0394	90.8966	91.6458	70.8497	53.7887	62.8647	64.3847	69.0881	75.9261	78.655	76.6251	83.2263	67.6766
3	91.835	92.6635	**93.8736**	**72.0084**	53.8229	60.7987	63.763	67.6286	78.2847	79.2447	76.68	85.0477	68.1101
4	81.4973	82.7985	84.7675	67.6509	**54.5611**	59.7862	63.6131	65.3938	**82.2827**	81.0785	79.8074	86.0132	70.0571
5	76.0206	81.334	81.8992	62.3552	54.2198	59.8363	63.1911	63.4352	81.3367	83.009	82.3993	86.8997	76.0478
6	73.7216	79.4467	65.6085	61.7028	53.2096	59.521	62.8355	62.5827	77.7728	**83.4339**	83.6866	**88.129**	81.0202
7	76.8992	80.2615	63.9098	62.0611	53.0928	59.4129	62.5519	62.4775	77.8095	83.4099	83.9997	87.968	82.3286
8	81.1237	79.8695	63.5199	62.3779	52.8911	59.3893	62.5011	62.567	78.0957	83.2847	84.2259	87.8036	**83.113**
9	83.5859	80.0319	63.6313	62.3163	52.5505	59.1276	62.4145	62.4463	78.5724	82.8127	**84.233**	87.7296	82.521
10	86.4846	79.4859	63.6591	62.0077	52.2197	58.8071	62.4778	62.3637	78.4301	82.2941	84.2296	87.2141	81.2565
15	81.834	73.3591	61.9048	59.4885	51.8159	58.2913	64.079	62.6077	77.5991	74.7197	83.6212	84.2117	76.4956
20	75.7471	66.087	60.039	57.6426	51.9354	58.3897	**66.2393**	63.229	75.8376	74.5246	82.6652	82.2935	69.4466
25	78.4144	64.0261	57.477	56.1996	51.8835	58.1819	65.2852	60.9291	74.7011	72.5505	76.1802	81.4026	66.2025
30	67.1717	62.864	58.6188	54.4137	51.7009	57.2397	62.8051	56.7944	73.6788	72.2385	69.8266	79.3361	66.7526
35	64.5781	55.8048	60.1504	53.5864	51.3675	56.0372	61.1164	54.7799	73.1983	77.5771	68.8685	77.5165	69.4951

Supplementary Table S27. Accuracy of the NormOrderedGraphlet-3-4(K) approach when varying the value of K, with respect to **AUPR values** (expressed as percentages), corresponding to the PSN sets of **group 3**. Given a third-level PSN data set (within a given column), the AUPR for the "best" K is shown in bold. These results are with respect to the third PSN construction strategy, **(any heavy atom type, 6 Å distance cut-off)**.

K	CATH (group 3 PSN sets)									SCOP (group 3 PSN sets)					
	1.10	1.20	2.30	2.40	2.60	2.160	3.10	3.30	3.40	a.118	b.1	c.1	c.23	c.26	c.55
1	73.3464	51.7107	71.9404	51.7997	74.8095	**88.1348**	69.46	44.3352	68.6623	73.0844	66.6309	49.9587	69.8545	64.9	71.0955
2	72.4505	52.549	76.38	53.7642	76.3355	86.1059	71.8524	46.2004	69.366	74.5029	67.0279	48.6893	67.8266	65.4839	69.5126
3	73.8951	56.2175	82.3302	56.9433	78.4936	85.8195	76.0914	46.7265	70.988	77.3768	71.8415	50.2696	68.1077	67.9796	70.7193
4	**74.8457**	**60.3167**	84.7865	58.3922	79.7607	81.5785	79.5998	49.0952	71.8837	78.3421	73.0356	49.9303	71.3339	68.2132	69.7907
5	73.3909	56.9476	86.4977	58.5572	80.6421	81.1403	83.5227	51.782	72.6383	73.528	73.0066	51.9297	77.0661	**69.8665**	69.3367
6	63.4072	54.4516	**86.5798**	59.0244	81.1062	82.9711	85.0122	**53.4345**	72.848	78.1865	73.4767	54.0276	**81.8192**	66.7403	71.459
7	64.3385	54.7762	86.1548	58.7385	81.1875	86.5017	**85.5274**	53.3159	73.2776	78.1345	73.2922	54.8217	81.5819	66.5758	71.7911
8	65.2086	54.2585	85.3645	**59.2963**	81.2643	84.4888	85.25	53.1767	73.9421	77.5746	73.2169	54.8639	80.9587	66.0262	73.2361
9	64.7207	53.389	84.9986	59.0635	81.3087	81.6493	85.006	52.8294	74.6591	77.5682	73.3925	55.1888	80.3124	65.7048	74.3009
10	65.2603	53.1642	84.0543	57.9443	**81.3156**	80.4183	84.3116	51.8442	75.1203	**78.8409**	73.4419	54.8851	79.6761	65.3196	76.0515
15	65.5948	53.994	78.3551	59.0749	76.1645	72.1139	78.902	47.0125	**77.0645**	68.123	67.0202	54.9751	71.4143	65.675	**76.867**
20	53.3127	52.2912	76.1787	56.4339	73.2966	72.1231	72.4788	45.0718	75.9707	64.1886	68.9241	54.2924	70.1878	63.6064	70.6776
25	47.0291	48.9395	66.6881	54.4938	71.5527	71.6799	69.9541	40.0075	71.0925	64.8172	70.8678	54.4296	70.3954	61.7495	64.0474
30	48.0621	44.1294	58.7377	52.1793	70.0379	68.4914	67.5603	36.5691	66.2675	60.9841	68.9509	61.5459	68.4738	65.9126	59.6724
35	47.08	44.5991	57.337	50.3339	74.415	61.7766	62.3279	33.2249	60.4583	58.8941	**73.5016**	**63.6018**	66.5243	61.3145	59.5543

Supplementary Table S28. Accuracy of the NormOrderedGraphlet-3-4(K) approach when varying the value of K, with respect to **AUROC values** (expressed as percentages), corresponding to the PSN sets of **group 3**. Given a third-level PSN data set (within a given column), the AUROC for the "best" K is shown in bold. These results are with respect to the third PSN construction strategy, **(any heavy atom type, 6 Å distance cut-off)**.

K	CATH (group 3 PSN sets)									SCOP (group 3 PSN sets)					
	1.10	1.20	2.30	2.40	2.60	2.160	3.10	3.30	3.40	a.118	b.1	c.1	c.23	c.26	c.55
1	90.3512	78.1976	85.4841	75.7795	71.6692	**86.9358**	89.7475	76.8867	66.6324	72.923	55.5962	61.383	80.0345	59.7924	70.187
2	90.2306	77.8876	88.0409	77.126	73.5867	84.9228	90.4166	78.1152	67.2896	74.6517	57.2989	59.8272	78.5558	60.5923	67.9548
3	**90.8662**	79.3433	90.9974	79.5513	75.5564	84.8777	92.1119	78.3789	68.1351	76.6354	62.6217	60.7446	78.2967	62.7023	68.474
4	90.7976	**82.6673**	92.2943	81.1171	77.001	80.4899	92.8166	79.1032	69.224	77.4464	63.4621	60.0975	80.5057	62.7753	67.7679
5	90.4571	80.2337	**93.2626**	**81.5552**	77.8482	80.1649	93.7317	79.9461	69.3786	73.0753	62.712	62.2665	84.5572	**64.77**	68.1682
6	84.9792	79.263	93.2373	81.3895	78.3381	81.6964	94.622	80.9716	69.336	77.8312	62.7475	63.4894	**88.5832**	61.3577	70.3097
7	85.3743	79.3445	93.0972	80.6127	78.4411	85.3479	**94.897**	81.1894	69.8094	77.7108	62.6773	63.8302	88.1089	60.9438	70.8489
8	85.8789	79.7942	92.7415	80.7929	78.5041	83.2381	94.8292	81.3503	70.4496	77.0165	62.7638	63.694	87.4711	60.2684	72.4464
9	85.8093	79.7576	92.5388	80.4676	**78.5756**	80.5959	94.7764	**81.7169**	71.3149	77.0856	63.1464	63.9097	87.1325	60.4072	73.8337
10	86.2108	79.6715	92.0647	80.0025	78.5635	79.22	94.4495	81.3741	71.9475	**78.2375**	63.9187	63.695	86.4182	59.731	75.7714
15	86.4524	79.3982	88.7265	81.1494	72.8969	70.7611	91.6763	78.5716	**74.472**	66.7698	57.2883	64.9264	79.6166	59.8413	**77.0489**
20	84.0493	77.9498	86.1494	78.5863	68.0633	70.1213	87.4151	76.466	73.4138	64.3417	59.4421	63.9768	77.874	56.9009	69.8104
25	82.4376	75.2028	80.6208	77.0498	65.779	72.0817	86.7011	73.4768	69.0898	64.528	61.9304	66.4029	79.439	54.5309	62.2427
30	83.5784	73.5827	76.2613	76.2916	64.0554	71.1403	86.1663	72.4495	64.8653	61.4233	62.743	74.4789	80.7766	57.4628	57.103
35	85.3346	73.3971	74.6712	74.0204	66.9849	63.9075	84.1	71.475	60.5565	60.7473	**68.0637**	**75.5238**	79.1023	55.7328	57.3212

Supplementary Table S29. Accuracy of the NormOrderedGraphlet-3-4(K) approach when varying the value of K, with respect to **AUPR values** (expressed as percentages), corresponding to the PSN sets of **group 4**. Given a fourth-level PSN data set (within a given column), the AUPR for the "best" K is shown in bold. These results are with respect to the third PSN construction strategy, **(any heavy atom type, 6 Å distance cut-off)**.

K	CATH (group 4 PSN sets)						SCOP (group 4 PSN sets)			
	2.60.40	2.60.120	3.20.20	3.40.50	3.30.390	3.30.420	b.1.1	c.1.8	c.2.1	c.37.1
1	77.9858	64.8291	39.2295	78.9917	85.3935	73.4688	95.1654	73.9114	81.0933	56.4116
2	78.6522	62.6146	39.5297	82.7287	86.4397	72.8405	93.6485	74.7088	80.5665	59.8416
3	80.957	62.6319	41.5066	85.8506	89.5249	73.5117	94.6433	74.9081	79.1726	66.3687
4	81.9884	65.2562	42.0838	88.4677	92.3308	73.123	94.7234	79.1917	79.8349	69.5546
5	82.2424	68.2039	43.9341	88.8578	97.1183	73.8882	95.0068	82.0343	79.8275	72.109
6	82.4941	69.6826	45.296	88.6551	98.5066	77.6157	95.8659	83.9304	80.5696	77.8323
7	82.775	**69.9771**	45.6653	88.6735	98.8062	78.7963	**95.9229**	84.3143	83.3808	79.1121
8	82.834	68.1406	45.4503	**88.9744**	98.788	80.3937	95.6233	85.6794	84.3907	80.092
9	82.8356	66.0073	45.7117	88.9354	**98.8557**	81.0712	95.7139	**86.8204**	85.6773	79.7612
10	82.8436	63.6326	45.4137	88.8957	98.6711	82.9536	95.0836	86.3755	86.5759	**80.5846**
15	80.981	55.6233	46.8515	87.8728	97.4894	**84.1434**	89.9936	83.192	88.7548	79.6738
20	84.2647	52.072	45.917	87.2269	94.6558	78.7218	90.6825	83.8135	87.433	80.0391
25	85.7027	52.1553	45.4607	87.1634	89.2777	72.486	93.1599	83.1068	**91.2196**	78.3667
30	84.2555	52.2714	47.4657	81.3427	88.0537	69.1638	82.7558	76.7973	89.7553	65.5113
35	**90.5197**	51.5954	**48.7118**	76.2751	85.8521	67.5988	84.8251	64.8331	88.1383	46.8531

Supplementary Table S30. Accuracy of the NormOrderedGraphlet-3-4(K) approach when varying the value of K, with respect to **AUROC values** (expressed as percentages), corresponding to the PSN sets of **group 4**. Given a fourth-level PSN data set (within a given column), the AUROC for the "best" K is shown in bold. These results are with respect to the third PSN construction strategy, **(any heavy atom type, 6 Å distance cut-off)**.

K	CATH (group 4 PSN sets)						SCOP (group 4 PSN sets)			
	2.60.40	2.60.120	3.20.20	3.40.50	3.30.390	3.30.420	b.1.1	c.1.8	c.2.1	c.37.1
1	61.9231	80.846	56.3984	76.8403	81.6132	68.7071	92.7306	73.3351	89.0949	77.8485
2	63.8503	79.0992	56.6986	81.0313	82.4887	67.0766	90.2817	74.135	88.3023	80.7745
3	67.2827	77.7912	58.5039	84.3937	86.4468	67.5066	91.8375	74.21	86.8602	85.153
4	68.9244	79.6809	59.2373	87.1569	90.2437	67.3605	91.8503	79.1163	87.3636	87.0635
5	69.502	81.9001	61.6687	**87.262**	96.395	68.4568	92.38	82.195	87.0944	88.9676
6	69.8672	82.4634	62.9092	86.7584	98.1737	72.1418	93.8426	83.6867	87.1866	92.0212
7	70.4001	**83.1234**	63.2443	86.7105	98.5664	73.6554	**93.8988**	84.1994	89.2934	92.5617
8	70.6582	81.997	63.0541	87.0681	98.5218	75.3497	93.4065	85.6497	90.5112	92.8251
9	70.5884	80.53	63.301	86.9024	**98.6032**	76.6545	93.5643	**86.7921**	91.7889	92.8549
10	70.9854	78.6116	63.0485	86.7211	98.3719	79.5705	92.5483	86.3104	92.6652	**93.0744**
15	67.3774	72.5291	64.0915	85.2328	96.838	**81.4138**	84.6318	83.0901	94.2143	92.4696
20	73.4461	70.2905	62.8801	84.2771	93.1287	74.0553	86.0789	83.3695	93.7121	92.1235
25	75.5516	70.7965	61.7688	84.3693	84.7498	67.4313	89.8401	82.5214	94.8091	91.3631
30	75.0603	72.2758	64.5822	77.3308	83.9394	62.3828	73.478	75.6132	**95.2585**	85.5408
35	**84.1389**	71.9648	**66.7003**	72.6947	81.7413	60.6012	77.8861	63.4086	94.8795	76.3867

Supplementary Table S31. Accuracy of the NormOrderedGraphlet-3-4(K) approach when varying the value of K, with respect to **AUPR values** (expressed as percentages), corresponding to the PSN sets of **"equal size", group 1, and group 2**. Given a PSN data set (within a given column), the AUPR for the "best" K is shown in bold. These results are with respect to the fourth PSN construction strategy, (**α-carbon heavy atom type, 7.5 Å distance cut-off**).

K	"Equal size" PSN sets			CATH group 1 and group 2 PSN sets				SCOP group 1 and group 2 PSN sets					
	CATH-95	CATH-99	CATH-251-265	primary	α	β	α/β	primary	α	β	α/β	$\alpha+\beta$	Multi domain
1	88.8431	83.9819	90.8628	60.5659	51.8956	46.7468	**48.3169**	45.5014	25.548	30.1474	27.6516	33.4799	81.0428
2	88.8431	83.9819	90.8628	**60.6806**	51.9012	**46.8516**	48.2409	**45.7172**	25.548	29.6523	26.9975	32.5223	80.9343
3	86.381	**88.2146**	**91.9734**	59.3747	52.9777	43.4511	41.6074	39.0322	31.1765	32.7238	25.3564	34.8323	75.6246
4	84.2478	84.3159	82.7428	57.0439	**53.8082**	42.7943	41.4263	38.0364	**37.7605**	32.2532	32.0617	39.2211	78.1303
5	79.6175	73.6212	62.6654	47.021	52.1721	42.6578	41.3971	33.9409	32.3074	32.6131	35.8785	42.1094	**84.3341**
6	88.9318	77.3292	65.5177	47.4131	51.8073	42.7277	41.4308	33.8321	31.179	32.2488	36.8116	42.2822	83.8473
7	**91.8595**	78.0833	68.7525	47.7573	51.7635	42.6411	41.4459	33.886	29.4273	31.8257	37.6402	**42.5397**	83.0552
8	91.6725	79.1494	67.777	48.1764	51.4675	42.3898	41.5452	34.0373	27.4921	31.3551	38.5109	41.5578	83.0559
9	90.944	78.8992	69.7704	48.4487	51.2689	41.9587	41.8135	34.2945	26.4537	30.4943	39.6777	39.8464	82.8464
10	87.7737	78.5728	72.5591	48.6414	51.0047	41.4769	42.1468	34.6819	25.3205	29.6575	**40.1891**	38.6534	82.3846
15	87.3403	74.313	72.5396	47.8486	50.4027	39.8599	44.7694	36.6845	21.848	27.5757	39.5936	34.4612	80.9101
20	84.5015	61.2599	70.397	45.8211	50.1355	39.095	45.7274	37.8416	19.6916	26.7424	35.4019	29.4524	78.9807
25	75.4033	57.9999	64.9769	42.9801	49.7002	38.4053	41.2381	34.7138	19.7529	24.5951	22.58	26.4226	76.7365
30	68.5486	58.4315	64.7421	40.8205	50.2964	38.9307	37.1878	30.8145	18.6314	29.07	15.2035	25.4298	78.1124
35	77.58	61.7191	67.885	40.3655	50.4257	40.3843	34.3219	29.5807	17.9394	**38.4692**	13.661	26.5499	83.8187

Supplementary Table S32. Accuracy of the NormOrderedGraphlet-3-4(K) approach when varying the value of K, with respect to **AUROC values** (expressed as percentages), corresponding to the PSN sets of **"equal size", group 1, and group 2**. Given a PSN data set (within a given column), the AUROC for the "best" K is shown in bold. These results are with respect to the fourth PSN construction strategy, (**α-carbon heavy atom type, 7.5 Å distance cut-off**).

K	"Equal size" PSN sets			CATH group 1 and group 2 PSN sets				SCOP group 1 and group 2 PSN sets					
	CATH-95	CATH-99	CATH-251-265	primary	α	β	α/β	primary	α	β	α/β	$\alpha+\beta$	Multi domain
1	90.2567	84.921	90.8382	72.3223	53.2511	63.462	64.1874	70.8334	71.6488	72.8474	76.4193	82.6118	74.1232
2	90.2567	84.921	90.8382	**72.4669**	53.2492	**63.7353**	**64.2412**	**71.0422**	71.6488	73.3018	76.1368	82.1871	74.8201
3	87.8314	**89.1521**	**91.8129**	72.1141	**54.5761**	58.9671	59.617	65.3181	74.7569	73.2836	74.6204	82.4707	65.8021
4	85.9533	84.7586	82.6789	71.5377	54.5524	58.0529	59.3533	64.4246	**78.0794**	72.7056	79.041	84.9444	69.3963
5	80.182	74.1683	66.3882	63.1415	52.7972	57.9825	59.2771	60.5741	73.1639	73.2468	81.0659	86.8488	78.6505
6	89.2256	77.5426	68.3932	63.3173	52.5797	58.0038	59.209	60.3691	73.7127	73.0074	81.4648	**87.0866**	78.0709
7	**92.7031**	78.097	69.1729	63.5987	52.4051	57.8828	59.2331	60.3237	72.7913	72.7123	81.9616	87.0253	76.9128
8	92.5768	78.3294	66.4439	63.8186	52.3703	57.5046	59.3461	60.3782	71.4651	72.1416	82.1102	86.7915	76.7777
9	92.0402	78.4694	68.3375	63.858	52.2751	56.7477	59.5146	60.5505	70.7327	71.5235	82.3385	86.2224	76.9629
10	89.1572	77.691	69.8134	63.8394	52.4097	55.994	59.7935	60.8114	70.0342	70.7656	**82.4454**	85.7461	76.7751
15	90.5513	74.0703	74.7981	62.2419	52.4556	53.8546	61.7069	62.4813	68.1002	69.5742	81.5948	81.583	75.7913
20	88.5154	61.7552	70.8716	60.0075	52.1863	53.3326	63.5354	64.0217	67.2697	66.5133	78.8775	78.5447	73.6682
25	77.1886	57.4513	62.0162	58.1845	51.6357	53.0684	61.4442	61.7411	66.4302	63.8628	69.0297	77.5128	69.6175
30	70.9491	59.5486	60.8744	56.4741	52.1547	54.0725	58.0096	57.3902	65.4942	68.1078	61.632	76.3373	71.2653
35	79.5244	60.3999	65.8591	55.7898	52.1073	55.305	54.9449	55.4426	64.8771	**75.7502**	58.5756	76.094	**78.8876**

Supplementary Table S33. Accuracy of the NormOrderedGraphlet-3-4(K) approach when varying the value of K, with respect to **AUPR values** (expressed as percentages), corresponding to the PSN sets of **group 3**. Given a third-level PSN data set (within a given column), the AUPR for the "best" K is shown in bold. These results are with respect to the fourth PSN construction strategy, **(α-carbon heavy atom type, 7.5 Å distance cut-off)**.

K	CATH (group 3 PSN sets)									SCOP (group 3 PSN sets)					
	1.10	1.20	2.30	2.40	2.60	2.160	3.10	3.30	3.40	a.118	b.1	c.1	c.23	c.26	c.55
1	58.1354	39.3314	67.1498	55.422	72.824	**79.0184**	72.2957	43.6019	65.8378	77.839	65.7082	46.2581	73.2493	63.4422	63.262
2	56.2387	39.2412	67.1498	51.9556	73.055	**79.0184**	72.2957	42.6326	65.8378	77.839	65.7082	46.2581	74.4931	63.4422	63.262
3	60.7524	40.3047	69.0648	55.4771	75.3805	77.5306	73.2492	39.5227	66.1794	79.5365	68.7664	45.96	68.1593	**65.6002**	67.3167
4	**69.5787**	**42.7341**	70.8689	55.5767	76.2066	68.0356	**78.3367**	43.6873	68.5006	**84.2318**	69.5806	48.2045	72.1491	65.0757	69.8172
5	60.6952	35.8952	**72.7234**	54.9064	76.4945	73.4	77.0082	43.6895	70.0832	76.887	68.0369	48.0778	79.4193	63.6442	71.9891
6	57.3741	35.1044	71.486	55.6484	76.7197	73.3735	76.6601	44.2515	70.5803	81.0892	67.6773	47.8417	**80.7025**	63.594	73.9224
7	54.8832	34.3091	69.2816	**56.4255**	76.9387	72.0977	75.6384	46.0683	70.9857	82.897	67.4799	47.8269	79.3018	63.2579	73.5932
8	52.2273	33.8036	68.8645	56.1739	77.2385	71.6683	74.7304	**46.148**	72.0293	82.7581	67.6465	47.946	77.8573	63.3885	73.6144
9	49.4512	31.9445	67.3261	54.6589	77.5304	71.125	73.0255	46.0811	72.9776	82.8344	67.5611	47.064	77.3485	63.3211	73.9265
10	45.2042	30.8215	67.4013	52.3085	77.7316	71.7083	72.1019	45.6857	73.6544	83.1045	67.4162	46.6947	76.0877	62.6587	73.8141
15	36.4922	31.0134	59.8178	46.5085	**77.8497**	70.6491	68.5533	39.8523	77.6632	80.3234	67.5138	45.298	70.256	62.6678	**77.2722**
20	33.6279	29.9751	57.6753	48.7534	74.9074	68.5509	59.7453	35.8337	**77.8226**	60.2597	67.294	43.6858	71.4202	64.8257	71.6166
25	33.1401	29.6154	55.0862	43.9259	72.5768	69.6571	57.6179	31.9714	69.4368	54.7952	66.35	46.8379	68.6183	64.176	68.1967
30	33.0309	28.2701	47.6857	45.6634	73.5493	69.418	57.6615	28.2461	61.8741	54.3218	68.5835	55.1746	65.7308	63.7937	61.3078
35	32.0668	28.3149	43.1478	39.8804	77.8126	60.6809	58.1238	29.1973	56.4644	51.133	**72.1915**	**56.5878**	55.9888	57.0831	58.0056

Supplementary Table S34. Accuracy of the NormOrderedGraphlet-3-4(K) approach when varying the value of K, with respect to **AUROC values** (expressed as percentages), corresponding to the PSN sets of **group 3**. Given a third-level PSN data set (within a given column), the AUROC for the "best" K is shown in bold. These results are with respect to the fourth PSN construction strategy, **(α-carbon heavy atom type, 7.5 Å distance cut-off)**.

K	CATH (group 3 PSN sets)									SCOP (group 3 PSN sets)					
	1.10	1.20	2.30	2.40	2.60	2.160	3.10	3.30	3.40	a.118	b.1	c.1	c.23	c.26	c.55
1	87.6375	71.1662	82.9643	81.9219	69.6707	**77.0557**	92.033	76.8625	65.664	77.5674	56.8016	57.6255	82.1679	55.7089	61.9367
2	87.0885	71.0421	82.9643	80.5781	69.9049	**77.0557**	92.033	76.2905	65.664	77.5674	56.8016	57.6255	83.1213	55.7089	61.9367
3	87.7353	71.511	82.5421	**82.2409**	72.7248	76.6211	90.9587	75.2006	64.7439	78.9976	60.2077	57.9818	78.5257	**58.4678**	64.9943
4	**90.0882**	**72.652**	83.0183	82.1303	73.7774	65.0399	92.5605	77.5103	65.62	83.8522	60.5179	59.8306	80.3366	57.5098	68.3321
5	86.0937	68.0106	**83.917**	81.8253	74.1819	71.4721	92.6245	78.2413	66.6256	76.3553	58.5178	59.485	85.5604	56.0784	71.2737
6	86.19	68.5764	83.1906	81.2978	74.4199	71.3711	92.6522	78.2629	67.0518	80.7421	57.9859	59.5557	**86.614**	55.5187	73.4135
7	85.6461	67.8442	82.0842	81.3976	74.7086	69.4766	**92.7969**	78.6377	67.634	83.4349	57.3657	59.4786	85.6392	55.2786	73.2542
8	85.2324	67.3279	82.0387	80.6917	75.1553	68.7024	92.6449	**78.6625**	69.106	83.8675	57.5425	59.6238	84.7139	55.5397	73.5871
9	84.2239	65.7371	81.4923	79.1972	75.5296	67.8896	92.7051	78.6568	70.4356	84.2055	57.541	58.8698	84.6407	55.5582	74.3091
10	82.4121	65.7452	81.66	77.2219	75.8189	68.018	92.4845	78.5185	70.992	**84.3111**	57.3684	58.4974	83.9965	54.9725	74.5965
15	77.4718	66.7315	78.6178	75.4216	**75.8511**	67.8143	87.4993	75.3538	75.9702	80.6043	57.7412	57.0519	79.0796	55.185	**77.8438**
20	75.3094	66.0918	74.2646	75.0568	70.4325	66.204	80.4314	73.6894	**76.8839**	59.6677	57.7063	55.0779	79.6403	57.0979	71.9201
25	75.2843	65.4233	71.6921	70.0207	65.8945	71.3203	80.6005	70.2963	70.3734	55.5534	55.6421	59.2079	76.9718	57.6381	66.5605
30	74.4962	64.9241	66.933	68.83	66.3088	72.7648	80.2978	70.0081	62.342	53.7483	59.7428	69.3336	78.2393	56.0599	59.1726
35	75.0192	65.1138	66.2893	65.4057	70.489	62.0254	82.9052	69.2266	56.6622	51.1041	**67.979**	**72.079**	67.1492	51.9152	56.2232

Supplementary Table S35. Accuracy of the NormOrderedGraphlet-3-4(K) approach when varying the value of K, with respect to **AUPR values** (expressed as percentages), corresponding to the PSN sets of **group 4**. Given a fourth-level PSN data set (within a given column), the AUPR for the "best" K is shown in bold. These results are with respect to the fourth PSN construction strategy, **(α-carbon heavy atom type, 7.5 Å distance cut-off)**.

K	CATH (group 4 PSN sets)						SCOP (group 4 PSN sets)			
	2.60.40	2.60.120	3.20.20	3.40.50	3.30.390	3.30.420	b.1.1	c.1.8	c.2.1	c.37.1
1	75.9748	**60.281**	40.3468	76.7057	87.6089	65.4781	**96.158**	81.0388	71.8395	48.3007
2	75.9748	59.6238	39.9837	76.7057	89.3839	65.4781	**96.158**	81.0388	69.6346	48.3007
3	78.0723	55.5108	38.9854	76.8475	88.8841	68.1251	94.5529	79.3179	66.9285	53.5829
4	78.9764	53.3714	38.9187	**84.7305**	90.5671	71.0885	94.9053	80.784	65.4408	63.1727
5	78.6744	54.2546	38.9512	83.504	94.0713	71.73	95.1607	82.2099	61.3826	67.4267
6	77.9063	55.3686	39.0337	84.3483	95.1756	72.7377	95.0044	82.7554	63.9909	69.5374
7	77.0711	56.5628	38.7899	83.9928	**95.8933**	73.6223	94.9092	82.8515	65.5323	70.8651
8	76.7891	54.7631	38.9813	82.5367	95.279	75.0661	94.5455	**83.37**	67.7004	70.954
9	76.5725	51.9151	38.5553	83.1121	95.1389	75.56	94.2697	83.2591	68.5594	69.8676
10	76.4074	49.9229	38.41	83.7956	94.3019	76.2949	93.8737	82.6478	68.6326	71.0514
15	77.3597	43.5707	38.9282	83.6732	91.4096	**82.8086**	92.743	76.0964	70.9192	72.2997
20	78.9179	41.995	37.8712	80.8182	89.0286	80.0477	91.1395	73.1607	68.3985	**74.0515**
25	78.1325	42.3784	41.4048	80.7814	85.2432	72.7014	91.3212	72.6468	74.3658	70.0479
30	78.8802	44.1019	44.1644	74.79	83.9161	66.9427	76.1695	64.4536	73.1715	54.547
35	**85.1779**	45.3235	**47.0565**	70.647	72.9256	62.7003	75.366	58.2631	**74.8209**	38.3095

Supplementary Table S36. Accuracy of the NormOrderedGraphlet-3-4(K) approach when varying the value of K, with respect to **AUROC values** (expressed as percentages), corresponding to the PSN sets of **group 4**. Given a fourth-level PSN data set (within a given column), the AUROC for the "best" K is shown in bold. These results are with respect to the fourth PSN construction strategy, **(α-carbon heavy atom type, 7.5 Å distance cut-off)**.

K	CATH (group 4 PSN sets)						SCOP (group 4 PSN sets)			
	2.60.40	2.60.120	3.20.20	3.40.50	3.30.390	3.30.420	b.1.1	c.1.8	c.2.1	c.37.1
1	59.8358	**77.7223**	58.5183	73.7225	85.2117	58.6433	**94.7276**	81.1325	81.406	74.181
2	59.8358	77.5636	58.4297	73.7225	86.6791	58.6433	**94.7276**	81.1325	79.7128	74.181
3	62.7657	73.8902	56.691	73.8067	87.5161	60.4669	92.1326	79.1385	77.7052	78.1819
4	63.8209	73.4341	56.4112	**82.5153**	88.7374	64.216	92.6955	80.6655	76.5516	84.5689
5	62.9196	74.4412	56.4837	81.0979	92.359	65.9266	92.9437	82.2518	72.0792	86.8237
6	61.3439	76.2139	56.6554	82.1668	93.8205	67.7378	92.8057	82.7001	73.9608	87.7055
7	59.8937	76.8999	56.538	81.6057	**94.7698**	68.6206	92.5581	82.5395	75.6909	88.2454
8	59.1361	74.7375	56.9021	79.9357	93.9884	70.4182	91.8584	**82.9576**	77.2546	88.7652
9	58.7285	71.9884	56.3349	80.3321	93.7606	70.9957	91.3487	82.7408	78.1883	88.8296
10	58.3989	70.3568	56.032	81.082	92.5692	72.0937	90.7072	81.7822	79.36	89.2673
15	60.4502	65.4611	55.264	80.0487	89.3785	**80.5082**	88.7669	74.0557	81.5591	89.823
20	63.4902	61.8238	53.8288	75.5086	87.3536	78.0603	85.0819	72.0505	79.2255	**90.2375**
25	63.694	61.533	58.395	77.2325	81.5426	67.4442	86.2435	71.0509	85.082	89.0826
30	66.3266	64.3655	62.7956	69.877	81.6746	58.8211	63.2119	60.8881	87.3229	80.6136
35	**78.3525**	65.7359	**65.0625**	65.0186	66.7942	55.7494	68.1158	54.5799	**87.7236**	66.3309

References

1. Berman, H. M. *et al.* The Protein Data Bank. *Nucleic Acids Research* **28**, 235–242 (2000).
2. Sillitoe, I. *et al.* CATH: comprehensive structural and functional annotations for genome sequences. *Nucleic Acids Research* **43**, D376–D381 (2015).
3. Orengo, C. A. *et al.* The CATH database provides insights into protein structure/function relationships. *Nucleic Acids Research* **27**, 275–279 (1999).
4. Murzin, A. G., Brenner, S. E., Hubbard, T. & Chothia, C. SCOP: a structural classification of proteins database for the investigation of sequences and structures. *Journal of Molecular Biology* **247**, 536–540 (1995).
5. Milenković, T., Lai, J. & Pržulj, N. GraphCrunch: a tool for large network analyses. *BMC Bioinformatics* **9** (2008).
6. Kuchaiev, O., Stevanović, A., Hayes, W. & Pržulj, N. GraphCrunch 2: Software tool for network modeling, alignment and clustering. *BMC Bioinformatics* **12** (2011).
7. Malod-Dognin, N. & Pržulj, N. GR-Align: fast and flexible alignment of protein 3D structures using graphlet degree similarity. *Bioinformatics* **30**, 1259–65 (2014).
8. Pržulj, N. Biological network comparison using graphlet degree distribution. *Bioinformatics* **23**, e177–e183 (2007).
9. Pržulj, N., Corneil, D. G. & Jurisica, I. Modeling interactome: Scale-free or geometric? *Bioinformatics* **20**, 3508–3515 (2004).
10. Yaveroglu, O. N. *et al.* Revealing the Hidden Language of Complex Networks. *Scientific Reports* **4**, 4547 (2014).
11. Vacic, V., Iakoucheva, L. M., Lonardi, S. & Radivojac, P. Graphlet Kernels for Prediction of Functional Residues in Protein Structures. *Journal of Computational Biology* **17**, 55–72 (2010).
12. Lugo-Martinez, J. & Radivojac, P. Generalized graphlet kernels for probabilistic inference in sparse graphs. *Network Science* **2**, 254–276 (2014).
13. Pabuwal, V. & Li, Z. Network pattern of residue packing in helical membrane proteins and its application in membrane protein structure prediction. *Protein Engineering, Design and Selection* **21**, 55–64 (2008).
14. Pabuwal, V. & Li, Z. Comparative analysis of the packing topology of structurally important residues in helical membrane and soluble proteins. *Protein Engineering, Design and Selection* **22**, 67–73 (2009).
15. Gao, J. & Li, Z. Conserved network properties of helical membrane protein structures and its implication for improving membrane protein homology modeling at the twilight zone. *Journal of Computer-Aided Molecular Design* **23**, 755–763 (2009).
16. Emerson, I. A. & Gothandam, K. M. Network analysis of transmembrane protein structures. *Physica A* **391**, 905–916 (2012).
17. Emerson, I. A. & Gothandam, K. M. Residue centrality in alpha helical polytopic transmembrane protein structures. *Journal of Theoretical Biology* **309**, 78–87 (2013).
18. Newman, M. E. J. Assortative mixing in networks. *Physical Review Letters* **89**, 208701 (2002).
19. Holm, L. & Rosenström, P. Dali server: conservation mapping in 3D. *Nucleic Acids Research* **38**, W545–W549 (2010).
20. Zhang, Y. & Skolnick, J. TM-align: a protein structure alignment algorithm based on the TM-score. *Nucleic Acids Research* **33**, 2302–09 (2005).
