# Supplementary material for: Author Correction: GRAFENE: Graphlet-based alignment-free network approach integrates 3D structural and sequence (residue order) data to improve protein structural comparison
Source: Sci Rep. 2020 Aug 10;10:13455. doi: 10.1038/s41598-020-69025-8 (PMC7417526; doi:10.1038/s41598-020-69025-8)
Supplement: Supplementary file 1 — Original Supplementary Information [file 41598_2020_69025_MOESM1_ESM.pdf]

Supplementary Information for: GRAFENE: Graphlet-based alignment-free network approach integrates 3D structural and sequence (residue order) data to improve protein structural comparison

Fazle E. Faisal[1,5,6,†], Khalique Newaz[1,5,6,†], Julie L. Chaney[2], Jun Li[3], Scott J. Emrich[1], Patricia L. Clark[2,4,6], and Tijana Milenković[1,5,6,*]

[1]Department of Computer Science and Engineering, University of Notre Dame, Notre Dame, IN 46556, USA
[2]Department of Chemistry and Biochemistry, University of Notre Dame, Notre Dame, IN 46556, USA
[3]Department of Applied and Computational Mathematics and Statistics, University of Notre Dame, Notre Dame, IN 46556, USA
[4]Department of Chemical and Biomolecular Engineering, University of Notre Dame, Notre Dame, IN 46556, USA
[5]Interdisciplinary Center for Network Science and Applications, University of Notre Dame, Notre Dame, IN 46556, USA
[6]Eck Institute for Global Health, University of Notre Dame, Notre Dame, IN 46556, USA
[†]These authors equally contributed to this work
[*]To whom correspondence should be addressed

I Supplementary Sections

S1 Data

We collect 3D atomic structures of proteins from the Protein Data Bank (PDB)[1]. Since PDB contains multiple copies of the same or nearly identical proteins, we aim to reduce the redundancy by selecting a set of proteins from PDB such that each protein in the set is not more than 90% sequence identical to any other protein in the set. If a protein is not more than 90% sequence identical to any other protein from PDB, we immediately select the protein. If a protein is more than 90% sequence identical to one or more proteins from PDB, we select a "representative" protein from such a protein group so that the representative protein is of the highest quality (in terms of resolution) among all proteins in the group. This strategy results in the selection of 17,036 proteins. We denote this data set as *ProteinPDB*. Each protein in the data is comprised of the X, Y, and Z orthogonal Angstrom (Å) coordinates of heavy atoms (i.e., *carbon*, *nitrogen*, *oxygen*, and *sulfur*) of each amino acid within the protein. The data is available at http://www.rcsb.org/pdb/home/home.do for free download.

Both Class, Architecture, Topology, Homology (CATH) and Structural Classification of Proteins (SCOP) are protein domain categorization databases[2–4]. A protein is typically composed of one or more domains (a domain refers to a part of a protein structure that can fold and often function independently). The purpose of CATH and SCOP is to annotate these domains. We use the protein domain categorization schemes of CATH and SCOP to assign labels to the protein domains from ProteinPDB.

S2 Synthetic networks

We generate synthetic networks by using different network models. A good approach should identify networks from the same network model (i.e., with the same label) as similar, and it should identify networks from different models (i.e., with different labels) as dissimilar. Specifically, we use three well-established network models: *Erdős-Rényi random graphs (ER)*, *geometric random graphs (GEO)*, and *scale-free random graphs (SF)*[5,6]. We note that these models are not necessarily representative of PSNs. Instead, they are general-purpose models. This is intentional, because the models that we use are intended to illustrate wide applicability of our GRAFENE approach to any domain where data can be modeled as networks. It is our analyses of real-world PSNs that focus specifically on the task of PC.

First, we evaluate the considered approaches on synthetic networks of the same size but of different labels (originating from the three network models). To evaluate the robustness of GRAFENE to the choice of network size, we repeat this analysis three times, by increasing the size of the considered networks. That is, we perform three separate analyses of three different network data sets, where in a given data set, all networks are of the same size, and one third of the networks in the set comes from each of the three network models. We denote these network sets as *Synthetic-100*, *Synthetic-500*, and *Synthetic-1000* (Supplementary Table S1), where each set consists of 50 networks per model (totaling to $50 \times 3 = 150$ networks). The numbers of nodes and edges in these networks are set to mimic sizes of real-world PSNs.

Second, we evaluate the considered approaches on networks of different sizes as well as different labels, to check whether the approaches can correctly identify as similar networks from the same model despite the networks being of different sizes, as well as that they can correctly identify as dissimilar networks from different models despite the networks being of the same size. To generate a synthetic network set of different sizes, we combine networks from Synthetic-100, Synthetic-500, and Synthetic-1000 together. We denote the combined network set as *Synthetic-all* (Supplementary Table S1).

S3 Forming real-world PSNs

Here, we continue our discussion regarding the fourth PSN construction strategy that uses the α-carbon atom type and the 7.5 Å distance cut-off. Note that the original GR-Align study used a distance cut-off of 12 Å because this study argued that when considering the α-carbon atom type, this cut-off showed better performance compared to all other tested cut-offs (in the 5 Å-20 Å range)[7]. However, we use the 7.5 Å cut-off for the following reasons. First, even at this cut-off, GR-Align is already much slower than our proposed GRAFENE approach (as we show in our evaluation), and increasing the distance cut-off would only result in more edges and thus further slow down GR-Align. And it was the original GR-Align study that recommended using the 7.5 Å cut-off when aiming to achieve speed-up (as reflected by linear time complexity at this cut-off). Second, as demonstrated in the GR-Align study, for two out of three evaluated performance measures, the improvement when using the 12 Å cut-off compared to when using the 7.5 Å cut-off is negligible and thus not worth the extra increase in computational time that would result from using the 12 Å cut-off compared to using the 7.5 Å cut-off.

S4 Real-world PSNs with CATH categorization

ProteinPDB contains 17,884 protein domains that have CATH categorization, which for a given PSN construction strategy results in 17,884 PSNs. Of these PSNs, to ensure that PSNs are of reasonable "confidence", we focus for further analyses on those PSNs that meet all of the following criteria: 1) the given network has more than 100 nodes, 2) the maximum diameter of the network is more than five, and 3) the network is composed of a single connected component. For different PSN construction strategies, the above criteria can result in different numbers of PSNs. For the first PSN construction strategy (any heavy atom type, 4 Å distance cut-off), this results in 9,509 such PSNs. In the main paper (also, see Supplementary Table S2), we report the number of PSNs with respect to this PSN construction strategy. The number of PSNs resulting from using one of the other three PSN construction strategies is of the similar order.

First, we test how well the considered PC approaches can compare PSNs between the top hierarchical categories (i.e., labels) of CATH: *alpha* (α), *beta* (β), *alpha/beta* (α/β), and *few secondary structures*. Only for few secondary structures, none of the domains in ProteinPDB belong to this category, and so we remove the few secondary structures category from further consideration. Of the 9,509 PSNs, 2,628, 3,085, and 3,796 PSNs belong to (i.e., are labeled with) α, β, and α/β categories, respectively. We denote this PSN set as *CATH-primary* (Fig. 2 in the main paper). The set contains a large enough number of PSNs in each category, which ensures enough statistical power for further analyses.

Second, we test how well the PC approaches can compare PSNs between the second-level hierarchical categories of CATH. That is, within each of the top-level categories of CATH, we compare PSNs belonging to their sub-categories, i.e., second-level categories of CATH. To ensure enough statistical power for further analyses, we focus only on those top-level categories that have at least two sub-categories with at least 30 PSNs each. Each of the three top-level CATH categories satisfies this, and hence, for each of them, we analyze all of their sub-categories that each contain at least 30 PSNs. This results in three PSN sets, denoted as α, β, and α/β (Fig. 2 in the main paper).

Third, we test how well the PC approaches can compare PSNs between the third-level hierarchical categories of CATH. That is, within each of the second-level categories of CATH, we compare the PSNs belonging to their sub-categories, i.e., third-level categories of CATH. To ensure enough statistical power for further analyses, we focus only on those second-level categories that have at least two sub-categories with at least 30 PSNs each. This results in nine PSN sets, denoted as 1.10, 1.20, 2.30, 2.40, 2.60, 2.160, 3.10, 3.30, and 3.40 (Fig. 2 in the main paper).

Fourth, we test how well the PC approaches can compare PSNs between the fourth-level hierarchical categories of CATH. That is, within each of the third-level categories of CATH, we compare PSNs belonging to their sub-categories, i.e., fourth-level categories of CATH. To ensure enough statistical power for further analyses, we focus only on those third-level categories that

have at least two sub-categories with at least 30 PSNs each. This results in six PSN sets, denoted as 2.60.40, 2.60.120, 3.20.20, 3.30.390, 3.30.420, and 3.40.50 (Fig. 2 in the main paper).

Thus, in total, we analyze $1+3+9+6=19$ CATH PSN sets (Fig. 2 in the main paper and Supplementary Tables S3-S5).

S5 Real-world PSNs with SCOP categorization

ProteinPDB has 15,762 protein domains with SCOP categorization, which results in 15,762 PSNs. Of these PSNs, to ensure that PSNs are of reasonable "confidence", we focus on those PSNs that meet the same three criteria that PSNs with CATH categorization are also required to meet, resulting in 11,451 PSNs with SCOP categorization (again, for the first of the four PSN construction strategies). For details, see Supplementary Table S2.

Again, first, we evaluate how well the considered PC approaches can compare PSNs between the top hierarchical categories of SCOP: α, β, α/β, *alpha plus beta* (α+β), *coiled coil*, *membrane*, *multi-domain*, *small*, *low resolution*, *peptide*, and *designed*. For small, low resolution, peptide, or designed, none of the domains in ProteinPDB belong to these categories, and so we remove these four categories from further consideration. Of the 11,451 PSNs, 1,678, 2,541, 3,835, 2,879, 44, 156, and 318 PSNs belong to α, β, α/β, α+β, coiled coil, membrane, and multi-domain categories, respectively. This PSN set, denoted as *SCOP-primary* (Fig. 2 in the main paper), contains enough PSNs in each category to ensure enough statistical power for further analyses. Second, we test how well the PC approaches can compare PSNs between the second-level hierarchical categories of SCOP. This results in five PSN sets, denoted as α, β, α/β, $\alpha+\beta$, and multi-domain (Fig. 2 in the main paper). Third, we test how well the PC approaches can compare PSNs between the third-level hierarchical categories of SCOP. This results in six PSN sets, denoted as *a*.118, *b*.1, *c*.1, *c*.23, *c*.26, and *c*.55 (Fig. 2 in the main paper). Fourth, we test how well the PC approaches can compare PSNs between the fourth-level hierarchical categories of SCOP. This results in four PSN sets, denoted as *b*.1.1, *c*.1.8, *c*.2.1, and *c*.37.1 (Fig. 2 in the main paper).

Thus, in total, we analyze $1+5+6+4=16$ SCOP PSN sets (Fig. 2 in the main paper and Supplementary Tables S3-S5).

S6 Real-world PSNs of the same size

To benchmark PSN-based approaches for protein comparison in a way that the comparison cannot be biased by PSN size, we need PSN data of the same (or at least similar) network size (analogous to the synthetic network data sets). For this analysis, we focus on PSNs of α and β labels from the CATH-primary data set. First, within this data set, we aim to identify PSNs that are of reasonable size, i.e., that have $\sim$100 nodes. We further filter the resulting PSN set according to the following rules: 1) the number of nodes in all α and β PSNs is the same, 2) the number of edges in all α and β PSNs is statistically significantly similar (Mann-Whitney U test; p-value < 0.05), and 3) there are at least six PSNs in each of the two label categories. We end up with two such PSN sets. The first set is comprised of 24 PSNs having 95 nodes and 343-362 edges, where 12 PSNs are from α and 12 PSNs are from β. We denote this PSN set as *CATH-95*. The second set is comprised of 28 PSNs having 99 nodes and 347-374 edges, where 12 PSNs are from α and 16 PSNs are from β. We denote this PSN set as *CATH-99*. Second, within the CATH-primary data set, we aim to identify even larger PSNs, i.e., PSNs that have $\sim$250 nodes. We again further filter the resulting PSN set according to the same three rules as above, except that in rule 1, we do not force the number of nodes of all PSNs to match (as we could not identify multiple PSNs that satisfy this constraint) but instead it is sufficient that the PSNs are of statistically significantly similar size in terms of the number of nodes (Mann-Whitney U test; p-value < 0.05). This results in another PSN set, which is comprised of 16 PSNs having 251-265 nodes and 1,003-1,076 edges, where nine PSNs are from α and seven PSNs are from β. We denote this PSN set as *CATH-251-265*. Note that the reported numbers of PSNs in these three "equal size" PSN sets are with respect to the first PSN construction strategy (any heavy atom type, 4 Å distance cut-off). Yet, the numbers remain the same for the other three PSN construction strategies.

S7 Existing approaches

S7.1 Existing network approaches

Existing approaches of this type that we use for PC (not all of which were proposed for PC but can be adapted to it) can be categorized into graphlet and non-graphlet approaches. None of them use PCA as we do.

Existing graphlet approaches. These include graphlet degree distribution agreement (GDDA)[8], relative graphlet frequency distance (RGFD)[9], graphlet correlation distance (GCD)[10], and GR-Align[7]. Among them, GDDA, RGFD, and GCD can compare any type of networks, while GR-Align has been specifically designed to compare PSNs. GDDA, RGFD, and GCD are alignment-free, while GR-Align is alignment-based. For each network pair, each of the four existing graphlet-based network approaches outputs a similarity (or equivalently, a distance) score. Then, for each approach, we sort all network pairs in terms of their increasing distance and evaluate the given approach the given approach as discussed in Section "Evaluation of PC accuracy" of the main manuscript.

Two alternative graphlet approaches were used in the context of PSNs[11,12], but they were used to predict (classify in a supervised manner) functional residues in PSNs (where residues are nodes in PSNs) and not for PSN comparison. Since these approaches compare nodes rather than networks, and since they are supervised (while our study is unsupervised, per our discussion in Section "Evaluation of PC accuracy" of the main manuscript), the approaches do not fit the context of our study. As such, we do not consider them further.

Existing non-graphlet approaches. Several PSN measures have already been used for PC: *average degree*, *average distance*, *maximum distance*, *average closeness centrality*, *average clustering coefficient*, *intra-hub connectivity*, and *assortativity*[13–17]. For each measure, for each pair of networks, we compute Euclidean distance between the networks' vectors (because all vectors are 1-dimensional, here we cannot use cosine similarity as for our GRAFENE approach). We describe these measures below.
Average degree. The average degree of a network can be interpreted as a measure of the overall connectivity of the network. The degree of a node is the number of its network neighbors. The average degree of a network is the average of degrees of all nodes in the network. This measure has been used for analyzing protein structures by[13–17].
Average distance. The distance between two nodes in a network is the length of the shortest path between the nodes. The average distance of a network is the average of distances over all pairs of nodes in the network. This measure has been used for analyzing protein structures by[16,17].
Maximum distance. The maximum distance of a network is the largest of all distances in the network. This measure has been used for analyzing protein structures by[16].
Average closeness centrality. The *closeness centrality* of a node in a network can be interpreted to be the *nearness* of the node to all other nodes in the network. The closeness centrality $cl(v)$ of a node $v \in V$ is computed as $cl(v) = \frac{1}{\sum_{u \in V} d(u,v)}$, where $d(v,u)$ is the distance between nodes v and u. The average closeness centrality of a network is the average of the closeness centrality values of all nodes in the network. This measure has been used for analyzing protein structures by[16,17].
Average clustering coefficient. The *clustering coefficient* of a node in a network can be interpreted as a measure of the connectivity between the neighbors of the node. Given a node v with m neighbors, the clustering coefficient $cc(v)$ of the node v is computed as $cc(v) = \frac{b}{\frac{m(m-1)}{2}}$, where b is the number of edges in the network connecting the m neighbors of v. The average clustering coefficient of a network is the average of clustering coefficient values of all nodes in the network. This measure has been used for analyzing protein structures by[16,17].
Intra-hub connectivity. The intra-hub connectivity of a network can be interpreted as the overall connectivity of the hub nodes within the network.[14] defined a node to be a hub in a PSN if the degree of the node is at least three. We adopt the same strategy to define a hub node in this study. Given k such hub nodes in a network, the intra-hub connectivity of the network is computed as $\frac{m}{\frac{k(k-1)}{2}}$, where m is the number of connections between the hub nodes and $\frac{k(k-1)}{2}$ is the maximum possible number of connections between the hub nodes. This measure has been used for analyzing protein structures by[14].
Assortativity. The assortativity of a network can be interpreted as the tendency of the high degree nodes to be connected with other high degree nodes (see[18] for details). This measure has been used for analyzing protein structures by[16].

We combine the seven measures into an eighth measure, *Existing-all*, to investigate whether the integration of different and complementary topological measures helps PC. We use Existing-all within our PCA framework. This way, we can fairly compare our graphlet measures (i.e., different versions of our GRAFENE approach) and the existing non-graphlet measures within the same framework.

S7.2 Existing 3D contact approaches

These include DaliLite[19] and TM-align[20]. Given two proteins (i.e., 3D co-ordinates of their residues), each of DaliLite and TM-align outputs the proteins' structural similarity score: z-score in the case of DaliLite and TM-score in the case of TM-align. In our evaluation framework, we sort all protein pairs in terms of their increasing distance, i.e., decreasing z-scores for DaliLite and decreasing TM-scores for TM-Align, and then we evaluate DaliLite and TM-Align as discussed in Section "Evaluation of PC accuracy" of the main manuscript.

S7.3 Existing sequence approach

The sequence-based approach that we use, which we call AAComposition, works as follows. For a given protein, for each amino acid type i (out of 20 possible types), we divide the number of amino acids of type i by the total number of amino acids in the protein sequence. We use the resulting 20 values, along with the length of the protein sequence, as the protein's sequence-based measure (i.e., feature vector). Then, we use this measure within our PCA framework. This way, we can fairly compare network- and sequence-based measures within the same framework.

S8 Performance trends of different PC approaches on same PSN sets and of same PC approaches on different PSN sets

Performance trends of different PC approaches on same PSN sets. We sometimes observe a difference in trends between different PC approaches for same PSN sets. Specifically, in the case of the CATH database, all approaches result in a consistent trend that their accuracy for CATH-α is higher than their accuracy for CATH-β. Similarly, in the case of the SCOP database, the majority of the approaches show a consistent trend that their accuracy for SCOP-β is higher than their accuracy for SCOP-α, except the GDDA, GCD, and AAComposition PC approaches, whose accuracy for SCOP-α is higher than their accuracy for SCOP-β. This difference in the trends between the different approaches (GDDA, GCD, and AAComposition versus all others) for SCOP is an approach-specific issue, meaning that some approaches might simply work better for (i.e., better capture patterns in) data of type 1 (e.g., α) than for data of type 2 (e.g., β), while other approaches might show the opposite trend (i.e., work better for data of type 2 than for data of type 1). It is hard to explain why this is, especially for the network-based approaches, because these approaches are heuristics (due to the computational intractability, i.e., NP-hardness, of the network comparison problem) without a theoretic guarantee on their accuracy (and especially on their accuracy on certain data types as opposed to other data types).

Performance trends of same PC approaches on different PSN sets. Additionally, we observe a difference in the performance of same PC approaches on different PSN sets. Specifically, a given approach might have higher accuracy for CATH-α than for CATH-β, but the same approach might have lower accuracy for SCOP-α than for SCOP-β. This trend inconsistency holds for all considered PC approaches except GDDA, GCD, and AAComposition; for both CATH and SCOP, the accuracy of these three approaches is higher for α than for β. This trend inconsistency is likely a data-specific issue: 1) CATH and SCOP do not necessarily contain the exact same PSNs (meaning that some PSNs that are in CATH might be missing from SCOP, and vice versa), and 2) for those PSNs that are in both CATH and SCOP, the PSNs might be categorized into some protein domain group (e.g., α) in CATH but to a different protein domain group (e.g., α/β) in SCOP, because the methodologies that CATH and SCOP use to categorize proteins into domain groups are not identical. If any of these two conditions is met, this could explain the observed trend inconsistency. Indeed, we find that:

1. Of all (α, β, or α/β) PSNs that are in CATH, only 27% are in SCOP. Similarly, of all (α, β, α/β, or α+β) PSNs that are in SCOP, only 24% are in CATH. That is, most of the PSNs are unique to CATH and SCOP.
2. For all PSNs that are present in both CATH and SCOP:
    - 8% of the PSNs that are labeled as α in CATH are labeled as β, α/β, or α+β in SCOP.
    - 0.3% of the PSNs that are labeled as α in SCOP are labeled as β or α/β in CATH.
    - 37% of the PSNs that are labeled as β in CATH are labeled as α, α/β, or α+β in SCOP.
    - 38% of the PSNs that are labeled as β in SCOP are labeled as α or α/β in CATH.
    - 40% of the PSNs that are labeled as α/β in CATH are labeled as α or β in SCOP.
    - 43% of the PSNs that are labeled as α/β or α+β in SCOP are labeled as α or β in CATH.

Clearly, both of the above conditions are met, and hence, the observed trend inconsistency is not surprising.

Note that the above results are with respect to the first PSN construction strategy (any heavy atom, 4 Å) and the performance evaluation using AUPR.

II Supplementary Figures

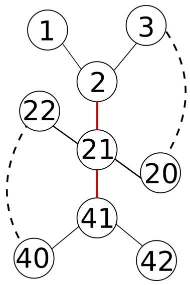

Supplementary Figure S1. Illustration of the importance of "long-range(K)" ordered graphlets. A PSN is shown for a toy protein that consists of 42 amino acids in the sequence, i.e., nodes in the PSN (amino acids 4-19 and 23-39 are not shown for simplicity, as indicated by dashed lines). The nodes are denoted by their amino acid positions (i.e., residue order) in the sequence. Black solid lines are network edges that indicate sequence closeness of the corresponding amino acids (meaning that the amino acids are adjacent in the sequence), which in turn yields sufficient 3D spatial proximity of the amino acids. On the other hand, red solid lines are network edges that indicate only spatial proximity, without sequence adjacentness. On the one hand, both the three-node path 1–2–3 as well as the three-node path 2–21–41 correspond to the same ordered graphlet, namely O_1 from Fig. 3 in the main manuscript, under the traditional ordered graphlet approach. However, we argue that the latter is more interesting than the former, as the former is O_1 simply because of sequence adjacentness of amino acids 1 and 2 as well as 2 and 3, while the latter is O_1 because of spatial proximity of amino acids 2 and 21 as well as 21 and 41. On the other hand, even for K value as low as two, the path 1–2–3 will not be detected as O_1 under the "long-range(K)" ordered graphlet approach, while the path 2–21–41 will, because all of its linked node pairs are at least two amino acids apart in the sequence. Note that the path 2–21–41 will be identified as O_1 up to K value of $min(21-2, 41-21) = 19$).

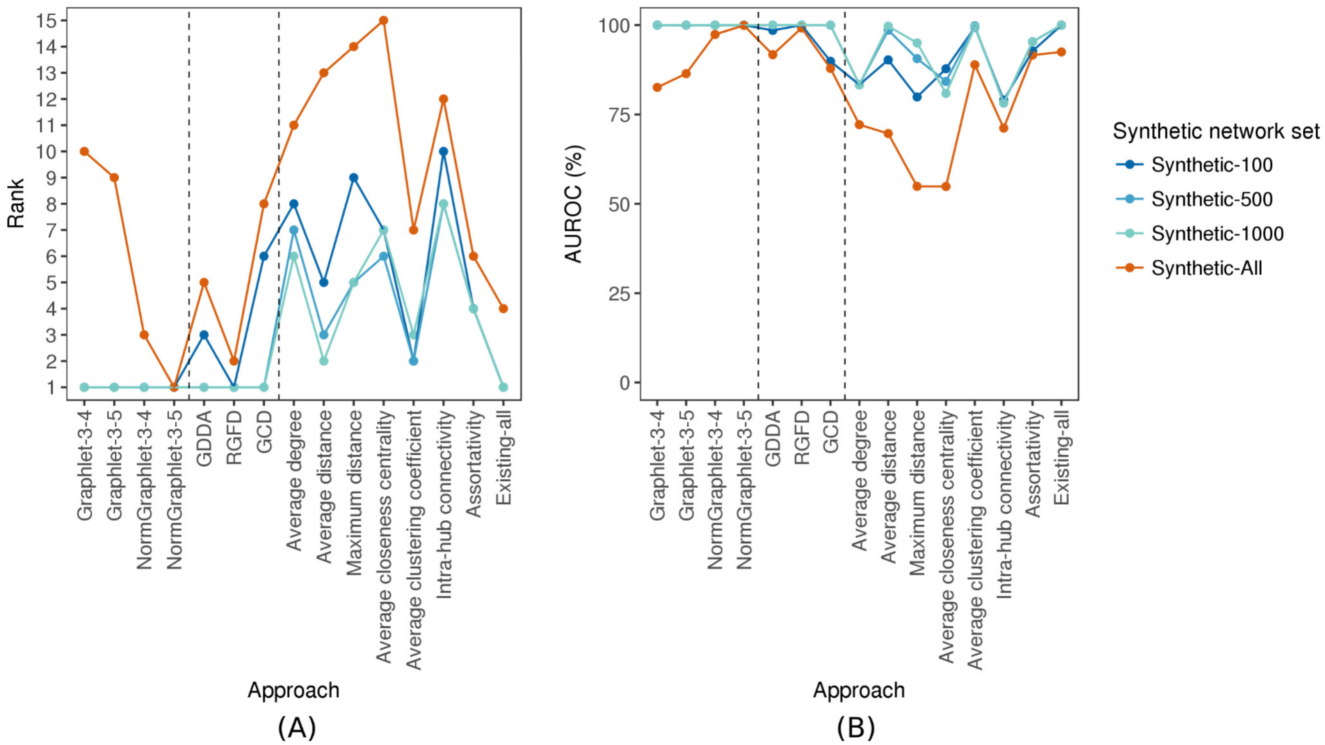

Supplementary Figure S2. The performance comparison of the 15 considered approaches on each of the four considered synthetic network sets, with respect to AUROC, in terms of: (A) the approaches' ranks compared to one another, and (B) the approaches' raw AUROC values. In panel (A), for a given synthetic network set, the 15 approaches are ranked from the best (rank 1) to the worst (rank 15). So, the lower the rank, the better the approach. In panel (B), for each approach, its raw AUPR value is shown for each of the four synthetic network sets. So, the higher the AUROC value, the better the approach. For equivalent results with respect to AUPR values, see Fig. 4 in the main manuscript.

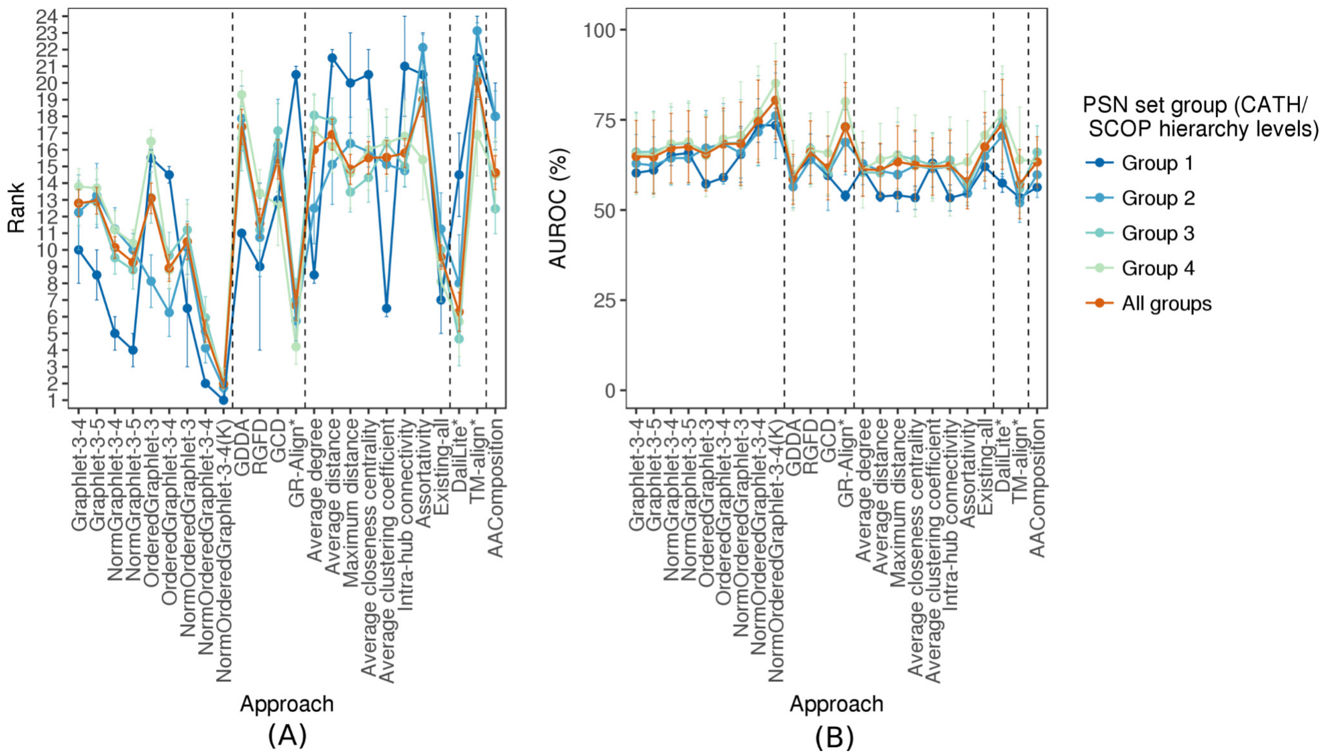

Supplementary Figure S3. The PSN set group-specific performance comparison of the 24 considered approaches, averaged over all PSN sets in the given PSN set group, with respect to AUROC, in terms of: (A) the approaches' ranks compared to one another, and (B) the approaches' raw AUROC values. In panel (A), for a given PSN set, the 24 approaches are ranked from the best (rank 1) to the worst (rank 24). Then, for a given approach, its ranks over all group-specific PSN sets are averaged (the average ranks are denoted by circles, and bars denote the corresponding standard deviations). So, the lower the average rank, the better the approach. In panel (B), for each approach, its group-specific raw AUROC scores are averaged (the average values are denoted by circles, and bars denote the corresponding standard deviations). So, the higher the average AUROC value, the better the approach. The trends are very similar with respect to AUPR as well (Fig. 7 in the main manuscript). These results are for the best PSN construction strategy. Equivalent results for each of the PSN construction strategies are shown in Supplementary Fig. S4-S7.

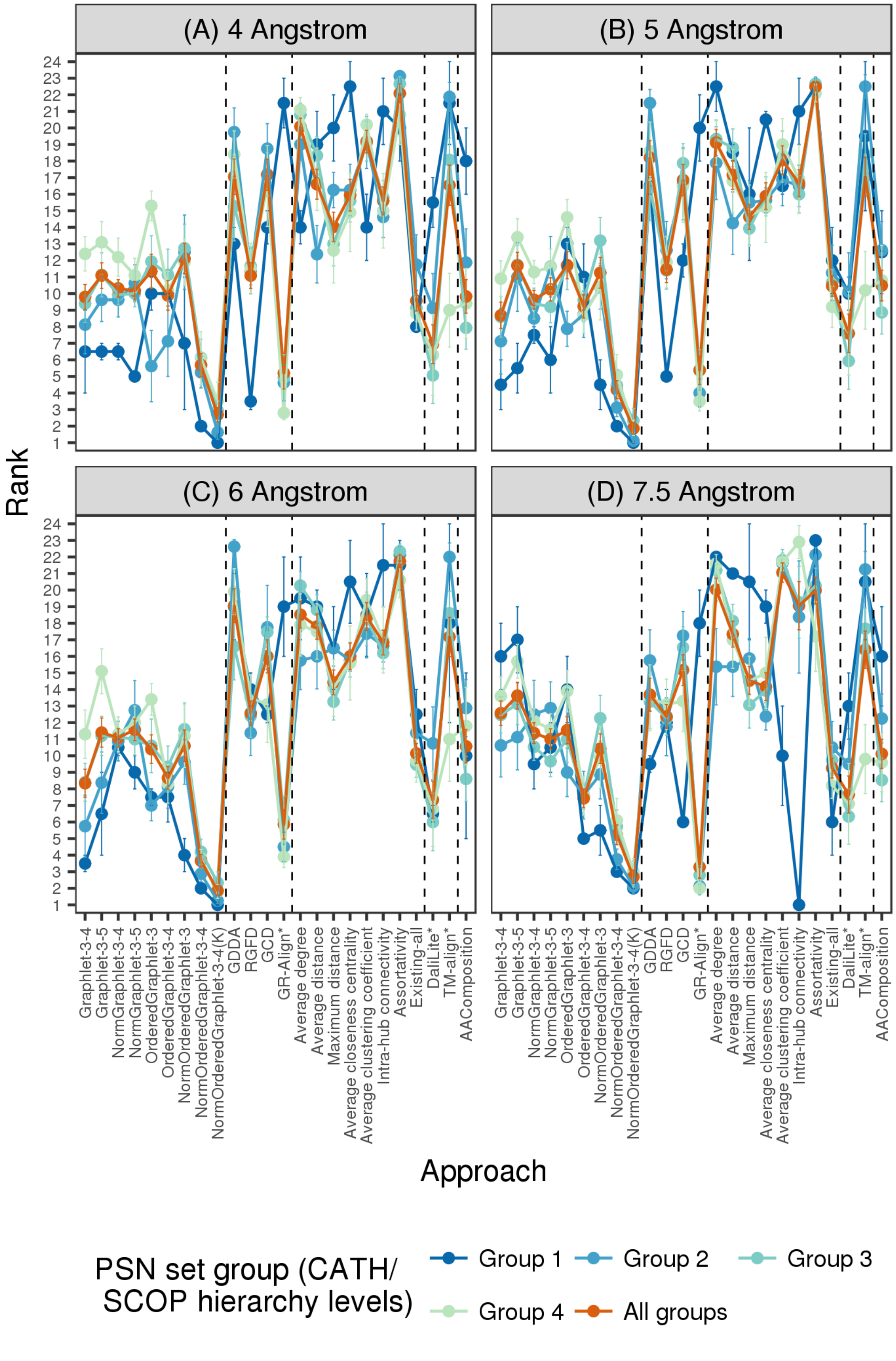

Supplementary Figure S4. The PSN set group-specific rank performance comparison of the 24 considered approaches, averaged over all PSN sets in the given PSN set group, with respect to AUPR, corresponding to the (A) first (any heavy atom, 4 Å), (B) second (any heavy atom, 5 Å), (C) third (any heavy atom, 6 Å), and (D) fourth (α-carbon heavy atom, 7.5 Å) PSN construction strategy. For a given PSN set, the 24 approaches are ranked from the best (rank 1) to the worst (rank 24). Then, for a given approach, its ranks over all group-specific PSN sets are averaged (the average ranks are denoted by circles, and bars denote the corresponding standard deviations). So, the lower the average rank, the better the approach. The trends are very similar with respect to AUROC as well (Supplementary Fig. S5).

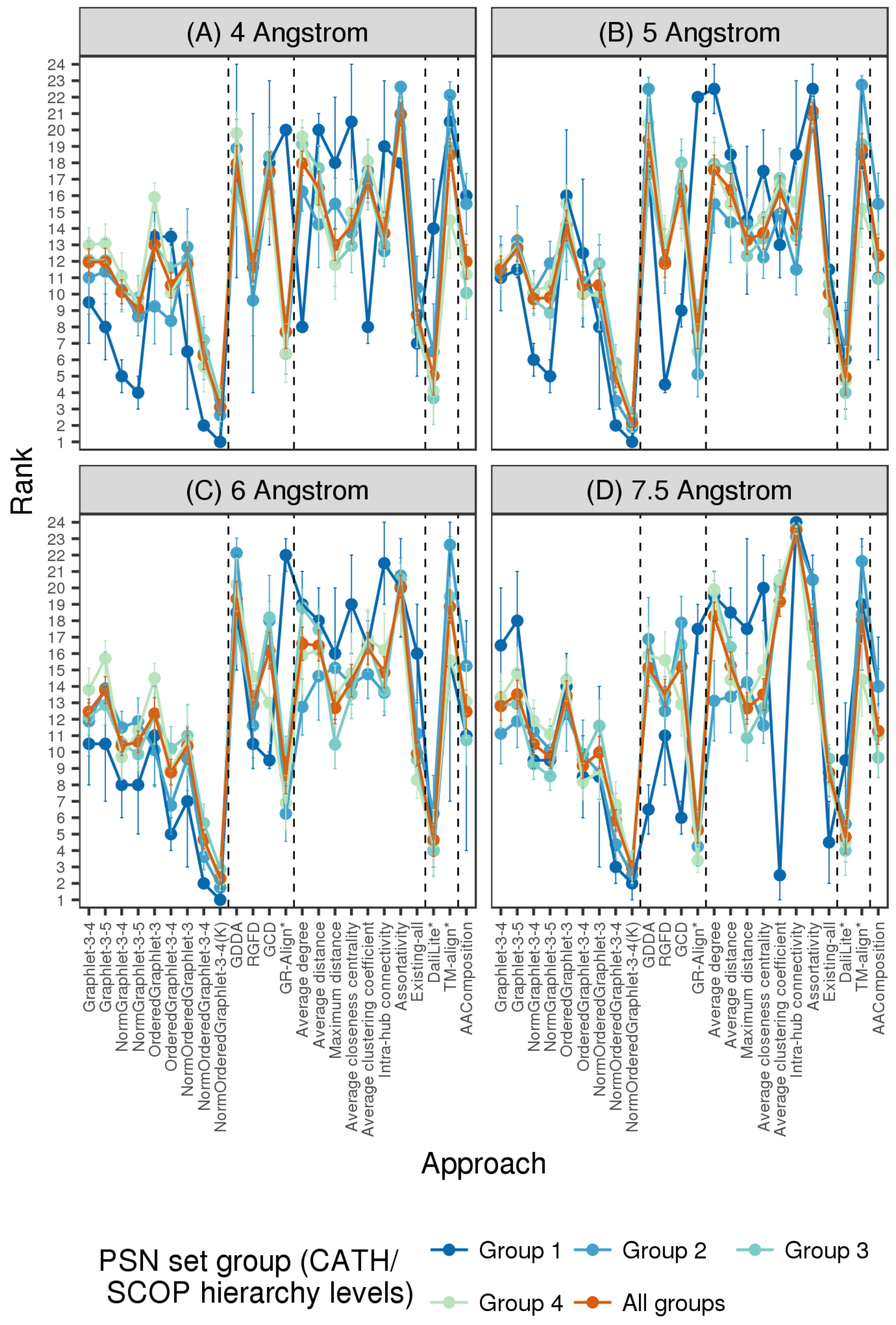

Supplementary Figure S5. The PSN set group-specific rank performance comparison of the 24 considered approaches, averaged over all PSN sets in the given PSN set group, with respect to AUROC, corresponding to the (A) first (any heavy atom, 4 Å), (B) second (any heavy atom, 5 Å), (C) third (any heavy atom, 6 Å), and (D) fourth (α-carbon heavy atom, 7.5 Å) PSN construction strategy. For a given PSN set, the 24 approaches are ranked from the best (rank 1) to the worst (rank 24). Then, for a given approach, its ranks over all group-specific PSN sets are averaged (the average ranks are denoted by circles, and bars denote the corresponding standard deviations). So, the lower the average rank, the better the approach. The trends are very similar with respect to AUPR as well (Supplementary Fig. S4).

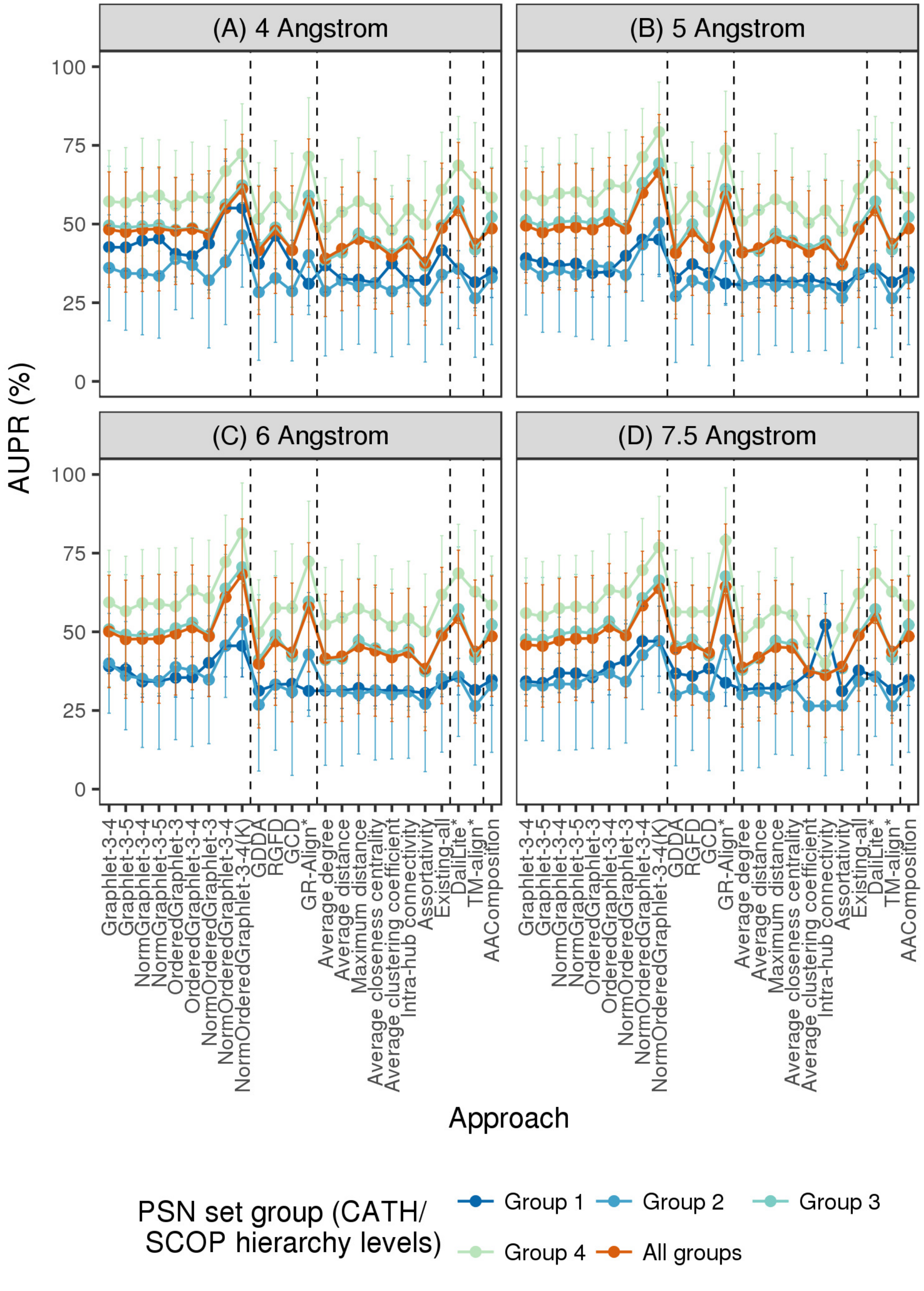

Supplementary Figure S6. The PSN set group-specific performance comparison of the 24 considered approaches, averaged over all PSN sets in the given PSN set group, with respect to AUPR values (expressed as percentages), corresponding to the (A) first (any heavy atom, 4 Å), (B) second (any heavy atom, 5 Å), (C) third (any heavy atom, 6 Å), and (D) fourth (α-carbon heavy atom, 7.5 Å) PSN construction strategy. For each approach, its group-specific raw AUPR values are averaged (the average values are denoted by circles, and bars denote the corresponding standard deviations). So, the higher the average AUPR value, the better the approach. The trends are very similar with respect to AUROC as well (Supplementary Fig. S7).

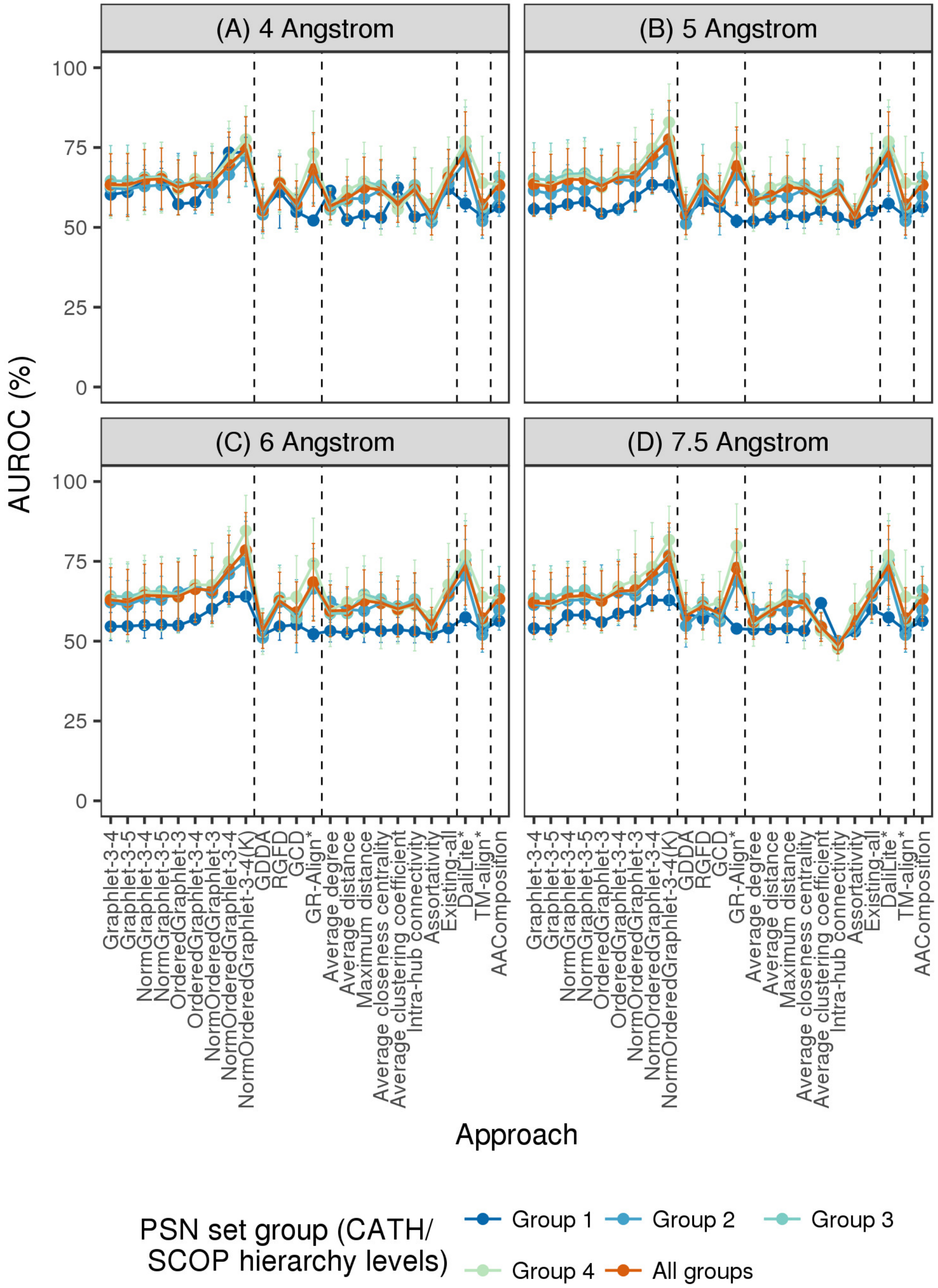

Supplementary Figure S7. The PSN set group-specific performance comparison of the 24 considered approaches, averaged over all PSN sets in the given PSN set group, with respect to AUROC values (expressed as percentages), corresponding to the (A) first (any heavy atom, 4 Å), (B) second (any heavy atom, 5 Å), (C) third (any heavy atom, 6 Å), and (D) fourth (α-carbon heavy atom, 7.5 Å) PSN construction strategy. For each approach, its group-specific raw AUROC values are averaged (the average values are denoted by circles, and bars denote the corresponding standard deviations). So, the higher the average AUROC value, the better the approach. The trends are very similar with respect to AUPR as well (Supplementary Fig. S6).

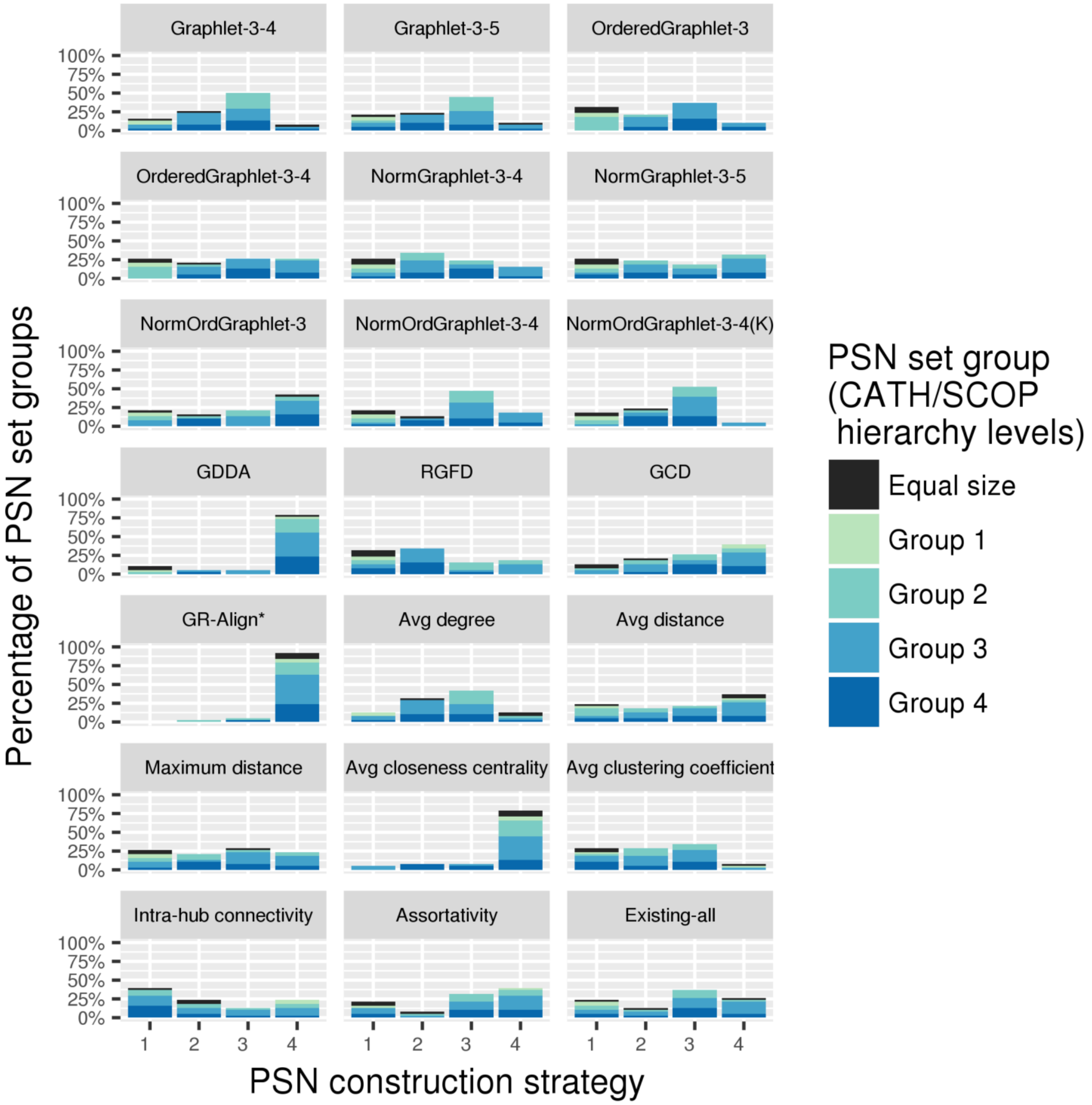

Supplementary Figure S8. Distribution of PSN sets across four PSN construction strategies: 1, 2, 3, and 4. The results are with respect to AUPR. Each panel (one panel per PC approach) shows the following: for each PSN construction strategy, we calculate the percentage of all $3+35=38$ real-world PSN sets for which the given PSN construction strategy performs the best; this is what the height of the given bar shows. Then, within each bar, we label the PSN sets according to the PSN set groups to which they belong.

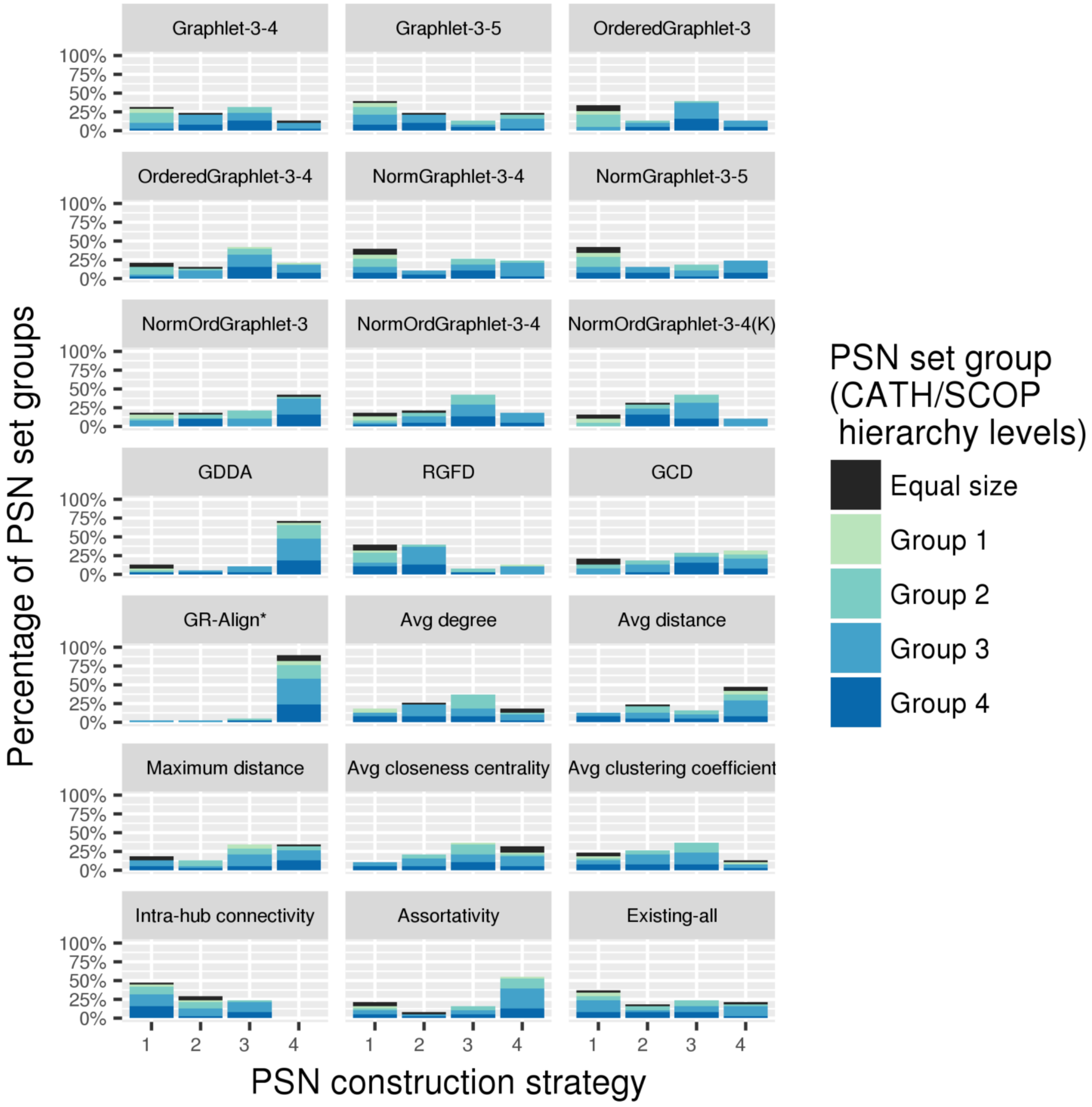

Supplementary Figure S9. Distribution of PSN sets across four PSN construction strategies: 1, 2, 3, and 4. The results are with respect to AUROC. Each panel (one panel per PC approach) shows the following: for each PSN construction strategy, we calculate the percentage of all $3+35=38$ real-world PSN sets for which the given PSN construction strategy performs the best; this is what the height of the given bar shows. Then, within each bar, we label the PSN sets according to the PSN set groups to which they belong.

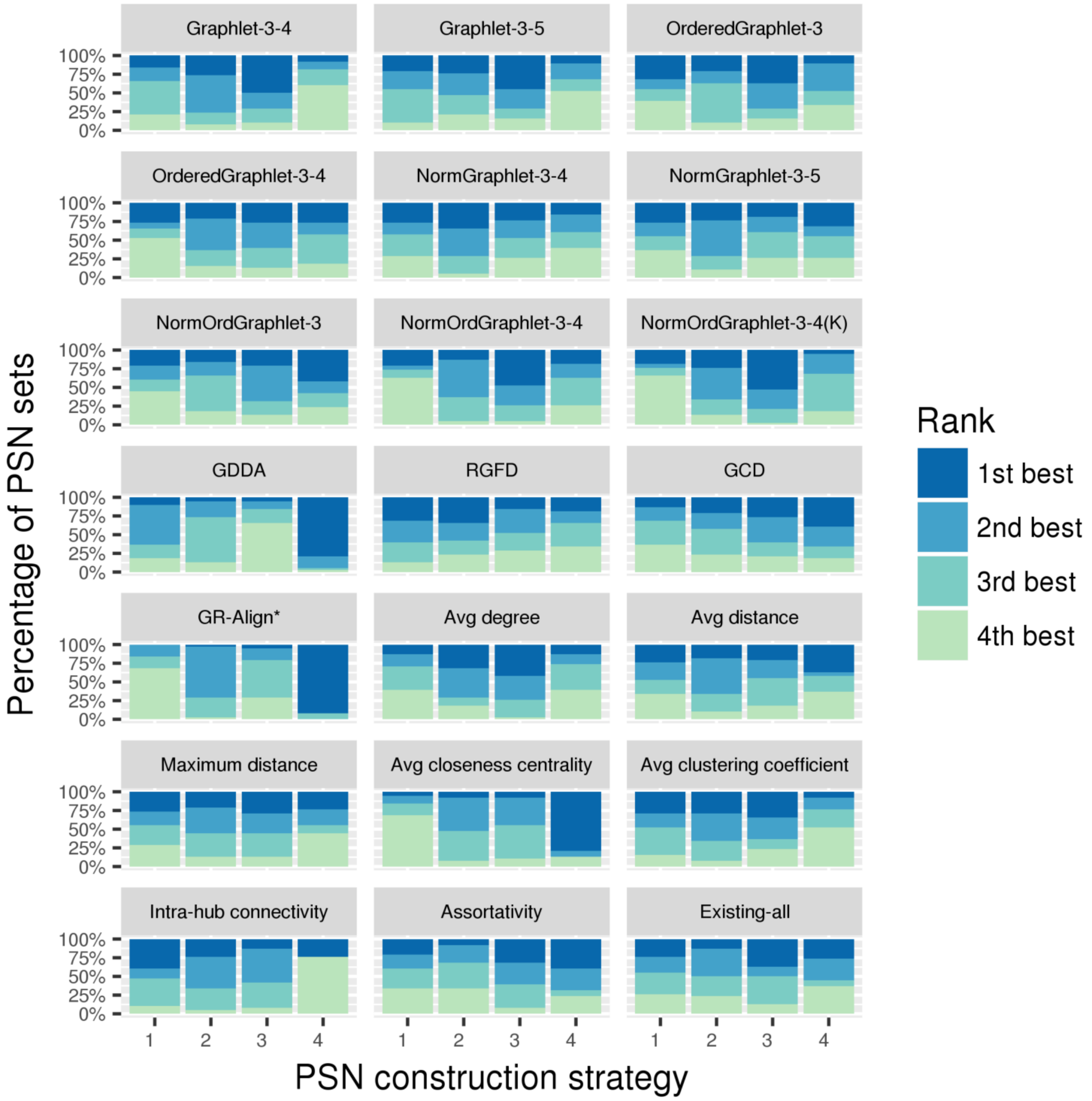

Supplementary Figure S10. The ranking of the four PSN construction strategies: 1, 2, 3, and 4. The ranking is shown with respect to AUPR. Each panel (one panel per PC approach) shows the following: for each PSN construction strategy, we calculate the percentage of all $3+35=38$ real-world PSN sets in which the given PSN construction strategy performs the best, the second best, the third best, and the fourth best.

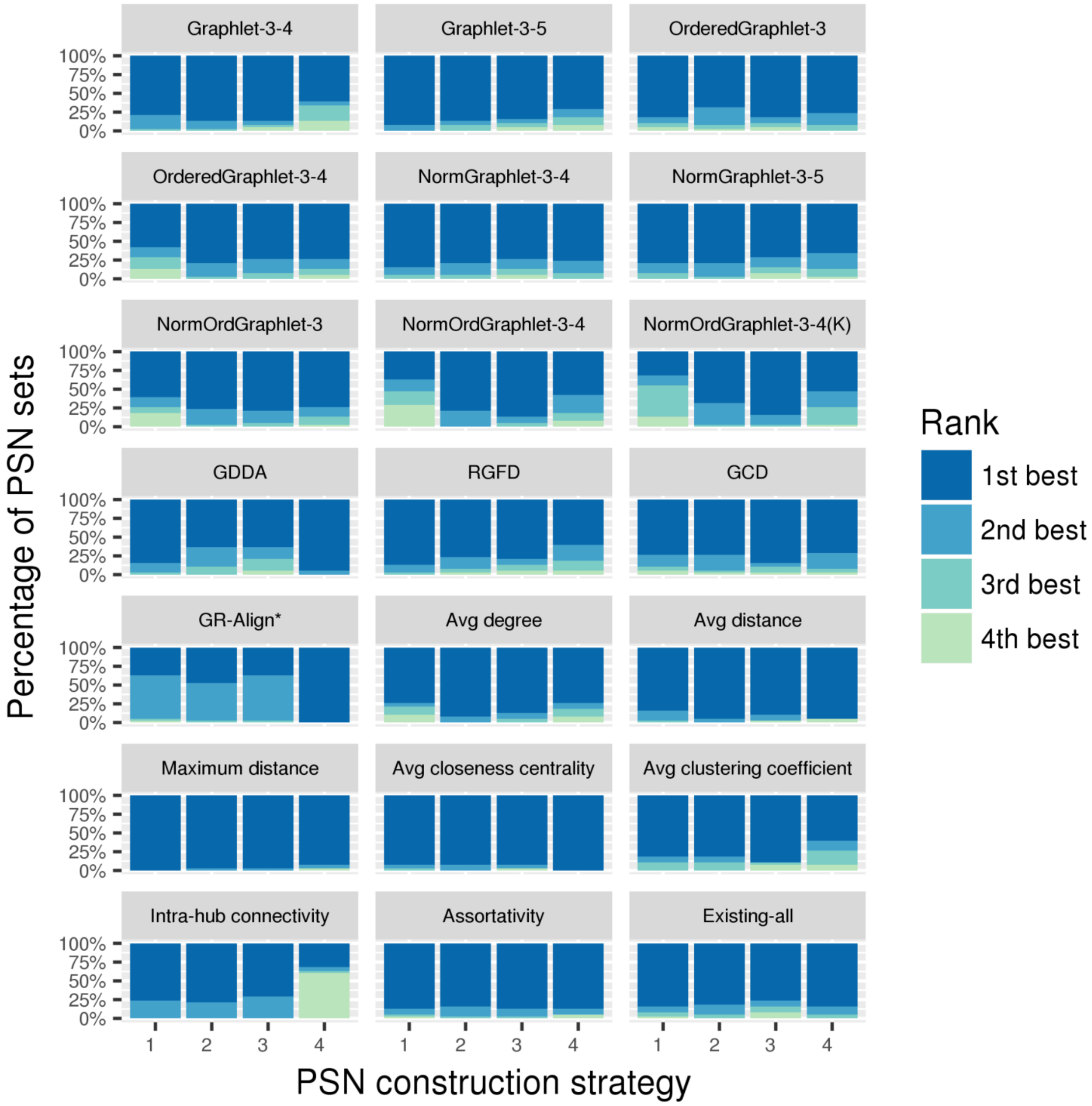

Supplementary Figure S11. The ranking of the four PSN construction strategies: 1, 2, 3, and 4. The ranking is shown with respect to AUPR. Each panel (one panel per PC approach) shows the following: for each PSN construction strategy, we calculate the percentage of all $3+35=38$ real-world PSN sets in which the given PSN construction strategy performs the best, the second best, the third best, and the fourth best. Note that unlike in Supplementary Fig. S10, here we consider two AUPR values to be tied if the absolute difference between them is $\leq 5\%$ of the maximum achievable AUPR value.

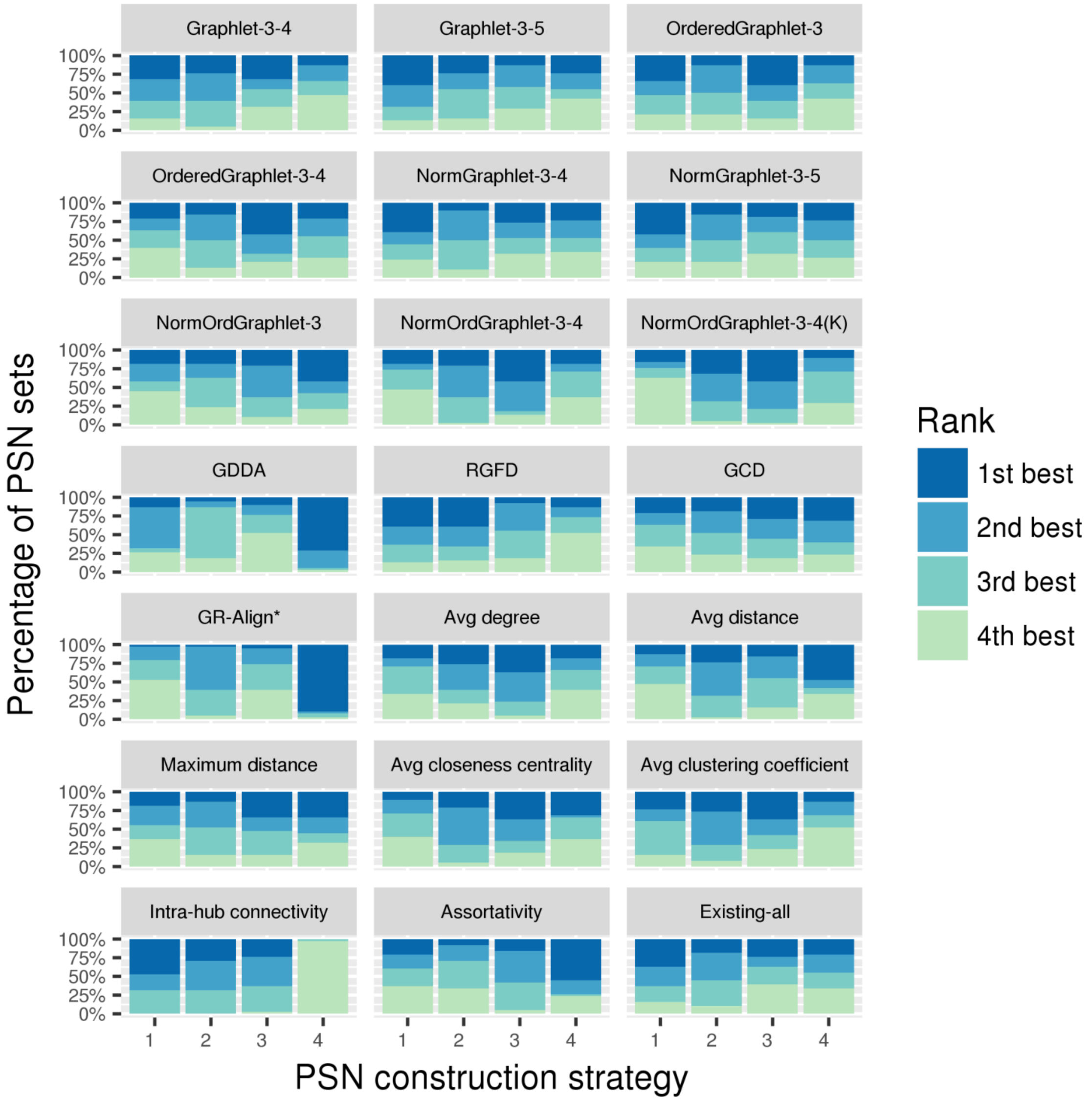

Supplementary Figure S12. The ranking of the four PSN construction strategies: 1, 2, 3, and 4. The ranking is shown with respect to AUROC. Each panel (one panel per PC approach) shows the following: for each PSN construction strategy, we calculate the percentage of all $3+35=38$ real-world PSN sets in which the given PSN construction strategy performs the best, the second best, the third best, and the fourth best.

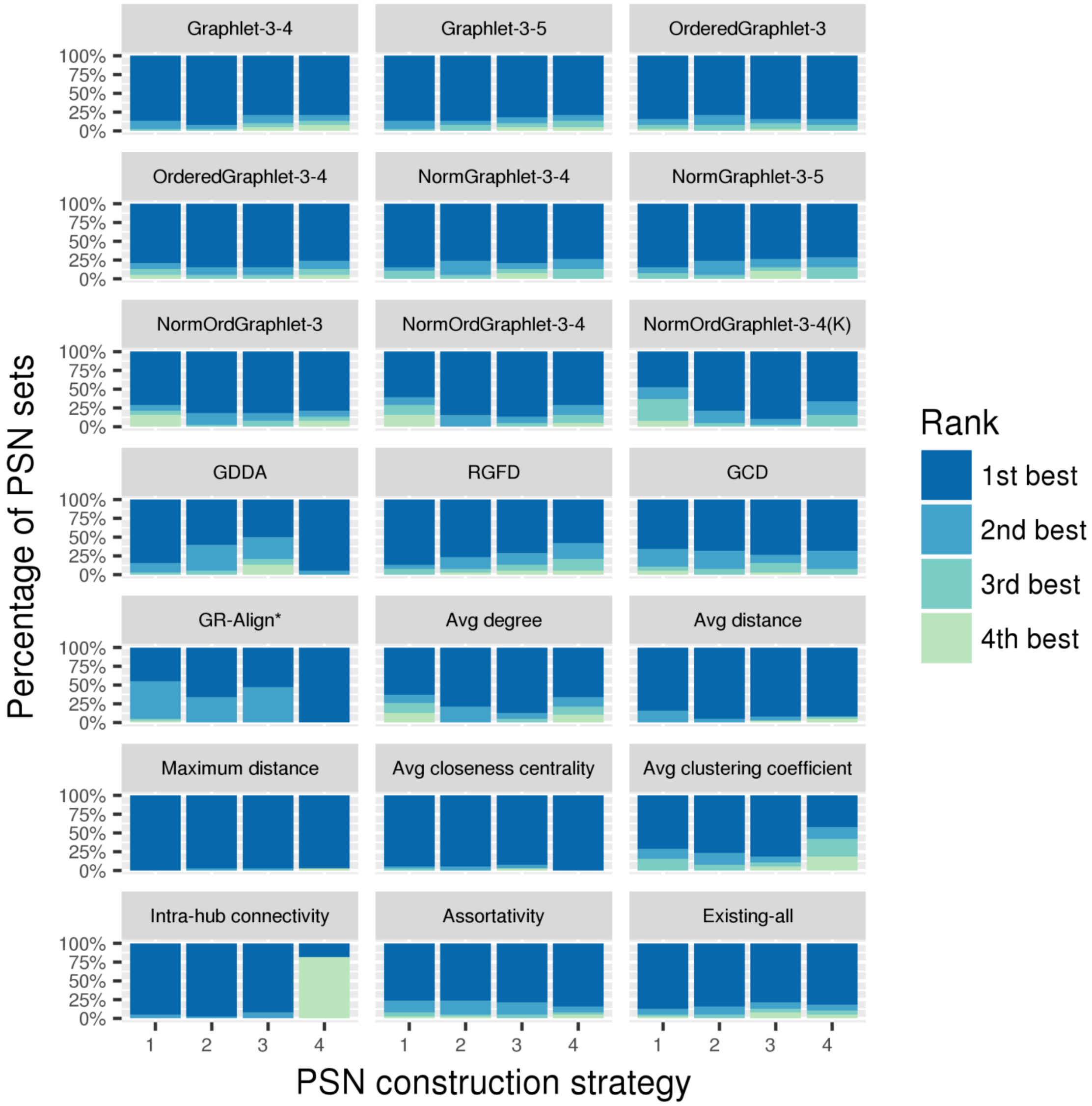

Supplementary Figure S13. The ranking of the four PSN construction strategies: 1, 2, 3, and 4. The ranking is shown with respect to AUROC. Each panel (one panel per PC approach) shows the following: for each PSN construction strategy, we calculate the percentage of all $3+35=38$ real-world PSN sets in which the given PSN construction strategy performs the best, the second best, the third best, and the fourth best. Note that unlike in Supplementary Fig. S12, here we consider two AUROC values to be tied if the absolute difference between them is $\leq 5\%$ of the maximum achievable AUROC value.

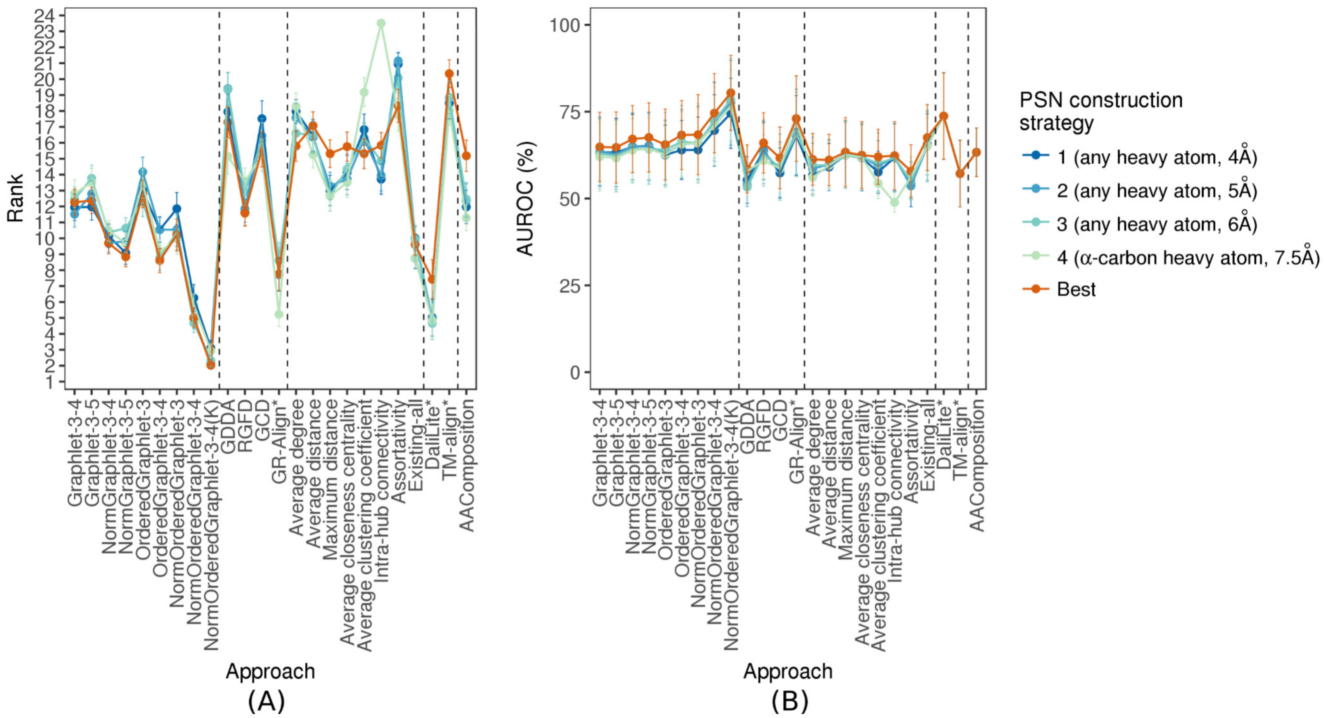

Supplementary Figure S14. The PSN construction strategy-specific performance comparison of the 24 considered PC approaches, with respect to AUROC, in terms of: (A) the approaches' ranks compared to one another, and (B) the approaches' raw AUROC values. In panel (A), for each PSN construction strategy, for a given PSN set, the 24 approaches are ranked from the best (rank 1) to the worst (rank 24). Then, for a given approach, its 35 ranks (corresponding to the 35 PSN sets) are averaged (the average ranks are denoted by circles, and bars denote the corresponding standard deviations). So, the lower the average rank, the better the approach. In panel (B), for each PSN construction strategy, for each approach, its 35 raw AUROC values (corresponding to the 35 PSN sets) are averaged (the average values are denoted by circles, and bars denote the corresponding standard deviations). So, the higher the average AUROC value, the better the approach. The trends are very similar with respect to AUPR as well (Fig. 8 in the main manuscript). These results are for the "all group" PSN set group that spans the 35 PSN sets of different sizes. Equivalent results for the individual groups 1-4 are shown in Supplementary Fig. S15-S18.

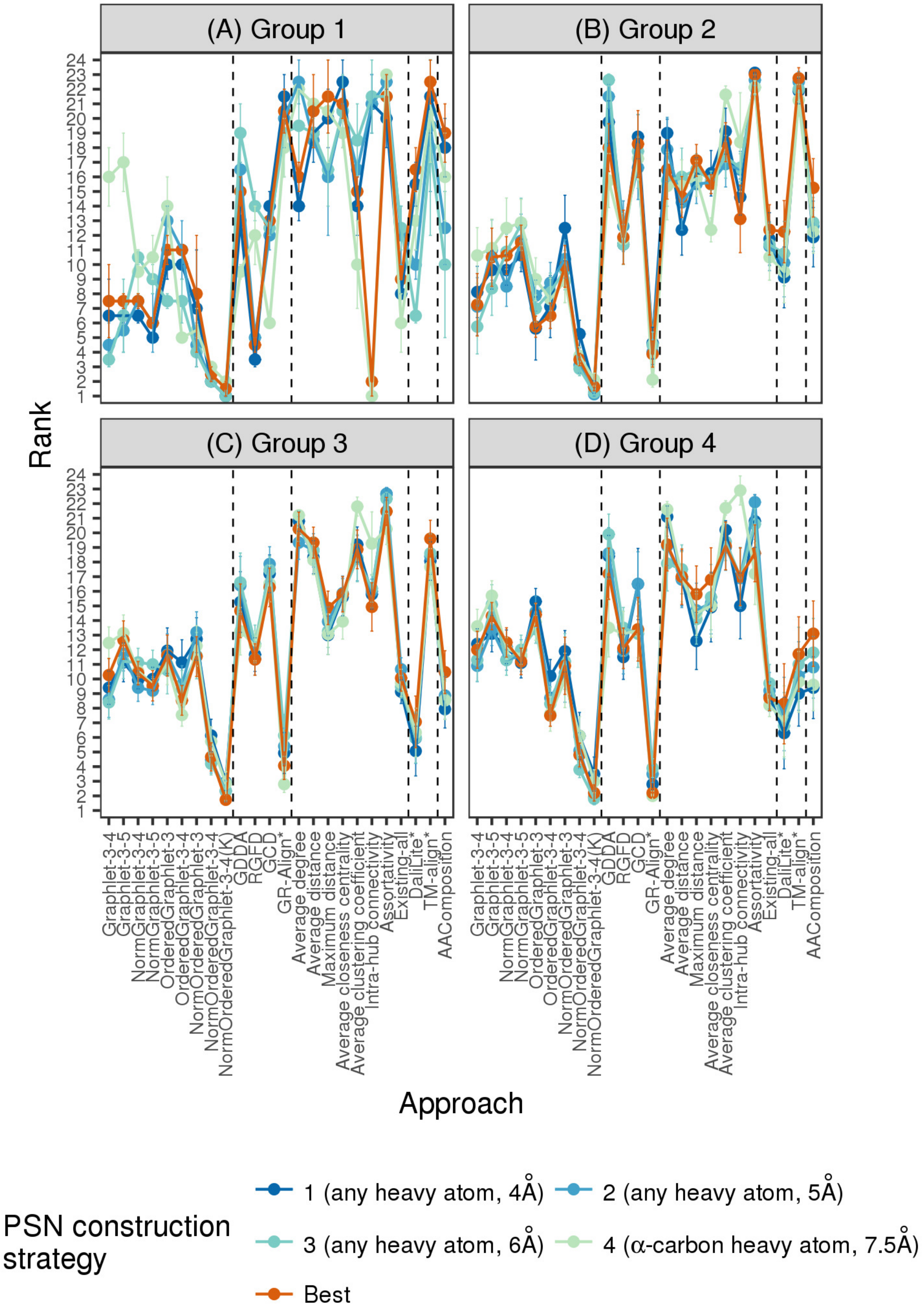

Supplementary Figure S15. The PSN construction strategy-specific rank performance comparison of the 24 considered PC approaches, with respect to AUPR, corresponding to PSN set group : (A) 1, (B) 2, (C) 3, and (D) 4. For each PSN construction strategy, for a given PSN set, the 24 approaches are ranked from the best (rank 1) to the worst (rank 24). Then, for a given approach, its 35 ranks (corresponding to the 35 PSN sets) are averaged (the average ranks are denoted by circles, and bars denote the corresponding standard deviations). So, the lower the average rank, the better the approach. The trends are very similar with respect to AUROC as well (Supplementary Fig. S16).

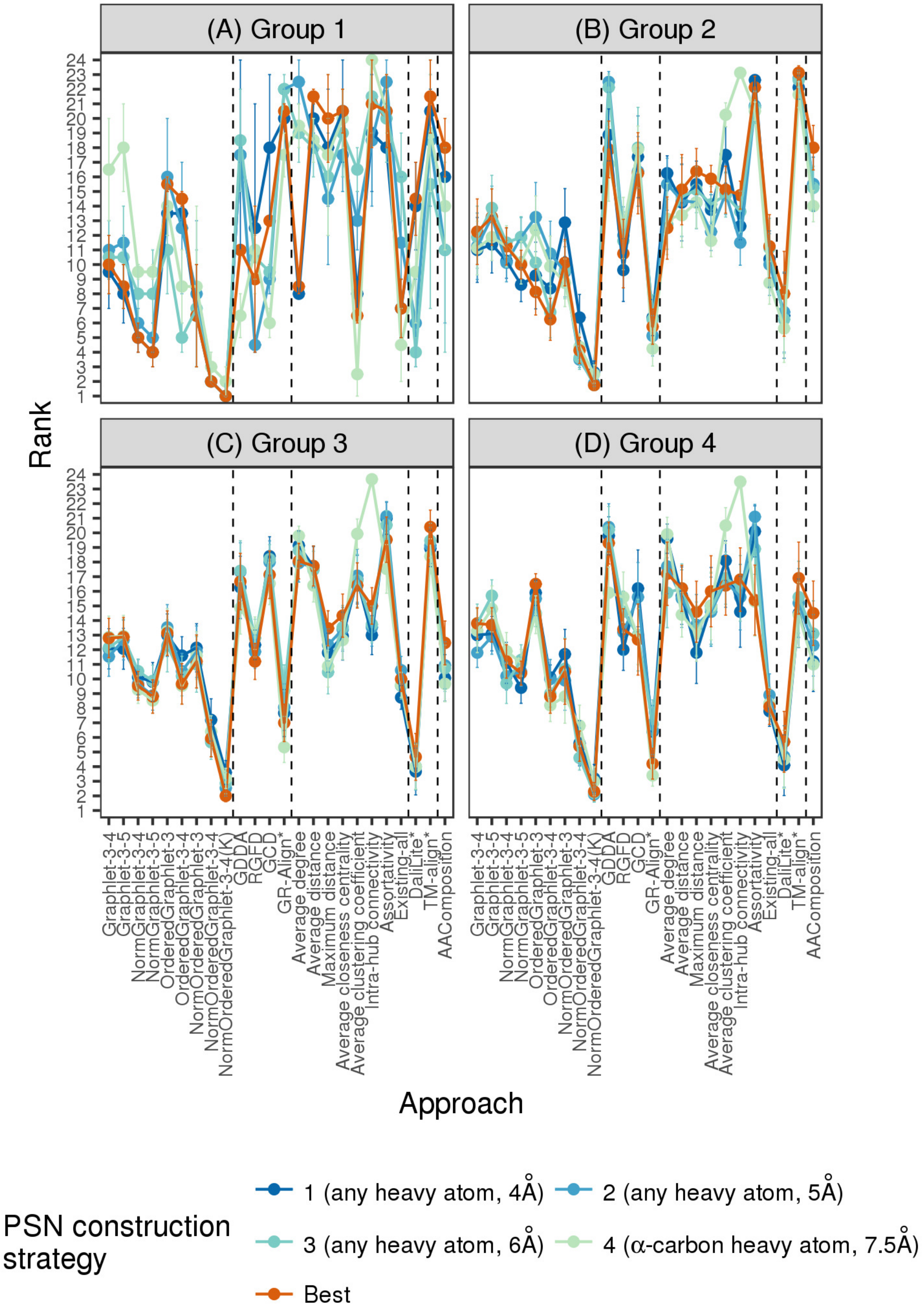

Supplementary Figure S16. The PSN construction strategy-specific rank performance comparison of the 24 considered PC approaches, with respect to AUROC, corresponding to PSN set group : (A) 1, (B) 2, (C) 3, and (D) 4. For each PSN construction strategy, for a given PSN set, the 24 approaches are ranked from the best (rank 1) to the worst (rank 24). Then, for a given approach, its 35 ranks (corresponding to the 35 PSN sets) are averaged (the average ranks are denoted by circles, and bars denote the corresponding standard deviations). So, the lower the average rank, the better the approach. The trends are very similar with respect to AUPR as well (Supplementary Fig. S15).

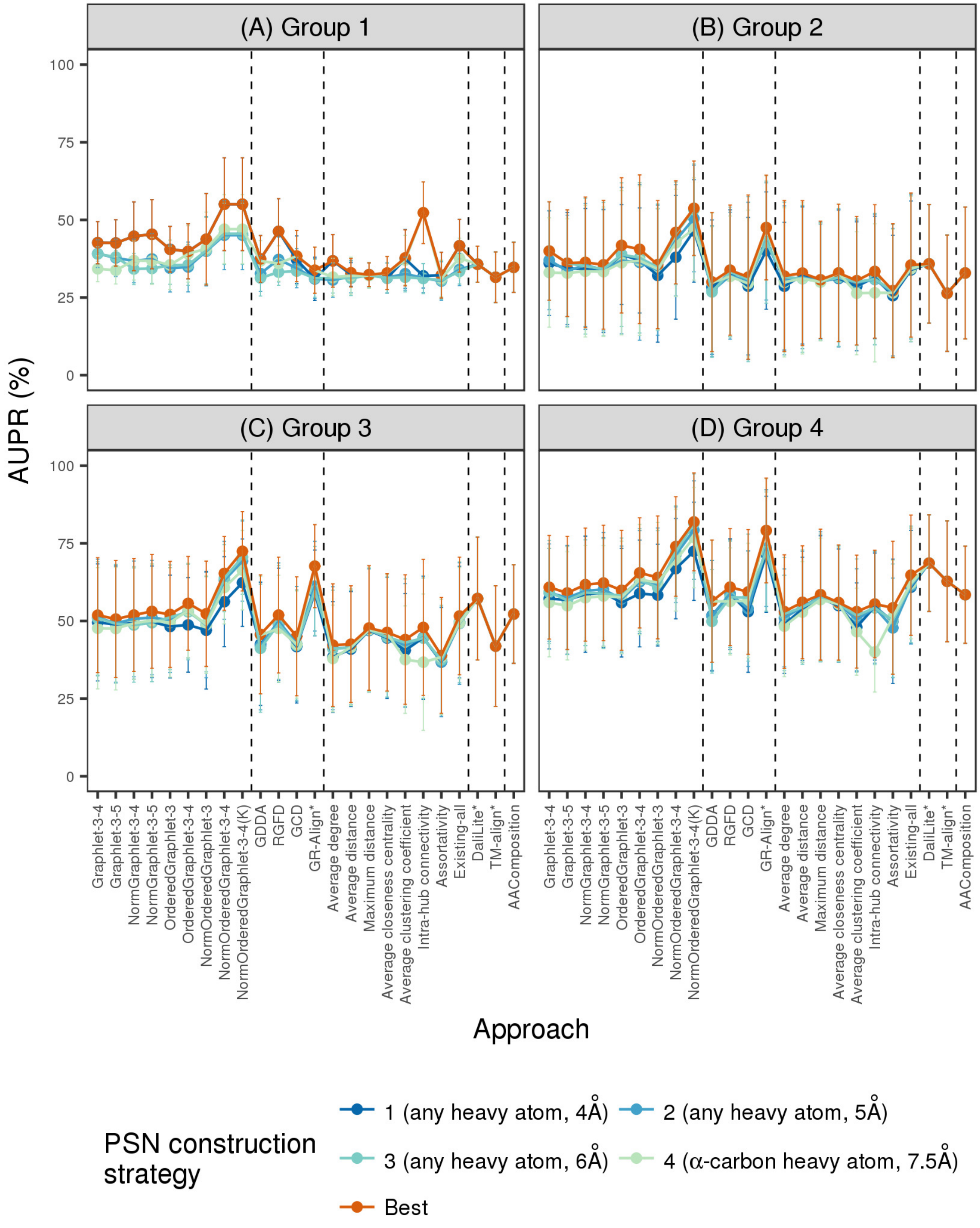

Supplementary Figure S17. The PSN construction strategy-specific performance comparison of the 24 considered PC approaches, with respect to AUPR values (expressed as percentages), corresponding to PSN set group : (A) 1, (B) 2, (C) 3, and (D) 4. For each PSN construction strategy, for each approach, its 35 raw AUPR values (corresponding to the 35 PSN sets) are averaged (the average values are denoted by circles, and bars denote the corresponding standard deviations). So, the higher the average AUPR value, the better the approach. The trends are very similar with respect to AUROC values as well (Supplementary Fig. S18).

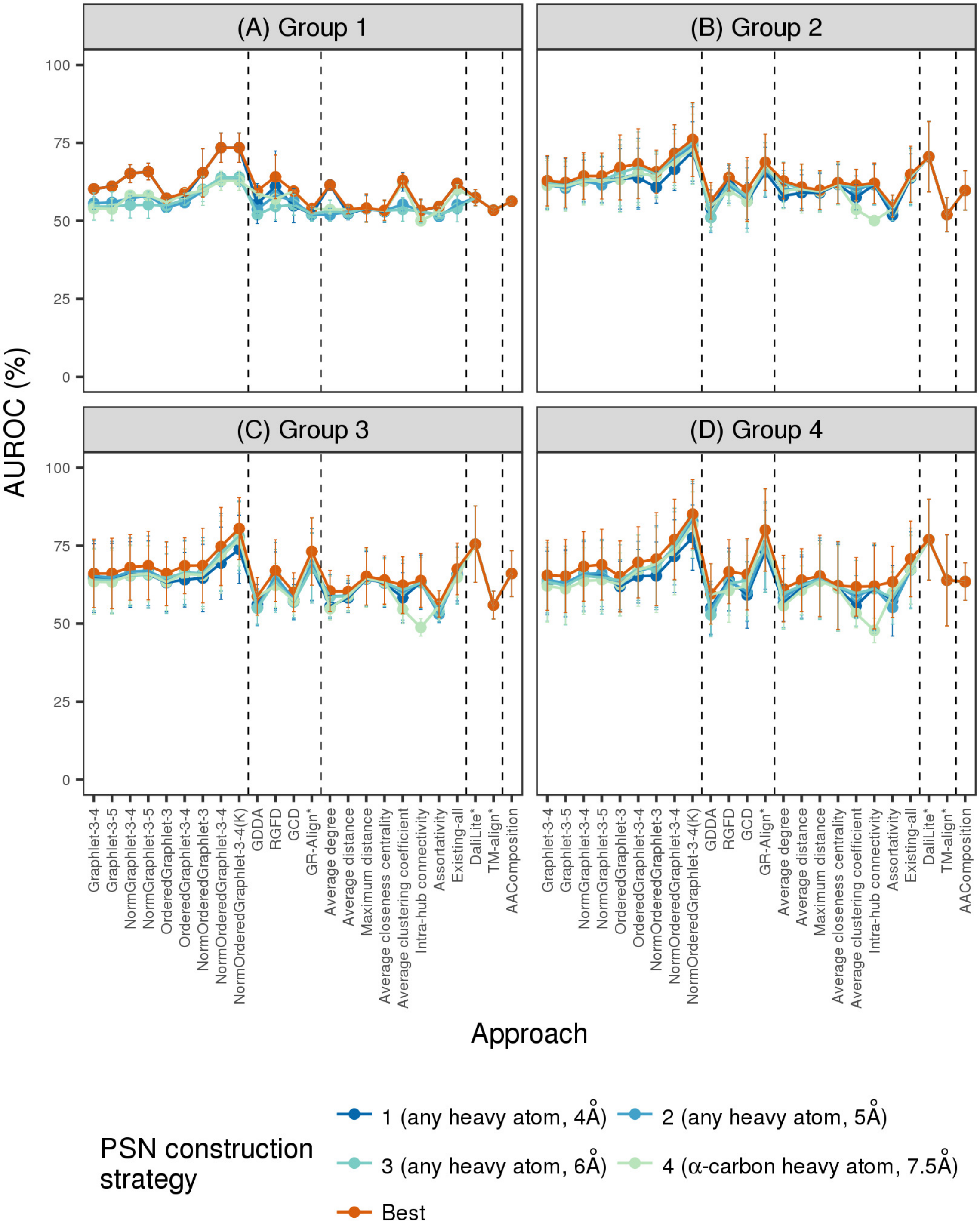

Supplementary Figure S18. The PSN construction strategy-specific performance comparison of the 24 considered PC approaches, with respect to AUROC values (expressed as percentages), corresponding to PSN set group : (A) 1, (B) 2, (C) 3, and (D) 4. For each PSN construction strategy, for each approach, its 35 raw AUROC scores (corresponding to the 35 PSN sets) are averaged (the average values are denoted by circles, and bars denote the corresponding standard deviations). So, the higher the average AUROC value, the better the approach. The trends are very similar with respect to AUPR values as well (Supplementary Fig. S17).

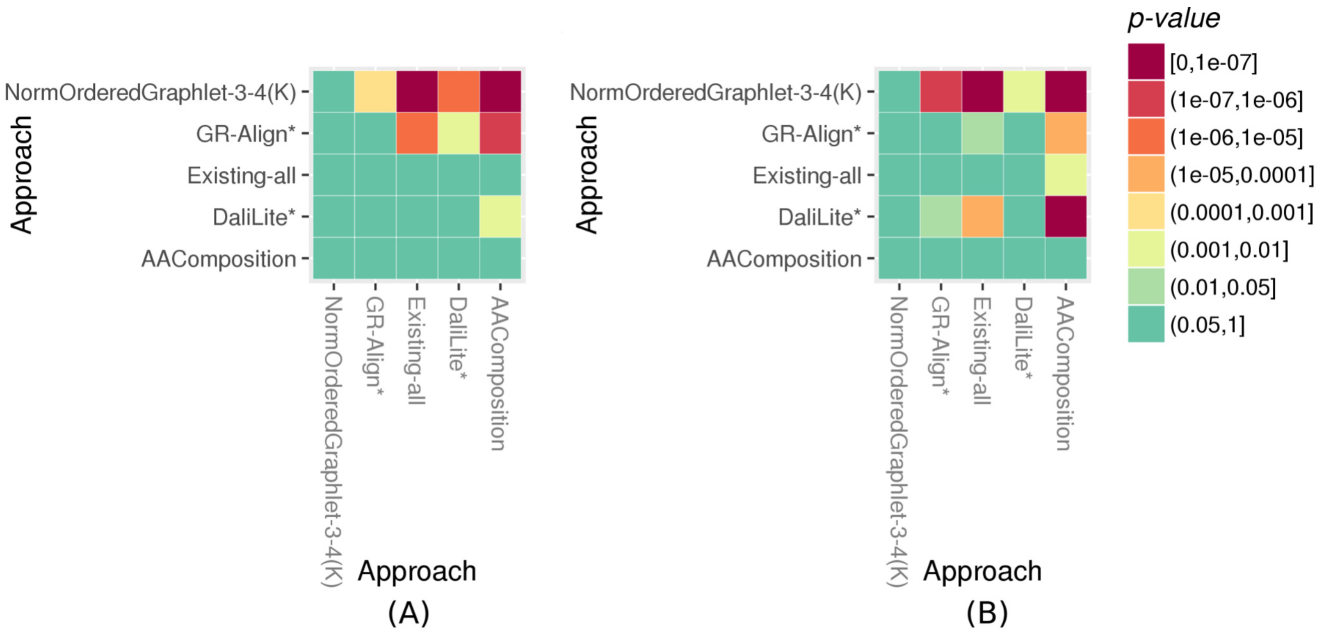

Supplementary Figure S19. Statistical significance of the difference between average ranks of the PC approaches, with respect to: (A) AUPR and (B) AUROC. For aesthetics, these results are only for the best approach in each category, namely: the best of our proposed PCA graphlet-based network approaches (GRAFENE version NormOrderedGraphlet-3-4(K)), the best of the existing non-PCA graphlet-based network approaches (GR-Align), the best of the existing non-graphlet network approaches (Existing-all), the best of the existing non-network 3D structural approaches (DaliLite), and the sequence-based approach (AAComposition). For each of the 35 PSN sets, the five approaches are ranked from the best (rank 1) to the worst (rank 5). Hence, for each approach, there are 35 ranks (corresponding to the 35 PSN sets). For each pair of approaches, we compare the two given approaches' 35 ranks using paired t-test. In the figure, every cell (i, j) indicates the statistical significance (in terms of p-value) of approach i being superior to approach j. The results are similar when we use raw AUPR/AUROC values instead of ranks (Supplementary Fig. S20).

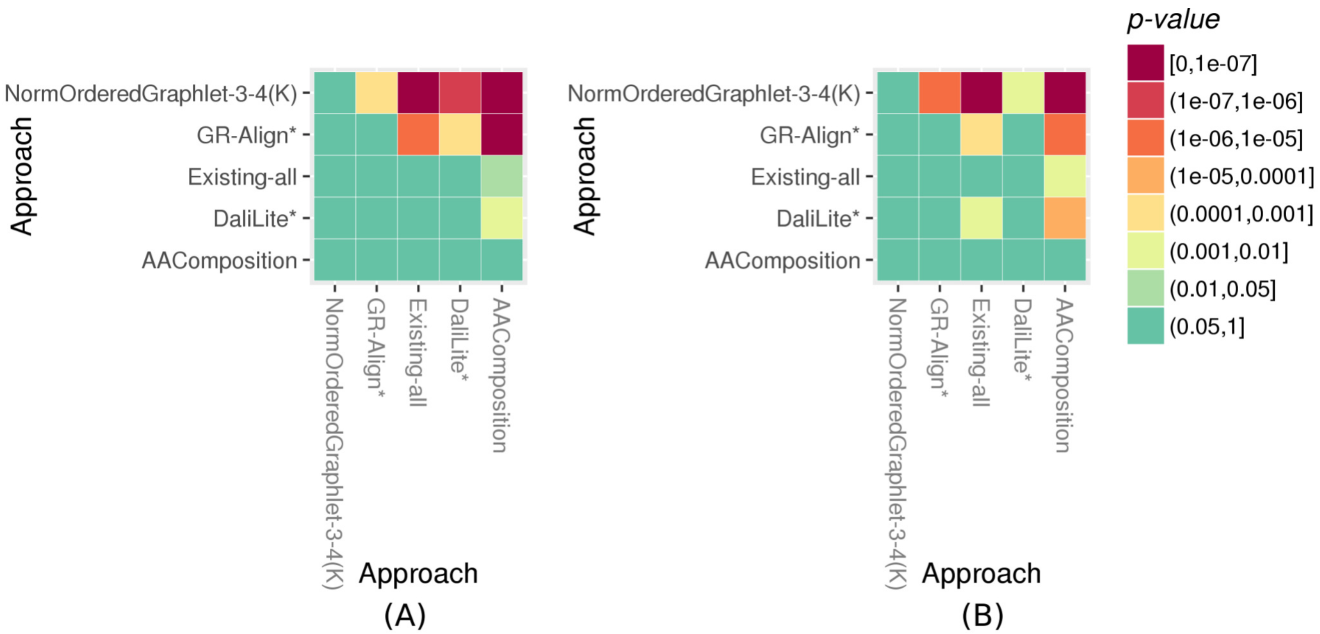

Supplementary Figure S20. Statistical significance of the difference between average raw values of the PC approaches, with respect to: (A) AUPR and (B) AUROC. For aesthetics, these results are only for the best approach in each category, namely: the best of our proposed PCA graphlet-based network approaches (GRAFENE version NormOrderedGraphlet-3-4(K)), the best of the existing non-PCA graphlet-based network approaches (GR-Align), the best of the existing non-graphlet network approaches (Existing-all), the best of the existing non-network 3D structural approaches (DaliLite), and the sequence-based approach (AAComposition). For each of the 35 PSN sets, raw AUPR/AUROC values for all five approaches are measured. Hence, for each approach, there are 35 raw AUPR/AUROC values (corresponding to the 35 PSN sets). For each pair of approaches, we compare the two given approaches' 35 raw AUPR/AUROC values using paired t-test. In the figure, every cell (i, j) indicates the statistical significance (in terms of p-value) of approach i being superior to approach j. The results are similar when we use ranks instead of raw AUPR/AUROC values (Supplementary Fig. S19).

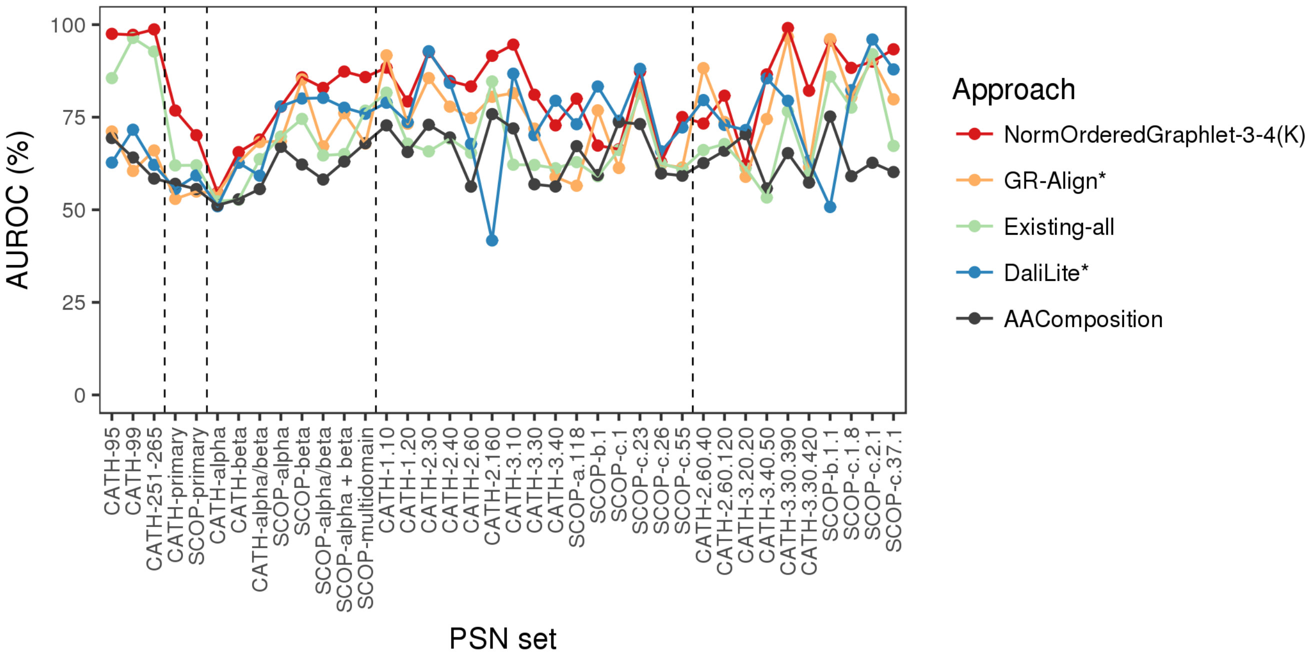

Supplementary Figure S21. The performance comparison of only the best PC approach in each category (for aesthetics purposes) on all three “equal size” PSN sets and all 35 PSN sets of different size, with respect to raw AUROC values. Namely, results are shown for: the best of our proposed PCA graphlet-based network approaches (GRAFENE version NormOrderedGraphlet-3-4(K)), the best of the existing non-PCA graphlet-based network approaches (GR-Align), the best of the existing non-graphlet network approaches (Existing-all), the best of the existing non-network 3D structural approaches (DaliLite), and the sequence-based approach (AAComposition). The vertical dotted lines separate the PSN sets into the five PSN set groups, namely (from left to right): “equal size”, group 1, group 2, group 3, and group 4. For the equivalent results in terms of raw AUPR values, see Fig. 9 in the main manuscript.

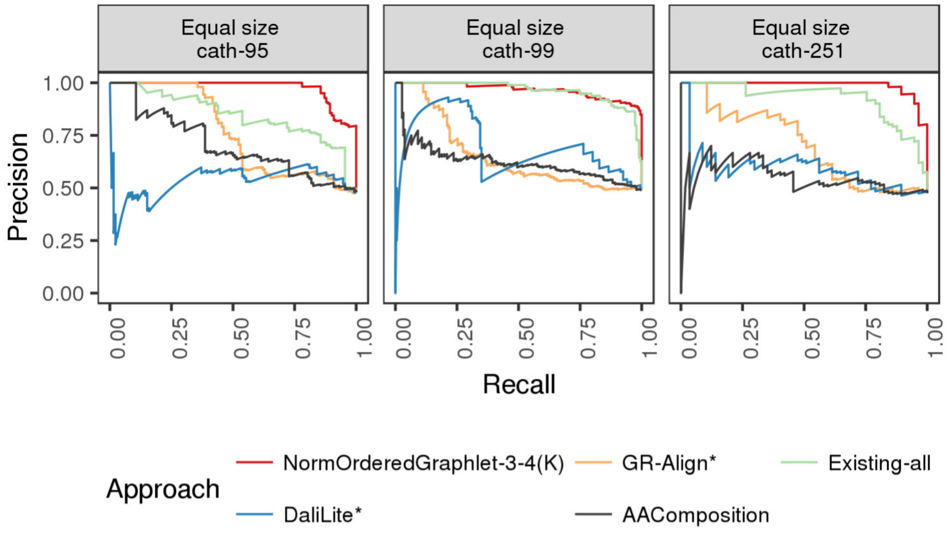

(A)

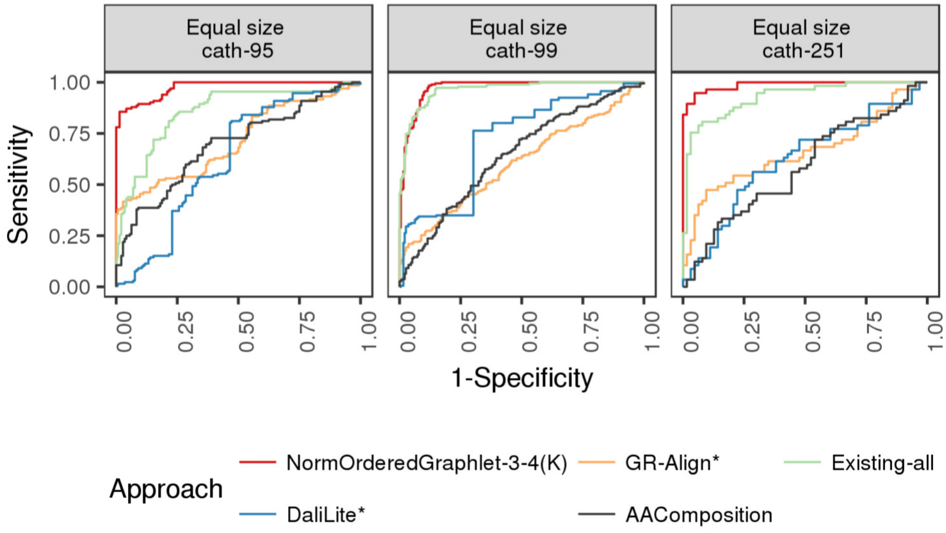

(B)

Supplementary Figure S22. (A) Precision-recall (PR) and (B) receiver operating characteristic (ROC) curves for the best approach in each category, namely: the best of our proposed PCA graphlet-based network approaches (GRAFENE version NormOrderedGraphlet-3-4(K)), the best of the existing non-PCA graphlet-based network approaches (GR-Align), the best of the existing non-graphlet network approaches (Existing-all), the best of the existing non-network 3D structural approaches (DaliLite), and the sequence-based approach (AAComposition). The results are for the three "equal-size" PSN sets. Also, these results are for the best PSN construction strategy.

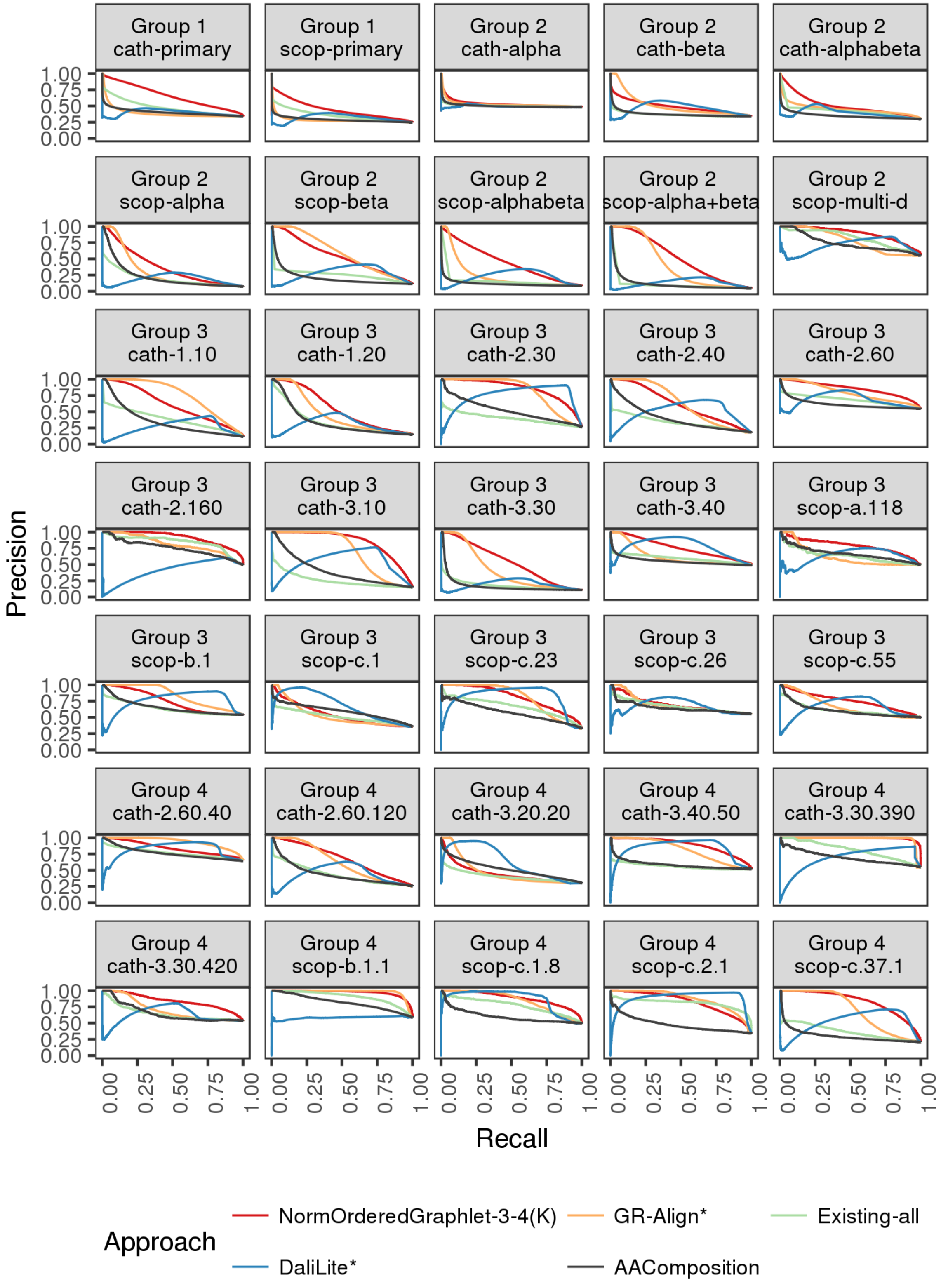

Supplementary Figure S23. Precision-recall (PR) curves for the best approach in each category, namely: the best of our proposed PCA graphlet-based network approaches (GRAFENE version NormOrderedGraphlet-3-4(K)), the best of the existing non-PCA graphlet-based network approaches (GR-Align), the best of the existing non-graphlet network approaches (Existing-all), the best of the existing non-network 3D structural approaches (DaliLite), and the sequence-based approach (AAComposition). These results are for the 35 PSN sets of different size. Also, these results are for the best PSN construction strategy.

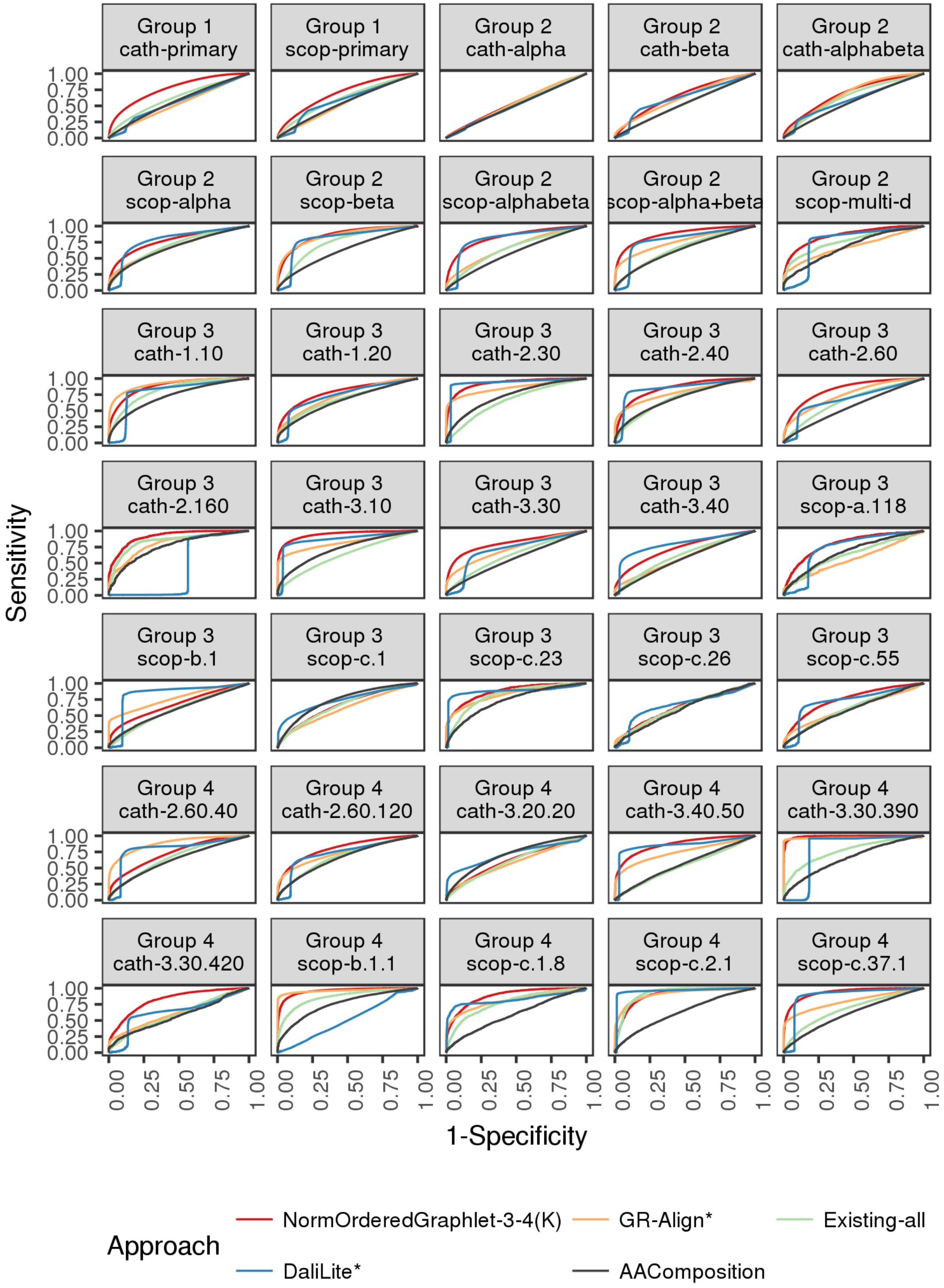

Supplementary Figure S24. Receiver operating characteristic (ROC) curves for the best approach in each category, namely: the best of our proposed PCA graphlet-based network approaches (GRAFENE version NormOrderedGraphlet-3-4(K)), the best of the existing non-PCA graphlet-based network approaches (GR-Align), the best of the existing non-graphlet network approaches (Existing-all), the best of the existing non-network 3D structural approaches (DaliLite), and the sequence-based approach (AAComposition). These results are for the 35 PSN sets of different size. Also, these results are for the best PSN construction strategy.

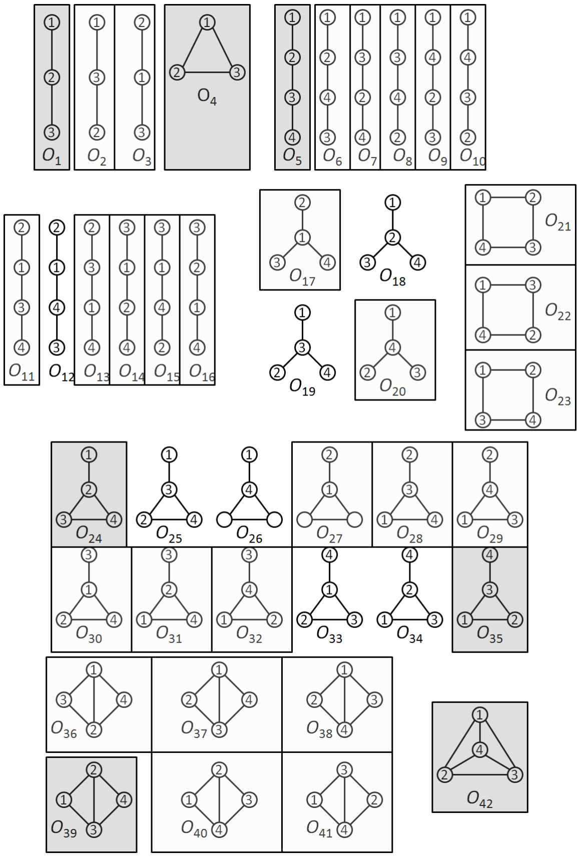

Supplementary Figure S25. Ordered graphlets that are significantly represented in α (dark gray) or β (light gray) PSNs.

III Supplementary Tables

Supplementary Table S1. Synthetic network sets that we use. For the given data set, the second column indicates whether its networks are of the same size or different sizes, and the last three columns indicate the number of its networks as well as their size(s) in terms of the number of nodes and edges.

Data set			Number of		
Type	Size	Name	Networks	Nodes	Edges
Synthetic networks	Same	Synthetic-100	150	100	400
		Synthetic-500	150	500	2,000
		Synthetic-1000	150	1,000	4,000
	Different	Synthetic-all	450	100-1,000	400-4,000

Supplementary Table S2. The number of categories and the number of PSNs averaged over all categories for each of the 35 real-world PSN sets, with respect to four different PSN construction strategies: first (any heavy atom, 4 Å), second (any heavy atom, 5 Å), third (any heavy atom, 6 Å), and fourth (α-carbon heavy atom, 7.5 Å).

	PSN construction strategy 1		PSN construction strategy 2		PSN construction strategy 3		PSN construction strategy 4	
	# of categories	Avg # of PSNs/category	# of categories	Avg # of PSNs/category	# of categories	Avg # of PSNs/category	# of categories	Avg # of PSNs/category
CATH-primary	3	3170	3	3167	3	3133	3	3153
CATH-α	4	655	4	656	4	650	4	541
CATH-β	10	297	10	297	10	295	10	295
CATH-α/β	4	947	4	947	4	935	4	944
CATH-1.10	12	72	12	72	12	71	12	72
CATH-1.20	8	60	8	59	8	59	8	59
CATH-2.30	4	51	4	51	4	51	4	51
CATH-2.40	7	76	7	76	7	75	7	74
CATH-2.60	2	717	2	718	2	716	2	716
CATH-2.160	2	35	2	35	2	35	2	35
CATH-3.10	7	62	7	62	7	61	7	61
CATH-3.30	14	79	14	79	14	79	14	78
CATH-3.40	3	212	3	212	3	203	3	212
CATH-2.60.40	3	212	3	212	3	210	3	212
CATH-2.60.120	4	92	4	93	4	93	4	92
CATH-3.20.20	5	123	5	123	5	123	5	123
CATH-3.30.390	2	44	2	44	2	44	2	44
CATH-3.30.420	2	78	2	78	2	78	2	78
CATH-3.40.50	2	145	2	145	2	145	2	145
SCOP-primary	7	1636	7	1638	7	1628	7	1624
SCOP-α	16	57	16	58	16	58	16	57
SCOP-β	21	88	21	88	21	88	21	88
SCOP-α/β	26	113	26	114	26	112	26	113
SCOP-$\alpha+\beta$	28	57	28	57	28	57	28	57
SCOP-multidomain	2	63	2	63	2	63	2	63
SCOP-a.118	2	35	2	35	2	35	2	35
SCOP-b.1	3	144	3	144	3	144	3	144
SCOP-c.1	4	75	4	75	4	75	4	75
SCOP-c.23	3	36	3	36	3	34	3	35
SCOP-c.26	2	47	2	47	2	47	2	47
SCOP-c.55	2	90	2	90	2	90	2	90
SCOP-b.1.1	2	141	2	141	2	141	2	141
SCOP-c.1.8	2	54	2	54	2	54	2	54
SCOP-c.2.1	4	54	4	54	4	54	4	54
SCOP-c.37.1	6	55	6	54	6	54	6	54

Supplementary Table S3. Details about our PSN sets belonging to the second-level hierarchical categories of CATH and SCOP. At the top-level of the CATH hierarchy, there are three categories: α, β, and α/β. At the top-level of the SCOP hierarchy, there are five categories: α, β, α/β, α+β, and Multi domain. Each top-level category has multiple second-level categories, as shown in the table. For example, the α top-level hierarchical category of CATH has four second-level categories: Orthogonal Bundle, Up-down Bundle, Alpha Horseshoe, and Alpha/Alpha Barrel. For each top-level hierarchical category, we specify its name and label (separated by semicolon), where the labels are as given by CATH/SCOP. For each second-level hierarchical category, we specify its name and the number of PSNs (shown in parentheses).

	**Top-level hierarchical categories**	**Second-level hierarchical categories**
**CATH**	α; 1	1. Orthogonal Bundle (1632)<br>2. Up-down Bundle (807)<br>3. Alpha Horseshoe (133)<br>4. Alpha/Alpha Barrel (53)
	β; 2	1. Ribbon (44)<br>2. Roll (242)<br>3. Beta Barrel (699)<br>4. Sandwich (1562)<br>5. Distorted Sandwich (102)<br>6. Trefoil (79)<br>7. 6 Propellor (45)<br>8. 7 Propellor (42)<br>9. 3 Solenoid (70)<br>10. Beta Complex (87)
	α/β; 3	1. Roll (611)<br>2. Alpha-Beta Barrel (839)<br>3. 2-Layer Sandwich (1668)<br>4. 3-Layer(aba) Sandwich (675)
**SCOP**	α; a	1. Globin-like (95)<br>2. Cytochrome c (35)<br>3. DNA/RNA-binding 3-helical bundle (113)<br>4. Spectrin repeat-like (41)<br>5. Four-helical up-and-down bundle (76)<br>6. Ferritin-like (66)<br>7. 4-helical cytokines (38)<br>8. Bromodomain-like (41)<br>9. EF Hand-like (64)<br>10. GST C-terminal domain-like (49)<br>11. SAM domain-like (33)<br>12. Alpha/alpha toroid (53)<br>13. Alpha-alpha superhelix (113)<br>14. Tetracyclin repressor-like, C-terminal domain (35)<br>15. Nuclear receptor ligand-binding domain (30)<br>16. Phospholipase A2, PLA2 (37)
	β; b	1. Immunoglobulin-like beta-sandwich (528)<br>2. Common fold of diphtheria toxin/transcription factors/cytochrome f (85)<br>3. Cupredoxin-like (77)<br>4. C2 domain-like (33)<br>5. Galactose-binding domain-like (68)<br>6. Concanavalin A-like lectins/glucanases (119)<br>7. SH3-like barrel (60)<br>8. PDZ domain-like (39)<br>9. OB-fold (122)<br>10. Beta-Trefoil (61)<br>11. Reductase/isomerase/ elongation factor common domain (39)<br>12. Split barrel-like (33)<br>13. Trypsin-like serine proteases (96)<br>14. Acid proteases (33)<br>15. PH domain-like barrel (83)<br>16. Lipocalins (65)<br>17. 6-bladed beta-propeller (33)<br>18. 7-bladed beta-propeller (35)<br>19. Single-stranded right-handed beta-helix (37)<br>20. Nucleoplasmin-like/VP (viral coat and capsid proteins) (95)<br>21. Double-stranded beta-helix (114)
	α/β; c	1. TIM beta/alpha-barrel (519)<br>2. NAD(P)-binding Rossmann-fold domains (291)<br>3. FAD/NAD(P)-binding domain (102)<br>4. The ”swivelling” beta/beta/alpha domain (35)<br>5. Leucine-rich repeat, LRR (right-handed beta-alpha superhelix) (35)<br>6. ClpP/crotonase (38)<br>7. Flavodoxin-like (173)<br>8. Adenine nucleotide alpha hydrolase-like (95)<br>9. Thiamin diphosphate-binding fold (THDP-binding) (45)<br>10. P-loop containing nucleoside triphosphate hydrolases (422)<br>11. Thioredoxin fold (108)<br>12. Anticodon-binding domain-like (31)<br>13. Restriction endonuclease-like (61)

Supplementary Table S2 – continued on next page

	Top-level hierarchical categories	Second-level hierarchical categories
SCOP	α/β; c	14. Ribonuclease H-like motif (211)<br>15. Phosphorylase/hydrolase-like (76)<br>16. PRTase-like (39)<br>17. S-adenosyl-L-methionine-dependent methyltransferases (128)<br>18. PLP-dependent transferase-like (87)<br>19. Nucleotide-diphospho-sugar transferases (42)<br>20. Alpha/beta-Hydrolases (117)<br>21. Ribokinase-like (33)<br>22. Periplasmic binding protein-like I (32)<br>23. Periplasmic binding protein-like II (95)<br>24. Thiolase-like (43)<br>25. HAD-like (61)<br>26. NagB/RpiA/CoA transferase-like (31)
	α+β; d	1. Lysozyme-like (33)<br>2. Cysteine proteinases (73)<br>3. Ribosomal protein S5 domain 2-like (53)<br>4. Beta-Grasp (ubiquitin-like) (56)<br>5. Cystatin-like (79)<br>6. UBC-like (40)<br>7. Glyoxalase/Bleomycin resistance protein/Dihydroxybiphenyl dioxygenase (45)<br>8. Thioesterase/thiol ester dehydrase-isomerase (56)<br>9. Alpha/beta-Hammerhead (32)<br>10. Ferredoxin-like (213)<br>11. Bacillus chorismate mutase-like (63)<br>12. FwdE/GAPDH domain-like (50)<br>13. Zincin-like (70)<br>14. SH2-like (38)<br>15. Acyl-CoA N-acyltransferases (Nat) (79)<br>16. Profilin-like (55)<br>17. Nudix (31)<br>18. TBP-like (71)<br>19. ATP-grasp (41)<br>20. Protein kinase-like (PK-like) (84)<br>21. Ntn hydrolase-like (63)<br>22. Metallo-hydrolase/oxidoreductase (34)<br>23. Metallo-dependent phosphatases (31)<br>24. LDH C-terminal domain-like (30)<br>25. DNA breaking-rejoining enzymes (34)<br>26. C-type lectin-like (67)<br>27. Nucleotidyltransferase (30)<br>28. Class II aaRS and biotin synthetases (44)
	multidomain; e	1. Beta-lactamase/transpeptidase-like (42)<br>2. DNA/RNA polymerases (84)

Supplementary Table S4. Details about our PSN sets belonging to the third-level hierarchical categories of CATH and SCOP. At the second-level of the CATH hierarchy, there are nine categories: 1.10, 1.20, 2.160, 2.30, 2.40, 2.60, 3.10, 3.30, and 3.40. At the second-level of the SCOP hierarchy, there are six categories: $a.118$, $b.1$, $c.1$, $c.23$, $c.26$ and $c.55$. Each second-level category has multiple third-level categories, as shown in the table. For example, the 2.60 second-level hierarchical category of CATH has two third-level categories: Jelly-rolls and Immunoglobulin-like. For each second-level hierarchical category, we specify its name and label (separated by semicolon), where the labels are as given by CATH/SCOP. For each third-level hierarchical category, we specify its name and the number of PSNs (shown in parentheses).

	**Second-level hierarchical categories**	**Third-level hierarchical categories**
**CATH**	Orthogonal Bundle; 1.10	1. Endonuclease III; domain 1 (38)<br>2. Tetracycline Repressor; domain 2 (69)<br>3. Actin-binding protein, T-fimbrin; domain 1 (46)<br>4. Recoverin; domain 1 (58)<br>5. Cytochrome Bc1 Complex; Chain D, domain 2 (47)<br>6. DNA polymerase; domain 1 (65)<br>7. Tetracycline Repressor; domain 2 (69)<br>8. Retenoid X Receptor (51)<br>9. Arc Repressor Mutant, subunit A (97)<br>10. Globin-like (123)<br>11. Cytochrome p450 (42)<br>12. Lysozyme (33)
	Up-down Bundle; 1.20	1. Glutathoine S-transferase Yfyf (Class Pi); chain A, domain 2 (76)<br>2. Butyryl-CoA Dehydrogenase, subunit A; domain 3 (45)<br>3. Fumarase C; chain A, domain 2 (30)<br>4. Methane Monooxygenase Hydroxylase; chain G, domain 1 (56)<br>5. Ferritin (61)<br>6. Four Helix Bundle (120)<br>7. Phospholipase A2 (46)<br>8. Growth hormone; chain A (42)
	3 Solenoid; 2.160	1. UDP N-Acetylglucosamine Acyltransferase; domain 1 (34)<br>2. Pectate Lyase C-like (36)
	Roll; 2.30	1. SH3 type barrels (33)<br>2. Pdz3 Domain (54)<br>3. PH-domain like (70)<br>4. Pnp Oxidase; chain A (46)
	Beta Barrel; 2.40	1. Thrombin, subunit H (123)<br>2. Porin (31)<br>3. Elongation factor Tu; domain 3 (36)<br>4. Lipocalin (102)<br>5.Cyclophilin (32)<br>6. Cathepsin D; subunit A, domain 1 (81)<br>7. OB fold (125)
	Sandwich; 2.60	1. Jelly rolls (507)<br>2. Immunoglobulin-like (932)
	Roll; 3.10	1. Mannose-binding protein A; chain A (75)<br>2. Ubiquitin Conjugating enzyme (39)<br>3. Thiol ester dehydrase; chain A (55)<br>4. Ubiquitin-like (69)<br>5. Endonuclease I-creI (42)<br>6. Nuclear transport factor 2; chain A (85)<br>7. 2-3 Dihydroxybiphenyl 1,2-Dioxygenase; domain 1 (68)
	2-Layer sandwich; 3.30	1. 60s Ribosomal protein L30; chain A (90)<br>2. Ribosomal protein S5; domain 2 (48)<br>3. GMP synthetase; chain A, domain 3 (31)<br>4. Dihydrodipicolinate Reductase; domain 2 (69)<br>5. Enolase-like; domain 1 (93)<br>6. Nucleotidytransferase; domain 5 (177)<br>7. Beta-Lactamase (76)<br>8. Beta polymerase; domain 2 (45)<br>9. D-amino acid aminotransferase; chain A, domain 1 (62)<br>10. SHC adaptor protein (52)<br>11. Alpha-D-glucose-1,6-bisphosphate; chain A, domain 1 (30)<br>12. Heat shock protein 90 (45)<br>13. Alpha-Beta plaits (239)<br>14. Enolase-like; domain 1 (53)
	2-Layer(aba) Sandwich; 3.40	1. Glutaredoxin (154)<br>2. Peroxisomal Thiolase; chain A, domain 1 (71)<br>3. Rossmann fold (412)
**SCOP**	Alph-alpha superhelix; a.118	1. ARM repeat (37)<br>2. TPR-like (32)
	Immunoglobulin-like beta-sandwich; b.1	1. Fibronectin like III (55)<br>2. E-set domains (73)<br>3. Immunoglobulin (304)
	TIM beta/alpha-barrel; c.1	1. (Trans)glycosidases (160)<br>2. Adolase (54)<br>3. Ribulose-phosphate binding barrel (36)<br>4. Metallo-dependent hydrolases (49)

Supplementary Table S3 – continued on next page

	Second-level hierarchical categories	Third-level hierarchical categories
SCOP	Flavodoxin-like; c.23	1. CheY-like (41)<br>2. Class-1 glutamine amidotransferase-like (35)<br>3. Flavoproteins (32)
	Adenine nucleotide alpha hydrolase-like; c.26	1. Nucleotidyl transferase (62)<br>2. Adenine nucleotide alpha hydrolase-like (31)
	Ribonuclease H-like motif; c.55	1. Actin-like ATPase domain (88)<br>2. Ribonuclease H-like (92)

Supplementary Table S5. Details about our PSN sets belonging to the fourth-level hierarchical categories of CATH and SCOP. At the third-level CATH hierarchy, there are six categories: 2.60.120, 2.60.40, 3.20.20, 3.30.390, 3.30.420, and 3.40.50. At the third-level SCOP hierarchy, there are four categories: $b.1.1$, $c.1.8$, $c.2.1$, and $c.37.1$. Each third-level category has multiple fourth-level categories, as shown in the table. For example, the 3.40.50 third-level hierarchical category of CATH has two fourth-level categories: Vaccinia virus protein VP39 and P-loop containing nucleotide triphosphate hydrolase. For each third-level hierarchical category, we specify its name and label (separated by semicolon), where the labels are as given by CATH/SCOP. For each fourth-level hierarchical category, we specify its name and the number of PSNs (shown in parentheses).

	**Third-level hierarchical categories**	**Fourth-level hierarchical categories**
**CATH**	Jelly rolls; 2.60.120	1. Not yet named (71)<br>2. Jelly rolls (112)<br>3. Not yet named (106)<br>4. Galactose-binding domain-like (82)
	Immunoglobulin-like; 2.60.40	1. C2-domain Calcium/lipid binding domain (36)<br>2. Cupredoxins-blue copper proteins (102)<br>3. Immunoglobulins (501)
	TIM barrel; 3.20.20	1. NADP-dependent oxidoreduxtase (39)<br>2. Aldolase class I (267)<br>3. Glycosidases (184)<br>4. Enolase superfamily (67)<br>5. Metal-dependent hydrolases (58)
	Enolase-like, domain 1; 3.30.390	1. Not yet named (30)<br>2. Enolase-like; N-terminal domain (58)
	Nucleotidyltransferase, domain 5; 3.30.420	1. Not yet named (93)<br>2. Not yet named (53)
	Rossmann fold; 3.40.50	1. Vaccinia virus protein VP39 (175)<br>2. P-loop containing nucleotide triphosphate hydrolase (115)
**SCOP**	Immunoglobulin; b.1.1	1. C1 set domains (antibody variable domain-like) (81)<br>2. V set domains (antibody variable domain-like) (200)
	(Trans)glycosidases; c.1.8	1. Beta-glycanases (53)<br>2. Amylase, catalytic domain (55)
	NAD(P)-binding Rossmann-fol domain; c.2.1	1. LDH-N-terminal domain-like (30)<br>2. Glyceraldehyde-3-phosphate dehydrogenase-like, N-terminal domain (45)<br>3. Alcohol dehydrogenase-like, C-terminal domain (30)<br>4. Tyrosine-dependent oxidoreduxtases (110)
	P-loop containing nucleoside triphosphate hydrolase; c.37.1	1. Nucleotide and nucleoside kinases (48)<br>2. Nitrogenase iron protein-like (30)<br>3. Extended AAA-ATPase domain (40)<br>4. G proteins (111)<br>5. ABC transporter ATPase domain-like (33)<br>6. Tandem AAA-ATPase domain (63)

Supplementary Table S6. Accuracy with respect to AUPR values (expressed as percentages) on synthetic networks. Results for non-normalized approaches are highlighted in 1) light gray for network data of the same size and 2) dark gray for network data of different sizes. Results for normalized approaches are not highlighted. Given a network data set (within a column), the AUPR of the best approach is shown in bold. For equivalent results with respect to AUROC values, see Supplementary Table S7.

Approach	Synthetic			
	Synthetic-100	Synthetic-500	Synthetic-1000	Synthetic-All
Graphlet-3-4	**100.00**	**100.00**	**100.00**	81.76
Graphlet-3-5	**100.00**	**100.00**	**100.00**	83.28
NormGraphlet-3-4	**100.00**	**100.00**	**100.00**	94.37
NormGraphlet-3-5	**100.00**	**100.00**	**100.00**	**99.86**
GDDA	97.36	**100.00**	99.99	91.46
RGFD	**100.00**	**100.00**	**100.00**	98.55
GCD	89.26	**100.00**	**100.00**	86.27
Average degree	79.76	79.76	79.76	68.77
Average distance	82.47	98.12	99.60	57.10
Maximum distance	68.82	84.32	93.08	46.11
Average closeness centrality	86.10	88.46	85.33	48.41
Average clustering coefficient	98.93	99.68	99.25	79.37
Intra-hub connectivity	70.88	69.11	69.31	66.61
Assortativity	82.79	92.27	91.73	81.98
Existing-all	**100.00**	**100.00**	**100.00**	85.92

Supplementary Table S7. Accuracy with respect to AUROC values (expressed as percentages) on synthetic networks. Results for non-normalized approaches are highlighted in 1) light gray for network data of the same size and 2) dark gray for network data of different sizes. Results for normalized approaches are not highlighted. Given a network data set (within a column), the AUROC of the best approach is shown in bold. For equivalent results with respect to AUPR values, see Supplementary Table S6.

Approach	Synthetic			
	Synthetic-100	Synthetic-500	Synthetic-1000	Synthetic-All
Graphlet-3-4	**100.00**	**100.00**	**100.00**	82.58
Graphlet-3-5	**100.00**	**100.00**	**100.00**	86.43
NormGraphlet-3-4	**100.00**	**100.00**	**100.00**	97.39
NormGraphlet-3-5	**100.00**	**100.00**	**100.00**	**99.93**
GDDA	98.53	**100.00**	**100.00**	91.73
RGFD	**100.00**	**100.00**	**100.00**	99.21
GCD	89.88	**100.00**	**100.00**	87.89
Average degree	83.33	83.33	83.33	72.14
Average distance	90.28	98.61	99.70	69.66
Maximum distance	79.89	90.63	95.04	54.88
Average closeness centrality	87.80	84.24	80.89	54.84
Average clustering coefficient	99.39	99.81	99.48	88.91
Intra-hub connectivity	79.02	78.22	78.31	71.19
Assortativity	92.75	95.36	95.37	91.61
Existing-all	**100.00**	**100.00**	**100.00**	92.50

Supplementary Table S8. Accuracy with respect to AUPR values (expressed as percentages) on the three real-world PSN sets that form the "equal size" group, each of which contains networks of the same size. Also, average accuracy over all three PSN sets is shown ("Average"), along with the corresponding standard deviation ("SD"). Results for non-normalized approaches are highlighted in light gray. Results for normalized approaches are not highlighted. Given a PSN set (within a given column), the AUPR of the best approach is shown in bold. For equivalent results with respect to AUROC values, see Supplementary Table S9.

Approach	"Equal size" PSN sets			
	CATH-95	CATH-99	CATH-251-265	Average (SD)
Graphlet-3-4	93.31	92.05	98.77	94.71 (3.57)
Graphlet-3-5	89.67	92.78	**100.00**	94.15 (5.29)
NormGraphlet-3-4	96.03	**100.00**	95.28	97.1 (2.54)
NormGraphlet-3-5	94.11	99.73	97.67	97.17 (2.84)
OrderedGraphlet-3	90.99	95.93	91.02	92.65 (2.84)
OrderedGraphlet-3-4	96.69	91.56	97.20	95.15 (3.12)
NormOrderedGraphlet-3	91.53	98.9	93.51	94.65 (3.81)
NormOrderedGraphlet-3-4	**97.59**	96.63	98.74	**97.65 (1.06)**
NormOrderedGraphlet-3-4(K)	**97.59**	96.63	98.74	**97.65 (1.06)**
GDDA	80.21	80.78	71.46	77.48 (5.22)
RGFD	87.87	89.49	94.00	90.45 (3.18)
GCD	75.89	74.92	77.23	76.01 (1.16)
GR-Align	76.25	65.03	70.25	70.51 (5.61)
Average degree	80.47	86.91	85.57	84.32 (3.40)
Average distance	72.90	86.54	51.60	70.35 (17.60)
Maximum distance	62.86	73.49	54.89	63.75 (9.33)
Average closeness centrality	73.12	85.88	49.37	69.46 (18.53)
Average clustering coefficient	87.01	81.21	89.96	86.06 (4.45)
Intra-hub connectivity	70.24	84.24	63.76	72.75 (10.47)
Assortativity	79.94	85.34	93.31	86.20 (6.73)
Existing-all	84.66	96.32	92.48	91.15 (5.94)
DaliLite	53.38	69.12	58.96	60.49 (7.98)
TM-align	50.93	62.02	45.79	52.91 (8.29)
AAComposition	70.23	62.14	54.48	62.28 (7.88)

Supplementary Table S9. Accuracy with respect to AUROC values (expressed as percentages) on the three real-world PSN sets that form the "equal size" group, each of which contains networks of the same size. Also, average accuracy over all three PSN sets is shown ("Average"), along with the corresponding standard deviation ("SD"). Results for non-normalized approaches are highlighted in light gray. Results for normalized approaches are not highlighted. Given a PSN set (within a given column), the AUROC of the best approach is shown in bold. For equivalent results with respect to AUPR values, see Supplementary Table S8.

Approach	CATH of the same size			
	CATH-95	CATH-99	CATH-251-265	Average (SD)
Graphlet-3-4	93.629	92.55	98.80	94.99 (3.34)
Graphlet-3-5	91.97	92.65	**100.00**	94.87 (4.45)
NormGraphlet-3-4	96.48	**100.00**	94.35	96.94 (2.85)
NormGraphlet-3-5	94.114	99.73	97.83	97.22 (2.86)
OrderedGraphlet-3	91.49	96	91.97	93.15 (2.48)
OrderedGraphlet-3-4	96.69	97.15	97.05	96.96 (0.24)
NormOrderedGraphlet-3	89.69	99.04	93.62	94.11 (4.69)
NormOrderedGraphlet-3-4	**97.51**	97.253	98.72	**97.83 (0.78)**
NormOrderedGraphlet-3-4(K)	**97.51**	97.253	98.72	**97.83 (0.78)**
GDDA	80.62	79.33	68.98	76.31 (6.38)
RGFD	85.65	88.45	93.43	89.18 (3.94)
GCD	73.9	73.88	78.67	75.48 (2.76)
GR-Align	71.14	60.49	66.03	65.89 (5.33)
Average degree	85.36	88.99	84.71	86.35 (2.31)
Average distance	73.45	83.79	55.33	70.86 (14.41)
Maximum distance	60.39	71.80	59.45	63.88 (6.88)
Average closeness centrality	74.93	82.73	53.69	70.45 (15.03)
Average clustering coefficient	86.98	85.15	88.30	86.81 (1.58)
Intra-hub connectivity	73.98	86.52	64.88	75.13 (10.87)
Assortativity	85.48	90.19	94.79	90.15 (4.66)
Existing-all	85.55	96.41	92.73	91.56 (5.52)
DaliLite	62.74	71.62	62.13	65.16 (5.65)
TM-align	50.73	65.03	47.84	54.53 (9.20)
AAComposition	69.38	64.12	58.42	63.97 (5.48)

Supplementary Table S10. Summary of method accuracy and running times. Accuracy of the given approach is shown with respect to its average ranking as well as its average raw score compared to all considered approaches across all 35 different-size PSN sets, and the results are shown based on AUPR as well as AUROC. We rank the approaches as follows. For the given PSN set, we determine which approach results in the highest accuracy (rank 1), the second highest accuracy (rank 2), etc. Then, we average the rankings of the given method over all PSN sets. So, the lower the average rank, the better the method. Since NormOrderedGraphlet-3-4(K) has the best average rank with respect to both AUPR and AUROC (shown in bold), we compute the statistical significance of the improvement of NormOrderedGraphlet-3-4(K) over each of the other approaches in terms of their ranks using paired t-test. We also do the same in terms of raw AUPR/AUROC values. Note that in the case of raw values, the higher the average AUPR/AUROC value, the better the approach. Running times of the approaches are shown when comparing proteins from the CATH-α set. Running times for the other data sets are qualitatively the same.

	Rank-based				Raw score-based				
Approach	AUPR		AUROC		AUPR		AUROC		Running
	Avg rank	*p*-value	Avg rank	*p*-value	Avg score	*p*-value	Avg score	*p*-value	time (hrs)
Graphlet-3-4	9.91	2.94e-11	12.80	3.81e-15	51.18	5.92e-10	64.84	1.42e-11	0.43
Graphlet-3-5	12.34	1.61e-14	12.94	3.46e-15	49.23	9.53e-11	64.69	1.70e-11	0.49
NormGraphlet-3-4	10.89	2.25e-18	10.14	5.83e-15	50.73	9.50e-11	67.12	1.11e-11	0.44
NormGraphlet-3-5	10.25	5.87e-16	9.26	4.57e-14	51.24	1.67e-10	67.56	2.57e-11	0.51
OrderedGraphlet-3	11.03	1.92e-13	13.09	2.61e-14	51.30	1.78e-10	65.52	9.41e-12	0.38
OrderedGraphlet-3-4	7.91	1.49e-14	8.91	8.79e-10	54.11	2.36e-11	68.28	2.75e-11	2.39
NormOrderedGraphlet-3	10.77	3.49e-13	10.48	7.33e-11	51.34	1.23e-11	68.37	5.89e-11	0.39
NormOrderedGraphlet-3-4	4.31	5.23e-07	5.14	1.28e-06	62.79	6.95e-08	74.58	1.93e-06	2.41
NormOrderedGraphlet-3-4(K)	**1.83**	-	**1.97**	-	**69.88**	-	**80.42**	-	2.41
GDDA	16.17	1.66e-15	17.37	1.67e-15	44.68	1.76e-11	58.51	8.94e-13	0.54
RGFD	11.29	1.55e-13	11.60	2.01e-12	50.02	4.41e-10	66.01	1.59e-10	0.49
GCD	15.71	4.21e-16	15.43	2.93e-13	45.67	1.55e-11	61.68	7.47e-13	1.32
GR-Align	4.43	2.10e-03	6.68	8.91e-06	64.40	4.60e-04	73.02	5.42e-06	9.49
Average degree	18.85	3.32e-20	16.00	8.27e-16	42.64	1.58e-12	61.26	8.93e-13	0.39
Average distance	17.66	4.04e-19	16.91	1.19e-16	43.63	1.14e-12	61.08	6.27e-13	0.48
Maximum distance	16.03	3.18e-17	14.83	1.14e-14	46.04	3.08e-11	63.36	5.65e-11	0.49
Average closeness centrality	16.31	9.70e-18	15.51	2.31e-14	45.24	1.69e-11	62.49	5.19e-10	0.48
Average clustering coefficient	18.6	6.65e-22	15.54	4.83e-16	43.11	2.08e-12	62.02	3.29e-12	0.56
Intra-hub connectivity	14.37	8.99e-12	15.80	2.33e-16	47.01	1.88e-09	62.32	3.41e-10	0.64
Assortativity	21.00	2.29e-24	19.00	3.53e-17	40.22	2.46e-13	57.88	1.33e-14	0.46
Existing-all	10.14	6.49e-15	9.57	3.28e-11	51.10	1.49e-09	67.54	2.40e-10	1.01
DaliLite	9.14	1.84e-06	6.29	6.21e-04	54.36	7.78e-07	73.73	2.44e-03	2021.41
TM-align	18.23	5.38e-15	20.09	3.05e-19	43.72	3.26e-12	57.18	7.08e-15	168.32
AAComposition	12.80	6.40e-12	14.63	1.16e-13	48.58	1.62e-11	63.31	2.62e-12	0.24

Supplementary Table S11. Detailed accuracy results for each PC approach, each PSN set, and each PSN construction strategy, with respect to AUPR values.

http://nd.edu/~cone/PSN/ST11.xlsx

Supplementary Table S12. Detailed accuracy results for each PC approach, each PSN set, and each PSN construction strategy, with respect to AUROC values.

http://nd.edu/~cone/PSN/ST12.xlsx

Supplementary Table S13. Accuracy of the NormOrderedGraphlet-3-4(K) approach when varying the value of K, with respect to **AUPR values** (expressed as percentages), corresponding to the PSN sets of **“equal size”, group 1, and group 2**. Given a PSN set (within a given column), the AUPR for the “best” K is shown in bold. These results are with respect to the first PSN construction strategy, **(any heavy atom type, 4 Å distance cut-off)**.

K value	“Equal size” PSN sets			CATH group 1 and group 2 PSN sets				SCOP group 1 and group 2 PSN sets					
	CATH-95	CATH-99	CATH-251-265	primary	α	β	α/β	primary	α	β	α/β	$\alpha+\beta$	Multi domain
1	**97.5907**	**96.5535**	**98.7353**	**65.6432**	53.0727	**49.1096**	44.3478	**44.4874**	21.7461	26.1047	16.6605	19.9294	72.7278
2	95.2756	87.5503	95.4586	62.7671	53.0854	44.8639	42.8751	40.3947	23.3953	22.0232	16.4849	20.4017	71.813
3	88.8915	93.2875	95.4625	63.0474	**53.4148**	42.8472	40.944	40.1442	25.3495	24.3654	17.0831	23.8401	70.5777
4	82.6216	89.9151	87.9388	57.7995	53.387	41.8727	41.9876	38.2845	**31.6257**	26.0871	21.4571	27.443	72.0587
5	81.9265	80.9028	70.0715	46.0584	51.2881	41.9493	42.2158	34.8211	22.9261	28.5727	27.1045	32.5745	80.15
6	86.2847	84.4491	74.8161	45.958	50.8029	42.082	42.3182	34.6656	22.7179	28.6674	28.3451	33.8087	81.1718
7	86.0194	87.1074	75.9893	46.0629	50.4549	41.9585	42.4536	34.3967	22.0997	28.9025	29.3658	**33.8359**	80.7989
8	86.7051	87.0867	79.6482	46.2146	50.3169	41.8143	42.6578	34.2873	21.7503	28.7242	30.1709	33.5889	81.3369
9	85.8064	90.7948	77.2424	46.367	50.2707	41.5483	42.6963	34.2836	21.601	28.2805	30.8011	32.3956	**82.0152**
10	87.2977	91.0244	79.729	46.1782	50.1931	41.2059	43.2325	34.4044	21.1446	28.1126	31.5902	31.9091	80.5146
15	90.0798	88.9304	84.481	44.7598	50.0598	39.8044	46.1777	35.4319	17.9958	22.9517	**34.3508**	25.923	76.7302
20	85.0209	77.1504	84.173	44.0788	49.8056	40.6313	**48.3086**	34.3561	16.0554	24.9723	34.1548	23.0386	70.9673
25	76.7759	68.7322	70.2256	42.1777	49.8105	40.1944	45.1245	33.9106	16.061	24.3791	26.2348	22.3209	69.4278
30	68.4945	72.9198	66.7278	40.1754	49.9376	39.9909	40.3299	15.866	14.8808	27.3717	18.2031	19.2868	71.8487
35	72.8877	72.551	72.6056	39.1643	49.7902	42.4063	37.5206	28.5898	14.2925	**38.8313**	15.0003	16.8235	74.4817

Supplementary Table S14. Accuracy of the NormOrderedGraphlet-3-4(K) approach when varying the value of K, with respect to **AUROC values** (expressed as percentages), corresponding to the PSN sets of **“equal size”, group 1, and group 2**. Given a network data set (within a given column), the AUROC for the “best” K is shown in bold. These results are with respect to the first PSN construction strategy, **(any heavy atom type, 4 Å distance cut-off)**.

K value	“Equal size” PSN sets			CATH group 1 and group 2 PSN sets				SCOP group 1 and group 2 PSN sets					
	CATH-95	CATH-99	CATH-251-265	primary	α	β	α/β	primary	α	β	α/β	$\alpha+\beta$	Multi domain
1	**97.5063**	**96.553**	**98.719**	**76.7895**	53.6152	**65.5625**	62.2712	**70.1427**	70.1844	72.2486	66.175	75.8923	65.9186
2	95.4861	88.5529	95.5723	74.1997	53.7436	62.6332	60.8591	66.5967	71.1121	68.0998	66.2109	75.8605	63.8069
3	90.5303	92.8343	95.1267	74.5916	**54.6141**	59.4459	59.8402	66.38	72.2224	68.1506	67.6919	78.4727	62.4508
4	83.4701	89.7681	88.1649	71.0703	54.5974	58.17	60.3451	64.7223	**76.5624**	69.6688	73.1774	81.2198	63.4953
5	83.0387	82.7901	71.2058	61.5761	53.1395	58.0683	60.4218	61.3279	68.8734	71.7692	76.8098	83.4135	74.4544
6	86.8161	85.3355	74.826	61.4999	52.536	58.0416	60.5189	61.1776	68.6371	71.2808	77.3252	**83.7493**	75.8451
7	86.9581	88.1664	77.0259	61.6935	52.1318	57.8011	60.5151	60.9443	68.9523	71.297	77.7241	83.3214	75.4427
8	88.3733	87.7408	80.1448	61.8951	51.8638	57.6244	60.628	60.855	69.289	71.0992	78.1114	83.1375	76.1182
9	87.2106	90.4766	78.279	62.0832	51.7987	57.1752	60.5787	60.8541	69.6458	70.6048	78.3253	82.5695	**77.1106**
10	87.7736	90.3646	80.0334	61.8661	51.6322	56.5845	60.9359	60.9248	69.2482	70.0239	78.4249	81.8158	75.4035
15	91.0511	89.4741	84.9624	60.1361	51.3839	55.6159	63.1589	61.6798	67.3932	65.9048	**78.7761**	78.5889	71.3872
20	86.7582	77.3269	84.7396	59.1382	51.6232	56.1634	**65.3814**	64.4742	65.3899	66.6936	77.7261	75.6472	65.1717
25	79.1193	71.5082	72.8209	57.8993	51.5256	55.7813	63.7101	60.598	65.6534	65.4956	70.7485	74.8885	62.0886
30	72.6904	77.4502	64.7173	56.4285	51.6013	55.3446	60.4836	62.7463	65.5548	67.2713	64.6058	73.3497	65.6116
35	73.1534	73.1043	71.8184	55.4317	51.5061	55.909	58.1067	55.1192	65.2114	**77.5071**	62.6461	72.2011	68.721

Supplementary Table S15. Accuracy of the NormOrderedGraphlet-3-4(K) approach when varying the value of K, with respect to **AUPR values** (expressed as percentages), corresponding to the PSN sets of **group 3**. Given a PSN data set (within a given column), the AUPR for the "best" K is shown in bold. These results are with respect to the first PSN construction strategy, **(any heavy atom type, 4 Å distance cut-off)**.

K	CATH (group 3 PSN sets)									SCOP (group 3 PSN sets)					
	1.10	1.20	2.30	2.40	2.60	2.160	3.10	3.30	3.40	a.118	b.1	c.1	c.23	c.26	c.55
1	**50.99**	**41.35**	58.62	40.17	71.12	**91.56**	52.93	27.66	59.63	58.46	60.04	49.85	57.08	61.19	62.31
2	35.05	37.2	64.79	41.58	74.07	84.01	57.58	31.62	61.55	56.7	64.89	51.33	67.48	60.91	58.68
3	27.35	37.89	65.91	42.75	75.63	79.76	62.43	38.1	64.01	60.07	65.89	**51.44**	**73.34**	**62.75**	62.34
4	38.47	30.35	**66.23**	44.71	76.78	76.34	68.88	40.81	65.09	**65.77**	65.71	50.17	67.4	60.27	62.85
5	40.8	31.12	65.48	**45.72**	77.39	70.73	**69.7**	**40.91**	64.98	65.07	66.18	50.16	66.14	59.48	61.69
6	41.93	30.24	62.98	44.91	77.99	66.82	68.83	40.62	65.89	63.18	**66.4**	49.5	65.21	59.03	61.4
7	40.04	29.39	59.24	44.4	78.18	70.78	68.35	39.47	**66.66**	62.92	65.89	46.63	61.73	58.79	60.96
8	37.51	30.3	58.93	44.1	**78.33**	68.85	68.23	37.53	66.32	59.62	65.72	44.08	59.87	58.42	62.52
9	34.05	30.24	57.98	44.38	78.02	65.16	66.61	36.1	66.44	56.96	64.94	42.1	59.28	58.03	61.8
10	29.52	31.7	56.51	44.63	77.43	62.35	62.95	34.38	66.59	57.34	64.14	41.3	58.32	57.44	60.78
15	22.22	31.15	53.1	44.9	71.16	54.62	56.1	29.04	61.71	53.36	61.2	41.15	58.59	58.9	60.72
20	23.06	29.35	48.68	40.43	66.35	51.25	50.09	25.26	57.12	51.49	61.89	38.56	62.71	61.74	60.15
25	22.47	26.54	42.1	35.65	64.11	52.45	46.02	21.85	57	54.03	63.3	39.28	52.64	56.55	61.35
30	19.44	23.87	39.05	32.9	66.24	54.06	34.24	20.45	54.44	56.16	**66.4**	40.55	49.48	56.51	**63.5**
35	15.74	19.95	35.44	29.93	67.17	54.74	31.9	19.15	51.76	57.58	64.73	40.84	42.95	56.25	62.57

Supplementary Table S16. Accuracy of the NormOrderedGraphlet-3-4(K) approach when varying the value of K, with respect to **AUROC values** (expressed as percentages), corresponding to the PSN sets of **group 3**. Given a third-level PSN data set (within a given column), the AUROC for the "best" K is shown in bold. These results are with respect to the first PSN construction strategy, **(any heavy atom type, 4 Å distance cut-off)**.

K	CATH (group 3 PSN sets)									SCOP (group 3 PSN sets)					
	1.10	1.20	2.30	2.40	2.60	2.160	3.10	3.30	3.40	a.118	b.1	c.1	c.23	c.26	c.55
1	**84.65**	**74.22**	78.47	71.31	68.18	**91.08**	82.71	69.7	57.45	56.17	52.71	**63.6**	73.17	56.41	60.76
2	79.68	72.1	81.62	73.9	70.59	83.41	85.13	70.93	61.02	57.31	57.8	62.06	80.49	56.86	57.45
3	75.94	68.28	81.78	75.04	72.44	78.73	86.3	76.07	62.98	60.62	58.27	62.47	**81.18**	**56.92**	62.08
4	76.93	66.28	**82.27**	75.42	73.83	74.34	89.76	**77.1**	63.71	**64.64**	57.44	61.32	77.85	53.95	**63.03**
5	77.59	66.51	81.58	75.33	74.59	68.13	**90.49**	76.98	63.61	64.52	57.56	61.12	76.22	53.49	61.58
6	77.49	66.25	80.05	74.13	75.37	65.27	90.15	76.8	64.67	63.31	57.95	60.71	75.65	52.64	61.49
7	76.82	65.98	77.92	73.47	75.83	69.93	89.46	76.14	**65.66**	63.84	57.66	58.42	73.64	52.26	60.33
8	75.91	66.63	77.57	73.75	**76.19**	68.98	89.17	75.13	65.37	61.1	57.98	56	72.68	52.46	61.94
9	73.97	66.84	76.32	74.6	76.09	68.41	88.48	74.12	65.57	58.37	57.31	54.81	72.05	51.42	61.38
10	71.49	67.94	75.25	75.27	75.69	65.74	86.18	73.25	**65.66**	59.23	56.77	54.55	71.25	51.5	59.57
15	65.08	67.81	71.79	**75.76**	68.96	56.67	79.27	68.31	60.34	51.78	54.49	54.69	70.7	52.21	59.07
20	65.82	66.19	69.48	71.67	63.2	50.37	76.41	65.73	57.98	49.98	54.63	53.35	76.56	56.49	58.28
25	64.34	64.89	64.2	68.66	60.25	51.26	76.89	65.09	57.43	51.28	55.79	54.65	68.61	51.26	59.44
30	62.2	62.37	62.19	65.85	59.93	52.17	73.04	64.64	54.24	50.01	**59.64**	56.52	66.16	51.18	62.86
35	58.13	59.4	61.06	63.82	62.23	50.7	68.56	64.13	52.1	50.17	59.05	56.28	60.16	50.37	61.64

Supplementary Table S17. Accuracy of the NormOrderedGraphlet-3-4(K) approach when varying the value of K, with respect to **AUPR values** (expressed as percentages), corresponding to the PSN sets of **group 4**. Given a fourth-level PSN data set (within a given column), the AUPR for the "best" K is shown in bold. These results are with respect to the first PSN construction strategy, **(any heavy atom type, 4 Å distance cut-off)**.

K	CATH (group 4 PSN sets)						SCOP (group 4 PSN sets)			
	2.60.40	2.60.120	3.20.20	3.40.50	3.30.390	3.30.420	b.1.1	c.1.8	c.2.1	c.37.1
1	71.8	49.12	**42.28**	63.34	92.15	**70.14**	86.72	73.37	69.82	49.62
2	73.75	55.9	40.78	71.73	89.58	65.45	88.81	76.52	69.74	50.63
3	75.59	**56.88**	41.9	**79.35**	88.04	66.36	89.66	77.1	60.34	55.94
4	76.23	54.38	41.33	79.15	**92.98**	67.43	90.78	78.46	63.79	58.78
5	76.6	52.69	41.17	78.59	92.62	67.97	**91.78**	78.73	65.71	58.88
6	76.6	50.13	40.39	78.35	92.22	67.5	91.27	**79.65**	65.94	59
7	76.29	46.44	39.2	78.75	92.83	66.33	90.26	78.74	67.44	59.33
8	76.03	44.98	38.27	79.07	92.24	66.82	89.54	76.41	67.76	59.63
9	75.31	43.75	37.17	78.73	91.82	67.66	89.24	74.95	67.85	59.55
10	75.06	42.49	36.24	78.29	91.29	68.03	88.89	70.83	70.32	**60.12**
15	74.53	43.46	37.55	64.64	81.79	67.31	85.56	60.86	**71.76**	49.22
20	75.29	42.67	38.79	63.46	75.95	66.65	90.07	64.84	49.53	38.32
25	75.82	39.28	35.46	66.8	77.06	65.46	90.02	57.76	51.34	39.6
30	**79.24**	36.74	34.62	67.95	75.2	67.92	86.59	55.15	48.1	34.29
35	77.89	35.25	34.68	65.96	71.38	69.71	83.82	53.96	51.98	33.84

Supplementary Table S18. Accuracy of the NormOrderedGraphlet-3-4(K) approach when varying the value of K, with respect to **AUROC values** (expressed as percentages), corresponding to the PSN sets of **group 4**. Given a fourth-level PSN data set (within a given column), the AUROC for the "best" K is shown in bold. These results are with respect to the first PSN construction strategy, **(any heavy atom type, 4 Å distance cut-off)**.

K	CATH (group 4 PSN sets)						SCOP (group 4 PSN sets)			
	2.60.40	2.60.120	3.20.20	3.40.50	3.30.390	3.30.420	b.1.1	c.1.8	c.2.1	c.37.1
1	56.22	69.1	**60.9**	60.32	**91.24**	63.56	81.2	74.02	**85.25**	74.15
2	58.43	74.57	58.91	69.76	87.68	58.3	83.65	77.54	84.44	78.11
3	60.44	**75.64**	60.19	**77.91**	85.6	61.28	85.3	78.23	70.08	82.02
4	61.11	74.68	59.82	77.31	90.91	61.73	86.9	79.16	73.6	83.89
5	61.21	73.85	59.91	76.79	90.6	62.77	**88.24**	79.24	75.88	84.41
6	61.27	71.28	59.42	76.43	90.15	62.08	87.55	**80.29**	76.59	84.74
7	61.04	68.3	57.9	76.72	90.88	60.39	86.24	79.16	77.94	**84.76**
8	60.91	66.56	56.48	76.88	90.04	61.09	85.32	77.24	78.32	84.38
9	60.41	65.49	55.35	76.34	89.52	61.42	84.8	75.76	78.95	84.35
10	59.98	64.33	55	75.8	89.12	61.31	84.4	72.12	81.54	84.26
15	60.32	67.26	55.69	59.99	78.78	61.99	79.05	61.86	82.25	80.18
20	62.27	67.43	56.95	60.46	72.9	59.98	85.77	66.43	67.75	74.19
25	62.93	63.67	55.18	65.83	73.8	59.4	85.98	57.09	67.35	75.21
30	**67.46**	61.8	54.37	67.42	71.21	60.08	81.09	56.44	64.78	70.42
35	65.87	60.3	54.52	64.82	67.53	**63.98**	79.21	54.15	66.54	69.83

Supplementary Table S19. Accuracy of the NormOrderedGraphlet-3-4(K) approach when varying the value of K, with respect to **AUPR values** (expressed as percentages), corresponding to the PSN sets of **"equal size", group 1, and group 2**. Given a PSN data set (within a given column), the AUPR for the "best" K is shown in bold. These results are with respect to the second PSN construction strategy, **(any heavy atom type, 5 Å distance cut-off)**.

K	"Equal size" PSN sets			CATH group 1 and group 2 PSN sets				SCOP group 1 and group 2 PSN sets					
	CATH-95	CATH-99	CATH-251-265	primary	α	β	α/β	primary	α	β	α/β	$\alpha+\beta$	Multi domain
1	**90.1293**	**96.6286**	**92.7763**	**52.9226**	**52.5874**	**47.0492**	**47.3051**	**37.232**	28.4611	42.4837	20.2765	31.0348	78.3265
2	89.6195	95.2273	67.193	49.1635	50.8747	45.6473	47.1739	35.6826	25.6525	42.2464	24.3543	37.9497	87.8922
3	85.8699	95.7829	67.0638	44.8789	51.1422	45.7451	43.3687	34.6397	23.8388	46.5653	29.4657	43.3618	**89.0528**
4	76.2696	87.5723	67.1862	46.0384	51.0328	46.5143	41.3818	34.5581	28.6271	48.9183	32.9691	47.6121	88.0104
5	72.2232	83.5286	69.4609	48.3949	50.9314	46.7874	41.5733	34.9807	**31.3888**	50.1176	35.6024	**48.2585**	84.8499
6	74.4759	84.4109	71.2008	47.9608	50.8817	46.6286	41.7965	34.3337	29.0074	50.9931	**37.0611**	46.6793	79.7143
7	77.6668	87.7908	70.0946	47.2362	50.7636	46.5299	41.6575	33.7386	27.8268	**51.0821**	36.84	44.8139	77.4482
8	80.7965	87.522	66.4301	46.3593	50.8364	46.4849	40.9544	33.1075	26.8478	50.5899	33.1191	42.4918	76.7347
9	80.3752	89.2913	61.5194	45.3429	50.8007	45.9257	39.4201	32.4303	26.1365	49.1723	28.3289	40.6064	75.269
10	80.7622	88.6804	59.036	44.3988	50.6798	45.314	37.8329	31.8444	25.5496	46.2298	24.9365	38.162	76.1149
15	82.8087	84.3218	57.2975	41.0722	50.7824	43.2646	36.7109	29.6248	22.0483	35.3323	19.007	31.0906	76.0938
20	79.4233	76.2962	57.8944	39.4257	50.6986	42.2841	36.4621	28.2187	19.4718	30.3763	15.4103	25.0401	76.958
25	76.287	68.7135	61.783	38.5145	50.2616	41.3778	35.1202	27.7108	19.4538	24.1232	15.4994	21.1172	69.6598
30	71.5104	68.2464	50.3365	38.2127	50.423	41.8735	33.9592	27.0019	20.959	26.7283	14.6463	16.7388	67.7744
35	65.2411	64.5557	52.5491	37.9295	49.8745	41.8544	33.0769	26.7711	20.1306	27.932	13.5486	14.7098	67.0186

Supplementary Table S20. Accuracy of the NormOrderedGraphlet-3-4(K) approach when varying the value of K, with respect to **AUROC values** (expressed as percentages), corresponding to the PSN sets of **"equal size", group 1, and group 2**. Given a PSN data set (within a given column), the AUROC for the "best" K is shown in bold. These results are with respect to the second PSN construction strategy, **(any heavy atom type, 5 Å distance cut-off)**.

K	"Equal size" PSN sets			CATH group 1 and group 2 PSN sets				SCOP group 1 and group 2 PSN sets					
	CATH-95	CATH-99	CATH-251-265	primary	α	β	α/β	primary	α	β	α/β	$\alpha+\beta$	Multi domain
1	**92.1559**	**97.253**	**92.8154**	**64.9132**	**54.0484**	**61.6982**	**66.3703**	**61.7145**	71.9486	82.0656	68.6802	81.468	72.6387
2	90.4619	95.9313	66.0262	61.413	52.6914	59.0553	65.0137	60.803	71.6996	80.6121	73.6204	84.2634	83.5883
3	86.0848	95.1865	65.9705	58.3947	52.051	58.2127	61.2662	60.6236	72.2835	82.9934	77.5	86.2332	**85.8352**
4	76.8992	88.7881	66.8059	60.563	52.2507	58.1884	59.4255	60.1748	**74.5909**	84.2071	78.9938	**87.3249**	84.9401
5	70.4335	85.4475	67.1122	62.9896	51.8748	57.8519	59.0915	60.6441	74.1982	84.3766	**79.6859**	87.0462	80.7901
6	73.6637	85.0722	67.2793	62.5252	51.7947	57.1182	58.854	59.9899	73.3497	84.4453	79.4441	86.5254	74.3131
7	78.5669	87.9648	66.8059	61.89	51.6872	56.1646	58.4529	59.3303	72.472	**84.4774**	78.3338	85.926	72.5964
8	83.528	87.6624	65.7199	61.247	51.7367	55.3276	57.9244	58.6571	72.3752	84.2605	75.9383	85.1385	72.2244
9	83.5701	88.8721	63.4642	60.4646	51.8759	54.7189	57.036	57.9642	71.9669	83.8162	72.7459	84.089	70.2003
10	84.1698	88.4017	60.6238	59.6521	51.9814	54.4152	56.0152	57.2797	71.5617	82.5829	69.8894	83.0109	71.2147
15	85.0011	85.2039	59.9554	56.6982	52.2242	55.4201	55.739	54.8945	69.9921	76.1676	65.1307	79.0645	70.0531
20	81.5341	77.0021	57.8669	55.1886	51.8846	55.5238	56.2769	53.9974	67.2989	71.521	63.5112	76.579	72.6873
25	77.8304	71.0181	59.1757	54.2624	51.0143	55.2558	55.1755	53.2939	67.5178	65.9935	63.4282	73.9711	64.4481
30	74.2109	66.8375	54.3581	54.0428	50.5563	55.41	54.0227	52.5326	67.8453	65.0736	62.1002	71.8817	63.0047
35	65.2252	64.4237	49.6798	54.0465	50.2559	56.0247	52.9777	52.4741	66.458	69.3304	60.1751	69.6485	58.0457

Supplementary Table S21. Accuracy of the NormOrderedGraphlet-3-4(K) approach when varying the value of K, with respect to **AUPR values** (expressed as percentages), corresponding to the PSN sets of **group 3**. Given a third-level PSN data set (within a given column), the AUPR for the "best" K is shown in bold. These results are with respect to the second PSN construction strategy, **(any heavy atom type, 5 Å distance cut-off)**.

K	CATH (group 3 PSN sets)									SCOP (group 3 PSN sets)					
	1.10	1.20	2.30	2.40	2.60	2.160	3.10	3.30	3.40	a.118	b.1	c.1	c.23	c.26	c.55
1	**58.68**	**50.59**	77.73	51.3	80.41	89.61	68.31	41.22	63.45	58.07	66.84	52.04	63.32	61.64	62.73
2	48.04	50.34	81.79	53.86	80.81	**91.46**	69.82	44.16	64.01	58.8	72.39	53.33	70.02	60.65	60.46
3	38.43	49.89	82.77	56.23	82.73	85.89	78.27	49.41	65.96	60.65	73.86	**54.2**	**75.33**	61.56	62.93
4	49.76	43.71	**83.71**	57.82	84.04	82	**83.51**	**52.52**	67.21	68.01	72.94	52.85	74.85	61.38	65.37
5	56.56	48.66	81.96	59.17	84.64	77.73	83.41	52.29	68.54	**68.63**	73.68	52.91	74.46	60.54	65.36
6	56.23	48.37	78.82	58.56	85.06	74.99	82.04	51.43	69.65	67.76	74.05	52.3	73.78	60.04	65.8
7	53.74	46.66	76.45	58.32	**85.34**	76.89	81.26	50.22	70.4	66.23	74.01	49.41	73.66	59.39	65.6
8	50.35	47.26	75.8	58.61	85.21	74.9	80.37	48.52	70.66	61.34	**74.27**	46.99	70.7	59.04	65.31
9	46.73	47.92	76.13	60.05	85.01	71.37	79.02	46.22	70.99	58.34	73.16	44.97	68.18	58.5	64.54
10	43.99	48.95	75.48	**60.21**	84.34	65.74	76.42	45.05	**71.09**	58.5	71.7	44.03	66.5	57.91	62.96
15	37.69	46.37	71.03	56.47	79.03	57.78	68.86	39.15	65.52	52.61	69.85	45.95	66.67	59.94	60.99
20	39.07	42.69	62.88	51.37	74.48	51.06	63.12	34.96	62.05	52.04	68.98	42.05	74.69	**62.06**	62.34
25	38.39	40.19	50.39	48.01	70.34	52.65	56.39	28.61	62.42	56.2	69.75	41.75	62.26	58.42	62.36
30	31.75	35.22	47.89	41.61	68.66	54.02	44.65	25.31	59.75	56.4	69.19	42.91	57.82	57.88	66.68
35	22.27	28.09	45.08	37.72	71.95	54.75	39.71	23.56	54.98	57.34	67.94	45.05	47.72	58.08	**67.53**

Supplementary Table S22. Accuracy of the NormOrderedGraphlet-3-4(K) approach when varying the value of K, with respect to **AUROC values** (expressed as percentages), corresponding to the PSN sets of **group 3**. Given a third-level PSN data set (within a given column), the AUROC for the "best" K is shown in bold. These results are with respect to the second PSN construction strategy, **(any heavy atom type, 5 Å distance cut-off)**.

K	CATH (group 3 PSN sets)									SCOP (group 3 PSN sets)					
	1.10	1.20	2.30	2.40	2.60	2.160	3.10	3.30	3.40	a.118	b.1	c.1	c.23	c.26	c.55
1	**87.29**	**78.01**	87.93	77.24	78.59	89.28	88.76	74.96	59.97	55.91	58.38	**64.28**	75.42	**56.85**	60.58
2	84.85	77.06	90.17	80.69	78.2	**91.58**	90.38	77.04	62.11	58.22	64.94	62.51	81.87	55.35	58.5
3	82.21	74.42	90.55	**81.93**	80.31	85.85	91.77	80.6	63.94	61.09	66.44	63.15	84.08	55.92	61.73
4	82.7	72.12	**91.69**	81.51	81.68	80.9	94.16	80.56	64.46	**68.06**	64.45	61.71	84.01	55.11	64.16
5	84.08	75.73	90.7	81.34	82.42	75.81	**94.22**	**80.85**	65.81	67.2	65.06	61.18	83.29	54.2	64.52
6	83.62	76.05	88.82	80.29	82.93	72.48	93.54	80.71	67.16	66.87	65.44	61.2	83.01	53.02	**65.41**
7	82.23	75.86	87.41	80.46	**83.32**	74.99	92.98	80.59	68.19	65.64	65.96	60.22	82.7	52.54	65.01
8	81.16	76.56	86.92	80.81	83.31	73.72	92.62	79.8	68.55	61.59	**67.34**	58.82	80.48	52.75	64.53
9	79.33	76.65	86.85	81.63	83.21	72.81	91.85	78.71	**68.91**	58.37	67.12	57.94	78.53	52.14	63.57
10	78.58	77.11	86.55	81.89	82.61	67.02	90.27	78.11	68.75	58.17	66.15	57.23	77.53	52.69	61.45
15	75.86	75.36	82.79	80.88	76.61	57.39	84.52	72.73	62.25	50.85	63.6	57.65	77.11	53.71	58.58
20	76.31	72.65	76.56	77.08	71.35	51.18	83.18	69.63	61.39	49.7	60.95	55.3	**84.32**	55.87	59.26
25	76.15	71.81	70.09	75.68	66.85	51.91	81.75	67.89	60.92	53.71	62.86	55.51	75.23	52.28	59.36
30	73.45	69.39	68.63	72.15	62.22	51.37	77.12	67.19	57.36	51.5	62.78	57.76	71.1	52.18	65.01
35	66.05	64.94	67.68	69.61	66.19	50.87	72.49	66.61	53.1	50.71	60.27	60.09	61.6	52.17	64.65

Supplementary Table S23. Accuracy of the NormOrderedGraphlet-3-4(K) approach when varying the value of K, with respect to **AUPR values** (expressed as percentages), corresponding to the PSN sets of **group 4**. Given a fourth-level PSN data set (within a given column), the AUPR for the "best" K is shown in bold. These results are with respect to the second PSN construction strategy, **(any heavy atom type, 5 Å distance cut-off)**.

K	CATH (group 4 PSN sets)						SCOP (group 4 PSN sets)			
	2.60.40	2.60.120	3.20.20	3.40.50	3.30.390	3.30.420	b.1.1	c.1.8	c.2.1	c.37.1
1	75.22	59.15	**44.55**	68.08	94.68	69.89	92.15	78.24	74.14	57.07
2	78.57	67.25	42.76	77.15	94.79	66.68	94.85	86.92	72.96	61.45
3	80.59	**67.31**	43.53	84.2	96	70.62	96.26	**88.36**	70.23	65.92
4	80.68	66.19	42.88	84.89	98.5	73.56	96.55	87.03	76.17	68.58
5	81.08	62.11	42.85	85.19	99.24	74.09	96.91	85.35	79.95	70.19
6	81.54	57.75	42.33	85.18	**99.26**	**74.85**	**97.1**	86	80.24	**72.59**
7	81.58	53.35	41.38	**85.3**	99.24	73.92	96.54	82.91	**81.4**	72.03
8	**81.86**	51.1	40.9	84.86	99	74.02	95.63	80.1	80.13	70.73
9	81.35	49.93	39.34	84.21	98.27	73.34	93.89	78.51	79.76	70.75
10	80.75	49.04	38.12	83.45	97.89	72.9	90.35	76.13	80.93	71.5
15	80.52	49.07	37.92	70.39	95.84	70.67	87.82	65.99	78.72	61.41
20	79.47	47.4	37.67	69.08	89.19	69.96	92.39	68.89	62.73	44.45
25	79.17	45.25	36.06	70.41	88.01	66.09	91.43	58.59	62.61	44.52
30	80.21	41.1	35.18	70.14	82.02	66.01	87.66	54.7	52.85	36.6
35	80.83	37.68	34.82	67.48	71.53	66.17	87.13	56.2	50.96	37.98

Supplementary Table S24. Accuracy of the NormOrderedGraphlet-3-4(K) approach when varying the value of K, with respect to **AUROC values** (expressed as percentages), corresponding to the PSN sets of **group 4**. Given a fourth-level PSN data set (within a given column), the AUROC for the "best" K is shown in bold. These results are with respect to the second PSN construction strategy, **(any heavy atom type, 5 Å distance cut-off)**.

K	CATH (group 4 PSN sets)						SCOP (group 4 PSN sets)			
	2.60.40	2.60.120	3.20.20	3.40.50	3.30.390	3.30.420	b.1.1	c.1.8	c.2.1	c.37.1
1	60.42	74.01	**62.1**	63.31	93.47	62.98	88.53	78.82	87.31	75.65
2	65.13	79.9	59.68	74.63	93.32	59.47	92.38	86.9	86.88	81.88
3	68.14	80.52	60.63	82.27	94.82	65.05	94.46	**88.32**	78	85.92
4	68.45	**80.82**	60.49	82.57	98.11	68.09	94.92	86.93	82.87	87.2
5	68.68	79.13	60.52	82.87	99.08	69.33	95.46	85.37	86.47	88.27
6	69.22	75.69	60.22	82.91	**99.09**	**70.47**	**95.72**	86.09	86.76	**89.11**
7	69.34	71.97	59.21	**83**	99.07	69.86	94.83	83.3	88.57	88.58
8	**70.18**	69.43	58.29	82.37	98.76	69.77	93.43	80.09	88	87.74
9	70.17	68.08	57.05	81.42	97.75	68.4	90.58	78.8	88.47	87.59
10	69.36	67.21	56.06	80.3	97.27	66.86	84.89	76.79	**90**	87.72
15	67.55	70.06	55.93	64.2	94.44	64.72	82.32	66	87.85	83.53
20	66.41	70.53	55.56	67.28	85.76	60.86	88.42	70.34	75.8	78.79
25	67.14	67.57	54.58	69.93	84.31	57.03	87.35	58.63	74.84	79.23
30	67.64	65.45	53.74	69.28	77.61	58.6	82.55	53.71	69.26	73.01
35	70.12	63.05	54.33	66.61	65.75	60.46	83.48	54.65	66.48	73.52

Supplementary Table S25. Accuracy of the NormOrderedGraphlet-3-4(K) approach when varying the value of K, with respect to **AUPR values** (expressed as percentages), corresponding to the PSN sets of **"equal size", group 1, and group 2**. Given a PSN data set (within a given column), the AUPR for the "best" K is shown in bold. These results are with respect to the third PSN construction strategy, **(any heavy atom type, 6 Å distance cut-off)**.

	"Equal size" PSN sets			CATH group 1 and group 2 PSN sets				SCOP group 1 and group 2 PSN sets					
K	CATH-95	CATH-99	CATH-251-265	primary	α	β	α/β	primary	α	β	α/β	α+β	Multi domain
1	**93.8243**	**92.611**	**93.3112**	**52.5211**	**52.4408**	47.0419	**50.6321**	**38.5774**	31.0746	44.2166	25.3735	35.6291	78.9629
2	93.083	89.6121	62.6273	48.84	51.6715	46.3239	49.4317	37.4814	29.6531	45.4915	30.04	41.1876	85.5052
3	90.8128	92.436	63.1837	44.475	51.2541	46.513	47.3909	36.7855	30.0529	49.6021	36.237	45.3377	**87.4843**
4	80.0816	81.1256	61.2143	45.378	50.4647	47.2447	45.9051	36.9079	28.6072	51.8583	40.8009	50.5523	86.065
5	74.6997	79.6314	64.6979	48.0665	50.7507	47.5972	46.2874	38.0372	**37.7377**	52.718	43.9872	**51.4498**	84.0811
6	72.7505	78.5	65.3439	48.0391	50.7941	**47.6815**	45.951	37.5217	35.9741	53.6486	**44.7437**	49.2802	80.8049
7	75.0194	79.2788	66.2671	47.4178	50.7153	47.4744	45.1282	36.801	33.8771	**53.9914**	43.4987	46.6642	79.386
8	78.1553	79.8152	63.1784	46.6081	50.6341	47.1979	43.7262	35.9567	32.2978	53.3929	39.0275	44.2809	78.7475
9	80.531	80.8223	59.3312	45.5752	50.738	46.5637	41.4448	35.1269	31.6835	52.4958	33.7866	42.3294	77.8843
10	83.7978	79.9477	60.2262	44.5846	50.7346	45.855	39.0641	34.2358	30.9655	49.6446	29.5087	40.3274	77.4721
15	80.7855	70.8327	59.6969	40.9819	51.1259	43.9279	37.9139	30.6741	25.2963	35.5654	22.7173	34.9358	75.6362
20	76.6726	65.8775	49.5415	39.5711	50.8741	43.1598	38.2163	28.8821	19.9771	30.6734	19.4706	27.0215	76.7871
25	74.9264	62.8909	54.2533	38.7696	50.5394	42.4773	36.4476	28.1911	20.2152	23.9783	18.6826	23.1234	72.0598
30	69.2725	61.9828	53.2498	38.5478	50.6096	43.4612	35.1063	27.5047	21.2468	29.2477	17.5101	18.4619	68.2408
35	65.7627	60.3011	54.6863	38.4975	49.9079	44.149	34.172	27.4887	21.0341	29.3929	16.5785	17.1664	67.3231

Supplementary Table S26. Accuracy of the NormOrderedGraphlet-3-4(K) approach when varying the value of K, with respect to **AUROC values** (expressed as percentages), corresponding to the PSN sets of **"equal size", group 1, and group 2**. Given a PSN data set (within a given column), the AUROC for the "best" K is shown in bold. These results are with respect to the third PSN construction strategy, **(any heavy atom type, 6 Å distance cut-off)**.

	"Equal size" PSN sets			CATH group 1 and group 2 PSN sets				SCOP group 1 and group 2 PSN sets					
K	CATH-95	CATH-99	CATH-251-265	primary	α	β	α/β	primary	α	β	α/β	α+β	Multi domain
1	**94.3234**	**93.6548**	**93.8736**	**64.91**	**53.9657**	**61.6111**	**68.9726**	62.7895	74.2745	82.7252	72.157	82.2374	72.6066
2	94.0394	90.8966	65.6085	60.4247	53.1554	59.5478	66.7013	62.3918	74.1976	82.1325	76.88	84.6676	80.4685
3	91.835	92.6635	63.5199	57.247	51.9577	58.7881	63.8868	62.5036	74.7214	84.2466	80.7576	86.0685	**83.3957**
4	81.4973	82.7985	63.5199	59.3649	51.9834	58.6031	62.0106	62.2454	75.6928	85.3418	82.336	**87.2756**	82.2328
5	76.0206	81.334	63.1857	62.2511	52.1588	58.2527	61.63	**63.1167**	**77.839**	85.5366	**82.9089**	86.9995	79.568
6	73.7216	79.4467	62.0162	62.2196	52.1451	57.5821	61.1592	62.627	77.1334	85.7396	82.2198	86.3243	75.5008
7	76.8992	80.2615	61.8769	61.6995	52.0836	56.7736	60.5554	61.9194	76.0347	**85.7695**	81.0485	85.6102	73.9415
8	81.1237	79.8695	60.3175	61.0752	51.9385	55.9745	59.807	61.0283	75.2087	85.5596	78.5657	84.7613	73.1977
9	83.5859	80.0319	57.1707	60.3149	52.0983	55.3636	58.5924	60.2286	74.692	85.1856	75.3155	83.8778	71.9857
10	86.4846	79.4859	57.1429	59.4911	52.2315	54.99	57.0644	59.3009	74.3267	83.8611	72.3102	82.8704	71.528
15	81.834	73.3591	59.315	56.3619	52.6682	56.5119	56.5174	55.8903	71.0168	76.7889	68.2997	80.2249	68.8666
20	77.4884	66.087	50.4595	55.1045	52.018	56.4949	57.5224	54.7588	66.78	71.9175	67.6035	77.7061	70.8435
25	77.8093	64.0261	53.829	54.3573	51.1548	56.0647	56.0702	53.828	68.3999	66.0127	66.7558	74.6393	65.613
30	70.807	62.864	56.7251	54.1199	50.6631	56.0578	54.9224	53.0708	69.2862	65.8509	64.9267	72.888	62.7215
35	64.4729	55.8048	50.4038	54.2104	50.2092	57.1076	53.7047	53.131	68.4475	69.8933	63.0436	70.1864	58.2962

Supplementary Table S27. Accuracy of the NormOrderedGraphlet-3-4(K) approach when varying the value of K, with respect to **AUPR values** (expressed as percentages), corresponding to the PSN sets of **group 3**. Given a third-level PSN data set (within a given column), the AUPR for the "best" K is shown in bold. These results are with respect to the third PSN construction strategy, **(any heavy atom type, 6 Å distance cut-off)**.

K	CATH (group 3 PSN sets)									SCOP (group 3 PSN sets)					
	1.10	1.20	2.30	2.40	2.60	2.160	3.10	3.30	3.40	a.118	b.1	c.1	c.23	c.26	c.55
1	59.06	54.1	78.19	54.79	80.5	80.11	71.94	43.26	65.42	60.73	67.2	50.75	62.3	**63.98**	64.47
2	**60.74**	**54.29**	83.63	59.43	81.15	**80.19**	74.19	47.37	69.37	61.33	72.28	**52.84**	71.46	61.84	64.73
3	49.55	52.66	85.24	60.75	82.74	74.94	78.94	50.74	71.82	61.36	73.79	52.66	**80.74**	63.43	66.57
4	50.37	45.55	**85.42**	60.53	84.02	72.9	**85**	52.9	72.97	67.34	73.5	51.62	78.55	62.56	71.75
5	60.31	53.32	85.38	61.36	84.51	69.63	84.99	**53.52**	74.7	**69.2**	74.08	51.4	77.49	61.89	72.67
6	59.96	52.92	83.35	60.34	85.01	65.62	84.15	52.43	75.37	67.25	74.21	51.09	74.48	61.19	74.04
7	57.57	52.74	81.32	59.88	**85.21**	67.18	83.1	51.21	**75.87**	65.04	74.31	49.03	70.97	60.71	**75.36**
8	54.62	52.34	79.7	60.31	84.92	67.35	82.11	49.97	75.75	62.21	**74.51**	45.93	67.98	60.33	75.17
9	51.82	52.35	78.32	61.12	84.81	66.04	80.23	48.33	75.06	58.64	73.6	44.63	66.54	60.07	74.43
10	49.47	53.18	77.59	**61.38**	84.08	62.86	77.7	46.94	74.33	57.75	72.1	43.95	65.6	60.85	72.96
15	45.35	50.96	72.18	59.26	79.16	56.82	72.65	40.93	66.76	51.83	67.6	45.65	65.55	61.68	62.66
20	49.08	46.63	64.43	54.65	74.21	51.88	67.19	36.36	61.74	51.93	68.84	42.61	73.91	60.68	60.79
25	47.87	43.35	54.44	52.35	70.47	51.28	61.42	30.06	60.11	54.22	70.61	41.92	63.55	59.57	61.44
30	34.66	38.6	52.48	44.76	69.75	52.54	49.12	27.32	57.67	54.8	72.7	42.93	55.82	58.4	63.74
35	24.75	30.18	42.59	40.91	73.51	54.39	43.23	27.3	53.85	56.97	73.21	45.84	46.78	60.01	64.46

Supplementary Table S28. Accuracy of the NormOrderedGraphlet-3-4(K) approach when varying the value of K, with respect to **AUROC values** (expressed as percentages), corresponding to the PSN sets of **group 3**. Given a third-level PSN data set (within a given column), the AUROC for the "best" K is shown in bold. These results are with respect to the third PSN construction strategy, **(any heavy atom type, 6 Å distance cut-off)**.

K	CATH (group 3 PSN sets)									SCOP (group 3 PSN sets)					
	1.10	1.20	2.30	2.40	2.60	2.160	3.10	3.30	3.40	a.118	b.1	c.1	c.23	c.26	c.55
1	87.38	79.19	88.66	77.76	78.28	79.4	90.58	76.91	60.64	57.74	57.5	**62.19**	73.41	**58.32**	61.56
2	**88.39**	77.81	91.54	81.83	78.49	**79.53**	91.43	78.88	67.4	58.59	63.27	61.69	80.75	56.18	63.11
3	84.14	75.36	92.27	**83.05**	80.32	73.47	91.47	80.62	68.92	61.49	64.55	61.37	**87.29**	57.01	66.41
4	83.95	72.21	92.46	81.92	81.67	70.59	94.46	80.5	69.24	**67.64**	63.48	60.6	85.27	56.07	70.65
5	85.07	78.32	**92.61**	81.35	82.31	66.91	**94.6**	**81.07**	71.2	67.05	63.6	60.21	84.63	55.07	72.02
6	84.57	78.49	91.47	79.84	82.93	62.88	94.16	80.94	72	64.91	63.94	60.24	82.47	54.04	73.68
7	83.43	79.06	90.33	79.48	**83.26**	66	93.65	80.67	**72.8**	62.49	64.38	59.24	79.71	53.87	**75.05**
8	82.4	**79.25**	89.29	80.24	83.06	68.08	93.24	80.21	72.54	60.25	65.22	56.76	76.86	53.56	74.82
9	81.05	78.97	88.34	81.15	82.94	68.83	92.36	79.55	71.67	57.57	65.18	57.31	75.36	53.82	74.03
10	80.47	79.24	87.68	81.8	82.3	65.19	90.71	78.94	70.66	56.96	64.09	57.15	74.23	55.27	72.36
15	80.01	77.47	83.41	81.9	76.64	56.3	86.94	73.84	60.98	50.69	58.11	57.23	75.7	55.81	60.04
20	81.28	74.35	78.04	78.35	70.16	50.35	85.73	70.29	59.33	49.99	59.39	55.87	83.28	55.14	57.76
25	82.13	73	72.4	77.57	66.32	49.55	84	68.23	58.13	51.73	61.44	55.9	75.95	52.49	58.99
30	77.05	70.65	70.57	73.77	63.17	50.36	79.8	68.26	54.9	51.02	**65.75**	58.18	69.35	51.66	62.15
35	68.09	66.81	66.32	71.01	67.15	51.27	73.81	67.76	50.96	50.94	65.5	60.16	59.84	53.05	63.02

Supplementary Table S29. Accuracy of the NormOrderedGraphlet-3-4(K) approach when varying the value of K, with respect to **AUPR values** (expressed as percentages), corresponding to the PSN sets of **group 4**. Given a fourth-level PSN data set (within a given column), the AUPR for the "best" K is shown in bold. These results are with respect to the third PSN construction strategy, **(any heavy atom type, 6 Å distance cut-off)**.

K	CATH (group 4 PSN sets)						SCOP (group 4 PSN sets)			
	2.60.40	2.60.120	3.20.20	3.40.50	3.30.390	3.30.420	b.1.1	c.1.8	c.2.1	c.37.1
1	78.77	58.35	42.55	72.07	86.44	69.61	93.7	80.09	78.36	62.05
2	81.73	64.55	43.91	82.43	89.36	72.17	94.79	86.97	75.87	69.73
3	82.8	**65.27**	**44.04**	87.82	91.75	75.76	94.83	**87.14**	77.13	76.65
4	83	65.23	43.54	88.36	96.81	80.81	95.21	86.02	81.23	79.09
5	83.08	61.61	43.49	**88.62**	98.66	82.01	96.03	84.01	**82.6**	81.94
6	83.61	57.72	43.13	88.08	98.7	83.74	**96.37**	84.65	82.07	82.9
7	83.73	53.88	41.68	87.5	**98.74**	**84.01**	96.02	81.2	82.16	**82.97**
8	84.05	52.38	40.43	87.08	98.45	83.72	95.61	78.13	80.73	82
9	**84.13**	51.14	38.97	86.6	97.91	83.23	94.59	74.73	79.88	81
10	83.79	50.29	38.4	86.2	97.8	81.98	92.64	73.43	80.98	80.24
15	81.45	51	40.48	74.34	95.62	74.53	91.63	68.38	80.29	63.04
20	81.18	50.05	39.74	74.7	90.15	70.25	94.49	70.4	62.62	45.46
25	81.38	46.65	38.32	76.33	88.01	66.57	93.94	62.63	65.22	48.75
30	82.17	42.64	37.03	72.55	84.49	62.9	88.05	57.43	57.14	40.55
35	83.38	40.19	36.81	69.88	72.06	62.88	92.07	59.23	51.77	40.89

Supplementary Table S30. Accuracy of the NormOrderedGraphlet-3-4(K) approach when varying the value of K, with respect to **AUROC values** (expressed as percentages), corresponding to the PSN sets of **group 4**. Given a fourth-level PSN data set (within a given column), the AUROC for the "best" K is shown in bold. These results are with respect to the third PSN construction strategy, **(any heavy atom type, 6 Å distance cut-off)**.

K	CATH (group 4 PSN sets)						SCOP (group 4 PSN sets)			
	2.60.40	2.60.120	3.20.20	3.40.50	3.30.390	3.30.420	b.1.1	c.1.8	c.2.1	c.37.1
1	64.04	74.08	60.22	66.17	82.49	63.15	90.37	80.34	89.25	78.47
2	68.49	78.48	60.97	79.68	86.1	65.82	92.07	86.92	88.34	86.48
3	70.28	79.31	**61.4**	86.11	89.52	71.18	92.01	**87.03**	83.96	90.87
4	70.85	**79.51**	60.87	86.55	95.93	76.01	92.7	85.68	86.6	91.93
5	70.86	77.68	60.87	**86.56**	98.4	78.82	94.09	83.81	88.39	93.22
6	71.74	74.45	60.33	85.78	98.44	81.48	**94.63**	84.62	88.28	**93.34**
7	71.94	71.21	58.91	85.07	**98.5**	**82.15**	94.07	81.11	89.06	92.94
8	72.74	69.39	57.42	84.24	98.09	81.84	93.39	77.11	88.37	92.53
9	**73.29**	68.2	56.7	83.15	97.33	80.45	91.93	74.41	88.13	91.85
10	72.85	67.41	56.22	82.39	97.15	78.73	89.06	73.67	**89.43**	91.16
15	67.16	71.45	58.07	68	94.14	70.17	87.51	67.71	88.33	85.02
20	68.19	71.74	57.52	72.08	86.09	61.87	91.8	70.93	74.08	79.87
25	69.05	68.98	56.15	75.37	83.82	59.14	91.53	61.71	76.65	81.29
30	69.37	66.3	54.88	71.33	79.9	56.05	83.37	57.37	71.62	75.72
35	72.39	64.57	55.51	68.4	66.39	55.65	88.76	59.67	66.66	75.36

Supplementary Table S31. Accuracy of the NormOrderedGraphlet-3-4(K) approach when varying the value of K, with respect to **AUPR values** (expressed as percentages), corresponding to the PSN sets of **"equal size", group 1, and group 2**. Given a PSN data set (within a given column), the AUPR for the "best" K is shown in bold. These results are with respect to the fourth PSN construction strategy, (**α-carbon heavy atom type, 7.5 Å distance cut-off**).

	"Equal size" PSN sets			CATH group 1 and group 2 PSN sets				SCOP group 1 and group 2 PSN sets					
K	CATH-95	CATH-99	CATH-251-265	primary	α	β	α/β	primary	α	β	α/β	α+β	Multi domain
1	89.8215	85.0468	72.6334	48.394	50.7576	41.8728	40.9176	34.131	21.1685	28.4896	18.6503	21.6993	76.3279
2	89.8215	85.0468	71.746	**49.6821**	50.8409	42.4335	**46.3839**	33.7447	**27.3273**	30.0255	35.6719	37.3763	82.6743
3	87.9752	**88.6577**	72.6334	47.1422	50.8577	42.244	43.8359	33.3577	21.7516	31.0554	40.6176	37.7967	81.5683
4	85.3515	85.0252	75.3496	48.394	**50.9556**	**42.7641**	42.6236	33.683	26.5447	**31.5721**	**41.354**	**38.3998**	80.6637
5	82.7428	75.6205	74.9868	49.1069	50.7576	42.4553	42.4582	34.0212	25.0325	31.0944	40.7564	38.3417	80.1001
6	91.3784	79.3631	**75.6967**	49.4175	50.7126	41.8728	42.0466	34.3185	22.9606	30.7778	38.9241	37.5164	77.5144
7	**94.0243**	79.9115	74.3017	49.5053	50.4801	41.3762	40.9176	**34.4718**	22.2192	30.2087	35.1552	36.732	77.7576
8	93.9675	81.2546	74.2993	49.2268	50.4483	40.9973	39.6863	34.131	21.8231	29.6348	29.4325	36.0329	80.9981
9	93.2845	80.9277	69.3683	48.6194	50.3754	40.5913	37.7284	33.6661	21.1685	29.2442	24.0549	34.4269	81.2322
10	90.6966	80.3671	69.2988	47.7634	50.1442	40.1459	35.5678	32.9059	20.1706	28.4896	20.1053	32.4296	**83.2181**
15	89.9537	76.3192	70.9902	42.6924	50.0231	38.7087	36.3341	29.99	17.3105	27.8789	18.6503	23.7081	82.8044
20	88.7191	65.8203	66.0019	41.5087	49.6305	37.5869	37.8245	29.8656	16.9888	22.3999	17.6461	21.6993	79.9464
25	84.3599	62.4657	63.5928	41.6779	50.0707	37.6404	36.2046	30.6133	17.4211	19.8937	18.2314	22.4718	76.3279
30	76.3017	62.4005	61.6644	42.8473	50.0814	39.6575	35.438	31.4873	17.2376	26.9078	16.7607	22.324	67.976
35	79.2573	66.898	68.9738	43.4129	50.2873	38.9433	35.5941	31.4582	18.0783	25.0686	16.4576	21.184	68.1579

Supplementary Table S32. Accuracy of the NormOrderedGraphlet-3-4(K) approach when varying the value of K, with respect to **AUROC values** (expressed as percentages), corresponding to the PSN sets of **"equal size", group 1, and group 2**. Given a PSN data set (within a given column), the AUROC for the "best" K is shown in bold. These results are with respect to the fourth PSN construction strategy, (**α-carbon heavy atom type, 7.5 Å distance cut-off**).

	"Equal size" PSN sets			CATH group 1 and group 2 PSN sets				SCOP group 1 and group 2 PSN sets					
K	CATH-95	CATH-99	CATH-251-265	primary	α	β	α/β	primary	α	β	α/β	α+β	Multi domain
1	89.7131	84.6327	66.4147	63.9955	52.2025	55.3577	58.6221	59.8756	65.7058	74.1893	66.0626	75.1261	71.0309
2	89.7131	84.6327	69.2829	62.9797	51.9384	**58.3955**	**62.3037**	59.3067	71.0702	71.4766	79.7506	83.5443	76.2843
3	87.9525	**88.7237**	66.4147	61.0325	51.5474	57.2819	60.8592	59.2313	69.6389	72.698	**82.7688**	85.8659	75.3403
4	85.0026	83.9937	70.9534	63.9955	52.0396	57.449	59.9382	60.0542	**71.5534**	73.5371	82.6532	**85.8847**	75.0983
5	82.112	73.7105	72.8337	64.6079	52.2025	56.6177	59.7157	60.3849	69.6323	73.5854	82.1436	85.4359	74.4079
6	91.0421	77.2273	**73.3405**	**64.6791**	**52.395**	55.3577	59.397	**60.4897**	67.5968	73.7412	80.7629	84.3996	72.1252
7	**94.1605**	77.7829	71.4773	64.4807	52.2541	54.1979	58.6221	60.395	66.3619	74.084	78.428	83.3323	72.3226
8	94.1381	78.5196	68.3241	64.0713	52.2589	53.2247	57.7474	59.8756	66.0592	74.1491	74.6266	82.1533	75.9229
9	93.4056	78.5178	63.7163	63.4109	52.1926	52.5209	56.4735	59.2634	65.7058	**74.2652**	70.3463	80.661	76.5426
10	90.9991	77.6419	61.7985	62.5293	52.0517	52.1799	54.8142	58.3862	64.9918	74.1893	66.822	79.3437	79.9398
15	91.3567	73.4562	65.4874	58.108	51.6526	52.2793	55.3809	55.4423	62.7582	72.4605	66.0626	74.934	**80.5012**
20	90.1027	62.2525	59.7872	57.0161	51.1703	51.2372	56.4688	55.509	62.204	62.1358	67.0804	75.1261	76.7643
25	85.0486	57.4064	54.8634	57.458	51.3228	51.7993	55.5054	56.4704	61.496	59.4312	68.2709	74.8694	71.0309
30	75.2758	59.3654	51.7519	58.9919	51.3845	54.5892	55.5273	57.767	60.8944	69.8774	66.3345	74.6705	59.1623
35	79.4286	63.2044	54.6589	60.0369	51.7219	54.6584	56.1945	58.3489	60.136	71.5998	64.0878	72.7887	57.656

Supplementary Table S33. Accuracy of the NormOrderedGraphlet-3-4(K) approach when varying the value of K, with respect to **AUPR values** (expressed as percentages), corresponding to the PSN sets of **group 3**. Given a third-level PSN data set (within a given column), the AUPR for the "best" K is shown in bold. These results are with respect to the fourth PSN construction strategy, **(α-carbon heavy atom type, 7.5 Å distance cut-off)**.

K	CATH (group 3 PSN sets)									SCOP (group 3 PSN sets)					
	1.10	1.20	2.30	2.40	2.60	2.160	3.10	3.30	3.40	a.118	b.1	c.1	c.23	c.26	c.55
1	46.0916	**39.7028**	69.2343	55.5609	80.3951	65.0876	72.7146	43.7696	73.8642	66.6464	67.5527	40.2467	49.0343	59.7973	69.8496
2	43.3688	**39.7028**	69.9726	**58.782**	76.8226	**69.315**	71.4327	39.841	64.8448	64.7561	68.7363	**49.0905**	70.0794	66.1884	65.4888
3	47.8127	37.8907	69.2343	56.8414	77.8693	68.9932	73.7499	44.7918	68.6112	69.2514	**69.6515**	47.8989	72.4919	64.8403	67.8022
4	**53.6925**	35.4562	**71.1834**	55.5609	79.1897	68.8464	**75.2239**	43.611	70.959	**79.2**	67.9925	47.1533	**73.0792**	66.0309	68.6016
5	46.2587	34.3776	68.8933	54.9947	80.3951	68.0242	75.13	44.6438	71.9209	76.3134	67.7848	45.623	70.1648	65.3382	69.388
6	40.1154	33.6902	66.0743	55.0984	81.3657	65.0876	72.5966	**45.2782**	72.7802	73.4897	67.8568	44.1904	68.4677	64.8893	71.2102
7	38.2264	31.7325	64.1737	54.3849	81.7367	62.9294	72.7146	44.8749	73.7837	71.4872	68.0807	42.3212	67.7835	64.9444	71.9087
8	37.5012	30.0784	62.6794	51.9485	82.0811	62.1832	72.1955	43.7696	**73.9243**	68.4131	67.6824	40.9682	66.9202	65.5765	72.7923
9	36.3267	30.212	60.2473	50.2244	**82.1952**	65.5203	70.3824	42.0077	73.8642	67.657	67.4791	41.3557	66.5362	65.1489	73.3078
10	34.5602	30.574	57.2738	49.3623	81.9417	67.1015	69.5277	39.7914	73.4347	66.6464	67.0742	41.8612	67.3492	64.2347	**73.682**
15	31.0358	29.8486	55.2761	52.3889	78.3932	61.07	60.2524	32.4862	65.175	54.9985	67.5527	41.7313	64.7558	**69.3576**	61.3926
20	32.014	28.1603	50.1829	50.2426	72.151	54.8006	55.4569	28.3508	57.0169	54.5741	65.5314	40.2467	65.5352	64.0077	58.4955
25	23.2843	26.5084	47.1894	42.232	71.646	52.6113	55.319	27.2226	58.5202	55.1513	65.1489	37.5869	49.0343	58.81	58.2156
30	20.9455	26.1624	39.6379	37.6214	77.2724	54.1213	47.536	28.0697	62.3987	56.7455	69.1568	38.5811	47.4656	59.7973	62.7687
35	19.0415	23.7553	38.1365	35.9572	79.0743	54.6481	49.7884	32.6485	61.4259	59.4486	65.3159	38.6687	43.5351	57.2755	69.8496

Supplementary Table S34. Accuracy of the NormOrderedGraphlet-3-4(K) approach when varying the value of K, with respect to **AUROC values** (expressed as percentages), corresponding to the PSN sets of **group 3**. Given a third-level PSN data set (within a given column), the AUROC for the "best" K is shown in bold. These results are with respect to the fourth PSN construction strategy, **(α-carbon heavy atom type, 7.5 Å distance cut-off)**.

K	CATH (group 3 PSN sets)									SCOP (group 3 PSN sets)					
	1.10	1.20	2.30	2.40	2.60	2.160	3.10	3.30	3.40	a.118	b.1	c.1	c.23	c.26	c.55
1	80.6252	**71.1042**	82.2756	82.5861	79.103	61.0412	92.3114	76.5267	70.9949	64.2875	59.4314	53.709	64.2999	54.2062	65.3512
2	82.9429	**71.1042**	**83.4385**	**84.7758**	74.6753	65.4227	90.6548	74.1814	61.3462	60.6374	60.0984	**60.334**	78.9265	60.1495	64.0804
3	82.6379	66.4547	82.2756	84.0638	76.1017	65.0116	91.8333	77.632	64.5613	66.572	60.6746	58.971	80.7483	57.8496	67.6216
4	**84.7524**	68.8234	82.9248	82.5861	77.6834	65.6596	92.6199	**77.9001**	67.2287	**79.9991**	58.5417	58.3265	**81.6103**	58.9701	69.1003
5	81.5899	67.9714	81.6121	81.2539	79.103	64.3586	**92.6684**	77.6504	68.5711	76.2329	58.1494	56.4628	79.8738	59.345	69.9354
6	78.157	66.9362	80.0918	80.083	80.2609	61.0412	92.575	77.5869	69.8086	73.3477	57.8532	55.5382	78.693	58.9237	72.165
7	77.2532	65.4763	79.1128	78.7065	80.7062	61.4188	92.3114	76.9912	70.9698	70.5757	58.0656	54.4445	77.9134	58.8856	72.8045
8	77.192	64.6771	78.729	77.869	80.9864	63.097	91.4599	76.5267	**71.1325**	67.4516	57.8191	53.9088	77.1369	59.7171	73.4406
9	76.0846	65.7267	77.209	77.5058	**81.0362**	69.1658	89.7169	76.2834	70.9949	66.2638	57.6661	54.9587	77.1856	58.9744	**74.1793**
10	74.5253	67.0002	75.8441	77.887	80.8367	**71.4426**	87.3597	75.959	70.6736	64.2875	57.2558	55.8337	77.8355	55.5303	74.1249
15	72.5521	66.2437	73.5604	80.2763	76.6071	62.6878	80.4785	71.3543	62.436	52.542	59.4314	51.8184	74.4679	**62.9107**	60.0816
20	72.7055	63.7486	69.3866	76.1989	66.4357	56.2391	78.9998	67.347	56.1336	52.544	55.0221	53.709	77.9979	60.897	56.6613
25	65.2217	62.0126	67.4325	69.5173	65.6766	50.4842	80.7934	66.7517	55.1022	51.3573	54.712	50.7924	64.2999	52.6194	55.7737
30	62.026	62.2825	63.7329	64.3973	72.5406	50.4791	78.438	67.4916	59.1377	50.2697	**64.3013**	51.8399	62.5962	54.2062	59.9419
35	60.2107	59.9327	62.565	63.9053	75.7867	49.3556	77.1572	71.4792	59.5378	50.1626	61.3346	52.4292	59.1356	52.4369	65.3512

Supplementary Table S35. Accuracy of the NormOrderedGraphlet-3-4(K) approach when varying the value of K, with respect to **AUPR values** (expressed as percentages), corresponding to the PSN sets of **group 4**. Given a fourth-level PSN data set (within a given column), the AUPR for the "best" K is shown in bold. These results are with respect to the fourth PSN construction strategy, **(α-carbon heavy atom type, 7.5 Å distance cut-off)**.

K	CATH (group 4 PSN sets)						SCOP (group 4 PSN sets)			
	2.60.40	2.60.120	3.20.20	3.40.50	3.30.390	3.30.420	b.1.1	c.1.8	c.2.1	c.37.1
1	75.8816	**58.4616**	38.3153	82.5749	**95.6567**	75.3712	94.5096	72.0735	65.3061	68.9179
2	77.4575	**58.4616**	38.4069	79.2979	89.1637	68.7707	94.5592	**82.0271**	**73.833**	63.4718
3	78.1237	53.3269	38.3153	82.9614	90.2578	71.2026	94.8726	78.518	66.2737	**70.7923**
4	77.357	51.6812	38.2809	82.5749	94.2561	72.8214	**95.1139**	74.6519	64.3522	70.3898
5	77.3085	48.7674	38.41	84.0702	**95.6567**	73.4983	94.9448	73.4411	65.779	70.579
6	76.8425	46.7566	37.8846	**84.3196**	95.455	75.3712	94.7227	77.135	65.9218	70.003
7	76.7179	44.5734	37.7586	83.6889	94.7839	77.2121	94.5096	72.7784	65.6656	68.5189
8	76.6539	42.8106	37.5188	82.5145	93.6648	78.6272	94.2758	72.0735	64.3496	70.2163
9	76.5323	41.7292	37.0371	82.0808	91.7268	**79.3401**	93.9296	68.3247	65.3061	69.397
10	76.6195	40.6379	37.1898	81.5177	89.7123	78.4422	93.5372	64.544	70.2346	68.9179
15	77.0842	40.6017	**39.0024**	69.7073	90.4561	68.5072	94.6486	57.9315	72.789	52.8262
20	75.4496	41.1356	35.7325	70.2733	85.294	65.9541	92.5017	59.4144	47.6911	37.0716
25	74.7763	40.6549	35.0491	73.0619	82.574	64.9274	92.0631	58.0545	54.2348	41.7487
30	**81.0489**	41.7773	35.5231	75.4411	74.3654	63.0755	78.9102	57.954	52.4251	34.9562
35	76.1956	38.748	34.9793	71.4136	84.3921	70.1389	78.0406	62.194	55.4002	35.2848

Supplementary Table S36. Accuracy of the NormOrderedGraphlet-3-4(K) approach when varying the value of K, with respect to **AUROC values** (expressed as percentages), corresponding to the PSN sets of **group 4**. Given a fourth-level PSN data set (within a given column), the AUROC for the "best" K is shown in bold. These results are with respect to the fourth PSN construction strategy, **(α-carbon heavy atom type, 7.5 Å distance cut-off)**.

K	CATH (group 4 PSN sets)						SCOP (group 4 PSN sets)			
	2.60.40	2.60.120	3.20.20	3.40.50	3.30.390	3.30.420	b.1.1	c.1.8	c.2.1	c.37.1
1	59.6262	**75.4168**	56.3844	79.6051	**94.4527**	72.9606	91.7523	68.3907	78.0299	87.502
2	61.6358	**75.4168**	56.5127	76.5524	87.773	61.6226	92.1235	**81.4888**	**86.6865**	83.8308
3	62.2508	73.5024	56.3844	80.2401	88.4226	65.3557	92.6377	77.1793	73.7954	87.8295
4	60.581	73.1772	55.9068	79.6051	92.6236	68.7166	**92.8624**	72.8967	72.6324	88.3155
5	59.9998	70.9497	55.6171	**81.193**	**94.4527**	69.8623	92.7261	72.1963	74.8107	88.6798
6	59.4035	68.5182	54.1245	81.0558	94.2025	72.9606	92.2544	76.0909	76.0754	88.7495
7	59.1207	66.137	53.2779	80.1676	93.3004	75.6936	91.7523	69.8776	76.7365	88.354
8	58.871	63.855	53.4381	78.476	91.7577	77.2096	91.3702	68.3907	76.1525	**88.7526**
9	58.4667	62.2822	54.3596	77.3915	89.3195	**77.6995**	90.859	65.1355	78.0299	88.1902
10	58.8507	61.0788	55.5587	76.6597	86.6487	75.993	90.251	59.5838	82.2601	87.502
15	61.7305	63.2537	**56.609**	62.7989	89.0893	62.5776	91.9337	55.1386	81.7367	80.8892
20	60.52	64.4804	55.32	64.8864	81.6278	59.5474	88.0476	56.0015	60.4111	68.466
25	60.6953	63.6866	54.3543	68.7153	80.6553	59.6765	88.0363	57.0792	67.44	71.1506
30	**72.4831**	63.8646	54.5909	73.2522	68.8651	54.1386	71.8273	57.5216	66.7307	66.7725
35	66.3531	63.7919	54.3318	68.046	79.367	61.295	71.7888	61.5529	70.2028	69.7624

References

1. Berman, H. M. *et al.* The Protein Data Bank. *Nucleic Acids Research* **28**, 235–242 (2000).
2. Sillitoe, I. *et al.* CATH: comprehensive structural and functional annotations for genome sequences. *Nucleic Acids Research* **43**, D376–D381 (2015).
3. Orengo, C. A. *et al.* The CATH database provides insights into protein structure/function relationships. *Nucleic Acids Research* **27**, 275–279 (1999).
4. Murzin, A. G., Brenner, S. E., Hubbard, T. & Chothia, C. SCOP: a structural classification of proteins database for the investigation of sequences and structures. *Journal of Molecular Biology* **247**, 536–540 (1995).
5. Milenković, T., Lai, J. & Pržulj, N. GraphCrunch: a tool for large network analyses. *BMC Bioinformatics* **9** (2008).
6. Kuchaiev, O., Stevanović, A., Hayes, W. & Pržulj, N. GraphCrunch 2: Software tool for network modeling, alignment and clustering. *BMC Bioinformatics* **12** (2011).
7. Malod-Dognin, N. & Pržulj, N. GR-Align: fast and flexible alignment of protein 3D structures using graphlet degree similarity. *Bioinformatics* **30**, 1259–65 (2014).
8. Pržulj, N. Biological network comparison using graphlet degree distribution. *Bioinformatics* **23**, e177–e183 (2007).
9. Pržulj, N., Corneil, D. G. & Jurisica, I. Modeling interactome: Scale-free or geometric? *Bioinformatics* **20**, 3508–3515 (2004).
10. Yaveroglu, O. N. *et al.* Revealing the Hidden Language of Complex Networks. *Scientific Reports* **4**, 4547 (2014).
11. Vacic, V., Iakoucheva, L. M., Lonardi, S. & Radivojac, P. Graphlet Kernels for Prediction of Functional Residues in Protein Structures. *Journal of Computational Biology* **17**, 55–72 (2010).
12. Lugo-Martinez, J. & Radivojac, P. Generalized graphlet kernels for probabilistic inference in sparse graphs. *Network Science* **2**, 254–276 (2014).
13. Pabuwal, V. & Li, Z. Network pattern of residue packing in helical membrane proteins and its application in membrane protein structure prediction. *Protein Engineering, Design and Selection* **21**, 55–64 (2008).
14. Pabuwal, V. & Li, Z. Comparative analysis of the packing topology of structurally important residues in helical membrane and soluble proteins. *Protein Engineering, Design and Selection* **22**, 67–73 (2009).
15. Gao, J. & Li, Z. Conserved network properties of helical membrane protein structures and its implication for improving membrane protein homology modeling at the twilight zone. *Journal of Computer-Aided Molecular Design* **23**, 755–763 (2009).
16. Emerson, I. A. & Gothandam, K. M. Network analysis of transmembrane protein structures. *Physica A* **391**, 905–916 (2012).
17. Emerson, I. A. & Gothandam, K. M. Residue centrality in alpha helical polytopic transmembrane protein structures. *Journal of Theoretical Biology* **309**, 78–87 (2013).
18. Newman, M. E. J. Assortative mixing in networks. *Physical Review Letters* **89**, 208701 (2002).
19. Holm, L. & Rosenström, P. Dali server: conservation mapping in 3D. *Nucleic Acids Research* **38**, W545–W549 (2010).
20. Zhang, Y. & Skolnick, J. TM-align: a protein structure alignment algorithm based on the TM-score. *Nucleic Acids Research* **33**, 2302–09 (2005).
